# Lactate-mediated cholesterol uptake promotes liver cancer progression via the SCARB1-autophagy axis

**DOI:** 10.1038/s44319-026-00829-x

**Published:** 2026-06-10

**Authors:** Jiamin Chen, Mingyue Zhao, Guanxu Ji, Kecheng Liu, Shengqi Shen, Shi-Ting Li, Jin Cai, Linchong Sun, Jin Li, Ping Gao, Tong Zhang

**Affiliations:** 1 Department of Pathology and Institute of Molecular Pathology, The First Affiliated Hospital, The MOE Basic Research and Innovation Center for the Targeted Therapeutics of Solid Tumors, Jiangxi Medical College, Nanchang University, Nanchang, China; 2 School of Biomedical Sciences and Engineering, South China University of Technology, Guangzhou International Campus, Guangzhou, China; 3 Medical Research Institute, Guangdong Provincial People’s Hospital, Guangdong Academy of Medical Sciences, Southern Medical University, Guangzhou, China; 4 Oncology Department, The First Affiliated Hospital of Guangzhou Medical University, Guangzhou, China; 5 Department of General Surgery, The First Affiliated Hospital of USTC, Division of Life Sciences and Medicine, University of Science and Technology of China, Hefei, China; 6 Frontiers Medical Center, Tianfu Jincheng Laboratory, Chengdu, China

**Keywords:** Cancer, Metabolism, Molecular Biology of Disease

## Abstract

Metabolic reprogramming, including enhanced glycolysis and altered fatty acid metabolism, supports the proliferation of cancer cells under hypoxic stress. However, the mechanism underlying the regulation of cholesterol metabolism under hypoxic stress remains incompletely understood. Here, we report that lactate-induced cholesterol accumulation activates mammalian target of rapamycin complex 1 (mTORC1) signalling under hypoxic conditions, thereby promoting hepatocellular carcinoma (HCC) progression. Mechanistically, lactate upregulates scavenger receptor class B type 1 (SCARB1) expression by increasing histone H3 lysine 18 lactylation (H3K18la), leading to increased cholesterol levels. We further demonstrate that SCARB1-mediated cholesterol uptake is essential for the activation of mTORC1, which promotes tumour growth by preventing excessive autophagy in HCC cells. Importantly, analysis of clinical HCC samples reveals a positive correlation between H3K18la expression and SCARB1 expression. Taken together, these findings provide novel insights into hypoxia-driven metabolic reprogramming and reveal a previously unrecognized connection between lactate and cholesterol metabolism, suggesting a potential innovative cancer therapy for HCC.

## Synopsis

Lactate-induced histone lactylation upregulates SCARB1, enhancing cholesterol uptake. The increase of cholesterol activates mTORC1 signaling, thereby suppressing excessive autophagy and contributing to HCC progression.

- Lactate enhances cholesterol uptake in HCC cells under hypoxia.
- Lactate promotes SCARB1 expression through histone H3K18 lactylation.
- SCARB1-mediated cholesterol uptake activates mTORC1 signalling to suppress autophagy.
- Increased SCARB1 expression in human HCC predicts poor prognosis.

## Abstract (web summary)

Lactate-induced histone lactylation upregulates SCARB1, enhancing cholesterol uptake. The increase of cholesterol activates mTORC1 signaling, thereby suppressing excessive autophagy and contributing to HCC progression.

## Graphical abstract

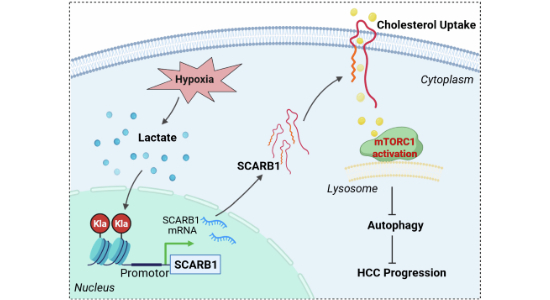

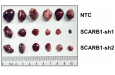

## Introduction

Metabolic reprogramming has been defined as a core hallmark of cancer (Ward and Thompson, 2012). The scope of cancer cell metabolism has expanded far beyond the original observation of aerobic glycolysis, the so-called Warburg effect. In addition to the well-known alterations in glucose metabolism, our group has previously demonstrated several other key metabolic reprogramming processes that critically drive tumour progression (Li et al, 2020a; Sun et al, 2023; Wei et al, 2025; Yan et al, 2024; Zhang et al, 2022). In addition, cancer cells also reprogram cholesterol metabolism to support membrane biosynthesis, signal transduction, and rapid proliferation (Huang et al, 2020; Riscal et al, 2021). This process requires both enhanced de novo biosynthesis and increased exogenous uptake. Previous studies have demonstrated that the master transcription factor Sterol regulatory element-binding proteins (SREBPs), along with their downstream targets (including enzymes in the mevalonate pathway), are significantly upregulated to promote de novo cholesterol biosynthesis in tumour cells under conditions such as ER stress, low pH, and inflammatory regulation (He et al, 2017; Huang et al, 2020; Kondo et al, 2017). However, cholesterol biosynthesis is highly energy-intensive and requires numerous enzymatic reactions and substantial inputs of acetyl-CoA, ATP, oxygen, and NADPH. Consequently, this pathway is tightly regulated (Luo et al, 2020). In contrast to time- and energy-consuming de novo cholesterol synthesis, tumour cells can alternatively maintain elevated intracellular cholesterol levels by increasing exogenous cholesterol uptake. For instance, SCARB1 upregulation in clear cell renal cell carcinoma and low-density lipoprotein receptor (LDLR) upregulation in diffuse large B-cell lymphoma serve as compensatory mechanisms when cholesterol biosynthesis is suppressed (Garcia-Bermudez et al, 2019; Riscal et al, 2021). These findings raise the fundamental question of how tumour cells adapt to environmental stress by balancing the high metabolic cost of cholesterol biosynthesis with the necessity of maintaining cholesterol homeostasis.

The Warburg effect describes the preference of cancer cells for aerobic glycolysis over oxidative phosphorylation, leading to a substantial accumulation of lactate within cancer cells (Ippolito et al, 2019). While initial research focused on the role of lactate in the tumour microenvironment (TME) via the glycolytic pathway, accumulating evidence has underscored the pivotal role of lactate in regulating diverse biological processes (Chen et al, 2025; Li et al, 2022). Indeed, far from being a minor byproduct of glucose utilization in anaerobic environments, lactate can serve as a substrate for aerobic cancer cells (Faubert et al, 2017; Guillaumond et al, 2013; Hensley et al, 2016; Kennedy et al, 2013). Moreover, lactate profoundly shapes the immune landscape. For instance, lactate dehydrogenase A (LDHA)-associated lactate accumulation inhibits tumour surveillance by disrupting the effector functions of T and NK cells, thereby facilitating tumour immune escape (Brand et al, 2016). Beyond this, the discovery of histone lysine lactylation (Kla) established a direct link between cellular metabolic state and gene regulation, with lactate acting as an epigenetic signal that shapes the tumor microenvironment (Zhang et al, 2019). For instance, H3K18 lactylation facilitates the expression and function of YTH domain-containing family protein 2 (YTHDF2), which promotes m6A-dependent mRNA degradation of the tumour suppressor genes period circadian regulator 1 (PER1) and tumor protein p53 (TP53) in ocular melanoma, thereby potentiating tumourigenesis (Yu et al, 2021).These findings collectively reposition lactate as a central regulator of both tumour metabolism and immune evasion in ways previously unappreciated (Dai et al, 2024). However, despite these findings regarding the role of lactate in various cellular functions, the potential connection between lactate and cholesterol metabolism remains largely unexplored.

In this study, we revealed a novel mechanism through which tumour cells adapt to hypoxic conditions via cholesterol metabolic reprogramming. Specifically, we demonstrated that lactate-driven histone H3K18la upregulates SCARB1 expression, leading to increased cholesterol uptake and consequently elevated cholesterol levels under hypoxic conditions. This SCARB1-mediated cholesterol uptake subsequently activates mTORC1 signalling, which in turn inhibits excessive autophagy in hypoxic HCC cells. Collectively, our findings establish the lactate-SCARB1-mTORC1 axis as a critical regulator of hypoxic adaptation and suggest that SCARB1 is a potential therapeutic target for liver cancer.

## Results

### Lactate upregulates SCARB1 to increase cholesterol uptake under hypoxia

**Figure 1:**
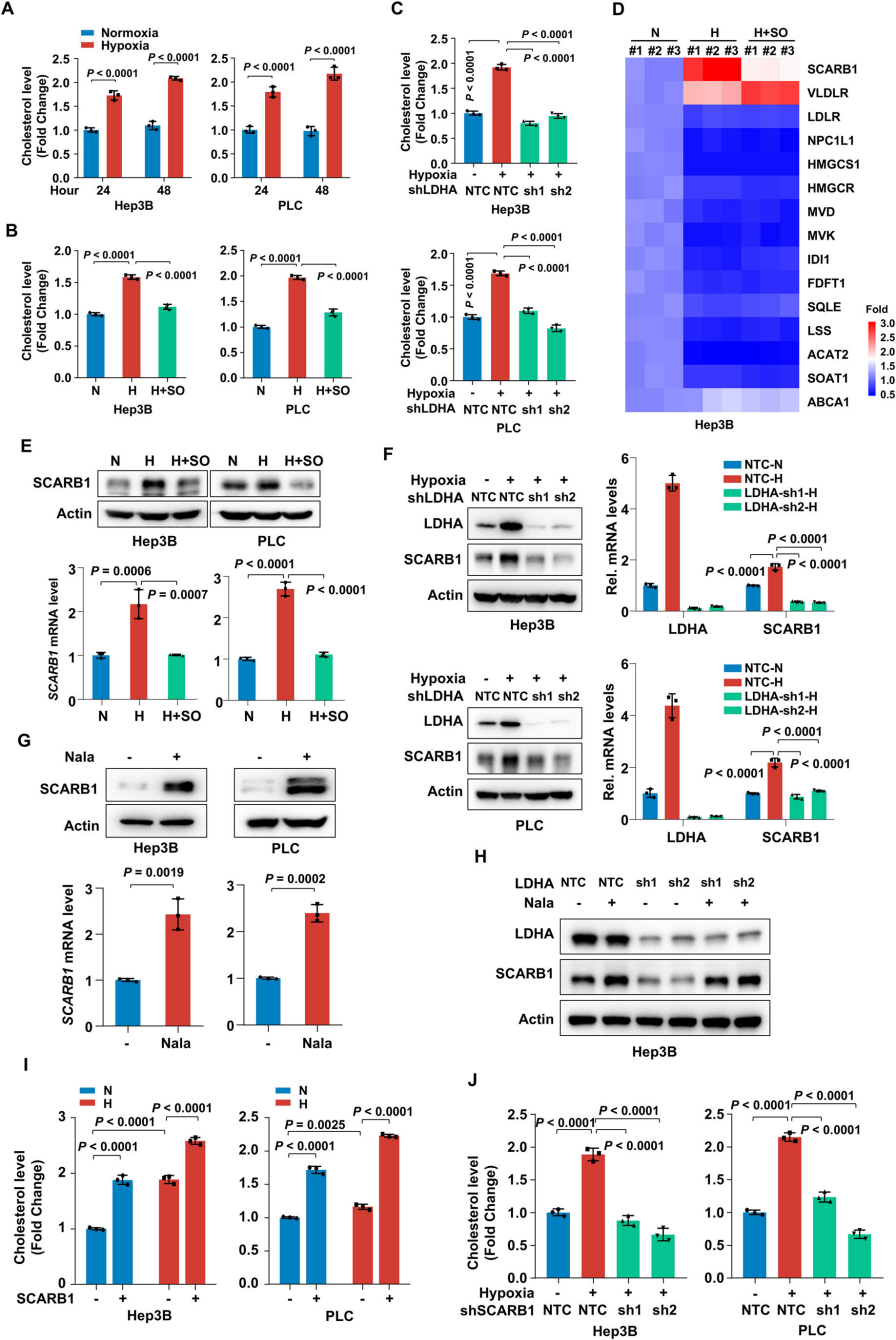
Lactate upregulates SCARB1 to increase cholesterol uptake under hypoxia. (**A**) Intracellular total cholesterol levels in Hep3B and PLC cells cultured under normoxia or hypoxia for 24 or 48 h. 24h-N vs. 24h-H: *P* = 9.2e-6 (Hep3B) and 9.8e-5 (PLC); 48h-N vs. 48-H: *P* = 8.3e-7 (Hep3B) and 4.3e-6 (PLC). (**B**) Hep3B and PLC cells were cultured under normoxia (N), hypoxia (H), or hypoxia with 20 mM sodium oxamate (H + SO) for 48 h. The cellular cholesterol content was measured. H vs. N: *P* = 1.5e-6 (Hep3B) and 5.6e-7 (PLC); H vs. H + SO: *P* = 5.3e-6 (Hep3B) and 4.3e-6 (PLC). (**C**) Hep3B and PLC cells expressing shNTC or shLDHA were cultured under normoxia or hypoxia for 48 h. The cellular cholesterol content was measured. NTC: non-targeting control. NTC-H vs. NTC-N: *P* = 2.9e-8 (Hep3B) and 1.2e-7 (PLC); NTC-H vs. shLDHA#1-H: *P* = 6.1e-9 (Hep3B) and 4.1e-7 (PLC); NTC-H vs. shLDHA#2-H: *P* = 1.8e-8 (Hep3B) and 2.0e-8 (PLC). (**D**) Heatmap of cholesterol metabolism-related gene expression in Hep3B cells cultured under normoxia, hypoxia, or hypoxia with 20 mM sodium oxamate for 48 h. (**E**) Hep3B and PLC cells were cultured under normoxia, hypoxia, or hypoxia with 20 mM sodium oxamate for 48 h. SCARB1 expression was detected by Western blotting and qRT‒PCR. H vs. N: *P* = 2.1e-6; H vs. H + SO: *P* = 3.1e-6. (**F**) Hep3B and PLC cells expressing shNTC or shLDHA were cultured under normoxia or hypoxia for 48 h. SCARB1 expression was detected by Western blotting and qRT‒PCR. NTC-H vs. NTC-N: *P* = 5.0e-6 (Hep3B) and 2.2e-6 (PLC); NTC-H vs. shLDHA#1-H: *P* = 8.3e-10 (Hep3B) and 5.7e-7 (PLC); NTC-H vs. shLDHA#2-H: *P* = 5.1e-10 (Hep3B) and 6.3e-6 (PLC). (**G**) Western blot and qRT‒PCR analyses showing SCARB1 expression in Hep3B and PLC cells treated with 20 mM sodium lactate (Nala) for 48 h. (**H**) Hep3B cells expressing shNTC or shLDHA were treated with or without 20 mM sodium lactate. SCARB1 protein levels were detected by western blotting. (**I**, **J**) Hep3B and PLC cells with SCARB1 overexpression (**I**) or knockdown (**J**) were cultured under normoxia or hypoxia for 48 h, after which the cellular cholesterol content was measured. (**I**) EV-N vs. SCARB1-N: *P* = 8.3e-7 (Hep3B) and 5.0e-8 (PLC); EV-N vs. EV-H: *P* = 7.8e-7 (Hep3B); EV-H vs. SCARB1-H: *P* = 5.2e-6 (Hep3B) and 2.1e-9 (PLC). (J) NTC-H vs. NTC-N: *P* = 3.1e-6 (Hep3B) and 4.4e-8 (PLC); NTC-H vs. shSCARB1#1-H: *P* = 1.1e-6 (Hep3B) and 2.7e-7 (PLC); NTC-H vs. shSCARB1#2-H:*P* = 2.5e-7 (Hep3B) and 5.9e-9 (PLC). Immunoblots are representative of three independent experiments (**E**–**G**). Data are presented as the mean ± s.d. of three independent experiments. Statistical significance was determined by one-way ANOVA (**A**–**C**, **E**, **F**, **I**), unpaired Student’s *t* test (**G**), and two-way ANOVA (**J**). Source data are available online for this figure.

**Figure EV1:**
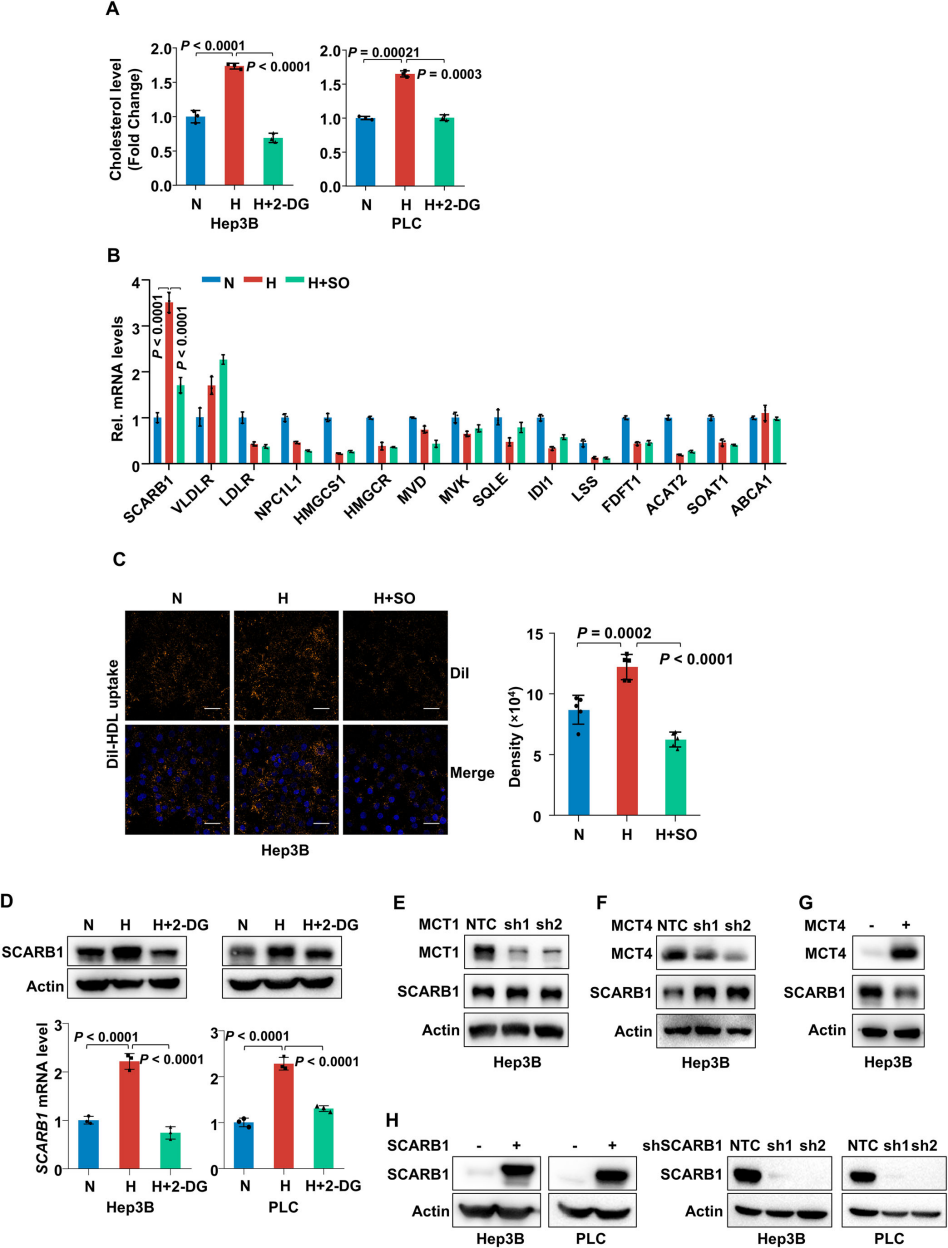
Lactate upregulates SCARB1 to increase cholesterol uptake under hypoxia. (**A**) Hep3B and PLC cells were cultured under normoxia (N), hypoxia (H), or hypoxia with 10 mM 2-DG (H + 2-DG) for 48 h. Cellular cholesterol content was measured. H vs. N: *P* = 2.4e-5; H vs. H + 2-DG: *P* = 3.0e-6. (**B**) mRNA levels of cholesterol metabolism-related genes were determined by RT‒qPCR in Hep3B cells cultured under normoxia, hypoxia, or hypoxia with 20 mM sodium oxamate (H + SO) for 48 h. H vs. N: *P* = 3.8e-6; H vs. H + SO: *P* = 2.6e-5. (**C**) Hep3B cells were cultured under normoxia, hypoxia, or hypoxia with 20 mM sodium oxamate (H + SO) for 48 h, followed by measurement of DiI–HDL uptake. Scale bar: 50 μm. *P* = 1.1e-6. (**D**) Hep3B and PLC cells were cultured under normoxia, hypoxia, or hypoxia with 10 mM 2-DG for 48 h. SCARB1 expression was detected by western blotting and qRT‒PCR. H vs. N: *P* = 4.8e-5 (Hep3B) and 1.1e-5 (PLC); H vs. H + 2-DG: *P* = 1.5e-5 (Hep3B) and 5.2e-5 (PLC). (**E**) Western blot analysis of SCARB1 protein expression in Hep3B cells expressing MCT1 knockdown. (**F**, **G**) Western blot analysis of SCARB1 protein expression in Hep3B cells expressing MCT4 knockdown (**F**) or overexpression (**G**). (**H**) Western blot analysis of SCARB1 expression in Hep3B and PLC cells with SCARB1 overexpression or knockdown. Immunoblots are representative of three independent experiments (**D**, **H**). Data are presented as the mean ± s.d. of three independent experiments. Statistical significance was determined by one-way ANOVA (**A**, **C**, **D**) and two-way ANOVA (**B**). Source data are available online for this figure.

To investigate the relationship between hypoxia and cholesterol metabolism, intracellular cholesterol levels were measured in HCC cells, including Hep3B and PLC cells, cultured under normoxic (20% O_2_) or hypoxic (1% O_2_) conditions. The results showed that intracellular cholesterol levels were markedly higher under hypoxia compared with normoxia, with a further increase observed at 48 h relative to 24 h in both cell lines (Fig. 1A). Given that lactate accumulates under hypoxia and modulates diverse physiological processes (Li et al, 2022), we sought to explore whether lactate contributes to the hypoxia-induced increase in cholesterol levels. After blocking intracellular lactate production with sodium oxamate (SO, LDHA inhibitor), 2-deoxy-D-glucose (2-DG, glycolytic inhibitor), or shRNA targeting LDHA, we found that all the treatments attenuated the hypoxia-induced increase in cholesterol levels, indicating that lactate is responsible for the increased cholesterol levels under hypoxia (Figs. 1B,C and EV1A).

To elucidate the mechanisms underlying the hypoxia-induced increase in cholesterol in cancer cells, we performed RNA sequencing (RNA-seq) analysis of Hep3B cells cultured under normoxia, hypoxia, or treated with sodium oxamate under hypoxia for 48 h, with a specific focus on the cholesterol metabolism pathway. Interestingly, we observed that hypoxia resulted in the global downregulation of genes involved in de novo cholesterol biosynthesis, such as 3-hydroxy-3-methylglutaryl-CoA reductase (HMGCR), mevalonate kinase (MVK), and squalene epoxidase (SQLE), while simultaneously upregulating genes involved in cholesterol uptake, including SCARB1, very low-density lipoprotein receptor (VLDLR) (Fig. 1D). This expression pattern reflects a metabolic shift that enables cells to adapt to hypoxic conditions. Moreover, although the expression of both SCARB1 and VLDLR was increased under hypoxia, only SCARB1 exhibited consistent lactate-dependent regulation (Figs. 1D and  EV1B).

Considering that SCARB1 is known for its role in mediating selective high-density lipoprotein (HDL) uptake, we first confirmed that HDL uptake was increased under hypoxic conditions, and this effect was inhibited by sodium oxamate (Fig. EV1C), indicating that lactate-mediated upregulation of SCARB1 may be a key mechanism contributing to the hypoxia-induced increase in intracellular cholesterol. To validate this hypothesis, we first examined SCARB1 expression at both the mRNA and protein levels. The results revealed that SCARB1 expression was upregulated under hypoxia and downregulated by treatment with sodium oxamate or 2-DG, as well as by LDHA knockdown (Figs. 1E,F and EV1D). Conversely, supplementation with sodium lactate, the basic form of lactate (Zong et al, 2024), promoted SCARB1 expression (Fig. 1G) and fully rescued the suppression caused by LDHA knockdown (Fig. 1H).

Furthermore, we investigated the roles of monocarboxylate transporter 1 (MCT1) and monocarboxylate transporter 4 (MCT4) to elucidate the role of lactate in regulating SCARB1 expression. The results showed that MCT1 knockdown did not significantly affect SCARB1 expression (Fig. EV1E). In contrast, knockdown of MCT4, which limits lactate efflux and thereby promotes intracellular lactate accumulation, resulted in a marked upregulation of SCARB1 expression (Fig. EV1F). Consistently, overexpression of MCT4 significantly suppressed SCARB1 expression (Fig. EV1G). This is likely due to the fact that HCC cells, being highly glycolytic, produce the majority of their intracellular lactate endogenously, making the contribution of extracellular lactate minimal.

Finally, to investigate the role of SCARB1 in mediating hypoxia-induced cholesterol accumulation in HCC cells, we generated stable Hep3B and PLC cell lines with either SCARB1 overexpression or SCARB1 knockdown (Fig. EV1H). Under hypoxic conditions, SCARB1 further increased cholesterol uptake (Fig. 1I), whereas SCARB1 knockdown markedly attenuated the hypoxia-induced increase in cholesterol levels (Fig. 1J). These results demonstrate that lactate enhances cholesterol uptake by promoting SCARB1 expression under hypoxia.

### Histone lactylation activates SCARB1 transcription

**Figure 2:**
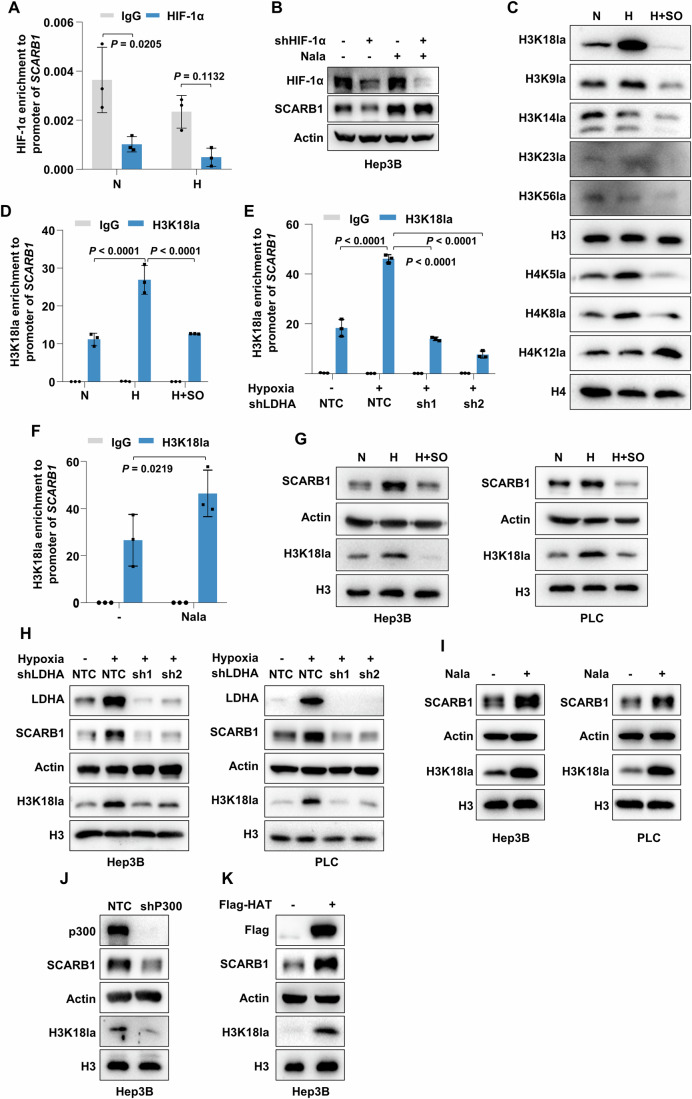
Histone lactylation activates SCARB1 transcription. (**A**) ChIP‒qPCR analysis of the occupancy of HIF-1α at the SCARB1 promoter in Hep3B cells cultured under normoxia (N) or hypoxia (H) for 6 h. (**B**) Hep3B cells expressing shNTC or shHIF1-α were treated with or without 20 mM sodium lactate. SCARB1 protein levels were detected by Western blotting. (**C**) Western blot analysis of H3K9la, H3K18la, H3K14la, H3K23la, H3K56la, H4K5la, H4K8la, and H4K12la levels in Hep3B cells cultured under normoxia, hypoxia, or hypoxia with 20 mM sodium oxamate for 48 h. (**D**) ChIP‒qPCR analysis of H3K18la occupancy at the SCARB1 promoter in Hep3B cells cultured under normoxia, hypoxia, or hypoxia with 20 mM sodium oxamate for 48 h. H vs. N: *P* = 1.7e-7; H vs. H + SO: *P* = 4.9e-7. (**E**) ChIP‒qPCR analysis of H3K18la occupancy at the SCARB1 promoter in Hep3B cells expressing shNTC or shLDHA and cultured under normoxia or hypoxia for 48 h. NTC-H vs. NTC-N: *P* = 2.3e-13; NTC-H vs. shLDHA#1-H: *P* = 2.4e-14; NTC-H vs. shLDHA#2-H: *P* = 1.3e-15. (**F**) ChIP‒qPCR analysis of the occupancy of H3K18la at the SCARB1 promoter in Hep3B cells cultured under normoxia with or without 20 mM sodium lactate. (**G**) Western blot analysis of H3K18la and SCARB1 expression in Hep3B and PLC cells cultured under normoxia, hypoxia, or hypoxia with 20 mM sodium oxamate for 48 h. (**H**) Western blot analysis of H3K18la and SCARB1 expression in Hep3B and PLC cells expressing shNTC or shLDHA and cultured under normoxia or hypoxia for 48 h. (**I**) Western blot analysis of SCARB1 expression in Hep3B and PLC cells cultured under normoxia with or without 20 mM sodium lactate for 48 h. (**J**) Western blot analysis of SCARB1 protein expression in Hep3B cells expressing shNTC or shP300. (**K**) Western blot analysis of SCARB1 protein expression in Hep3B cells with p300-HAT domain overexpression. Immunoblots are representative of three independent experiments (**G**–**I**). Data are presented as the mean ± s.d. of three independent experiments. Statistical significance was determined by two-way ANOVA (**A**, **D**–**F**). Source data are available online for this figure.

Next, we investigated the mechanism underlying the hypoxia-induced upregulation of SCARB1. As hypoxia-inducible factor 1-alpha (HIF-1α) is a key transcription factor and serves key roles in regulating glucose and lipid metabolism in tumour cells (Missiaen et al, 2023; Semenza, 2003, 2013), we first performed chromatin immunoprecipitation (ChIP) assays to determine whether HIF-1α directly regulates SCARB1 as a transcription factor. The results revealed that HIF-1α failed to bind to the SCARB1 promoter under either normoxia or hypoxia (Fig. 2A), indicating that HIF-1α does not directly regulate SCARB1 transcription. Given the established role of HIF-1α in driving glycolytic reprogramming and lactate production, we next explored a potential indirect regulatory mechanism. Notably, while HIF-1α knockdown reduced SCARB1 expression, supplementation with sodium lactate restored SCARB1 levels (Fig. 2B), indicating that hypoxia-induced SCARB1 expression is mediated through a HIF-1α–lactate axis rather than by direct transcriptional activation by HIF-1α itself.

**Figure EV2:**
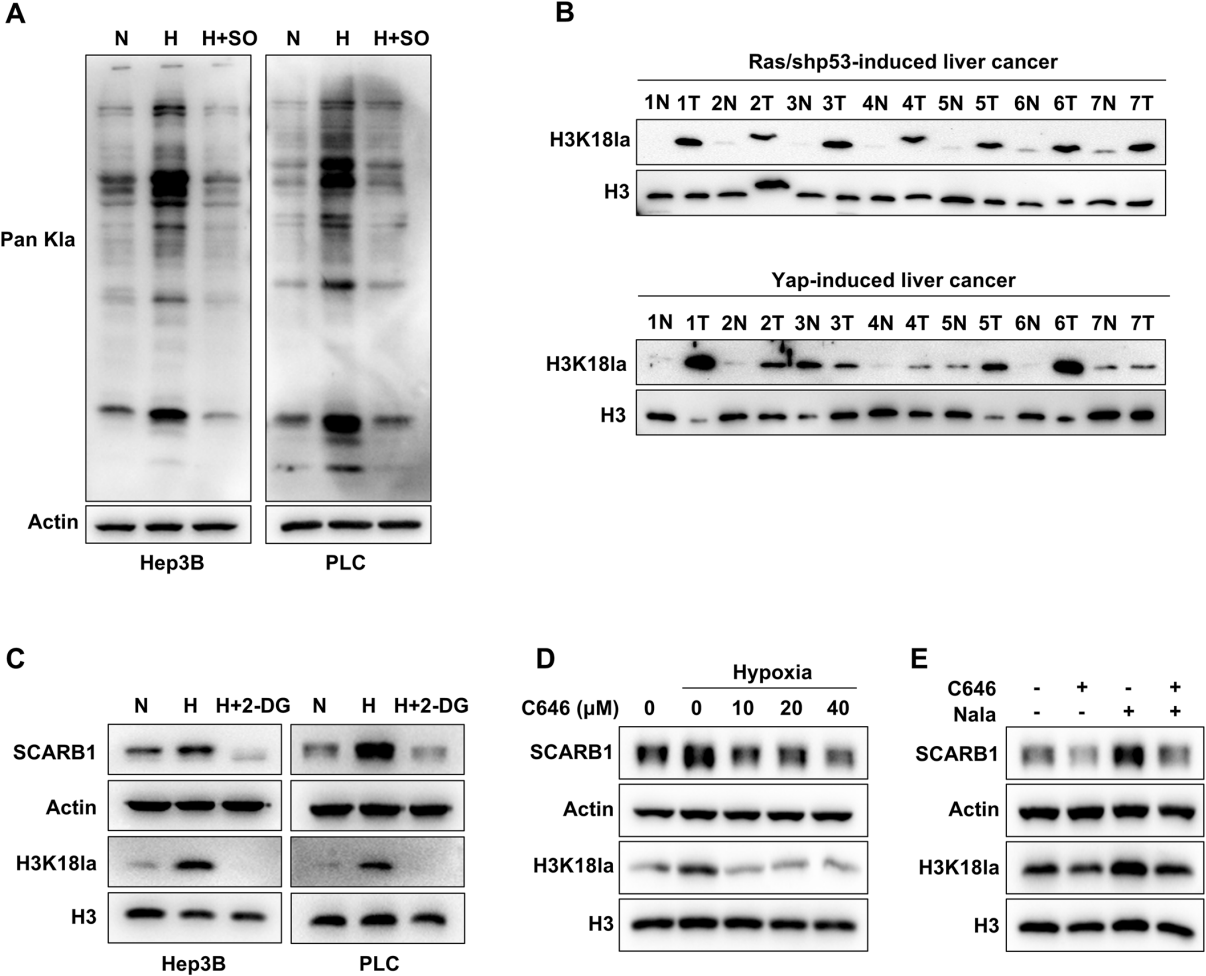
Histone lactylation activates SCARB1 transcription. (**A**) Western blot analysis of panlactylation in Hep3B and PLC cells cultured under normoxia, hypoxia, or hypoxia with 20 mM sodium oxamate for 48 h. (**B**) Western blot analysis of H3K18la expression in paired adjacent noncancerous liver tissues (N) and cancerous liver tissues (T) in YAP-5SA- or Ras/shp53-induced mouse HCC. (**C**) Western blot analysis of H3K18la and SCARB1 expression in Hep3B and PLC cells cultured under normoxia, hypoxia, or hypoxia with 10 mM 2-DG for 48 h. Immunoblots are representative of three independent experiments (**A**, **C**). (**D**) Western blot analysis of H3K18la and SCARB1 expression in Hep3B cells treated with the p300 inhibitor C646 and cultured under normoxia or hypoxia for 48 h. (**E**) Western blot analysis of H3K18la and SCARB1 expression in Hep3B cells treated with 40 μM C646, 20 mM sodium lactate, or a combination of sodium lactate and C646 and cultured under hypoxia for 48 h. Immunoblots are representative of three independent experiments (**A**, **C**–**E**). Source data are available online for this figure.

Because HIF-1α does not mediate the transcriptional regulation of SCARB1, we focused on an epigenetic mechanism—histone lactylation—as a potential explanation for the upregulation of SCARB1 at both the mRNA and protein levels. First, we observed increased lysine lactylation (Pan-Kla) under hypoxic conditions, which was attenuated by sodium oxamate treatment (Fig. EV2A), highlighting the importance of lactylation in HCC cells. Next, we examined the currently known sites of histone lactylation modification and found that H3K18la exhibited the most significant changes in tumour cells (Fig. 2C). Furthermore, this modification was markedly elevated in HCC tissues from YAP-5SA- or Ras/shp53-induced mouse models compared to matched adjacent noncancerous liver tissues (Fig. EV2B). Given that histone modifications can directly modulate gene transcription and considering the established role of H3K18la as a cancer-associated epigenetic marker (Ma et al, 2025; Qiao et al, 2024), we hypothesized that H3K18la mediates the lactate-dependent regulation of SCARB1 expression.

To confirm the regulatory role of H3K18la in SCARB1 transcription, we performed a ChIP‒qPCR assay with an anti-H3K18la antibody. The results revealed significant enrichment of H3K18la at the SCARB1 promoter, which was dependent on lactate, as it was decreased by sodium oxamate treatment (Fig. 2D) or LDHA knockdown (Fig. 2E) but increased by sodium lactate supplementation (Fig. 2F). Consistently, Western blot analysis revealed that the protein levels of H3K18la and SCARB1 decreased with sodium oxamate (Fig. 2G), 2-DG treatment (Fig. EV2C), or LDHA knockdown (Fig. 2H) but increased with sodium lactate supplementation (Fig. 2I).

To further determine the role of histone lactylation in the regulation of SCARB1 expression, we manipulated p300, a histone acetyltransferase that has also been identified as a key enzyme for histone lactylation (Yu et al, 2021; Zhang et al, 2019). The results showed that knockdown of p300 downregulated SCARB1 expression, while overexpression of the catalytic histone acetyltransferase (HAT) domain of p300 upregulated SCARB1 (Fig. 2J-K). Additionally, the hypoxia-induced upregulation of SCARB1 expression was attenuated by C646 treatment, an inhibitor of p300 (Fig. EV2D). C646 suppressed SCARB1 expression, even in the presence of sodium lactate, indicating that p300 is required for lactate-mediated SCARB1 upregulation (Fig. EV2E). Thus, these findings establish a mechanistic link between lactate and SCARB1 upregulation through histone lactylation and further demonstrate that targeting lactate production or p300 activity can effectively modulate SCARB1 expression.

### SCARB1 promotes cell growth by inhibiting autophagy

**Figure 3:**
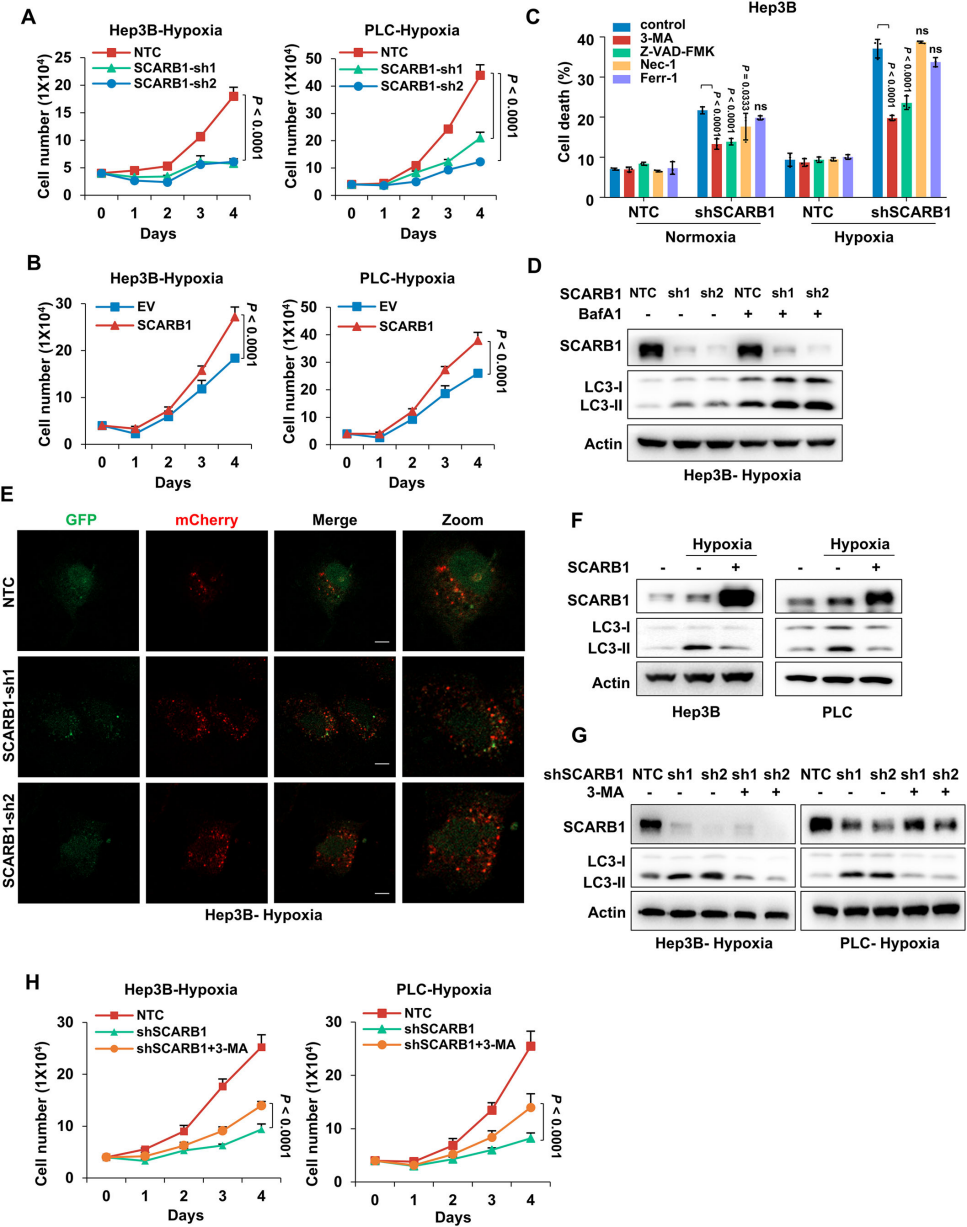
SCARB1 promotes cell growth by inhibiting autophagy. (**A**, **B**) Growth curves were generated for Hep3B and PLC cells expressing shNTC or shSCARB1 (**A**) and for cells expressing SCARB1 (**B**) under hypoxia. (**A**) NTC vs. shSCARB1#1: *P* = 3.7e-10 (Hep3B) and 1.0e-11 (PLC); NTC vs. shSCARB1#2: *P* = 4.2e-11 (Hep3B) and 3.6e-14 (PLC). (**B**) *P* = 9.6e-7 (Hep3B) and 2.1e-8 (PLC). (**C**) Analysis of the death of Hep3B cells expressing shNTC or shSCARB1 and treated with 2 mM 3-MA, 5 μM Z-VAD-FMK, 2 µM Nec-1, or 2 µM Ferr-1 under normoxia or hypoxia for 48 h. Normoxia: ctrl vs. 3-MA (*P* = 1.5e-8), ctrl vs. Z-VAD-FMK (*P* = 5.5e-8); Hypoxia: ctrl vs. 3-MA (*P* = 4.0e-15), ctrl vs. Z-VAD-FMK (*P* = 1.0e-12). (**D**) Cells expressing shNTC or shSCARB1 were treated with BafA1 (100 nM, 12 h) under hypoxia. LC3-II levels were detected by Western blotting. (**E**) Ad-GFP-mCherry-LC3 was expressed in Hep3B cells with SCARB1 knockdown under hypoxia. The number of LC3 puncta was analysed using a fluorescence microscope. Scale bars: 10 μm. (**F**) Western blot analysis of SCARB1 and LC3-I/II levels in Hep3B and PLC cells expressing SCARB1 under normoxia or hypoxia for 48 h. (**G**) Hep3B and PLC cells expressing shNTC, shSCARB1, or shSCARB1 with 2 mM 3-MA were cultured under hypoxia for 48 h. SCARB1 and LC3-I/II levels were detected by western blotting. (**H**) Growth curves were generated for Hep3B and PLC cells expressing shNTC, shSCARB1, or shSCARB1 with 2 mM 3-MA. *P* = 1.6e-5 (Hep3B) and 2.6e-6 (PLC). Immunoblots are representative of three independent experiments (**D**, **F**, **G**). Data are presented as the mean ± s.d. of three independent experiments. Statistical significance was determined by two-way ANOVA (**A**–**C**, **H**). Source data are available online for this figure.

**Figure EV3:**
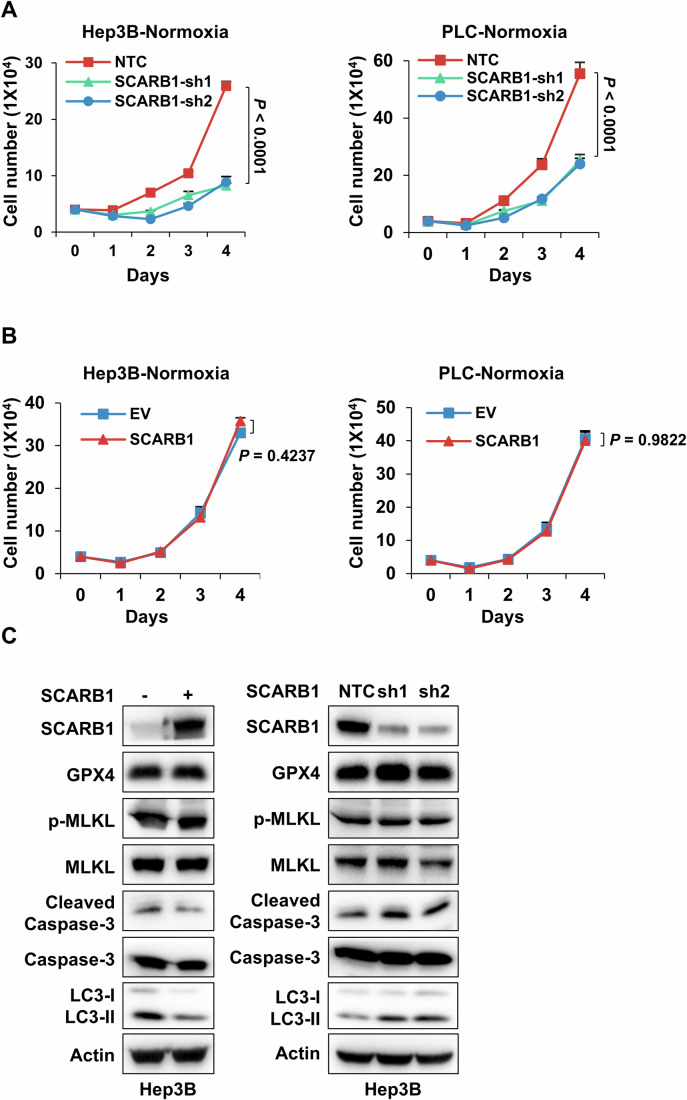
SCARB1 promotes cell growth by inhibiting autophagy. (**A**, **B**) Growth curves were generated for Hep3B and PLC cells expressing shNTC or shSCARB1 (**A**) and for cells expressing SCARB1 (**B**) under normoxia. (**A**) NTC vs. shSCARB1#1: *P* = 3.5e-11 (Hep3B) and 4.6e-11 (PLC); NTC vs. shSCARB1#2: *P* = 1.3e-15 (Hep3B) and 1.6e-10 (PLC). (**C**) Western blot analysis of GPX4, phosphor-MLKL, MLKL, cleaved caspase-3, Caspase-3, and LC3-I/II in Hep3B cells expressing SCARB1 overexpression or knockdown under hypoxia. Data are presented as the mean ± s.d. of three independent experiments. Statistical significance was determined by two-way ANOVA (**A**, **B**).Source data are available online for this figure.

Having established that lactate upregulates SCARB1 to promote cholesterol accumulation under hypoxic conditions, we next examined the functional role of SCARB1 in the proliferation of HCC cells. Our results revealed that compared with nontargeting control shRNA, SCARB1 knockdown significantly inhibited cell growth under both normoxia and hypoxia (Figs. 3A and EV3A), suggesting that SCARB1 is required for cell proliferation and cell survival under both conditions. However, SCARB1 overexpression promoted HCC cell growth under hypoxia (Fig. 3B) but did not have a comparable proproliferative effect under normoxia (Fig. EV3B). These results suggest that tumour cells become particularly dependent on SCARB1-mediated cholesterol uptake under hypoxic conditions to support their proliferative capacity.

Given the significant effect of SCARB1 on HCC cell proliferation, we sought to elucidate its underlying mechanism. To determine whether the growth inhibition induced by SCARB1 knockdown was mediated by specific cell death pathways, we first examined the impact of SCARB1 depletion on various forms of cell death. Our immunoblot analysis revealed that SCARB1 knockdown failed to induce necroptosis and ferroptosis, but induced autophagy and apoptosis (Fig. EV3C). Next, we treated SCARB1-deficient cells with inhibitors targeting four major forms of cell death: 3-methyladenine (3-MA, autophagy inhibitor), Z-VAD-FMK (apoptosis inhibitor), necrostatin-1 (Nec-1, necroptosis inhibitor), and ferrostatin-1 (Ferr-1, ferroptosis inhibitor), to investigate which inhibitor could rescue cell death. Strikingly, we found that the autophagy inhibitor 3-MA substantially rescued cell death induced by SCARB1 knockdown under hypoxia (Fig. 3C), indicating that SCARB1 depletion predominantly triggered autophagic cell death in HCC cells.

While autophagy typically functions as a cytoprotective mechanism during environmental stress (e.g., nutrient deprivation or hypoxia) (Lin et al, 2012; Lum et al, 2005), sustained or excessive autophagic activity can ultimately lead to cell death (Kondo et al, 2005; Shimizu et al, 2004). To determine whether SCARB1 modulates autophagy under hypoxic conditions, we monitored the protein accumulation of microtubule-associated protein 1A/1B-light chain 3-II (LC3-II), a well-established autophagy marker. Western blot analysis showed that SCARB1 knockdown significantly increased LC3-II accumulation both with and without Bafilomycin A1 (BafA1) treatment, indicating enhanced autophagosome formation (Fig. 3D). In addition, we tested the effects of SCARB1 on autophagic flux using the mCherry-GFP-LC3B adenovirus in SCARB1 knockdown cells. The data showed that SCARB1 knockdown enhanced autophagosome maturation (Fig. 3E). Conversely, SCARB1 overexpression suppressed hypoxia-induced autophagy (Fig. 3F). Importantly, the autophagy inhibitor 3-MA partially rescued both the autophagic flux and proliferation defects caused by SCARB1 knockdown (Fig. 3G,H). Collectively, these data demonstrate that SCARB1 promotes HCC cell survival under hypoxia by restraining excessive autophagy.

### SCARB1 activates mTORC1 signalling through cholesterol uptake

**Figure EV4:**
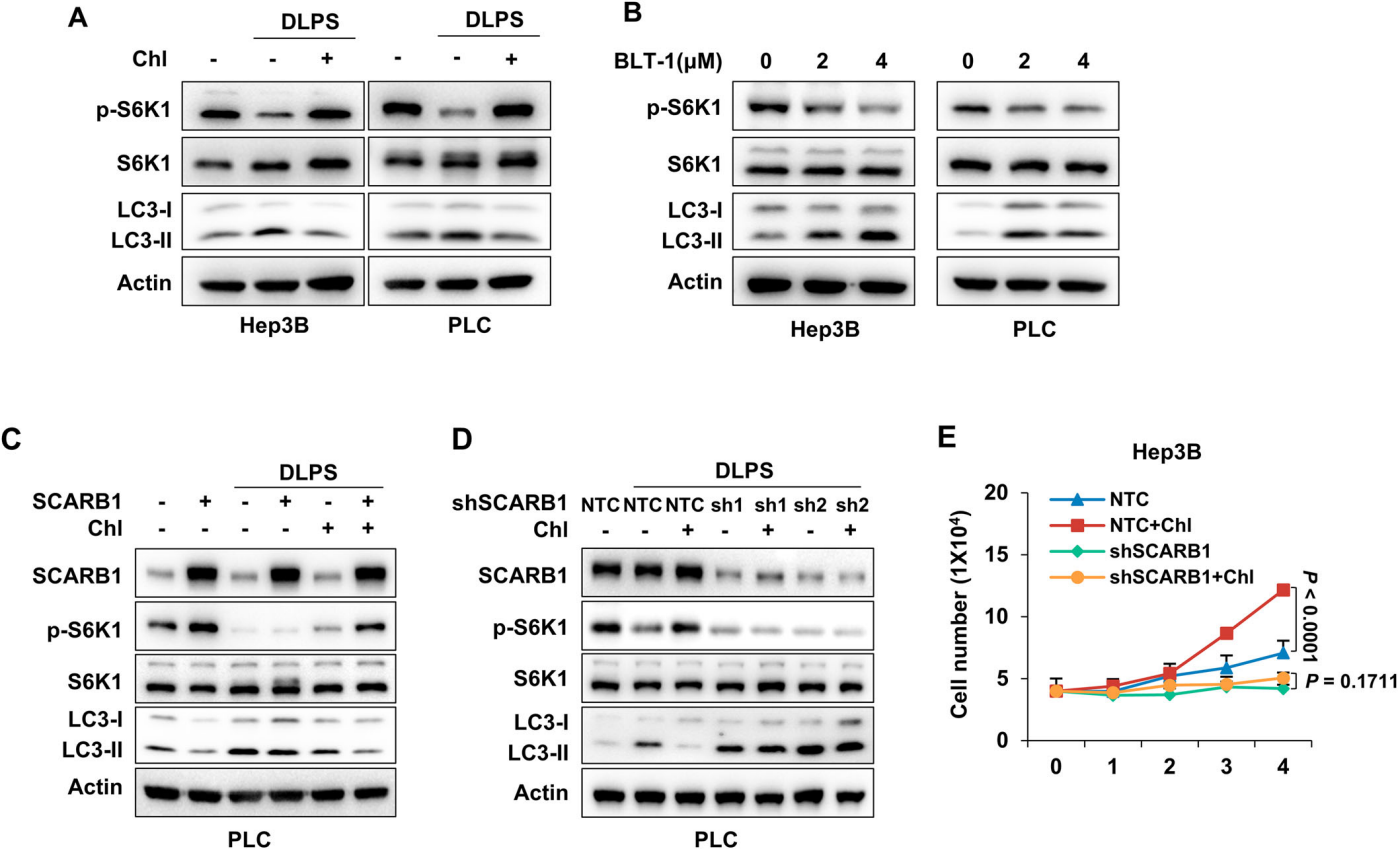
SCARB1 activates mTORC1 signalling through cholesterol uptake. (**A**) Western blot analysis of SCARB1, p-S6K1, S6K1, and LC3-I/II in Hep3B cells cultured in 10% FBS or 10% DLPS in the absence or presence of 10 μg/mL cholesterol for 2 h under hypoxia. (**B**) Western blot analysis of SCARB1, p-S6K1, S6K1, and LC3-I/II in Hep3B and PLC cells treated with BLT-1 under hypoxia for 48 h. (**C**, **D**) PLC cells expressing SCARB1 (**C**) or shSCARB1 (**D**) were cultured in 10% FBS or 10% DLPS in the absence or presence of 10 μg/mL cholesterol under hypoxia. SCARB1, p-S6K1, S6K1, and LC3-I/II protein levels were detected by Western blotting. (**E**) Growth curves were generated for Hep3B cells expressing shNTC or shSCARB1 and treated with or without 10 μg/mL cholesterol under hypoxia. *P* = 4.3e-10. Immunoblots are representative of three independent experiments (**A**–**D**). Data are presented as the mean ± s.d. of three independent experiments. Statistical significance was determined by two-way ANOVA (**E**). Source data are available online for this figure.

**Figure 4:**
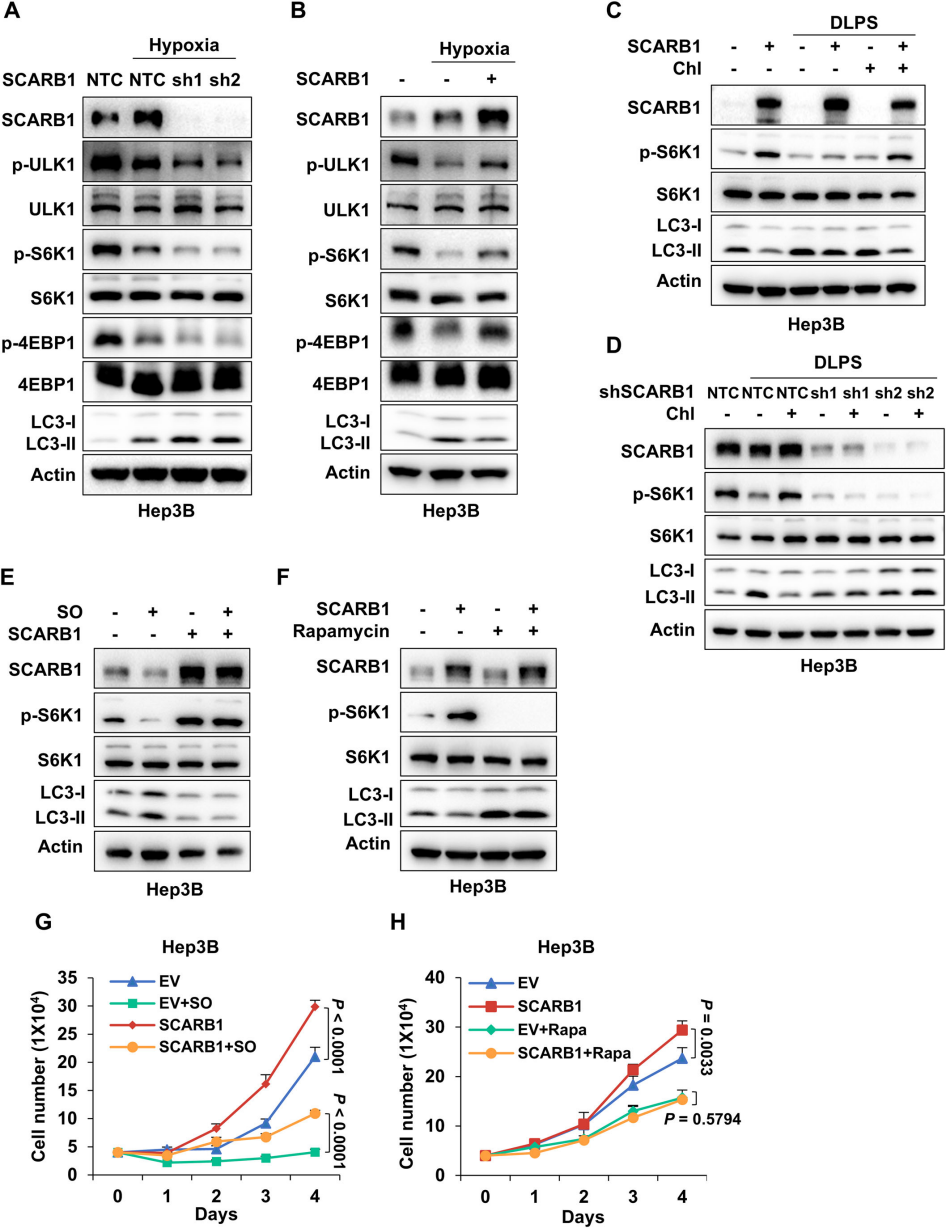
SCARB1 activates mTORC1 signalling through cholesterol uptake. (**A**, **B**) Western blot analysis of SCARB1, p-ULK1, ULK1, p-S6K1, S6K1, p-4EBP1, 4EBP1, and LC3-I/II in Hep3B cells expressing shNTC or shSCARB1 (**A**) or expressing SCARB1 (**B**) under hypoxia for 48 h. (**C**, **D**) Hep3B and PLC cells expressing SCARB1 (**C**) or shSCARB1 (**D**) were cultured in 10% FBS or 10% delipidated serum (DLPS) in the absence or presence of 10 μg/mL cholesterol (chl) under hypoxia. SCARB1, p-S6K1, S6K1, and LC3-I/II protein levels were detected by Western blotting. (**E**) Hep3B cells expressing SCARB1 were treated with or without 20 mM sodium oxamate under hypoxia. SCARB1, p-S6K1, S6K1, and LC3-I/II protein levels were detected by western blotting. (**F**) Hep3B cells expressing SCARB1 were treated with or without 100 nM rapamycin for 6 h under hypoxia. SCARB1, p-S6K1, S6K1, and LC3-I/II protein levels were detected by western blotting. (**G**, **H**) Growth curves of Hep3B cells expressing SCARB1 with or without 20 mM sodium oxamate (**G**) or 100 nM rapamycin (**H**). (**G**) EV vs. SCARB1: *P* = 2.7e-15; EV + SO vs. SCARB1 + SO: *P* = 2.7e-10. Immunoblots are representative of three independent experiments (**C**–**F**). Data are presented as the mean ± s.d. of three independent experiments. Statistical significance was determined by two-way ANOVA (**G**, **H**). Source data are available online for this figure.

We next explored the molecular mechanism through which SCARB1 modulates autophagy. mTORC1, which is activated in response to growth-promoting signals, including cholesterol and amino acids, regulates autophagy via its key downstream effector, p70S6 kinase 1 (p-S6K1) (Castellano et al, 2017; Lim et al, 2019; Russell et al, 2014; Sancak et al, 2008). As expected, supplementation with exogenous cholesterol reactivated mTORC1 signalling and inhibited autophagy in Hep3B and PLC cells cultured in medium supplemented with 10% delipidated serum (DLPS) (Fig. EV4A). Therefore, we hypothesized that tumour cells upregulate SCARB1 expression as a compensatory mechanism to counteract hypoxia-induced mTORC1 inhibition and subsequent autophagy, thereby promoting survival under hypoxic conditions. In support of this model, SCARB1 knockdown further reduced the phosphorylation of UNC-51-like kinase 1 (ULK1), S6K1, and eukaryotic translation initiation factor 4E-binding protein 1 (4EBP1) under hypoxia, all of which are established readouts of mTORC1 activity (Fig. 4A). Conversely, SCARB1 overexpression increased the phosphorylation of these targets under hypoxia (Fig. 4B). In addition, treatment with Block Lipid Transporter-1 (BLT-1) (Nieland et al, 2002), a potent inhibitor of SCARB1-mediated lipid transport, recapitulated the effects of SCARB1 knockdown (Fig. EV4B). Given that our data highlight the importance of SCARB1-mediated cholesterol uptake for HCC cell survival under hypoxic conditions, we further investigated the effects of cholesterol availability on mTORC1 activation and autophagy inhibition in the presence or absence of SCARB1. Strikingly, SCARB1 overexpression failed to increase S6K1 phosphorylation in the absence of exogenous cholesterol supplementation (Figs. 4C and EV4C). In contrast, SCARB1 knockdown eliminated the ability of exogenous cholesterol to induce S6K1 phosphorylation and to promote cell growth (Figs. 4D and EV4D,E). Together, these results further confirm that the SCARB1-mediated activation of mTORC1 is dependent on cholesterol uptake.

To elucidate how SCARB1 integrates lactate signalling with mTORC1 activation, we conducted a series of mechanistic rescue experiments. Strikingly, SCARB1 overexpression completely restored mTORC1 activity and suppressed autophagy in lactate-depleted cells (Fig. 4E), placing SCARB1 downstream of lactate but upstream of mTORC1. This hierarchical relationship was further validated when rapamycin (Rapa, an mTORC1 inhibitor) treatment abrogated the suppression of autophagy mediated by SCARB1 overexpression (Fig. 4F). Functionally, while lactate deprivation suppressed cell growth, SCARB1 overexpression partially reversed this growth inhibition (Fig. 4G). However, SCARB1 overexpression failed to counteract the cell proliferation arrest induced by the mTORC1 inhibitor rapamycin (Fig. 4H), demonstrating that the proproliferative effect of SCARB1 operates through the mTORC1 signalling pathway. Together, these in vitro findings indicate that lactate-induced SCARB1 upregulation facilitates cholesterol uptake to activate mTORC1 signalling, thereby preventing excessive autophagy-mediated cell death in HCC cells under hypoxia.

### SCARB1 promotes tumour progression in vivo

**Figure 5:**
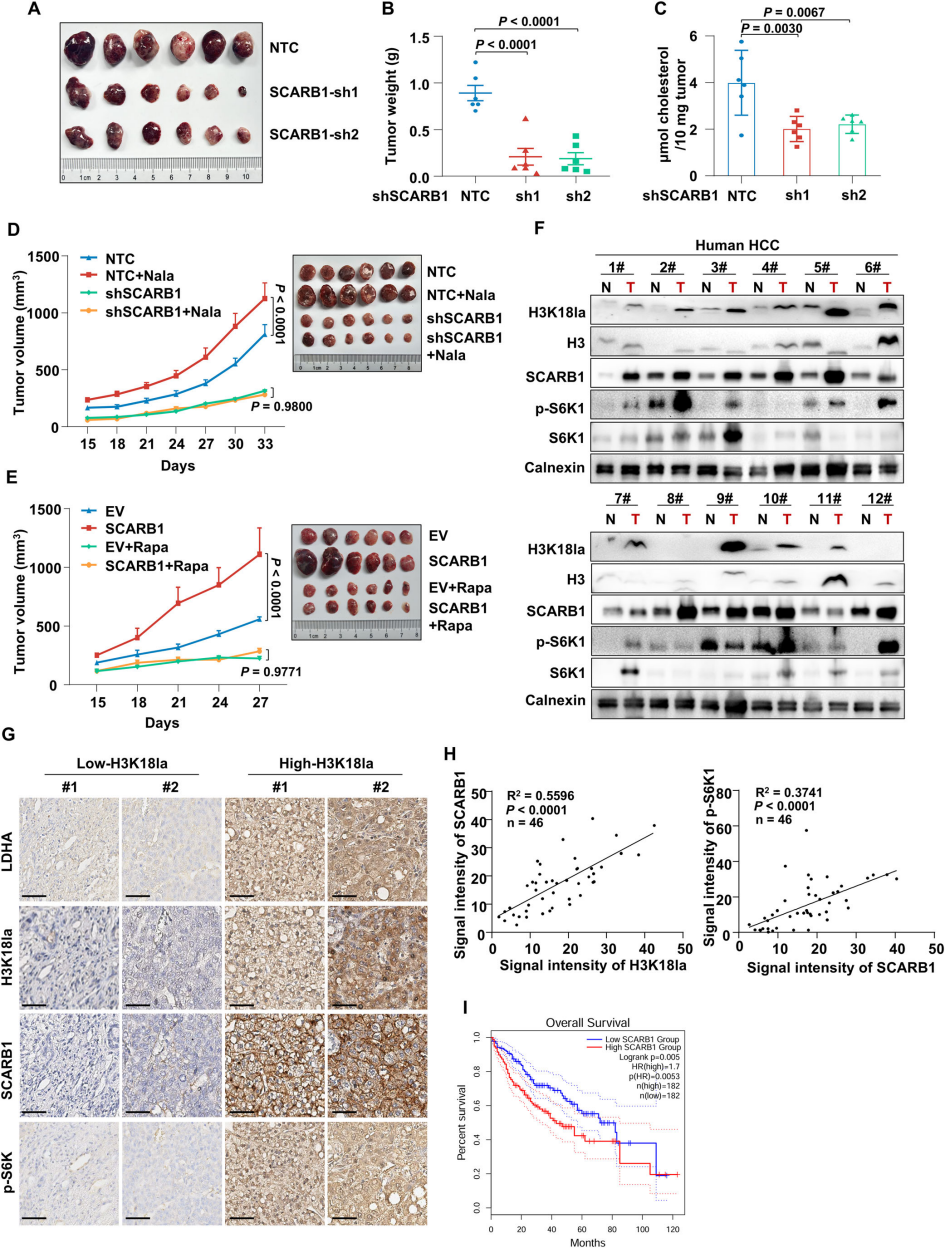
SCARB1 promotes tumour progression in vivo. (**A**, **B**) Hep3B cells expressing shNTC or shSCARB1 were injected subcutaneously into nude mice (*n* = 6 male mice per group). Tumours were extracted and compared at the end of the experiment. (**B**) NTC vs. shSCARB1#1: *P* = 4.2e-5; NTC vs. shSCARB1#2: *P* = 3.0e-5. (**C**) Cholesterol contents were measured in six independent tumours from each group as described in (**A**). (**D**) Hep3B cells expressing shNTC or shSCARB1 were injected subcutaneously into nude mice (*n* = 6 male mice per group). Tumour growth was measured starting 15 days after inoculation, and tumours were extracted and compared at the end of the experiment. *P* = 3.6e-12. (**E**) Hep3B cells expressing EV or SCARB1 were injected subcutaneously into nude mice (*n* = 6 male mice per group). Tumour growth was measured starting 15 days after inoculation, and tumours were extracted and compared at the end of the experiment. *P* = 3.3e-9. (**F**) Western blot analysis of H3K18la, SCARB1, and p-S6K1 expression in paired tumour-adjacent noncancerous liver tissues and human HCC tissues. (**G**) Representative immunohistochemistry images of LDHA, H3K18la, SCARB1, and p-S6K1 staining in HCC specimens; Scale bars, 30 μm. (**H**) Analysis of the correlation between the protein levels of H3K18la and SCARB1, as well as SCARB1 and p-S6K1 in HCC specimens. Pearson correlation analyses were performed. *P* = 2.3e-9 (left), *P* = 6.3e-6 (right). (**I**) Kaplan‒Meier curves with univariate analyses for patients with low versus high SCARB1 expression in the GEPIA database. Data are presented as the mean ± s.e.m. (**B**–**E**). Statistical significance was determined by two-way ANOVA (**D**, **E**), one-way ANOVA (**B**, **C**), and Pearson correlation analyses (**H**). Source data are available online for this figure.

**Figure EV5:**
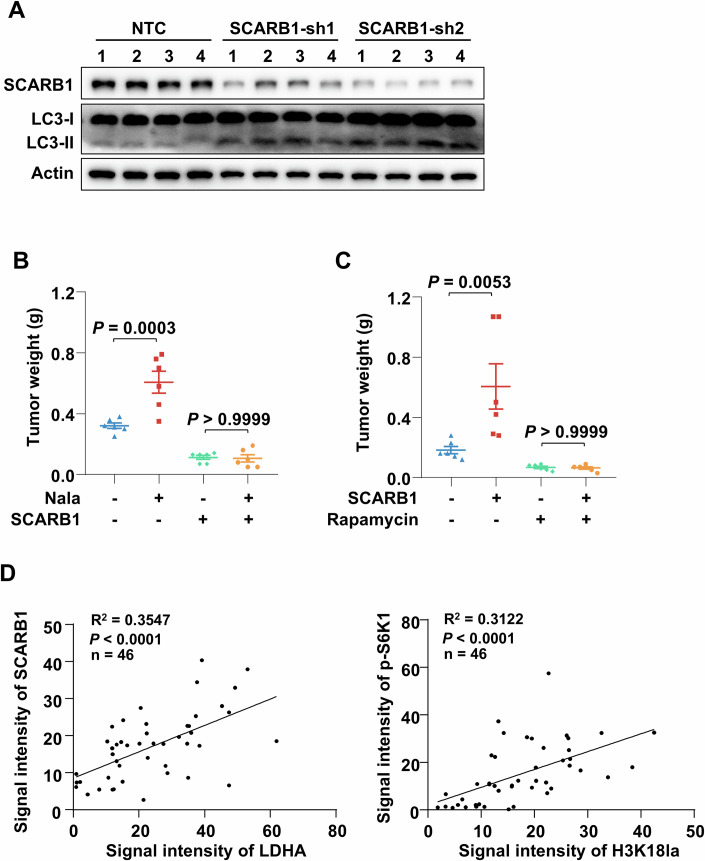
SCARB1 promotes tumour progression in vivo. (**A**) Protein levels of SCARB1 and LC3-I/II were detected by Western blot using lysates from four independent tumours of each group, as shown in Fig. 5A. (**B**) Hep3B cells expressing shNTC or shSCARB1 were injected subcutaneously into nude mice, and tumour mass was measured at the end of the experiment. (**C**) Hep3B cells expressing EV or SCARB1 were injected subcutaneously into nude mice, and tumour mass was measured at the end of the experiment. (**D**) Analysis of the correlation between the protein levels of LDHA and SCARB1, as well as H3K18la and p-S6K1 in HCC specimens. Pearson correlation analyses were performed. *P* = 1.3e-5 (left), *P* = 5.4e-5 (right). Data are presented as the mean ± s.e.m (**B**, **C**). Statistical significance was determined by one-way ANOVA (**B**, **C**). Source data are available online for this figure.

Solid tumours are characterized by complex microenvironments, including hypoxia, low pH, and nutrient stress, among other features. To evaluate the effect of SCARB1 on cancer cell proliferation in vivo, we performed xenograft experiments in nude mice. Stable knockdown of SCARB1 using shRNAs markedly reduced both the volume and weight (Fig. 5A,B) of the xenografts as well as the cholesterol level (Fig. 5C). Increased LC3-II levels were observed in tumour tissues generated from shSCARB1 Hep3B cells (Fig. EV5A), which is consistent with our in vitro findings.

Given the well-established role of lactate in promoting HCC progression, we sought to determine whether this effect requires SCARB1. Consistent with previous reports (Chen et al, 2025), compared with vehicle treatment, sodium lactate treatment significantly enhanced tumour growth (Figs. 5D and EV5B). However, this protumourigenic effect was completely abrogated in SCARB1-deficient xenografts, whose growth rate, size, and weight did not differ between the lactate-treated and control groups (Figs. 5D and EV5B). To further elucidate the underlying mechanism, we performed additional xenograft experiments and demonstrated that rapamycin treatment completely blocked SCARB1-driven tumour growth (Figs. 5E and EV5C), establishing mTORC1 signalling as the crucial downstream pathway. Taken together, these in vivo results indicate that SCARB1, which is positively regulated by lactate, promotes tumour growth through activation of mTORC1 signalling.

Next, to better illustrate the potential pathological and clinical relevance of the lactate-SCARB1-mTORC1 axis, SCARB1 expression was detected in 12 paired HCC lesions and adjacent noncancerous tissue samples. Western blot assays revealed increased H3K18la, SCARB1, and p-S6K1 protein levels in HCC tissue compared with those in adjacent noncancerous tissue (Fig. 5F). Immunohistochemistry (IHC) was also performed in a cohort of 46 pairs of clinicopathologically characterized liver cancer cases. Consistent with our proposed model, quantitative analysis revealed a strong positive correlation between LDHA or H3K18la and SCARB1 protein levels, as well as between H3K18la or SCARB1 and the phosphorylation of S6K1 (Figs. 5G,H and EV5D). These data reinforce the role of the lactate–SCARB1–mTORC1 axis in driving HCC progression.

Finally, the results of the Kaplan‒Meier test indicated that patients with low SCARB1 expression in their HCC lesions survived much longer than those with high expression did (Fig. 5I), suggesting that SCARB1 may represent a promising prognostic biomarker in HCC.

## Discussion

Cancer cells typically undergo comprehensive metabolic reprogramming to meet the demands of rapid proliferation and to adapt to the tumour microenvironment (DeBerardinis et al, 2008). Increased cholesterol biosynthesis is a hallmark of many cancers. Previous studies have shown that, in situations where lipids and/or oxygen are limited, the master transcription factor Sterol regulatory element-binding protein 2 (SREBP2) and its downstream targets, including mevalonate pathway enzymes, are significantly upregulated in tumours (Husain et al, 2022; Lewis et al, 2015). However, a small number of studies suggest that the activity of certain cholesterol biosynthetic enzymes is suppressed under hypoxia (Kucharzewska et al, 2015; Wu et al, 2020). Thus, the mechanism through which tumour cells reprogram cholesterol metabolism to adapt to hypoxic conditions remains unclear. In this study, we observed marked accumulation of cholesterol under hypoxic conditions (Fig. 1A). Interestingly, we demonstrated that the increase in cholesterol levels is attributed to increased cholesterol uptake, rather than de novo synthesis, as all genes involved in the cholesterol biosynthetic pathway were transcriptionally downregulated under hypoxic conditions (Fig. 1D). Therefore, our findings support a model in which cancer cells circumvent energy-intensive biosynthesis by prioritizing this more energy- and time-efficient mechanism to meet their high metabolic demands when they are situated in unfavourable conditions.

Although previous studies have reported that HIF-1α regulates cholesterol metabolism (Furuta et al, 2008; Hwang et al, 2017), emerging evidence now highlights the critical role of HIF-1-independent pathways in mediating the metabolic response to hypoxia (Dickson et al, 2023; Minikes et al, 2025). Here, we establish a novel paradigm in which lactate, rather than the canonical HIF-1α pathway, dominates cholesterol metabolic adaptation under hypoxia. The understanding of the role of lactate in cancer biology has evolved dramatically from being considered simply a glycolytic byproduct to recognition as a critical regulator of tumour progression (Chen et al, 2025). This paradigm shift has been further reinforced by the discovery of histone lactylation, an epigenetic modification that provides a direct mechanistic link between lactate metabolism and gene regulation (Zhang et al, 2019). Emerging evidence has demonstrated that histone lactylation plays multifaceted roles in tumour biology, such as promoting monocyte-derived macrophage (MDM) immunosuppressive activity and remodelling lipid metabolism to regulate immune responses and metabolic reprogramming (De Leo et al, 2024; Du et al, 2024). Our findings significantly expand this understanding by demonstrating that histone lactylation drives SCARB1 transcription (Fig. 2D–F), leading to increased cholesterol uptake in HCC cells under hypoxic conditions. Our study reveals a direct link between lactate and cholesterol metabolism and provides novel insights into how lactate orchestrates metabolic adaptation through epigenetic mechanisms.

Autophagy, the cellular self-digestion process mediated by autophagosome‒lysosome fusion, serves as a critical quality control mechanism by clearing damaged proteins, eliminating pathogens, and recycling cellular components during metabolic stress (Rabinowitz and White, 2010; Russell et al, 2014). Although autophagy is rapidly induced as a protective response to environmental challenges such as nutrient deprivation or hypoxia, its role in cancer biology remains complex and context dependent, with increasing evidence implicating autophagy in both tumour suppression and progression (Li et al, 2020b; White and DiPaola, 2009). While previous studies reported that SCARB1 localizes to lysosomes in macrophages, promoting autophagy through a peroxisome proliferator-activated receptor alpha (PPARα)–transcription factor EB (TFEB)-dependent transcriptional program to facilitate cholesterol clearance during atherosclerosis (Tao et al, 2021), we interestingly found that its deficiency enhances autophagy in HCC cells (Fig. 3D,F). This discrepancy likely reflects fundamental metabolic differences between immune and cancer cells, as SCARB1 predominantly localizes to the cancer cell membrane, where it facilitates cholesterol uptake. Moreover, the regulatory influence of lactate on autophagy appears to be bidirectional, with lactate promoting autophagy in certain contexts, while inhibiting it in others. A recent study revealed that lactate promotes TFEB activation and enhances autophagy in pancreatic cancer (Huang et al, 2024); however, Meng et al demonstrated that lactate-induced discoidin, CUB and LCCL domain-containing protein 1 (DCBLD1) lactylation suppresses its ubiquitination, thereby inhibiting autophagy and ultimately promoting cervical cancer progression (Meng et al, 2024). Therefore, the seemingly opposite effects of lactate-driven lactylation on autophagy may largely depend on the specific target proteins being modified. In our study, we report that lactate upregulates SCARB1 expression to enhance cholesterol uptake, thereby inhibiting autophagy, consistent with the fact that cholesterol has been shown to activate mTORC1 and suppress autophagy.

The regulation of autophagy by SCARB1 involves mTORC1, the master nutrient sensor that integrates multiple inputs, including energy status, oxygen levels, and growth signals, to maintain metabolic homeostasis (Dibble and Manning, 2013). Under hypoxic conditions, when mTORC1 activity is typically suppressed, we discovered that SCARB1 overexpression restored mTORC1 signalling and inhibited excessive autophagy (Fig. 4B). Consequently, we propose a novel mechanism through which tumour cells enhance their hypoxia adaptation: lactate-mediated upregulation of SCARB1 promotes cholesterol uptake in HCC cells, leading to partial mTORC1 reactivation. This process suppresses autophagy-associated cell death, thereby promoting cell survival and driving tumour progression (Figs. 4C,D and 4G,H).

Taken together, our results reveal a previously unrecognized mechanism through which HCC cells adapt to hypoxic conditions through the reprogramming of cholesterol metabolism. Specifically, lactate enhances histone lactylation, thereby upregulating SCARB1 expression. Elevated SCARB1 facilitates cholesterol uptake to reactivate mTORC1 signalling, which in turn protects HCC cells from excessive autophagy. These findings provide new insights into lactate-driven metabolic reprogramming and highlight the critical role of the lactate-SCARB1-mTORC1 axis in liver cancer progression.

## Methods

**Table:** Reagents and tools table

Reagent/resource	Reference or source	Identifier or catalog number
**Experimental models**		
Hep3B cells (*H. sapiens*)	ATCC	HB-8064
PLC cells (*H. sapiens*)	ATCC	CRL8024
HEK293T (*H. sapiens*)	ATCC	CL-0005
BALB/c nude mice	GemPharmatech	N/A
**Recombinant DNA**		
pCDH-Flag-SCARB1	This study	
pCDH-Flag-p300-HAT	This study	
pCDH-Flag-MCT4	This study	
**Antibodies**		
Rabbit anti-SCARB1	Abcam	ab52629
Rabbit anti-LDHA	Proteintech	21799-1-AP
Mouse anti-β-Actin	Proteintech	60008-1-Ig
Rabbit anti-HIF-1α	Cell Signalling Technology	36169T
Rabbit anti-Pan Kla	Jingjie	PTM-1401RM
Rabbit anti-Histone H3	Proteintech	17168-1-AP
Rabbit anti-H3K9la	Jingjie	PTM-1419RM
Rabbit anti-H3K18la (for ChIP)	Jingjie	PTM-1427RM
Rabbit anti-H3K18la	Jingjie	PTM-1406RM
Rabbit anti-H3K14la	Jingjie	PTM-1414RM
Rabbit anti-H3K23la	Jingjie	PTM-1413RM
Rabbit anti-H3K56la	Jingjie	PTM-1421RM
Rabbit anti-H4K5la	Jingjie	PTM-1407RM
Rabbit anti-H4K8la	Jingjie	PTM-1415RM
Rabbit anti-H4K12la	Jingjie	PTM-1411RM
Rabbit anti-Histone H4	Proteintech	16047-1-AP
Rabbit anti-LC3	Proteintech	14600-1-AP
Rabbit anti-phospho-T389 S6K1	Cell Signalling Technology	9234T
Rabbit anti-S6K1	Cell Signalling Technology	2708T
Rabbit anti-phospho-T389 S6K1 (for IHC)	Proteintech	28735-1-AP
Rabbit anti-MCT1	Proteintech	20139-1-AP
Rabbit anti-MCT4	Proteintech	22787-1-AP
Mouse anti-p300	Santa Cruz	sc-48343
Rabbit anti-Flag tag	Proteintech	20543-1-AP
Rabbit anti-phospho-ULK1 (Ser757)	Cell Signalling Technology	14202T
Rabbit anti-ULK1	Proteintech	20986-1-AP
Rabbit anti-phospho-4E-BP1 (Ser65)	Cell Signalling Technology	9451T
Rabbit anti-4E-BP1	Proteintech	13988-1-AP
Rabbit anti-GPX4	Abcam	Ab125066
Rabbit anti-phospho-MLKL (Ser 358)	Proteintech	82090-2-RR
Rabbit anti-MLKL	Proteintech	66675-1-Ig
Rabbit anti-Cleaved Caspase-3 (Asp175)	Cell Signalling Technology	9661T
Rabbit anti-Caspase-3	Cell Signalling Technology	9662S
**Oligonucleotides and other sequence-based reagents**		
Homo LDHA	GGCTACAACAGGATTCTA	TTACAAACCATTCTTATTTCTAAC
Homo SCARB1	ACTTCTGGCATTCCGATCAGT	ACGAAGCGATAGGTGGGGAT
Homo HMGCR	TGATTGACCTTTCCAGAGCAAG	CTAAAATTGCCATTCCACGAGC
Homo HMGCS1	GATGTGGGAATTGTTGCCCTT	ATTGTCTCTGTTCCAACTTCCAG
Homo MVK	CCTTTCGGAAGGACATGATCC	TCTCCGTGTGTCACTCACCA
Homo MVD	GGACCGGATTTGGCTGAATG	CCCATCCCGTGAGTTCCTC
Homo IDI1	TCCATTAAGCAATCCAGCCGA	CCCAGATACCATCAGACTGAGC
Homo SQLE	GGCATTGCCACTTTCACCTAT	GGCCTGAGAGAATATCCGAGAAG
Homo LSS	GTACGAGCCCGGAACATTCTT	CGGCGTAGCAGTAGCTCAT
Homo FDFT1	ACTTCCCAACGATCTCCCTTG	CCCATTCTCCGGCAAATGTC
Homo ACAT2	CCCAGCCAATGCTTCAGGAAT	AAGCCCACGTTTATCAGCTTC
Homo LDLR	AGTTGGCTGCGTTAATGTGA	TGATGGGTTCATCTGACCAGT
Homo VLDLR	CTGGGTATGCGACGATGATG	CTTGGTGTGTATGACTGGCTG
Homo NPC1L1	AGAGTGAGCCTTACACAACCA	GCAGGACACGTTGGAGAGT
SCARB1-promoter	TTCTTCCAGGGACAGTGAGG	CTCCTCTATCGGGACCATCA
ShSCARB1-1	*Homo sapiens*	CCGGCAAGGACAAGTTCGGATT ATTCTCGAGAATAATCCGAACTT GTCCTTGTTTTTG
ShSCARB1-2	*Homo sapiens*	CCGGAGCCAAGAGAAATGCTATT TACTCGAGTAAATAGCATTTCTCT TGGCTTTTTTG
shLDHA-1	*Homo sapiens*	CCGGGAACGGTATTACCAACCTT ATCTCGAGATAAGGTTGGTAATAC CGTTCTTTTTG
shLDHA-2	*Homo sapiens*	CCGGCCACCATGATTAAGGGTCT TTCTCGAGAAAGACCCTTAATCAT GGTGGTTTTTG
shMCT1-1	*Homo sapiens*	CCGGATCAGTCTTCCAAACAATTA ACTCGAGTTAATTGTTTGGAAGA CTGATTTTTTG
shMCT1-2	*Homo sapiens*	CCGGCATGTGGCGTCGTCCTAATT ACTCGAGTAATTAGGACGACGCC ACATGTTTTTG
shMCT4-1	*Homo sapiens*	CCGGCGTCTACATGTACGTGTTCA TCTCGAGATGAACACGTACATGTA GACGTTTTTG
shMCT4-2	*Homo sapiens*	CCGGCATCTTCTTTGGCATCTCCTA CTCGAGTAGGAGATGCCAAAGAA GATGTTTTTG
shP300	*Homo sapiens*	CCGGATGTTGCATTCAGCCATAAA TCTCGAGATTTATGGCTGAATGCA ACATTTTTTG
shHIF-1α	*Homo sapiens*	CCGGTGCTCTTTGTGGTTGGATC TACTCGAGTAGATCCAACCACAA AGAGCATTTTT
**Chemicals, enzymes and other reagents**		
Sodium Oxamate	Sangon Biotech	A600871
HEPES	Thermo Fisher Scientific	15630-080
Sodium L-lactate	Sigma-Aldrich	71718
2-DG	MCE	HY-13966
C646	MCE	HY-13823
Cholesterol	Sigma-Aldrich	C3045
3-MA	MCE	HY-19312
Z-VAD-FMK	MCE	HY-16658B
Necrostatin-1	MCE	HY-15760
Ferr-1	MCE	HY-100579
BLT-1	MCE	HY-116767
Rapamycin	MCE	AY-22989
Methyl-β-cyclodextrin	Sigma-Aldrich	C4555
Fetal Bovine Serum	Biological Industries	04-001-1ACS
Lipid Depleted Fetal Bovine Serum	VivaCell Biosciences	C3840-0050
Medium Dulbecco’s Modified Eagle Medium	Thermo Fisher Scientific	12100061
Penicillin-Streptomycin Solution	Biological Industries	03-031-1B
0.25% Trypsin	Biological Industries	B03-050-1A
Opti-MEM	Thermo Fisher Scientific	31985-088
Puromycin	Sigma-Aldrich	P8833-100mg
Polybrene	Sigma-Aldrich	H9268-5G
Cocktail	Sigma-Aldrich	5056489001
Dimethyl Sulfoxide	Sangon Biotech	A100231-0500
PMSF	Sangon Biotech	A610425-0005
Protein A/G Beads	Thermo Fisher Scientific	53133
Trizol	Thermo Fisher Scientific	15596-018
T4 ligase	Thermo Fisher Scientific	15224017
Ampicillin sodium	Sangon Biotech	A610028-0025
Paraformaldehyde-glutaraldehyde	Leagene Biotechnology	DF0139
Ringer’s solution	Leagene Biotechnology	CZ0045
Propidium iodide solution (PI)	Sigma-Aldrich	P4864
Total Cholestenone (TC) Content Assay Kit	Sangon Biotech	D799800
Bafilomycin A1	MCE	HY-100558
Ad-mCherry-GFP-LC3B	Beyotime	C3011
DiI-HDL	Solarbio	IL2170
**Software**		
GraphPadPrism8	https://www.graphpad.com/	
ImageJ	https://imagej.net/ij/	
FlowJo 10.0	https://www.flowjo.com/	
**Other**		

### Methods and protocols

Our research complies with all relevant ethical regulations of the South China University of Technology.

### Cell culture and reagents

Human liver cancer cell lines (Hep3B and PLC) and a human renal epithelial cell line (HEK293T) were maintained in Dulbecco’s modified Eagle’s medium (DMEM) supplemented with 10% foetal bovine serum (FBS) and 1% penicillin‒streptomycin. All cell lines were tested for mycoplasma contamination, and no cell lines were contaminated. All cells were cultured at 37 °C with 5% CO_2_ in humidified incubators. Hypoxia exposure was achieved by placing cells into a modular chamber (Don Whitley Scientific) flushed with a gas mixture consisting of 1% O_2_, 5% CO_2_, and 94% N_2_ at 37 °C. All drugs used in this study are listed in the Reagents and Tools Table.

### Western blotting assay

Cultured cells and tissues were lysed in RIPA buffer (50 mM Tris-HCl (pH 8.0), 150 mM NaCl, 5 mM EDTA, 0.1% SDS, and 1% NP-40) supplemented with protease inhibitor cocktails and 100 μM phenylmethylsulfonyl fluoride (PMSF) for 45 min on ice, after which the cell lysate samples were analysed using a Bradford assay kit. After denaturation, equal amounts of proteins were separated by 7–13% SDS‒PAGE and transferred to NC membranes, followed by blocking with 5% skim milk in TBST for 1 h. Subsequently, the blots were incubated with the indicated primary antibodies and HRP-conjugated secondary antibodies according to recommended protocols. The signals were visualized using an ECL kit and a chemiluminescence imaging system (Tanon-5200). All primary antibodies used for immunoblotting are listed in the Reagents and Tools Table.

### Lentivirus production and infection

Short-hairpin oligonucleotides (shRNAs) directed against SCARB1 and LDHA were cloned and inserted into the pLKO.1 lentiviral vector. Polymerase chain reaction (PCR)-amplified SCARB1 was subcloned and inserted into the pCDH-3×Flag, and GFP-LC3 was subcloned and inserted into the pSin-GFP according to the manufacturer’s instructions. Lentiviruses were produced by cotransfecting HEK293T cells with the indicated plasmids and packaging vectors (psPAX2 and pMD2.G) into HEK293T packaging cells using a PEI transfection reagent. For lentivirus infection, target cells were infected with viral supernatants containing 8 μg/mL polybrene for 6 h, after which the cells were supplemented with fresh media. Stable cell lines were selected by treatment with 0.5 μg/mL puromycin. shRNA targeting sequences are listed in the Reagents and Tools Table.

### Quantitative real-time PCR

Total cellular RNA was extracted using TRIzol reagent, and complementary DNA was synthesized from 3 µg of RNA using the HiScript II First Strand cDNA Synthesis Kit. Quantitative PCR assays were performed in triplicate with diluted cDNA, the indicated primers, and ChamQ^TM^ Universal SYBR qPCR Master Mix according to the manufacturer’s instructions. All samples were normalized to the expression of the housekeeping gene 18S, and the data were analysed via the 2^−ΔΔCt^ method. The sequences of the primers are listed in the Reagents and Tools Table.

### DiI-HDL uptake assay

The cholesterol uptake assay in HCC cells was performed using DiI-HDL. Cells were starved in serum-free DMEM for 6 h, then DiI-HDL (25 μg/mL) was added and incubated for 6 h. After incubation, cells were fixed with 0.3% formaldehyde for 15 min and subsequently incubated with DAPI for 5 min. Images were acquired and analysed with a confocal microscope system (Zeiss LSM 800).

### ChIP‒qRT‒PCR assay

The ChIP assay was performed using an EZ-ChIP Kit (Millipore) following the manufacturer’s instructions. Briefly, after being crosslinked by incubation with 1% formaldehyde (which was then quenched with 0.125 M glycine), the cells were sonicated to obtain randomly fragmented genomic DNA using an ultrasonic cell disruptor. DNA was immunoprecipitated with control IgG, H3K18la, or HIF-1α primary antibodies at 4 °C overnight. Protein A/G magnetic beads were added to the lysate the following morning, and the protein‒DNA complexes were subsequently eluted from the magnetic beads. RNA and protein were digested with RNase A and proteinase K, respectively. ChIP DNA and input DNA were analysed by qPCR. The sequences of the ChIP-Primers are listed in the Reagents and Tools Table.

### Cell proliferation assays

Cell proliferation was measured by cell counting. A total of 4 × 10^4^ cells from each cell line were seeded in 12-well plates in triplicate and maintained with DMEM supplemented with 10% FBS, 10% delipidated FBS, or 10% delipidated FBS supplemented with cholesterol, as indicated in the figures. The cells were placed in a standard incubator (normoxia) or a hypoxic incubator the following morning. The cells were counted every 24 h, and cell proliferation curves were plotted.

### RNA sequencing and data analysis

Total RNA was extracted from cell lines using TRIzol reagent. RNA integrity was assessed by determining the RNA integrity number using an Agilent 2100 Bioanalyzer. A total of 3 µg of RNA per sample was used for analysis. Sequencing was performed with three biological replicates. Libraries were generated using the NEBNext Ultra RNA Library Prep Kit for Illumina (NEB). RNA-seq was performed on an Illumina NovaSeq 6000 platform by Novogene (Tianjin). Reads were aligned to the human genome hg19. TopHat2 v.2.1.0 and Cufflinks v.2.2.1 were used to analyse the RNA-seq data. Gene differential expression analysis was carried out with the DEGSeq R package (1.26.0).

### Cell death assay

Cells were seeded into six-well plates one day before drug treatment. Two days after treatment, the cells were harvested and stained with 5 μg/mL propidium iodide (PI) for 10 min. Then, the stained cells were washed once with PBS and analysed using a BD FACSCelesta^TM^ flow cytometer. All analyses of flow cytometry data were performed using FlowJo 10.0 software.

### Intracellular cholesterol detection

The total cholesterol content in cells or tissues was measured with a Total Cholesterol Content Assay Kit, following the manufacturer’s instructions. Briefly, equal numbers of cells or equal amounts of tissue were collected and extracted with 200 μL of isopropanol extraction solution. After sonication and centrifugation, the supernatant was incubated with the reaction mixture at 37 °C for 15 min, and the absorbance was measured in a microplate reader at 500 nm.

### Immunofluorescence

Adherent cells were fixed with 4% paraformaldehyde for 20 min, gently washed with PBS twice, and then cells were stained with DAPI for 5 min. Images were acquired and analysed with a confocal microscope system (Zeiss LSM 800).

### Animal studies

All animal experiments and procedures were performed in compliance with ethical regulations and with the approval of the South China University of Technology (AEC number 2023140). The mice were housed at a suitable temperature (22–24 °C) and humidity (40–70%) under a 12/12-h light/dark cycle with unrestricted access to food and water throughout the experiment. For the xenograft experiments, 5 × 10^6^ Hep3B cells stably overexpressing SCARB1 or expressing shSCARB1 were subcutaneously inoculated into the flank of 5-week-old male nude mice. Before injection, the cells were resuspended in DMEM without FBS and mixed with Matrigel to a final volume of 100 μL per injection. Tumour size was measured every 3 days using callipers and converted to volume using the following formula: length (mm)× width^2^ (mm)× 0.52. Tumour volumes, tumour weights, images, and other experimental indicators were obtained at the end of the experimental period.

For sodium lactate treatment, daily doses of PBS or 1 g/kg sodium lactate were administered intraperitoneally 7 days before the Hep3B cells were inoculated. Injections were continued throughout the experiments.

For rapamycin treatment, one week after the Hep3B cells were subcutaneously inoculated, the mice bearing tumours were administered 5 mg/kg rapamycin intraperitoneally every 2 days.

Mouse liver samples derived from the YAP-5SA–induced and NRAS/shp53-induced HCC models were obtained from our previous study (Zhang et al, 2022).

### Clinical human HCC specimens

Snap-frozen HCC tissues and corresponding noncancerous tissues that were at least 2 cm from the edge of tumours were collected from patients with HCC at the First Affiliated Hospital of the University of Science and Technology of China. Total protein was extracted from paired HCC and noncancerous tissues and then measured by immunoblotting. Formalin-fixed, paraffin-embedded HCC tissues and adjacent noncancerous tissues were also collected from patients with HCC at the First Affiliated Hospital of the University of Science and Technology of China. Patients provided written informed consent to use these clinical materials for research purposes, and the study was approved by the Institutional Research Ethics Committee of the First Affiliated Hospital of the University of Science and Technology of China. All patients volunteered and received no compensation. The ethical approval number is 2025-ky156 and detailed patient information is provided in Table EV1.

### IHC

Immunohistochemistry was performed according to standard protocols. In brief, the samples were dewaxed with xylene and then rehydrated with graded ethanol. After antigen retrieval, the sections were incubated with hydrogen peroxide for 10 min to block endogenous peroxidase activity. Next, the sections were incubated with anti-H3K18la (PTM, 1:100), anti-SCARB1 (Abcam, 1:200), anti-LDHA (Proteintech, 1:500), and anti-phospho-T389 S6K1 (Proteintech, 1:80) antibodies at room temperature for 4 h. After incubation with HRP-conjugated secondary antibodies, the sections were visualized with DAB. Images of IHC staining were acquired with a Leica AperioCS2. Quantitative analysis of IHC staining was performed with ImageJ (IHC Profiler).

### Statistical analysis

Two-tailed unpaired Student’s *t* test and one- and two-way analysis of variance (ANOVA) were used to calculate *P* values unless otherwise specified in the figure legends. Statistical analysis was performed using GraphPad Prism 8.0. All data are presented as the mean ± SEM or mean ± SD. *P* < 0.05 was considered significant. The mice were randomly grouped before the different treatments. In in vitro studies, cells or conditions were assigned randomly to each experimental group. Data collection and analysis were not performed blind to the conditions of experiments, except for the acquisition and analysis of immunohistochemistry and immunofluorescence images, which were performed in a blinded manner.

## Supplementary information

Table EV1

Peer Review File

Source data Fig. 1

Source data Fig. 2

Source data Fig. 3

Source data Fig. 4

Source data Fig. 5

Figure EV1 Source Data

Figure EV2 Source Data

Figure EV3 Source Data

Figure EV4 Source Data

Figure EV5 Source Data

Expanded View Figures

## Data Availability

The RNA-seq data produced in this study were deposited in the public database Gene Expression Omnibus (GEO) GSE316557. The survival analysis was performed using the GEPIA database (http://gepia.cancer-pku.cn/) with the following parameters: gene = SCARB1, cancer type = LIHC, and median expression cutoff for grouping. The source data of this paper are collected in the following database record: biostudies:S-SCDT-10_1038-S44319-026-00829-x.
