# Supplementary material for: Lactate-mediated cholesterol uptake promotes liver cancer progression via the SCARB1-autophagy axis
Source: EMBO Rep. 2026 Jun 10;27(14):4141–65. doi: 10.1038/s44319-026-00829-x (PMC13400630; doi:10.1038/s44319-026-00829-x)
Supplement: Supplementary file 1 — Table EV1 [file 44319_2026_829_MOESM1_ESM.docx]

Table EV1. Clinical information of human HCC patients used in this study.

|  | ID | Sex | Age (Years) | Tumor | Liver Disease(s) | Ethnicity |
| --- | --- | --- | --- | --- | --- | --- |
|  | 02682438 | Male | 54 | HCC | HBV | Asian |
|  | 02697377 | Male | 62 | HCC | HBV | Asian |
|  | 2909695 | Male | 58 | HCC | HBV | Asian |
|  | 2918122 | Male | 46 | HCC | HBV | Asian |
|  | 02915290 | Male | 65 | HCC | HBV | Asian |
|  | 12812937 | Male | 52 | HCC | HBV | Asian |
|  | 2921732 | Male | 47 | HCC | HBV | Asian |
|  | 2926620 | Male | 50 | HCC | HBV | Asian |
|  | 2931297 | Female | 65 | HCC | HBV | Asian |
|  | 02923916 | Female | 42 | HCC | NO | Asian |
|  | 2936251 | Male | 74 | HCC | HBV | Asian |
|  | 2930975 | Male | 67 | HCC | HBV | Asian |
|  | 2935458 | Male | 71 | HCC | HBV | Asian |
|  | 2940663 | Male | 63 | HCC | HBV | Asian |
|  | 2929979 | Male | 53 | HCC | HBV | Asian |
|  | 2935846 | Male | 49 | HCC | HBV | Asian |
|  | 2949671 | Male | 69 | HCC | HBV | Asian |
|  | 2963212 | Male | 32 | HCC | HBV | Asian |
|  | 2972170 | Male | 71 | HCC | HBV | Asian |
|  | 2978296 | Male | 49 | HCC | HBV | Asian |
|  | 2812571 | Male | 67 | HCC | HBV | Asian |
|  | 2973850 | Female | 70 | HCC | NO | Asian |
|  | 2984773 | Male | 54 | HCC | HBV | Asian |
|  | 23191597 | Male | 71 | HCC | NO | Asian |
|  | 12758785 | Male | 73 | HCC | HBV | Asian |
|  | 16331689 | Male | 70 | HCC | NO | Asian |
|  | 3142903 | Female | 63 | HCC | HBV | Asian |
|  | 9740800 | Male | 61 | HCC | HBV | Asian |
|  | 9815744 | Male | 54 | HCC | HBV | Asian |
|  | 17487509 | Male | 47 | HCC | HBV | Asian |
|  | 0003434235 | Male | 77 | HCC | HBV | Asian |
|  | 0003099645 | Male | 59 | HCC | HBV | Asian |
|  | ID | Sex | Age (Years) | Tumor | Liver Disease(s) | Ethnicity |
|  | 0010368924 | Male | 57 | HCC | HBV | Asian |
|  | 0009555537 | Male | 58 | HCC | HBV | Asian |
|  | 0011465932 | Male | 54 | HCC | HBV | Asian |
|  | 17503316 | Male | 51 | HCC | HBV | Asian |
|  | 0001709301 | Male | 49 | HCC | HBV | Asian |
|  | 0011532847 | Male | 71 | HCC | HBV | Asian |
|  | 0011250174 | Female | 49 | HCC | HBV | Asian |
|  | 0011286553 | Female | 35 | HCC | HBV | Asian |
|  | 0002152656 | Male | 72 | HCC | HBV | Asian |
|  | 0001453605 | Male | 63 | HCC | HBV | Asian |
|  | 0009483248 | Male | 69 | HCC | HBV | Asian |
|  | 0011892456 | Female | 65 | HCC | HBV | Asian |
|  | 0004184167 | Male | 69 | HCC | HBV | Asian |
|  | 0000158883 | Male | 33 | HCC | HBV | Asian |
