# Supplementary figures and images for: Lactate-mediated cholesterol uptake promotes liver cancer progression via the SCARB1-autophagy axis (part 1 of 2)
Source: EMBO Rep. 2026 Jun 10;27(14):4141–65. doi: 10.1038/s44319-026-00829-x (PMC13400630; doi:10.1038/s44319-026-00829-x)

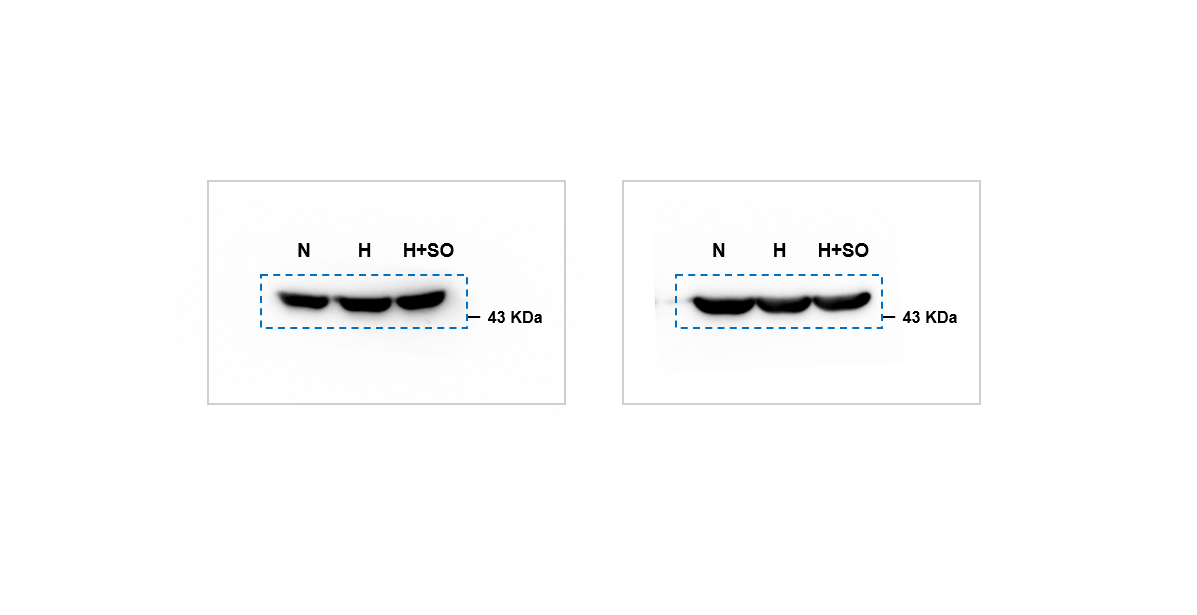

Supplement: Supplementary file 3 — Source data Fig. 1 [file 44319_2026_829_MOESM3_ESM.zip › Figure 1/E/western blot/ACTIN.png]

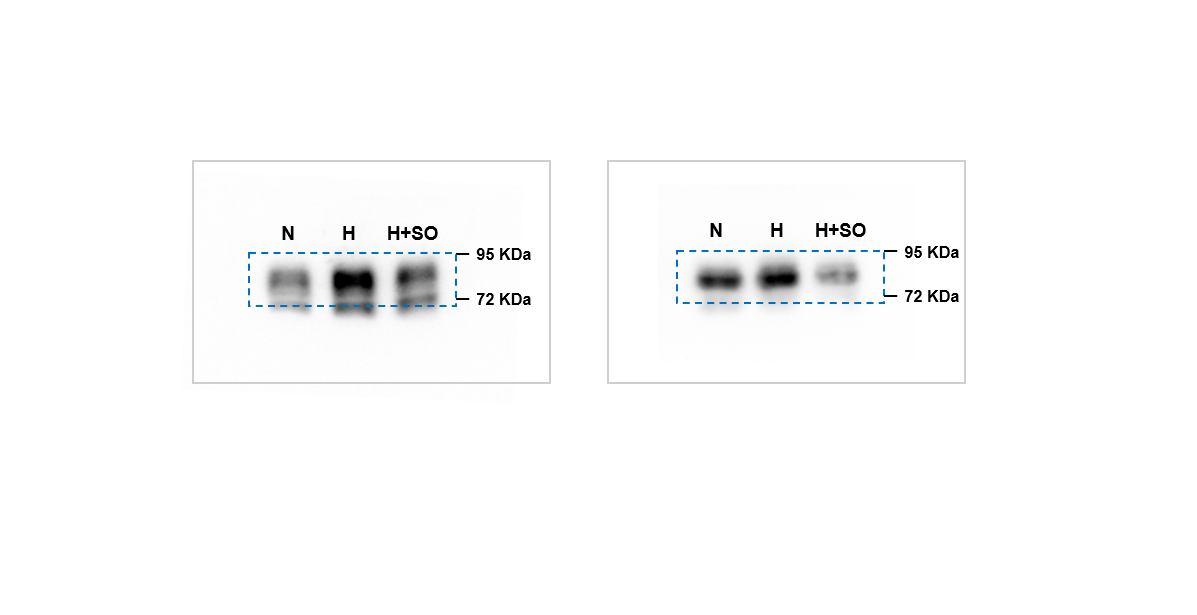

Supplement: Supplementary file 3 — Source data Fig. 1 [file 44319_2026_829_MOESM3_ESM.zip › Figure 1/E/western blot/SCARB1.png]

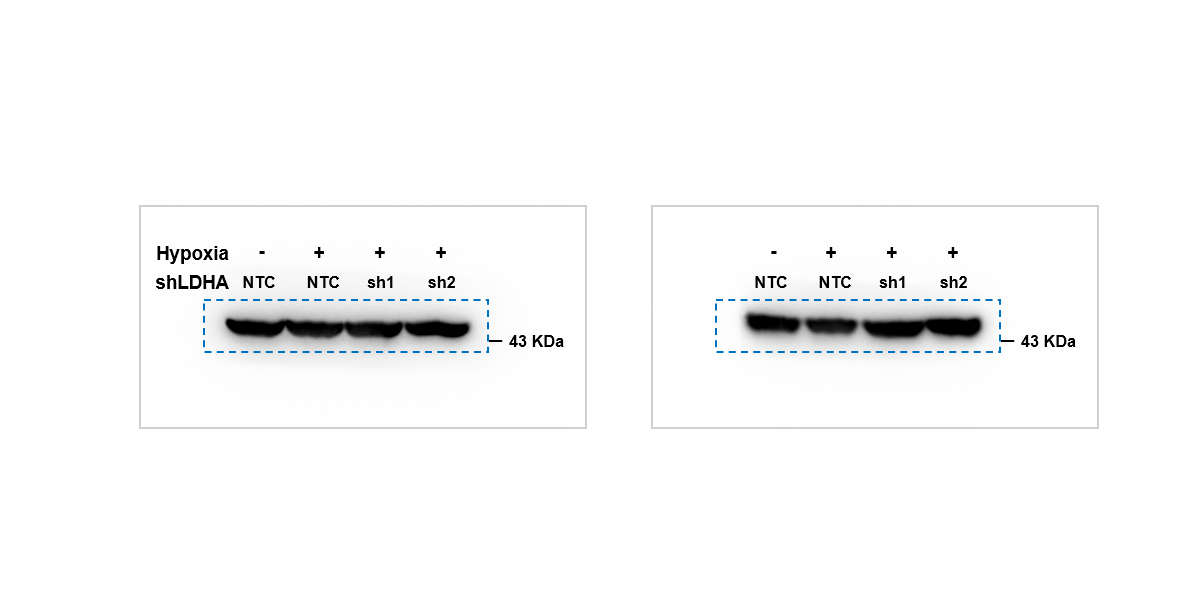

Supplement: Supplementary file 3 — Source data Fig. 1 [file 44319_2026_829_MOESM3_ESM.zip › Figure 1/F/western blot/ACTIN.png]

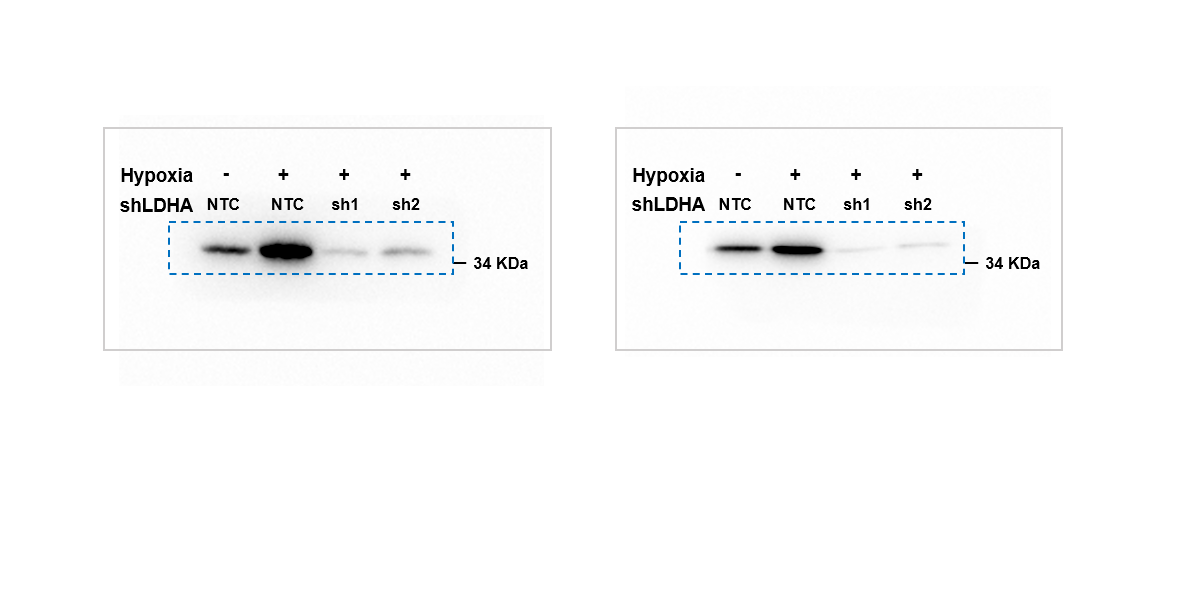

Supplement: Supplementary file 3 — Source data Fig. 1 [file 44319_2026_829_MOESM3_ESM.zip › Figure 1/F/western blot/LDHA.png]

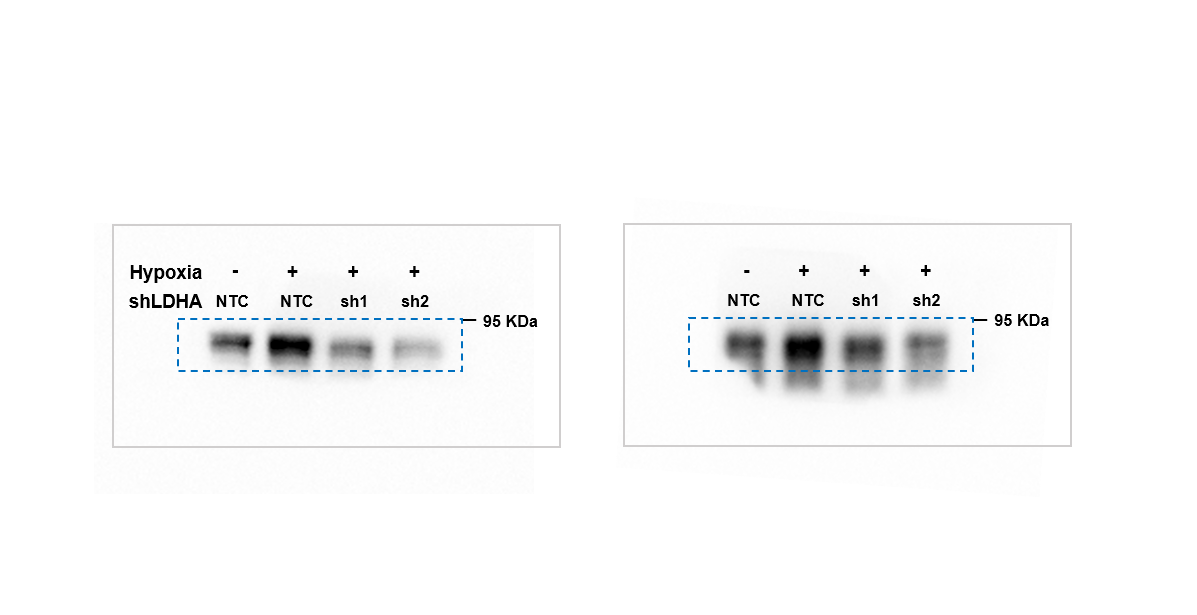

Supplement: Supplementary file 3 — Source data Fig. 1 [file 44319_2026_829_MOESM3_ESM.zip › Figure 1/F/western blot/SCARB1.png]

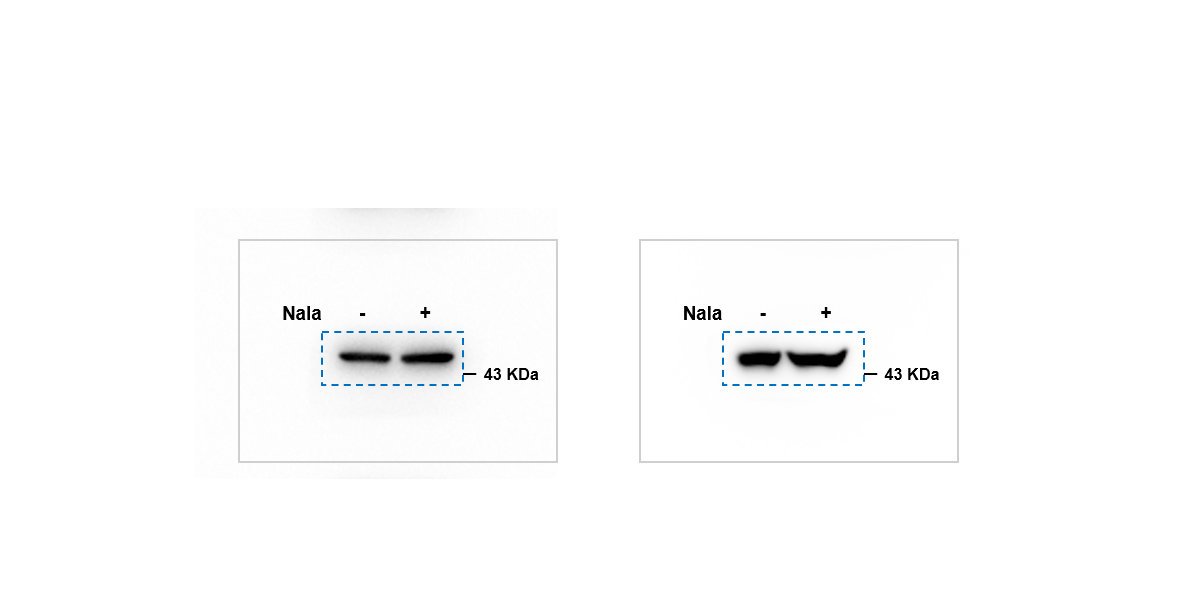

Supplement: Supplementary file 3 — Source data Fig. 1 [file 44319_2026_829_MOESM3_ESM.zip › Figure 1/G/western blot/ACTIN.png]

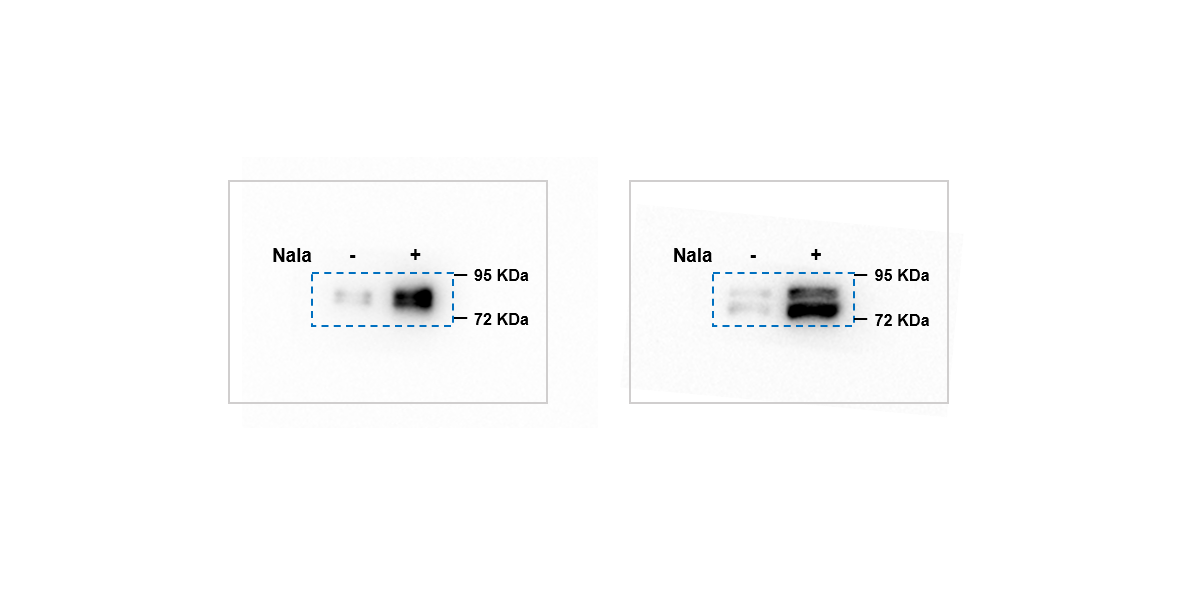

Supplement: Supplementary file 3 — Source data Fig. 1 [file 44319_2026_829_MOESM3_ESM.zip › Figure 1/G/western blot/SCARB1.png]

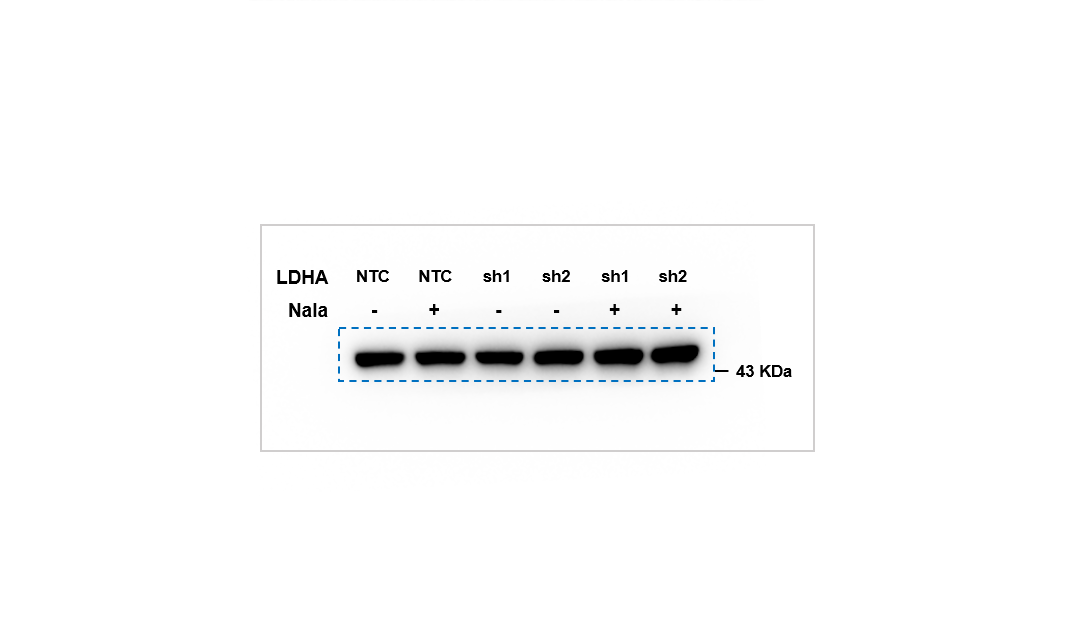

Supplement: Supplementary file 3 — Source data Fig. 1 [file 44319_2026_829_MOESM3_ESM.zip › Figure 1/H/ACTIN.png]

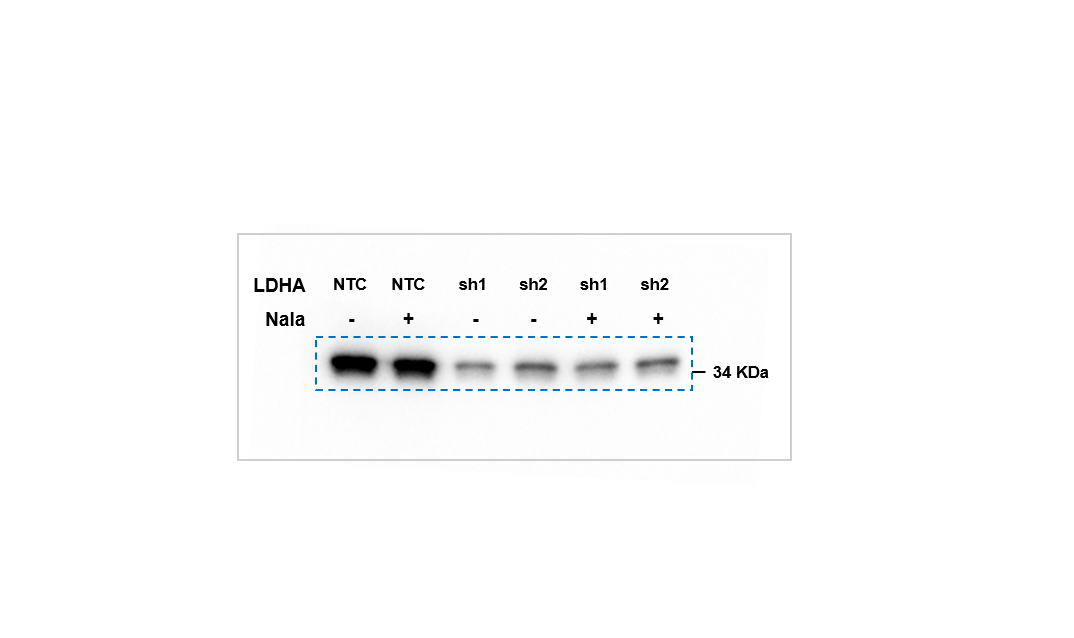

Supplement: Supplementary file 3 — Source data Fig. 1 [file 44319_2026_829_MOESM3_ESM.zip › Figure 1/H/LDHA.png]

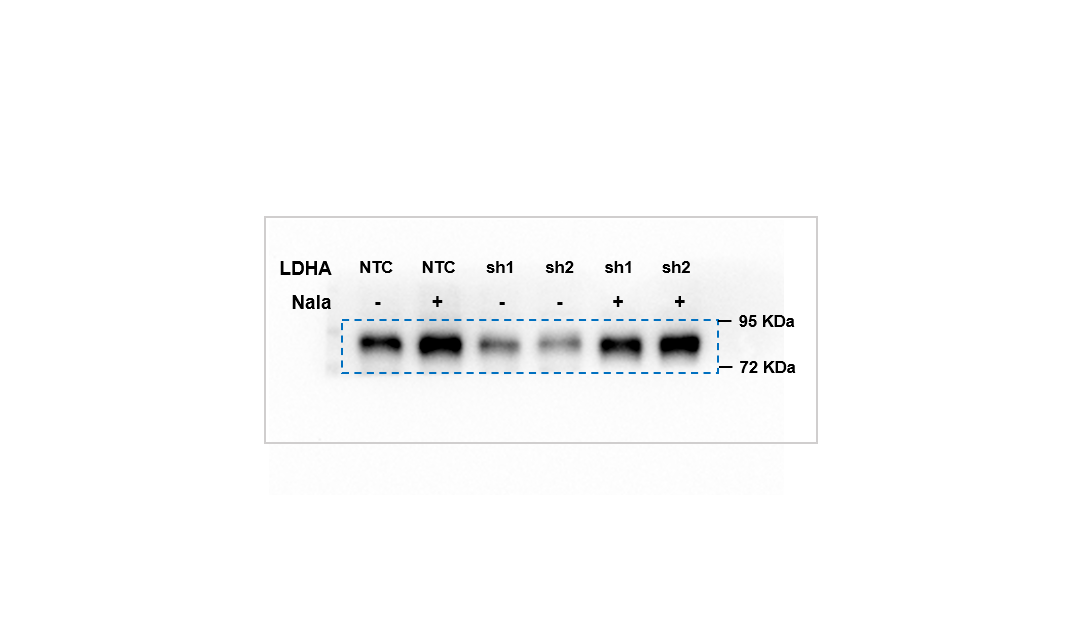

Supplement: Supplementary file 3 — Source data Fig. 1 [file 44319_2026_829_MOESM3_ESM.zip › Figure 1/H/SCARB1.png]

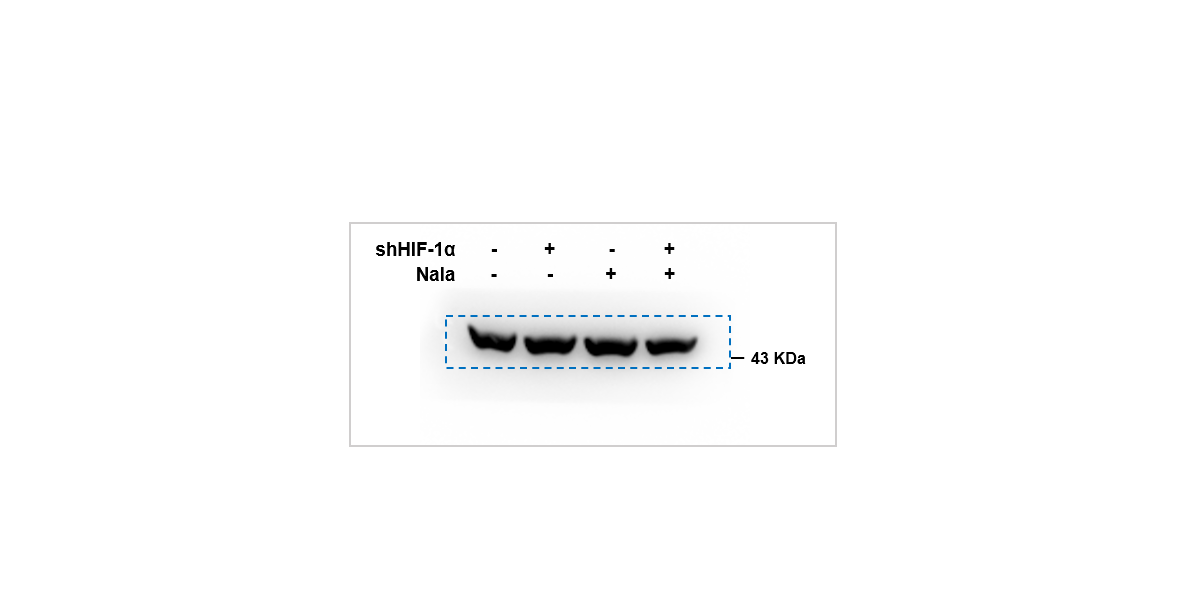

Supplement: Supplementary file 4 — Source data Fig. 2 [file 44319_2026_829_MOESM4_ESM.zip › Figure 2/B/ACTIN.png]

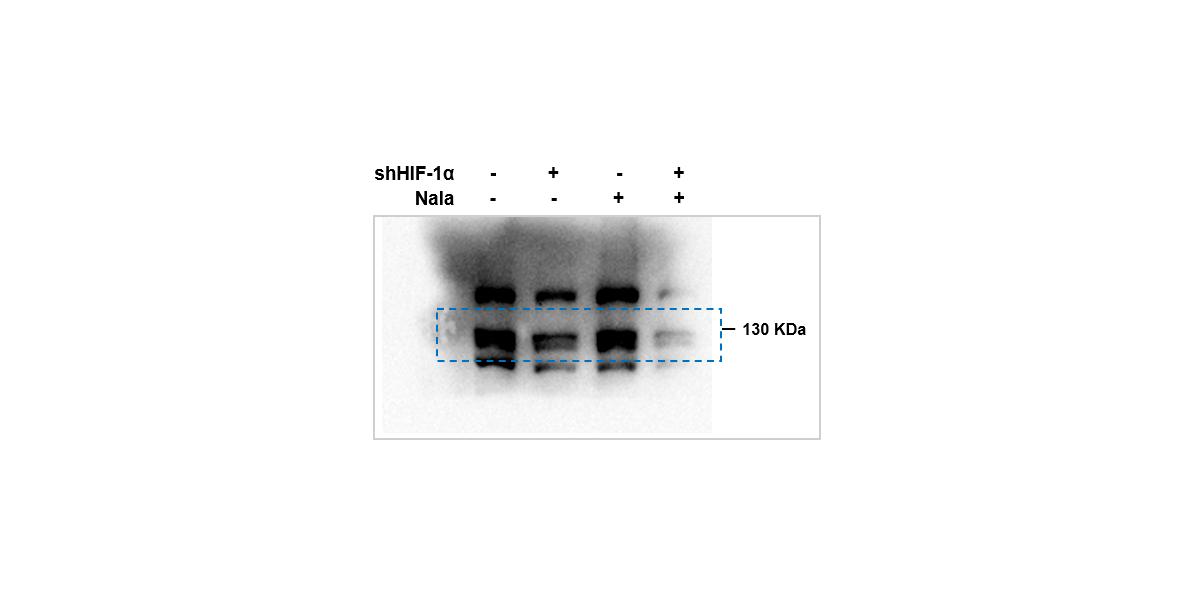

Supplement: Supplementary file 4 — Source data Fig. 2 [file 44319_2026_829_MOESM4_ESM.zip › Figure 2/B/HIF-1α.png]

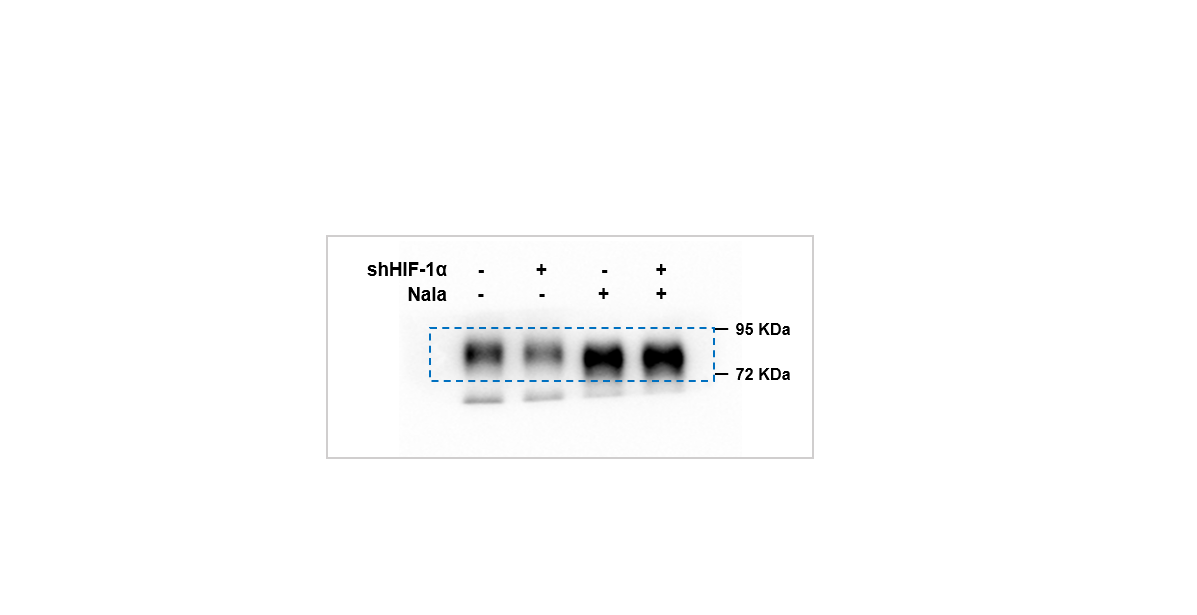

Supplement: Supplementary file 4 — Source data Fig. 2 [file 44319_2026_829_MOESM4_ESM.zip › Figure 2/B/SCARB1.png]

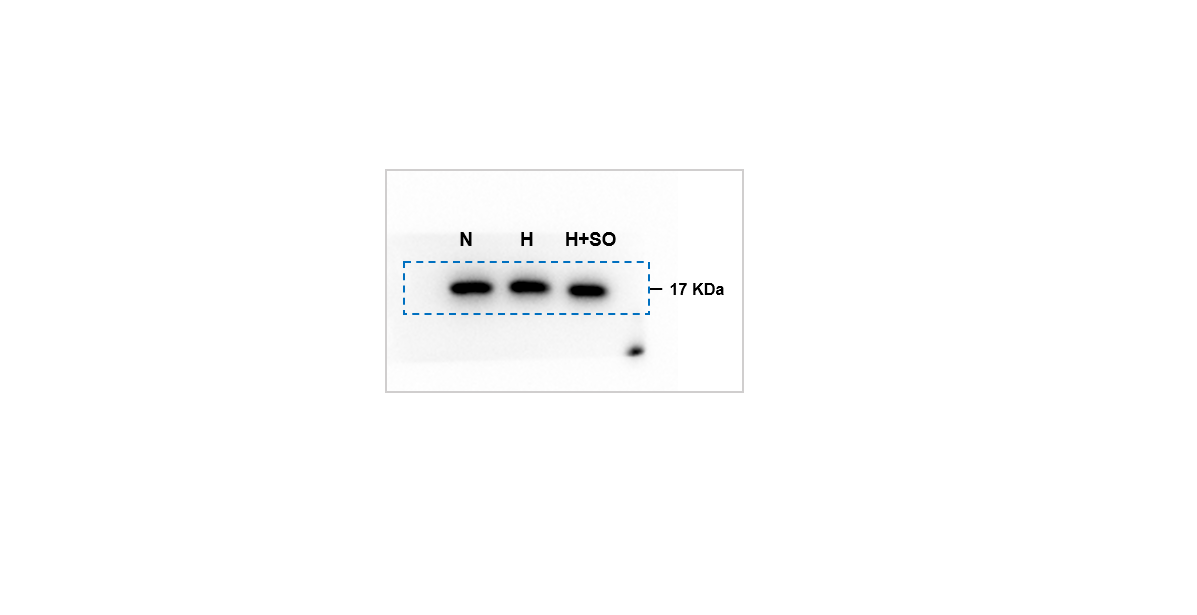

Supplement: Supplementary file 4 — Source data Fig. 2 [file 44319_2026_829_MOESM4_ESM.zip › Figure 2/C/H3.png]

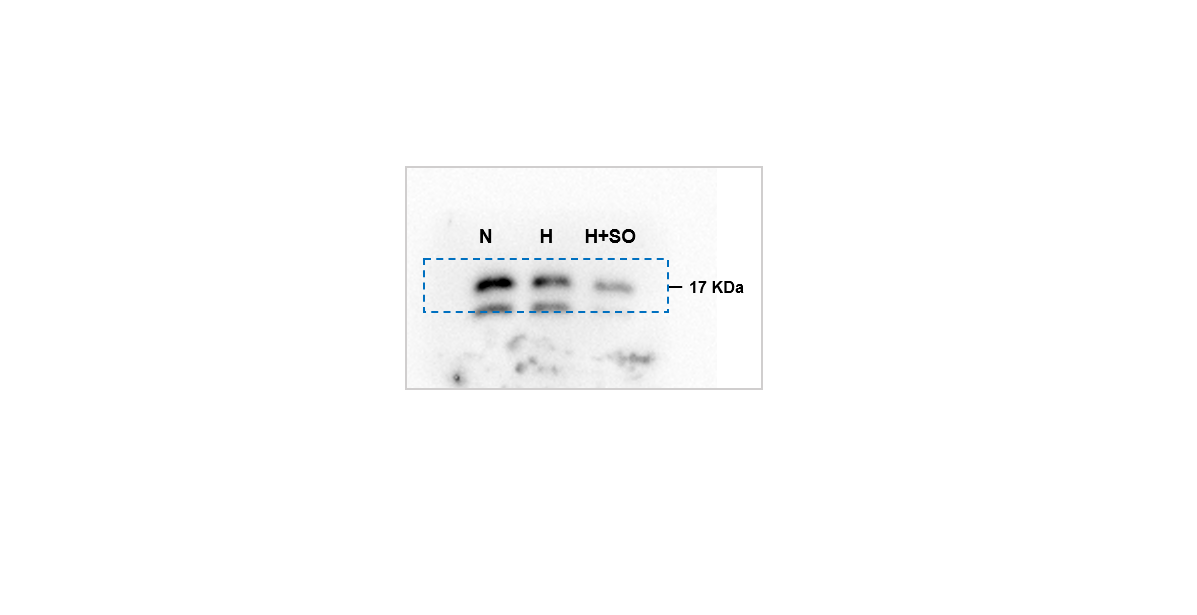

Supplement: Supplementary file 4 — Source data Fig. 2 [file 44319_2026_829_MOESM4_ESM.zip › Figure 2/C/H3K14la.png]

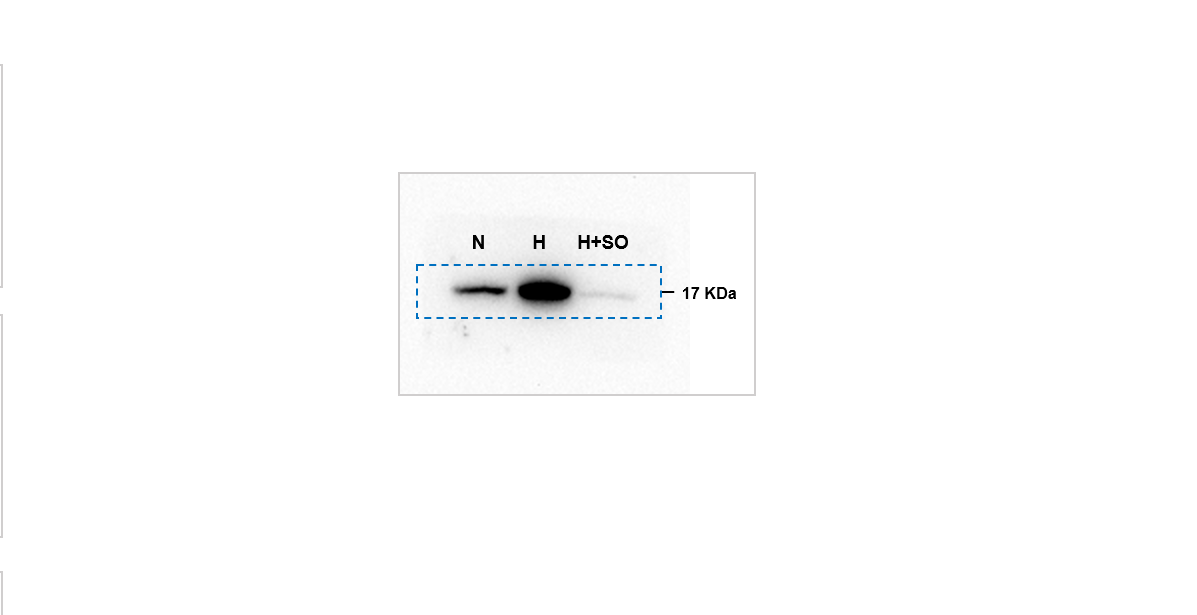

Supplement: Supplementary file 4 — Source data Fig. 2 [file 44319_2026_829_MOESM4_ESM.zip › Figure 2/C/H3K18la.png]

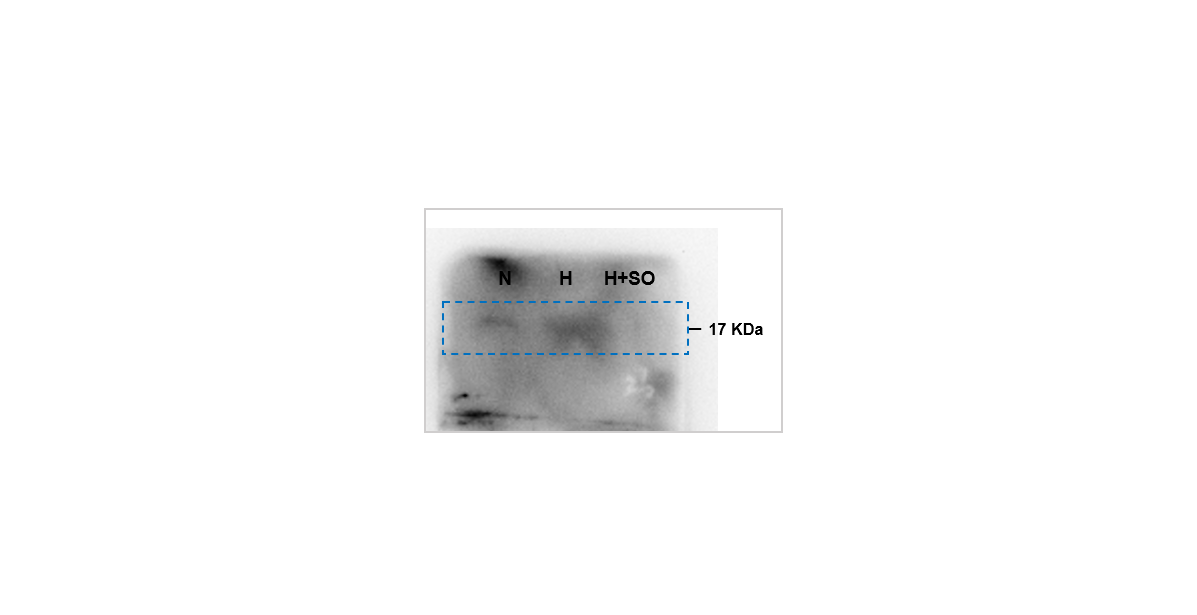

Supplement: Supplementary file 4 — Source data Fig. 2 [file 44319_2026_829_MOESM4_ESM.zip › Figure 2/C/H3K23la.png]

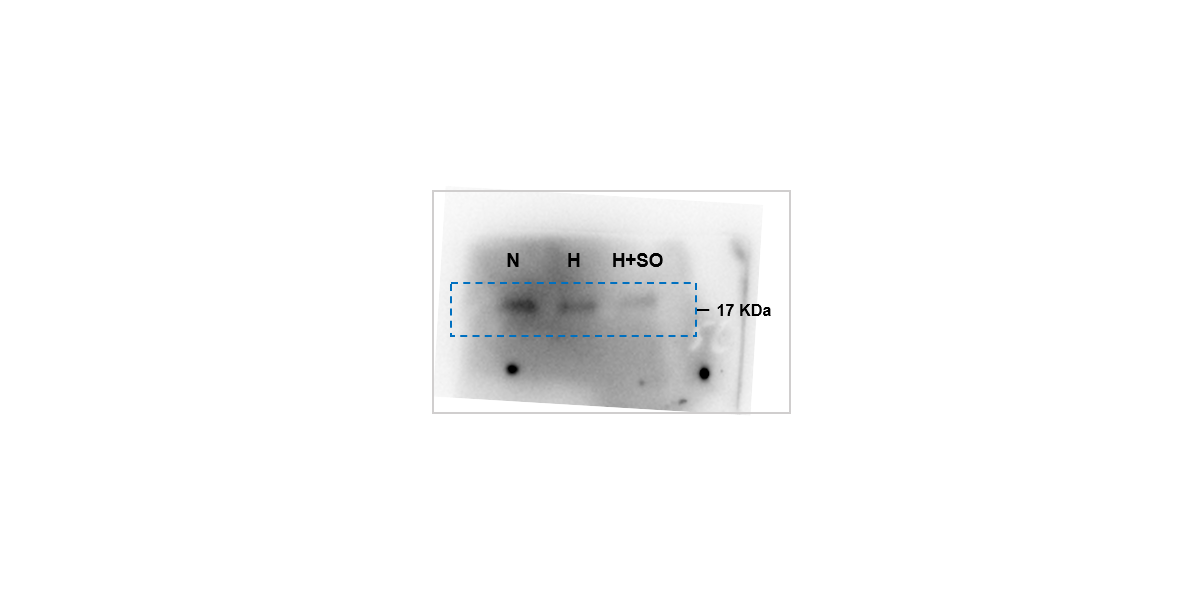

Supplement: Supplementary file 4 — Source data Fig. 2 [file 44319_2026_829_MOESM4_ESM.zip › Figure 2/C/H3K56la.png]

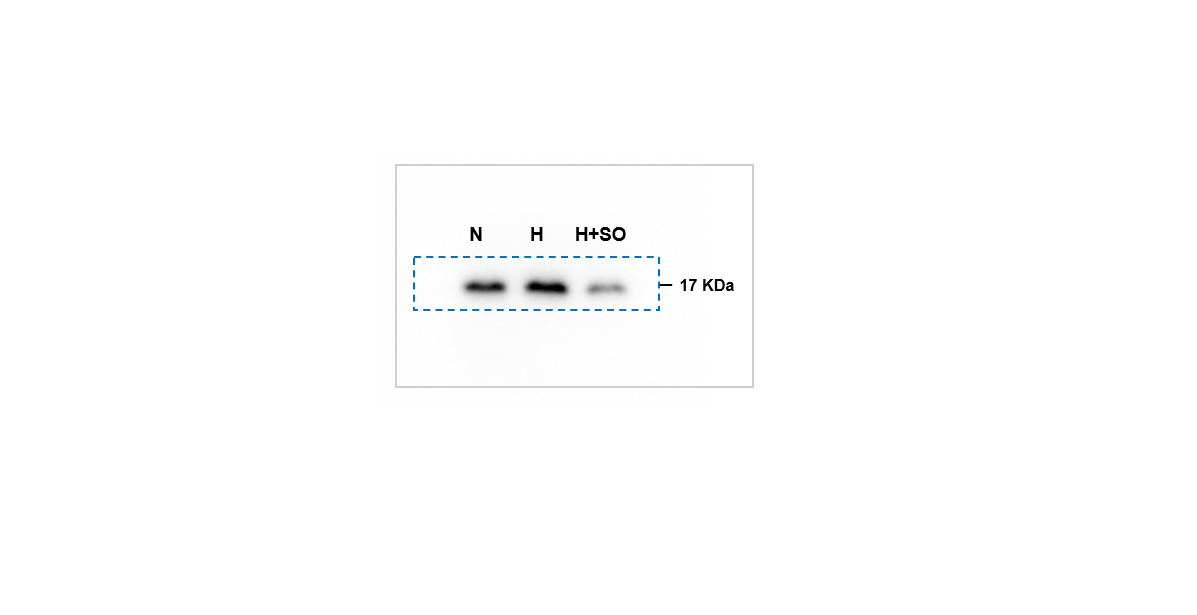

Supplement: Supplementary file 4 — Source data Fig. 2 [file 44319_2026_829_MOESM4_ESM.zip › Figure 2/C/H3K9la.png]

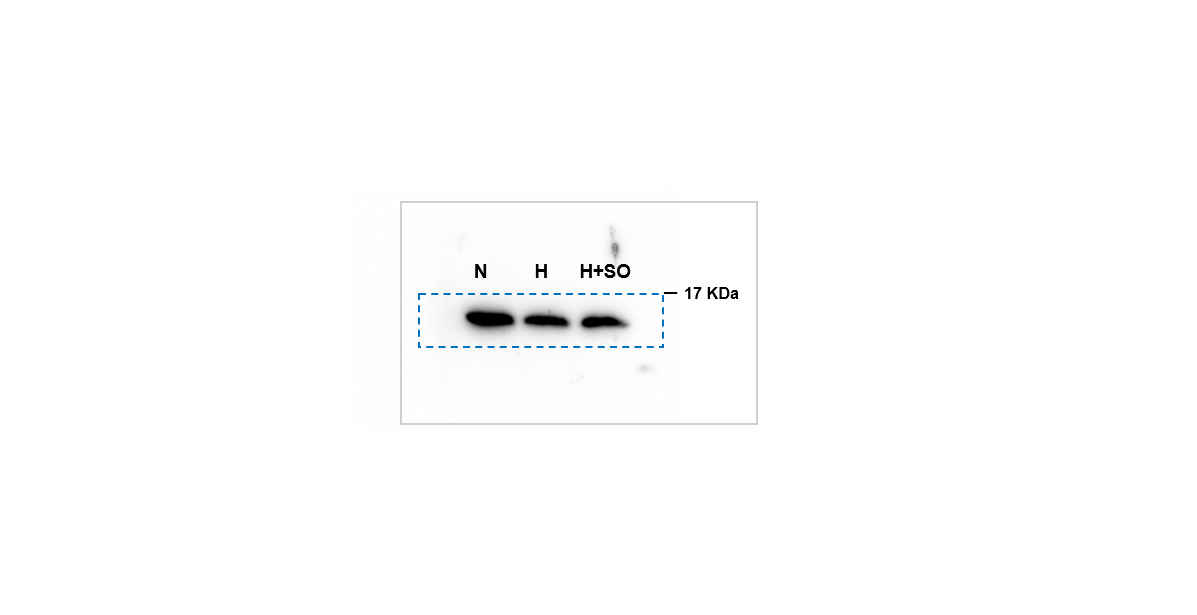

Supplement: Supplementary file 4 — Source data Fig. 2 [file 44319_2026_829_MOESM4_ESM.zip › Figure 2/C/H4.png]

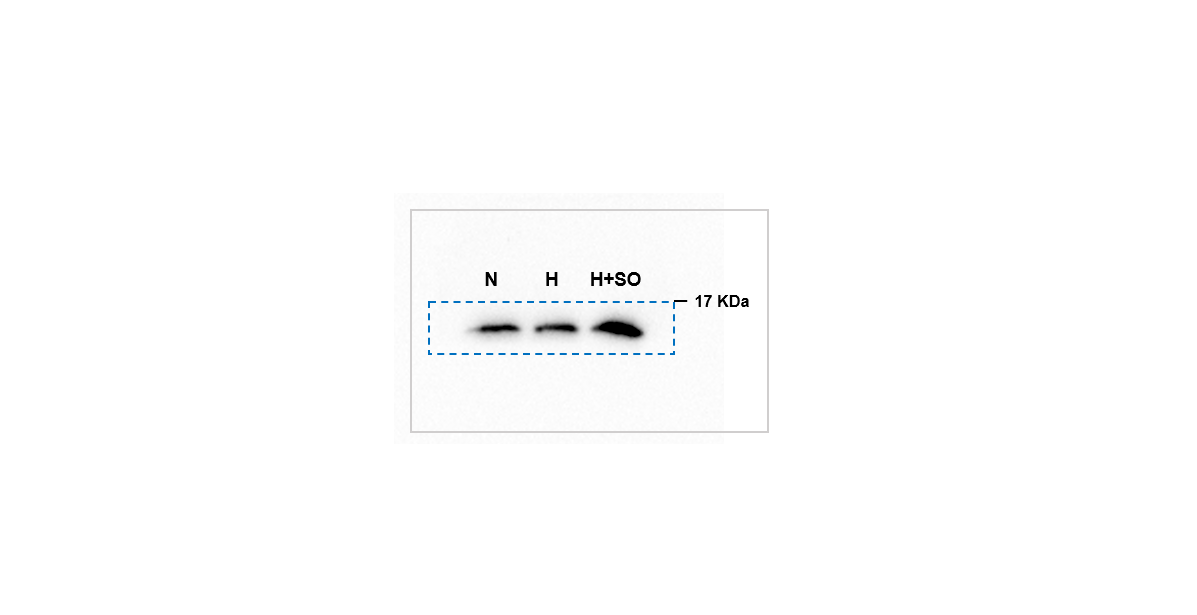

Supplement: Supplementary file 4 — Source data Fig. 2 [file 44319_2026_829_MOESM4_ESM.zip › Figure 2/C/H4K12la.png]

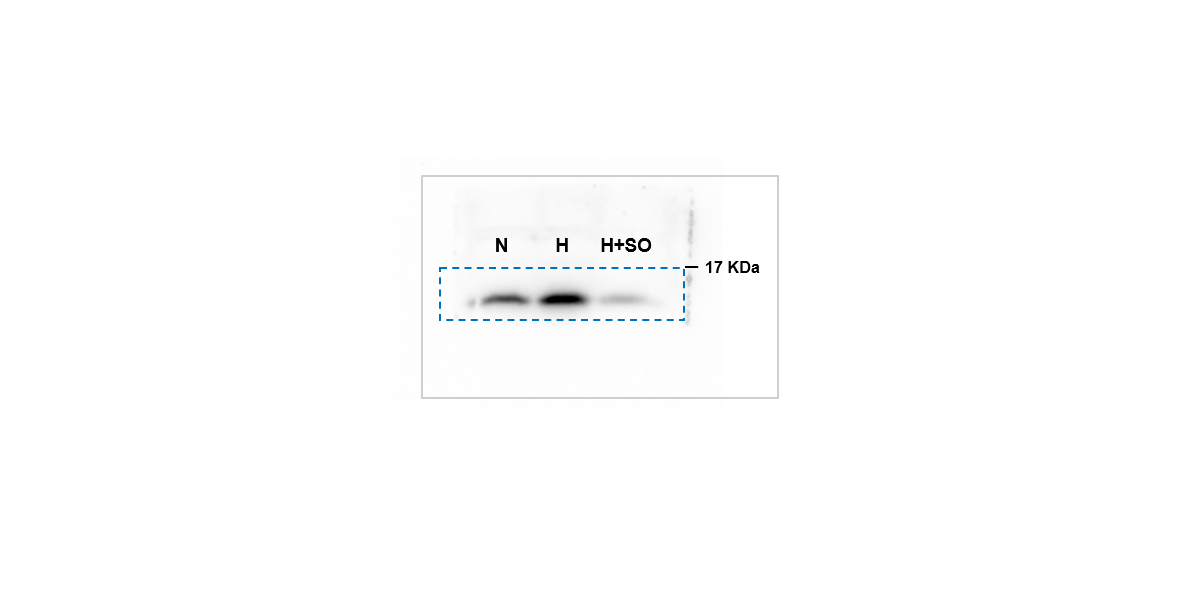

Supplement: Supplementary file 4 — Source data Fig. 2 [file 44319_2026_829_MOESM4_ESM.zip › Figure 2/C/H4K5la.png]

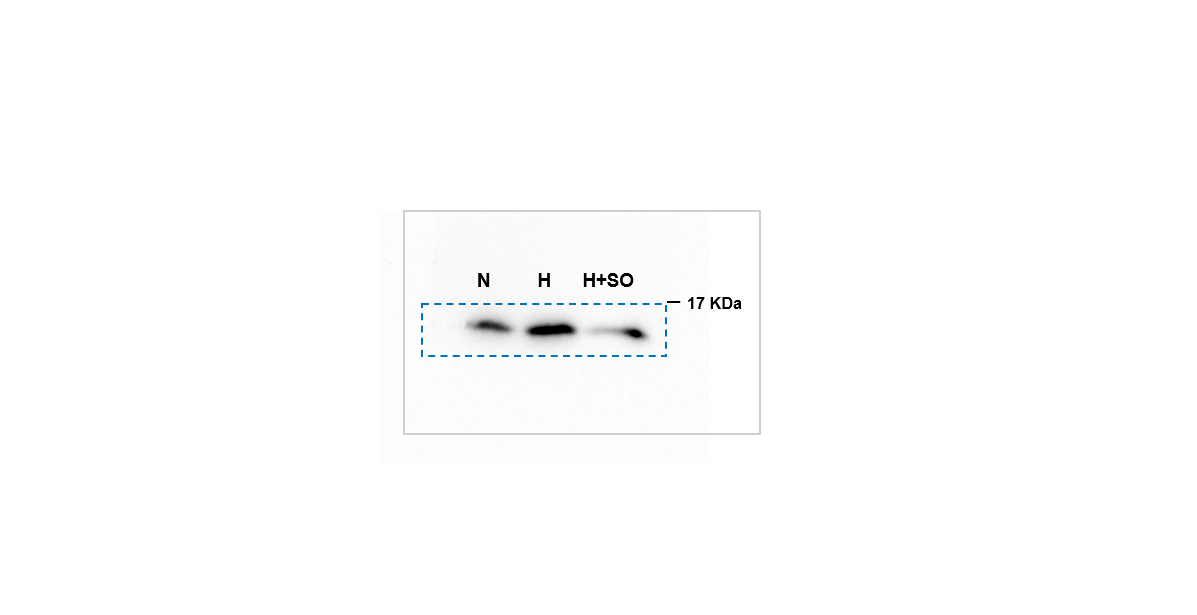

Supplement: Supplementary file 4 — Source data Fig. 2 [file 44319_2026_829_MOESM4_ESM.zip › Figure 2/C/H4K8la.png]

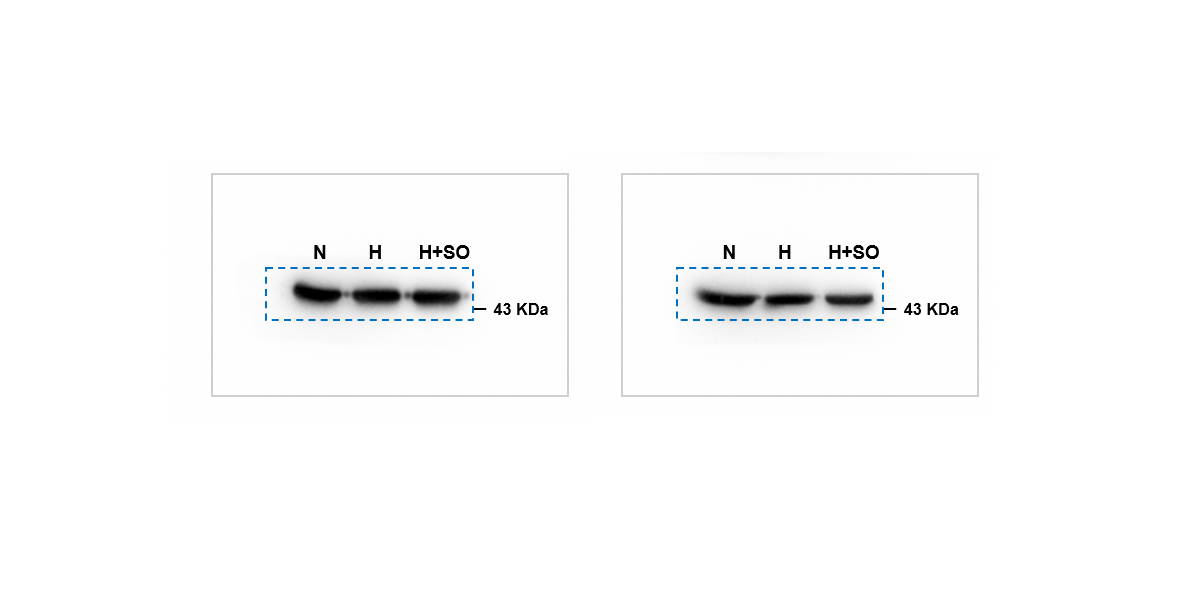

Supplement: Supplementary file 4 — Source data Fig. 2 [file 44319_2026_829_MOESM4_ESM.zip › Figure 2/G/ACTIN.png]

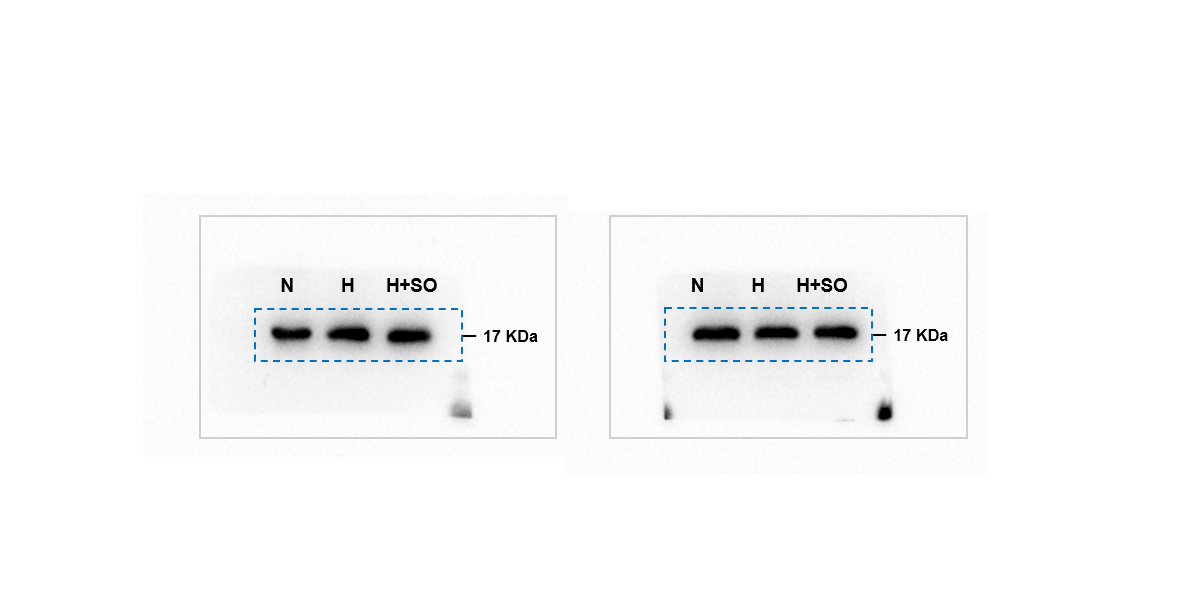

Supplement: Supplementary file 4 — Source data Fig. 2 [file 44319_2026_829_MOESM4_ESM.zip › Figure 2/G/H3.png]

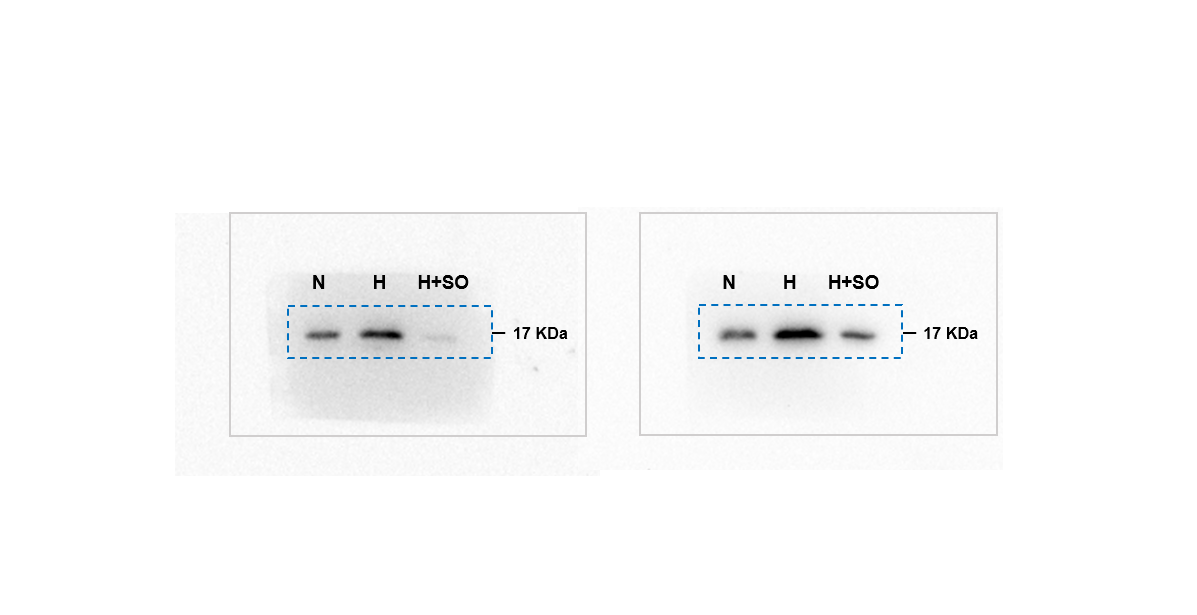

Supplement: Supplementary file 4 — Source data Fig. 2 [file 44319_2026_829_MOESM4_ESM.zip › Figure 2/G/H3K18la.png]

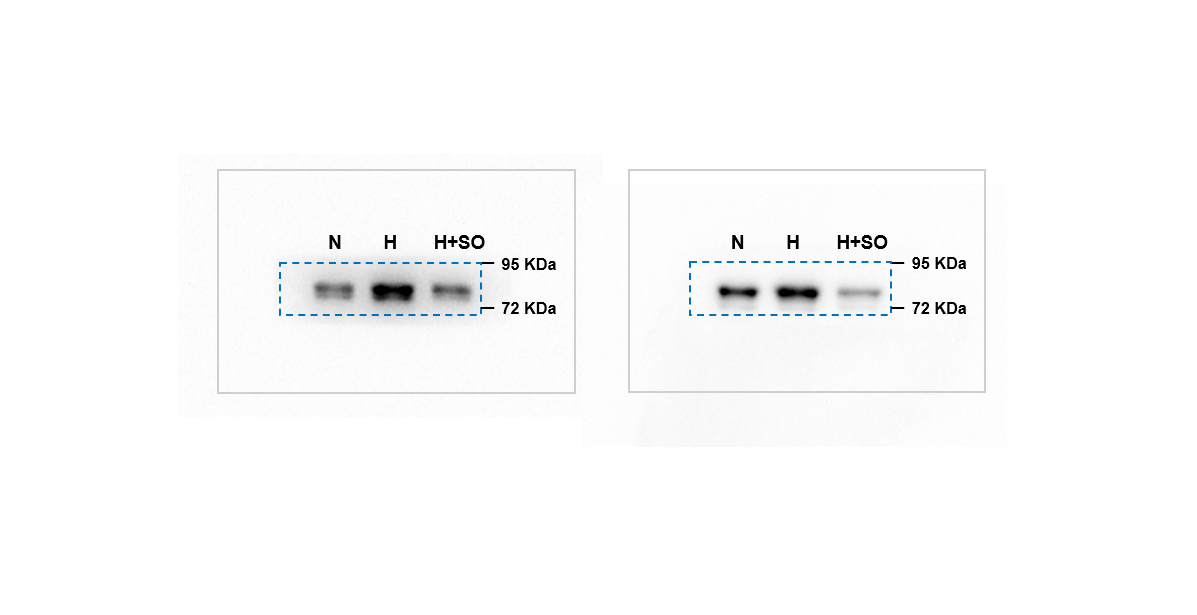

Supplement: Supplementary file 4 — Source data Fig. 2 [file 44319_2026_829_MOESM4_ESM.zip › Figure 2/G/SCARB1.png]

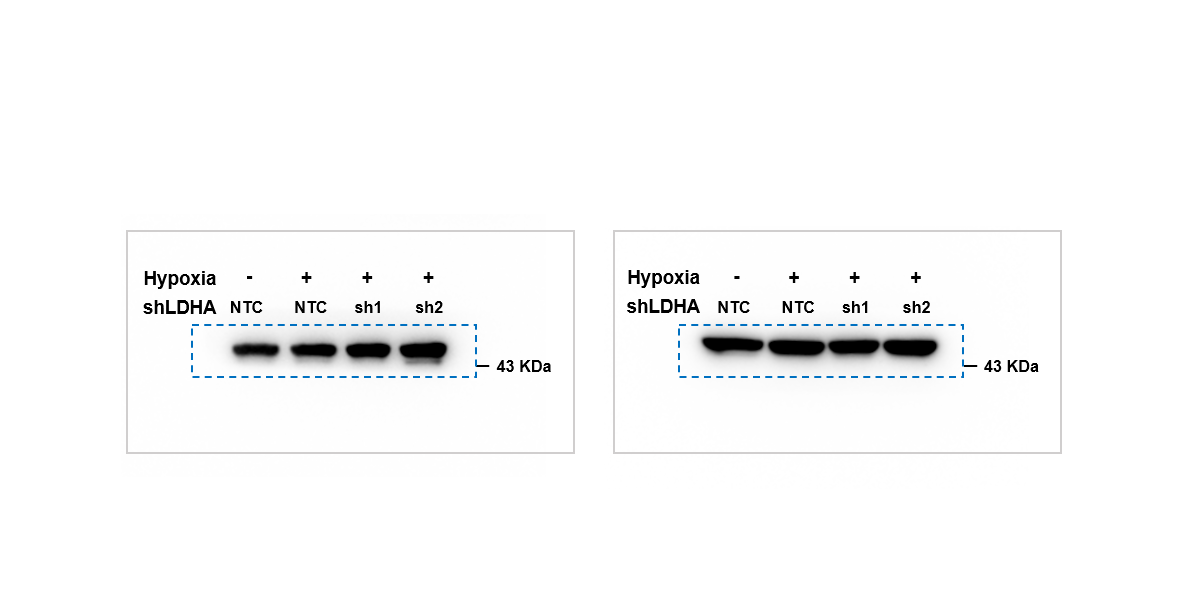

Supplement: Supplementary file 4 — Source data Fig. 2 [file 44319_2026_829_MOESM4_ESM.zip › Figure 2/H/ACTIN.png]

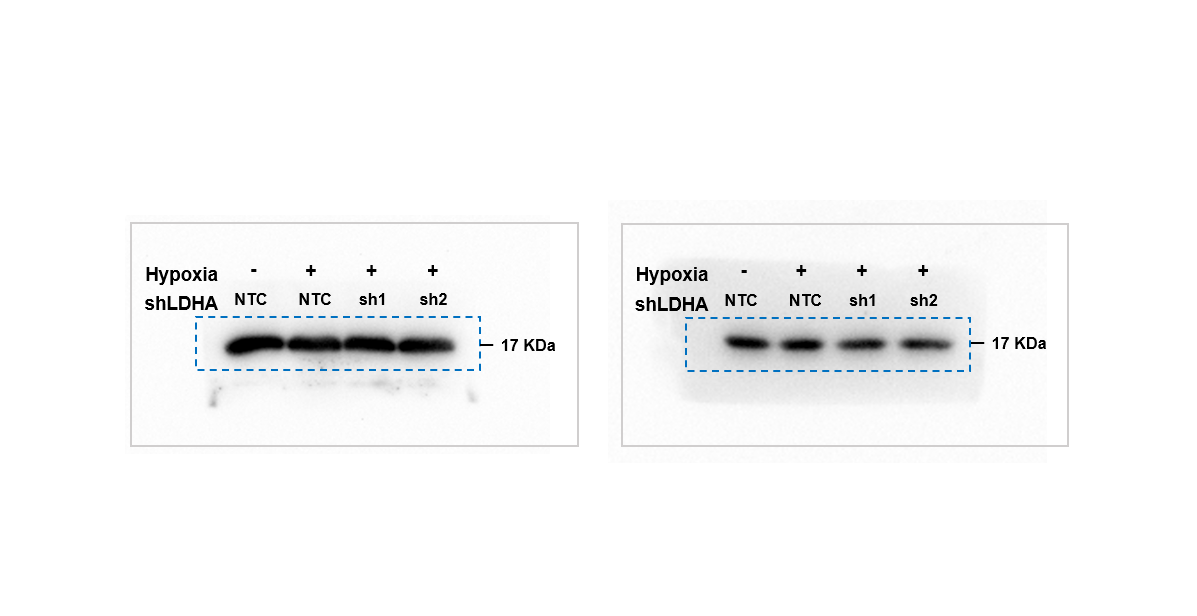

Supplement: Supplementary file 4 — Source data Fig. 2 [file 44319_2026_829_MOESM4_ESM.zip › Figure 2/H/h3.png]

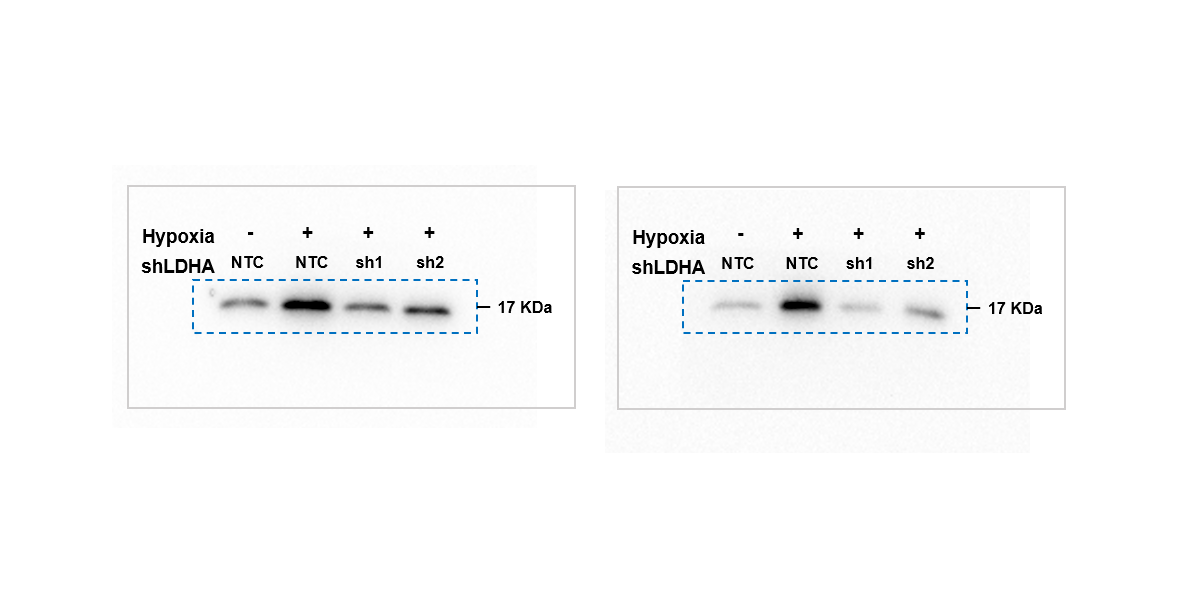

Supplement: Supplementary file 4 — Source data Fig. 2 [file 44319_2026_829_MOESM4_ESM.zip › Figure 2/H/H3K18la.png]

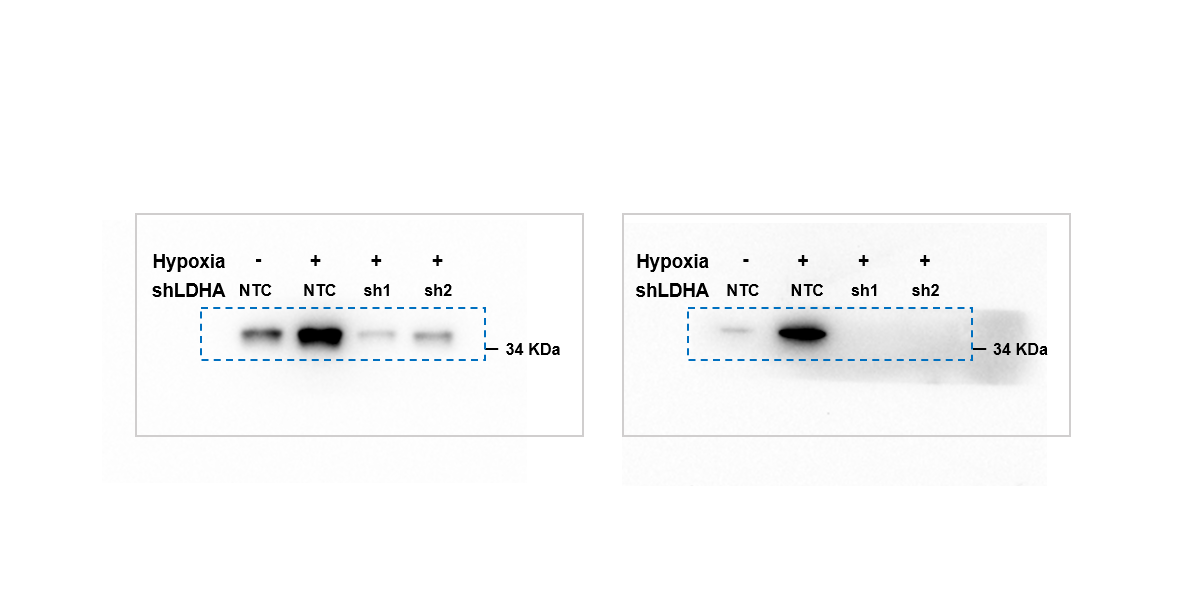

Supplement: Supplementary file 4 — Source data Fig. 2 [file 44319_2026_829_MOESM4_ESM.zip › Figure 2/H/LDHA.png]

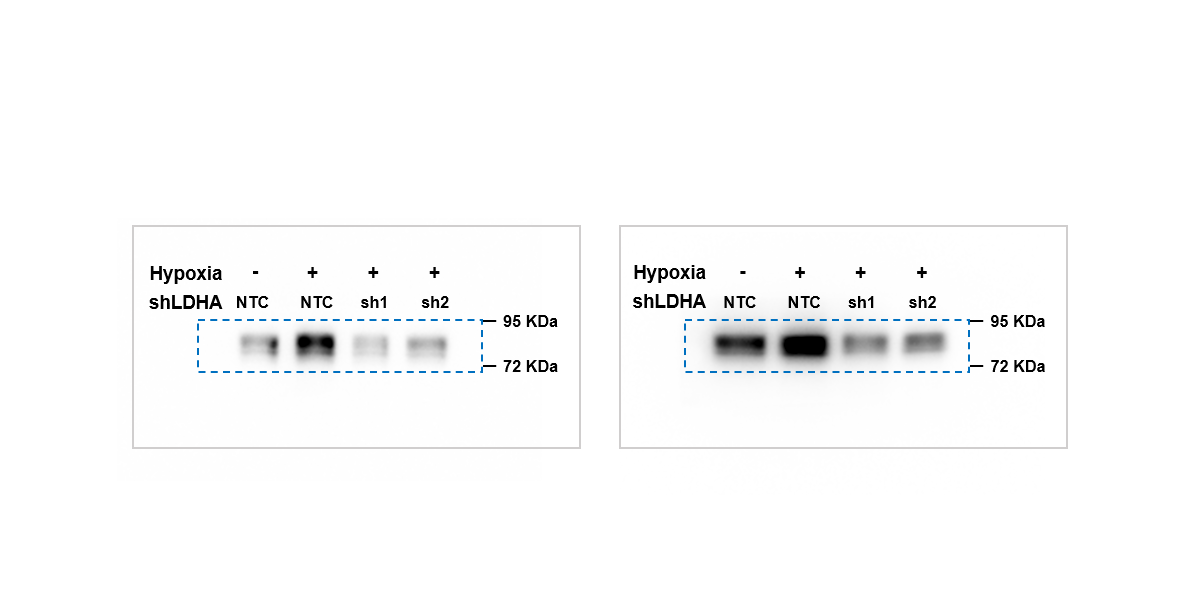

Supplement: Supplementary file 4 — Source data Fig. 2 [file 44319_2026_829_MOESM4_ESM.zip › Figure 2/H/SCARB1.png]

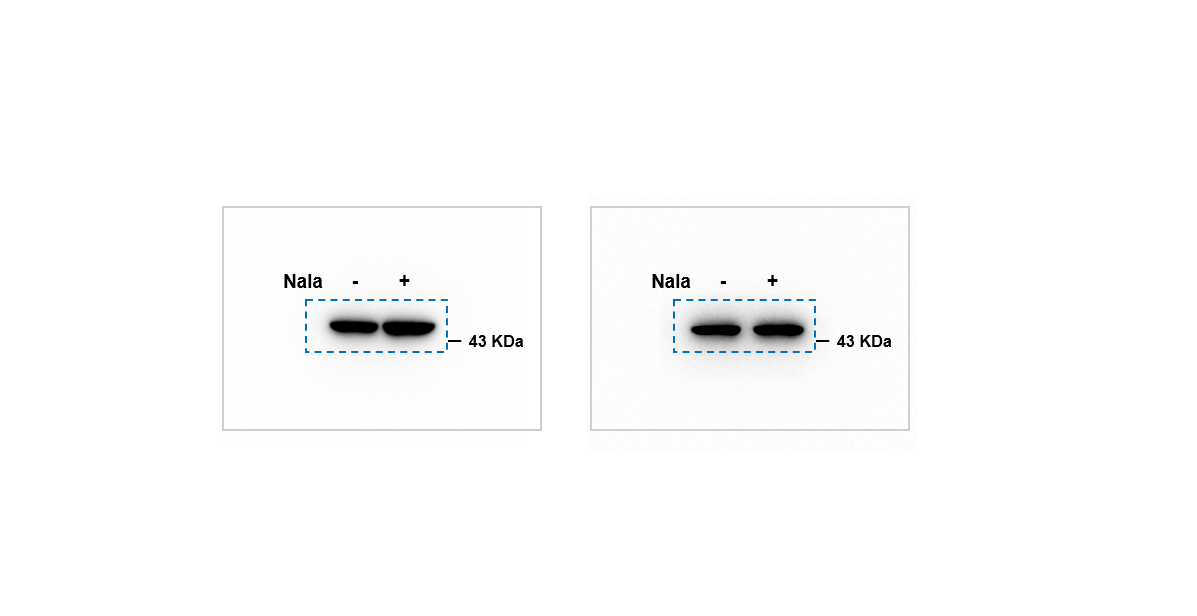

Supplement: Supplementary file 4 — Source data Fig. 2 [file 44319_2026_829_MOESM4_ESM.zip › Figure 2/I/ACTIN.png]

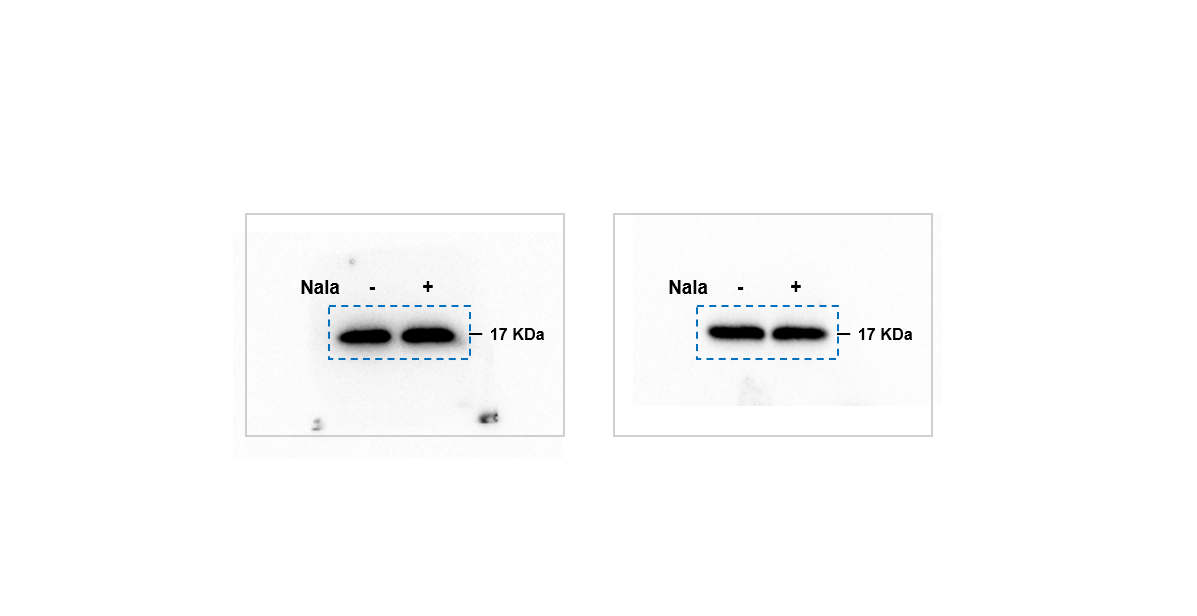

Supplement: Supplementary file 4 — Source data Fig. 2 [file 44319_2026_829_MOESM4_ESM.zip › Figure 2/I/h3.png]

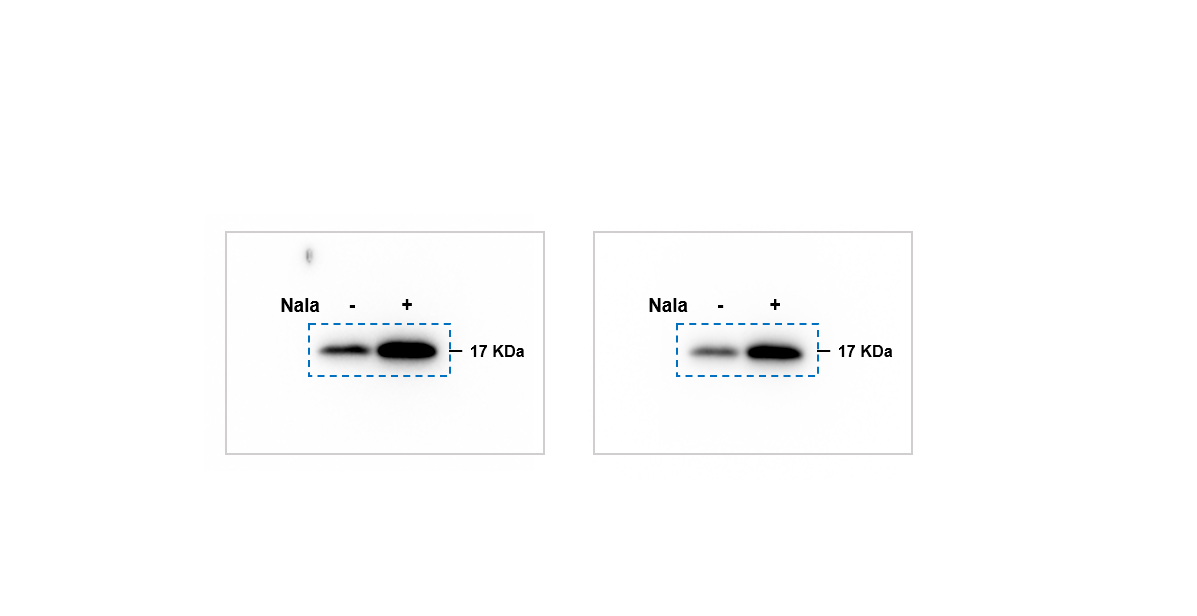

Supplement: Supplementary file 4 — Source data Fig. 2 [file 44319_2026_829_MOESM4_ESM.zip › Figure 2/I/H3K18la.png]

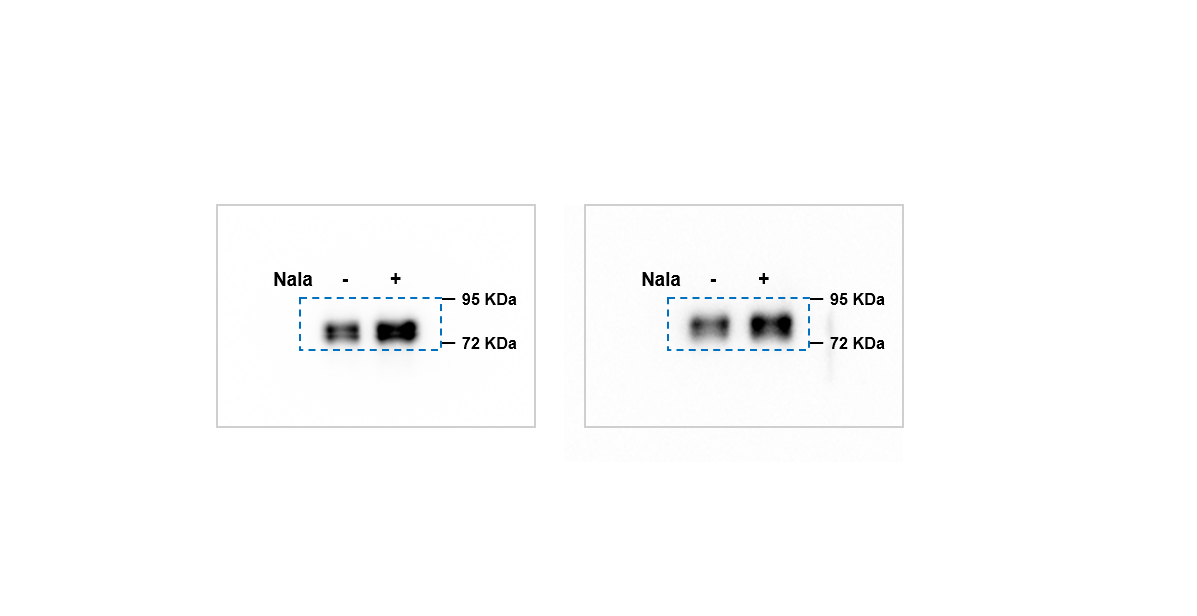

Supplement: Supplementary file 4 — Source data Fig. 2 [file 44319_2026_829_MOESM4_ESM.zip › Figure 2/I/SCARB1.png]

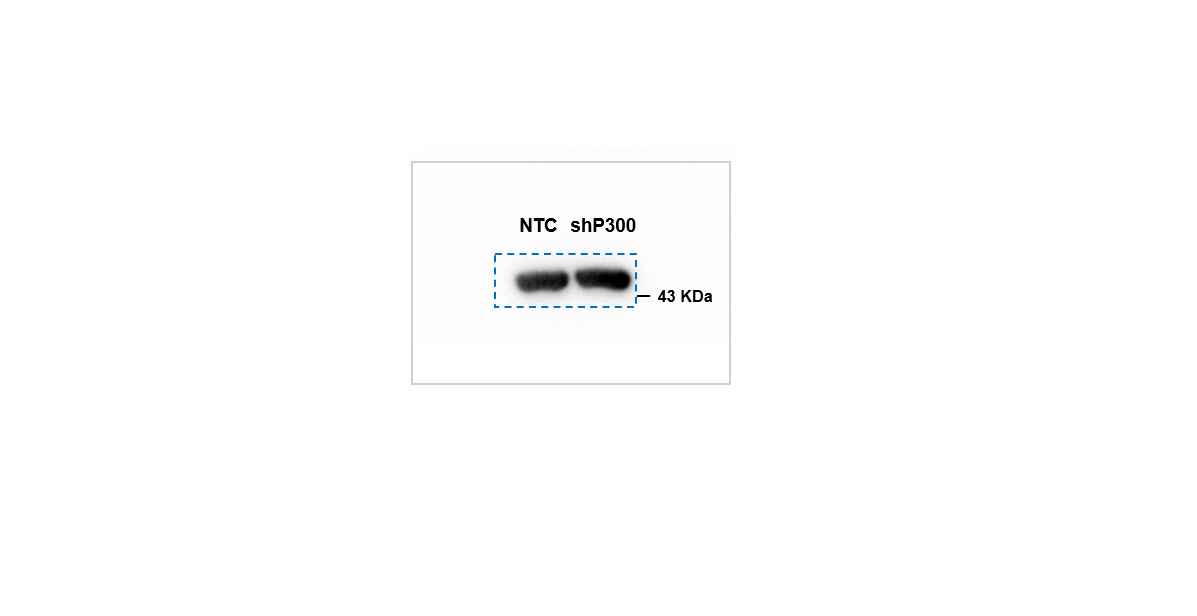

Supplement: Supplementary file 4 — Source data Fig. 2 [file 44319_2026_829_MOESM4_ESM.zip › Figure 2/J/ACTIN.png]

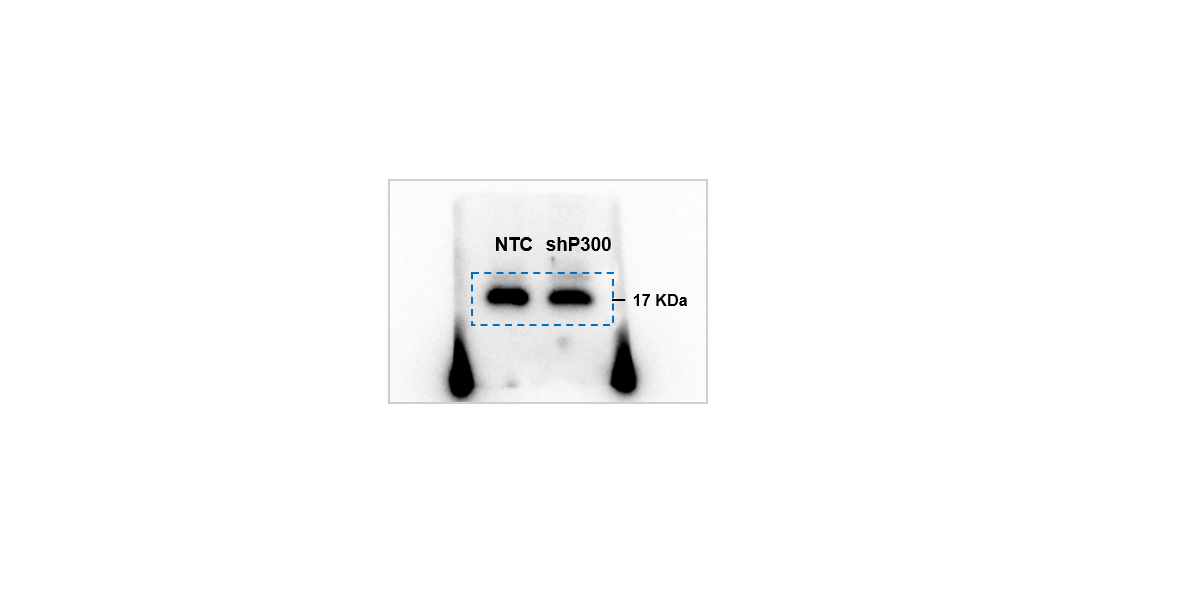

Supplement: Supplementary file 4 — Source data Fig. 2 [file 44319_2026_829_MOESM4_ESM.zip › Figure 2/J/H3.png]

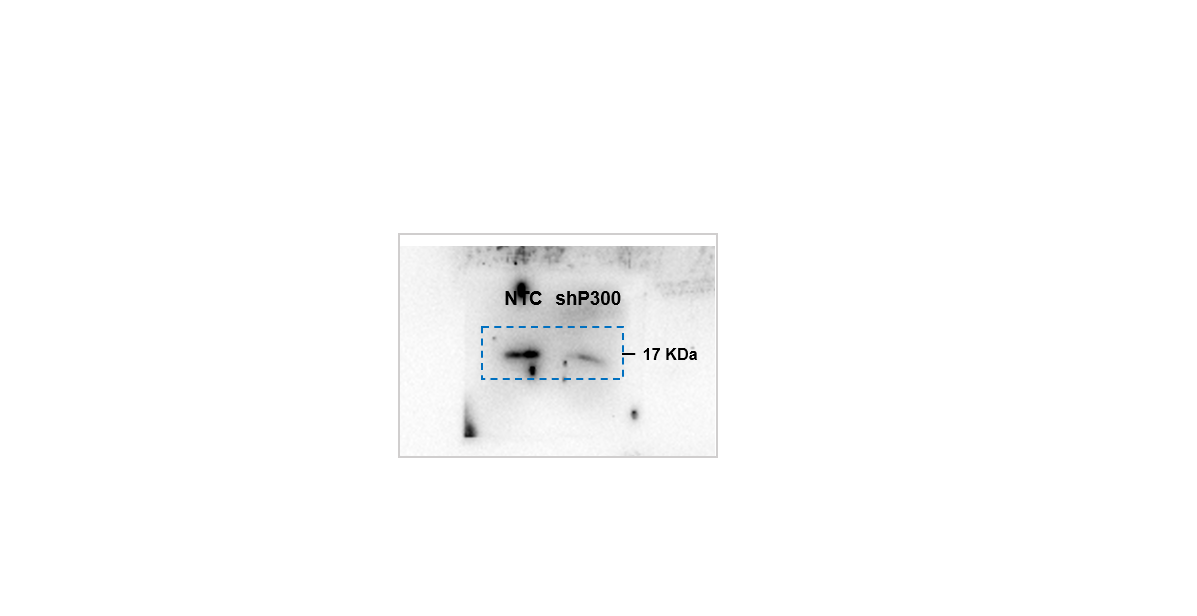

Supplement: Supplementary file 4 — Source data Fig. 2 [file 44319_2026_829_MOESM4_ESM.zip › Figure 2/J/H3K18la.png]

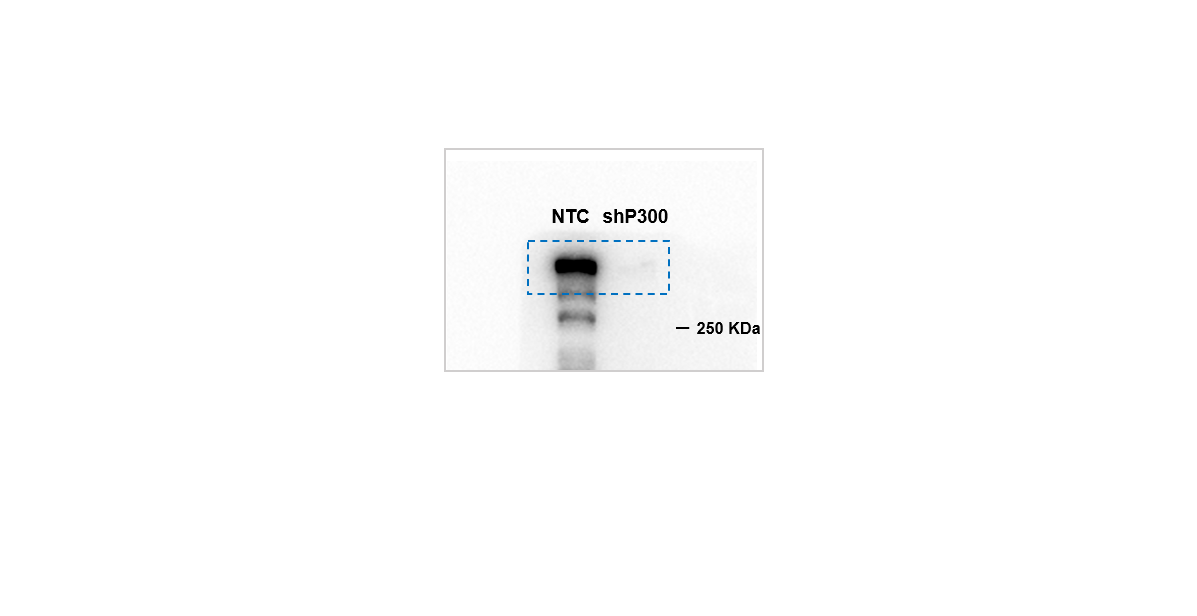

Supplement: Supplementary file 4 — Source data Fig. 2 [file 44319_2026_829_MOESM4_ESM.zip › Figure 2/J/P300.png]

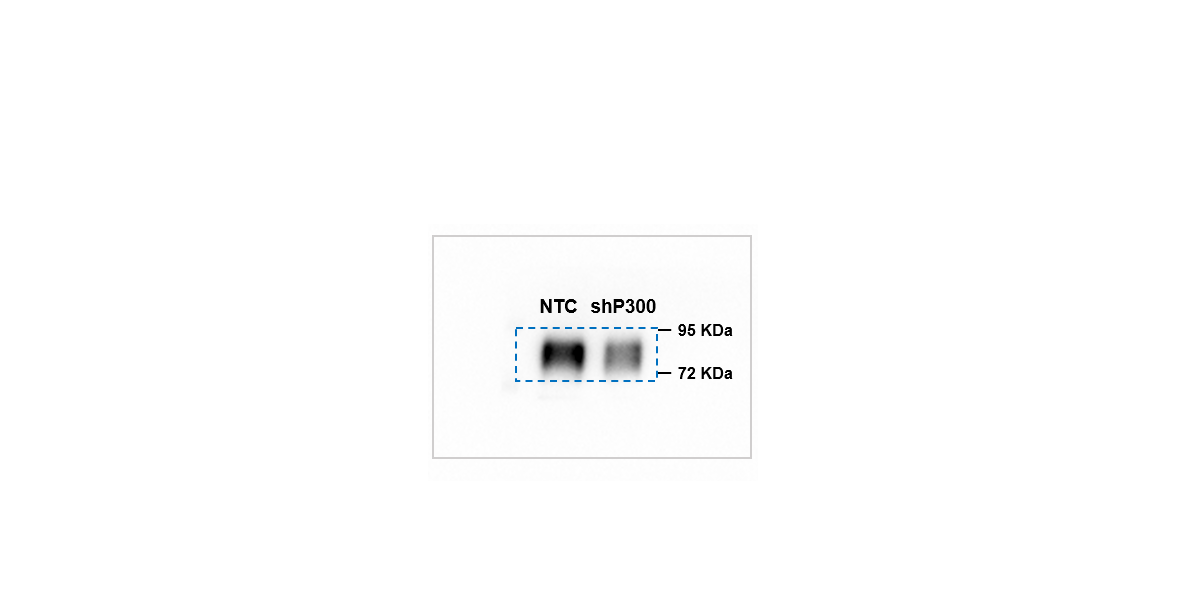

Supplement: Supplementary file 4 — Source data Fig. 2 [file 44319_2026_829_MOESM4_ESM.zip › Figure 2/J/SCARB1.png]

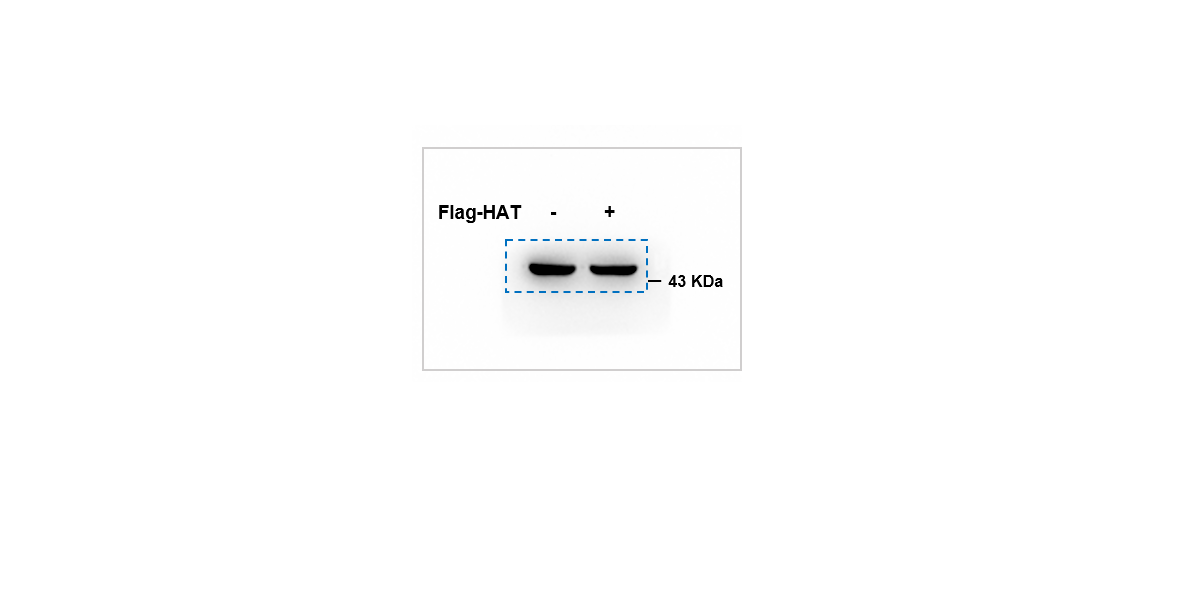

Supplement: Supplementary file 4 — Source data Fig. 2 [file 44319_2026_829_MOESM4_ESM.zip › Figure 2/K/ACTIN.png]

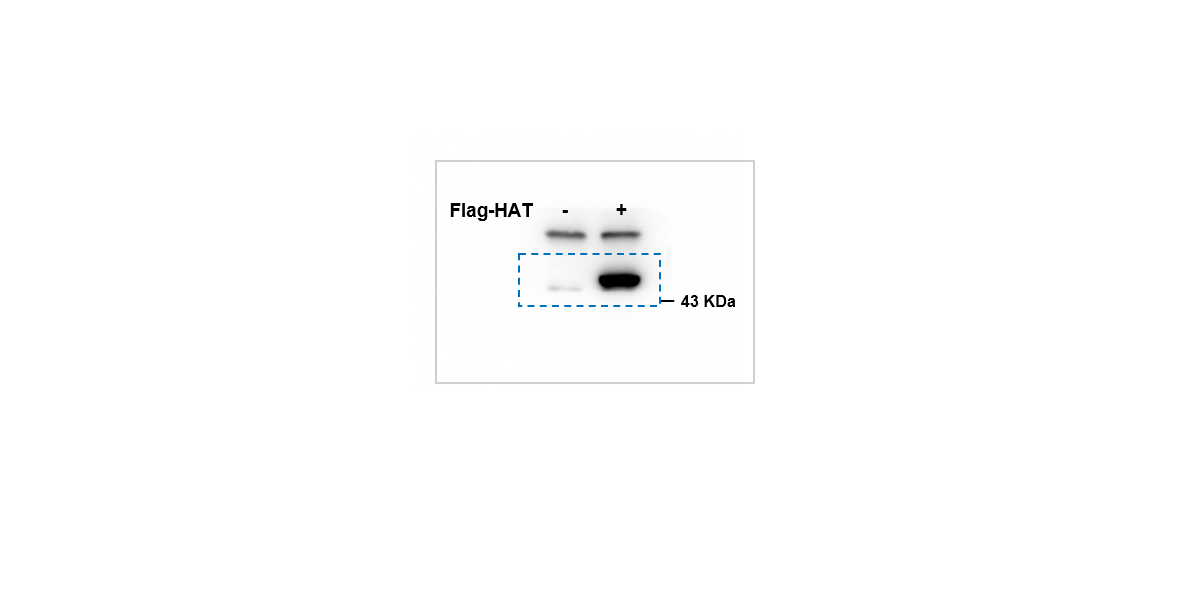

Supplement: Supplementary file 4 — Source data Fig. 2 [file 44319_2026_829_MOESM4_ESM.zip › Figure 2/K/Flag.png]

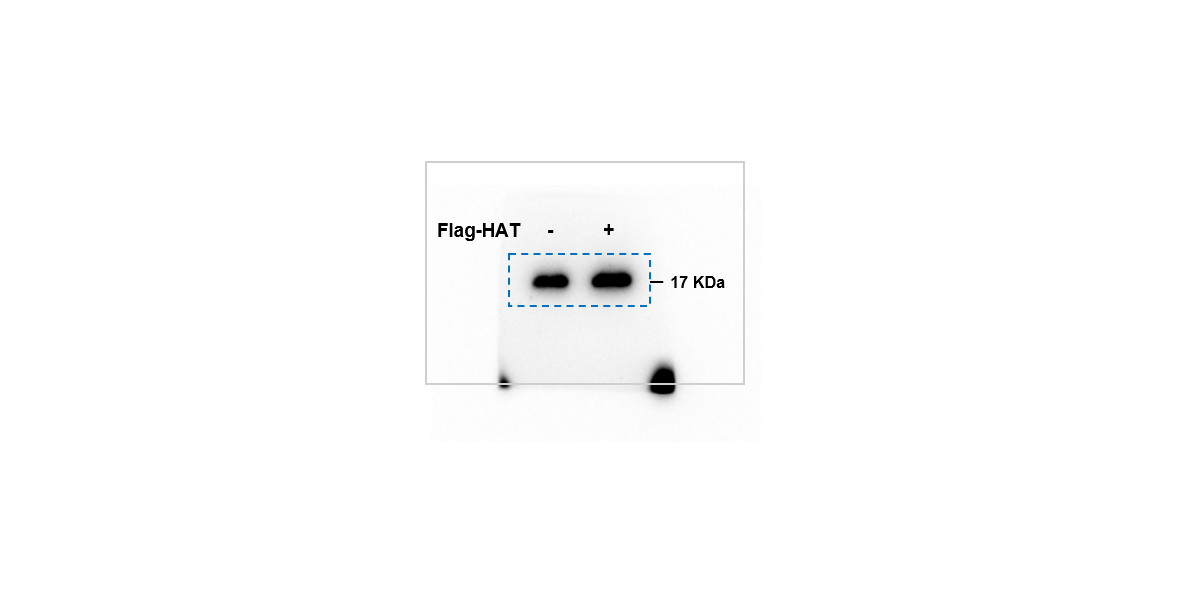

Supplement: Supplementary file 4 — Source data Fig. 2 [file 44319_2026_829_MOESM4_ESM.zip › Figure 2/K/H3.png]

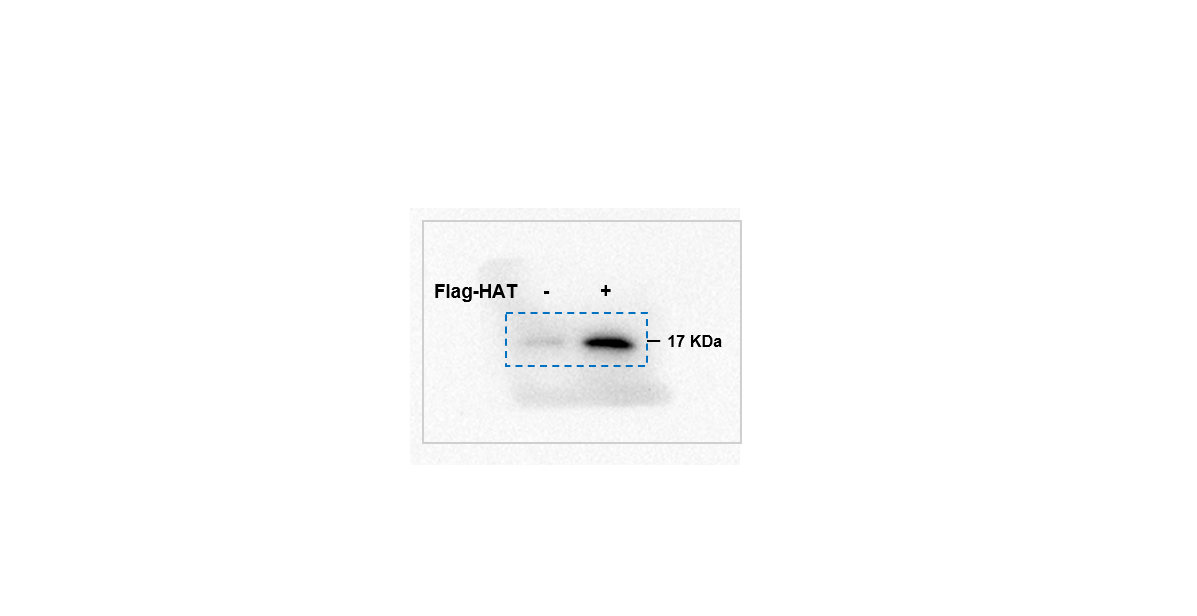

Supplement: Supplementary file 4 — Source data Fig. 2 [file 44319_2026_829_MOESM4_ESM.zip › Figure 2/K/H3K18la.png]

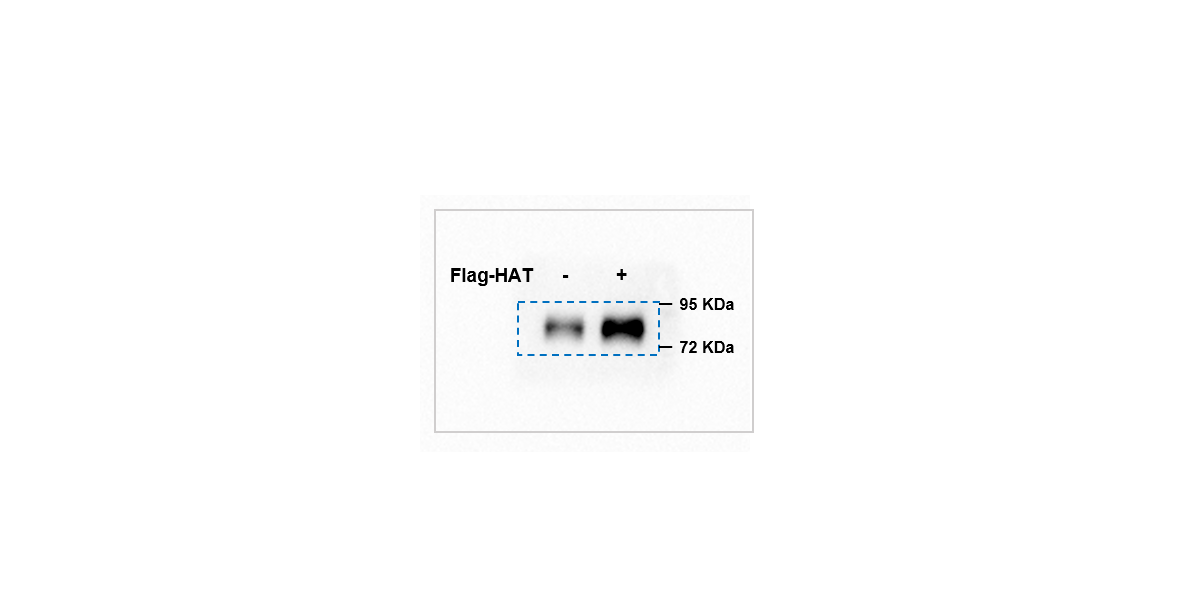

Supplement: Supplementary file 4 — Source data Fig. 2 [file 44319_2026_829_MOESM4_ESM.zip › Figure 2/K/SCARB1.png]

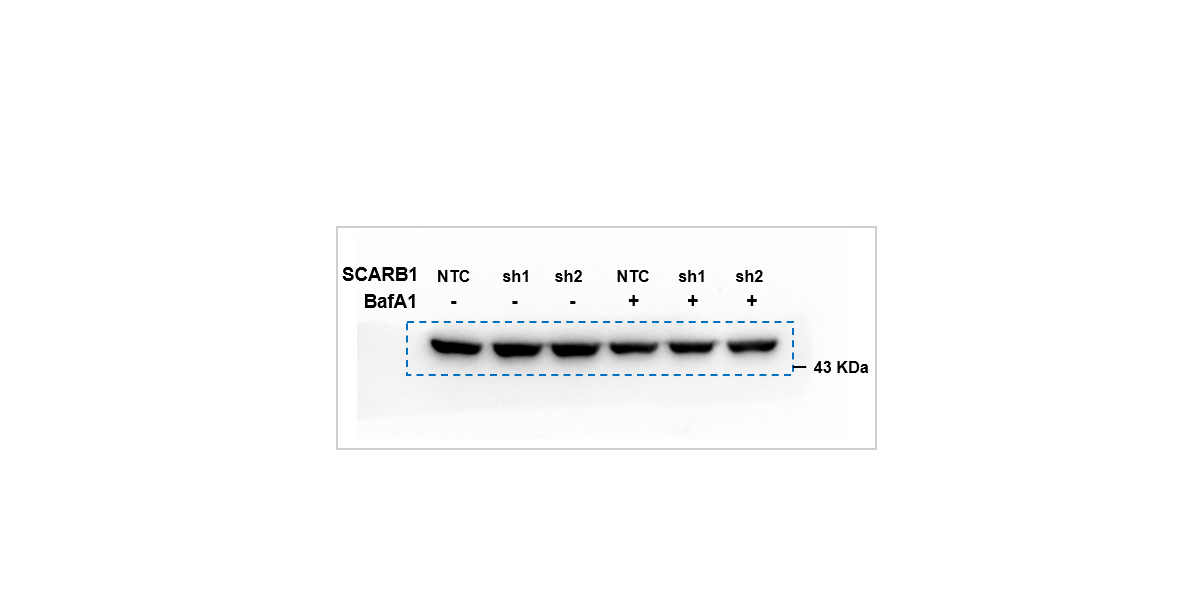

Supplement: Supplementary file 5 — Source data Fig. 3 [file 44319_2026_829_MOESM5_ESM.zip › Figure 3/D/ACTIN.png]

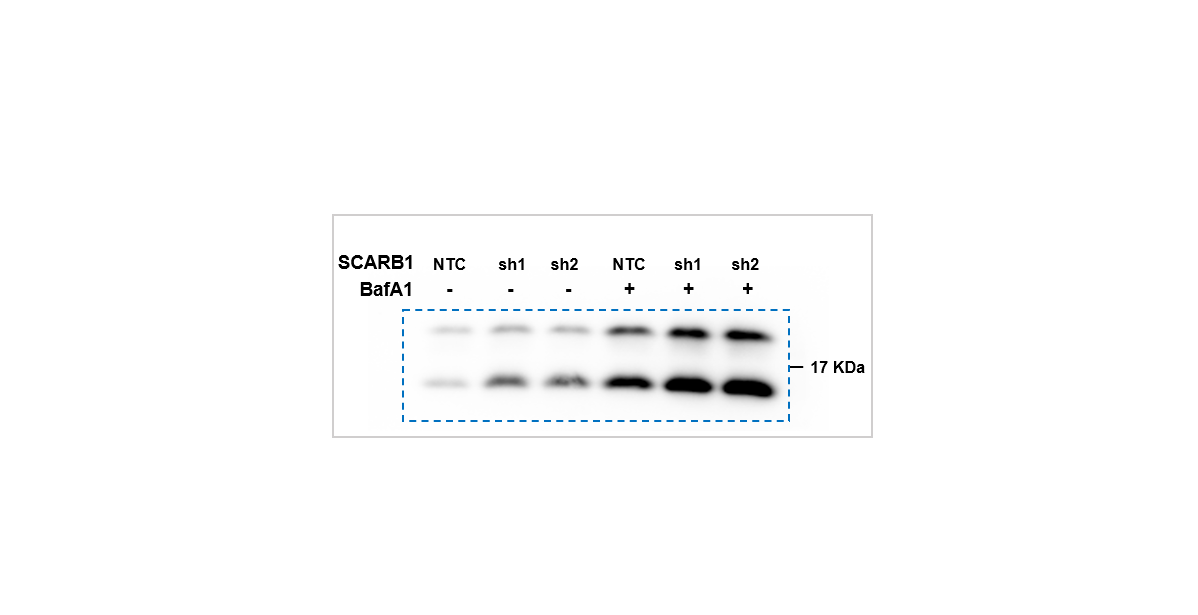

Supplement: Supplementary file 5 — Source data Fig. 3 [file 44319_2026_829_MOESM5_ESM.zip › Figure 3/D/LC3.png]

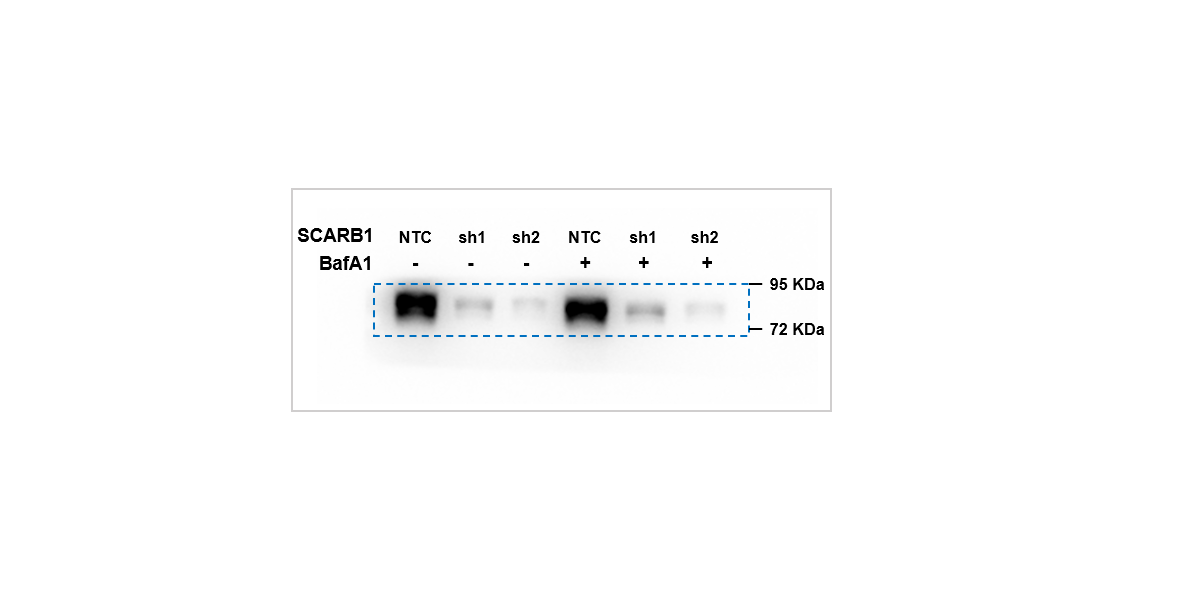

Supplement: Supplementary file 5 — Source data Fig. 3 [file 44319_2026_829_MOESM5_ESM.zip › Figure 3/D/SCARB1.png]

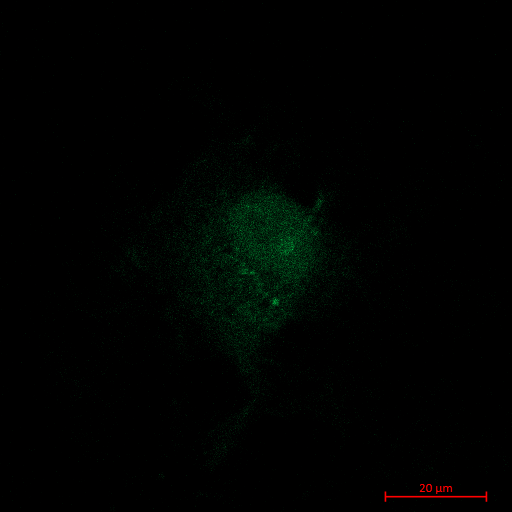

Supplement: Supplementary file 5 — Source data Fig. 3 [file 44319_2026_829_MOESM5_ESM.zip › Figure 3/E/NTC/GFP.tif]

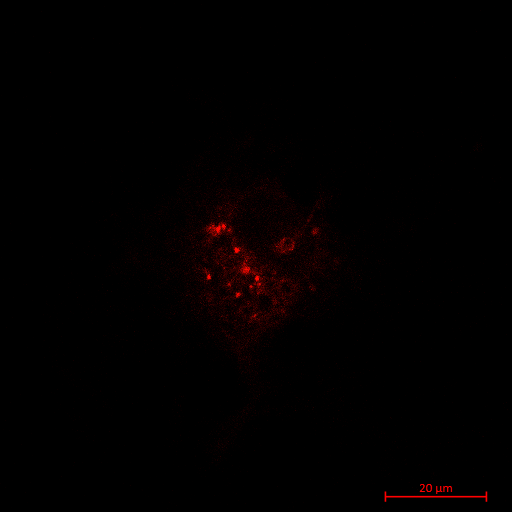

Supplement: Supplementary file 5 — Source data Fig. 3 [file 44319_2026_829_MOESM5_ESM.zip › Figure 3/E/NTC/mCherry.tif]

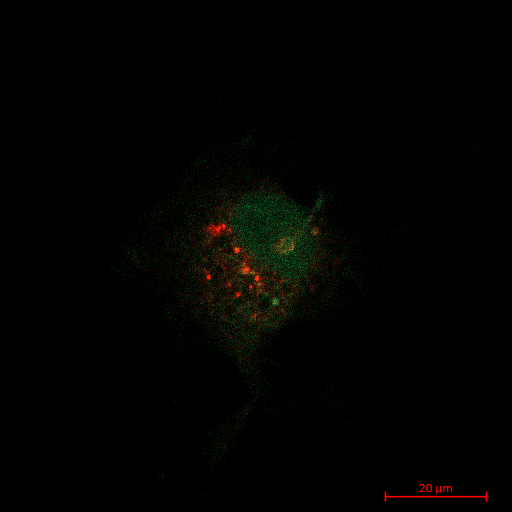

Supplement: Supplementary file 5 — Source data Fig. 3 [file 44319_2026_829_MOESM5_ESM.zip › Figure 3/E/NTC/MERGE.tif]

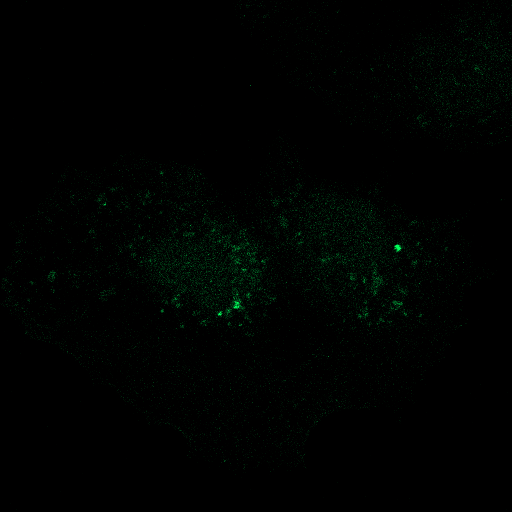

Supplement: Supplementary file 5 — Source data Fig. 3 [file 44319_2026_829_MOESM5_ESM.zip › Figure 3/E/SCARB1-sh1/GFP.tif]

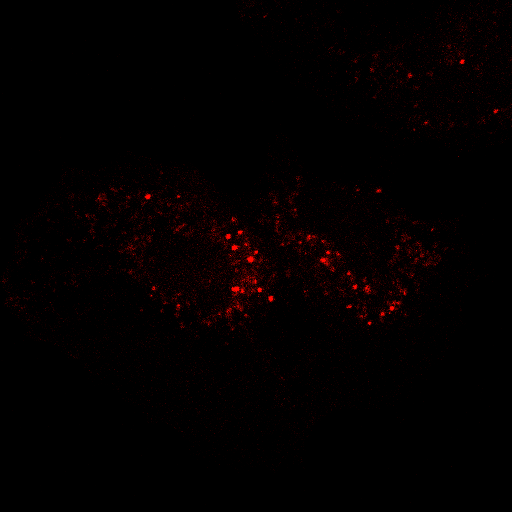

Supplement: Supplementary file 5 — Source data Fig. 3 [file 44319_2026_829_MOESM5_ESM.zip › Figure 3/E/SCARB1-sh1/mCherry.tif]

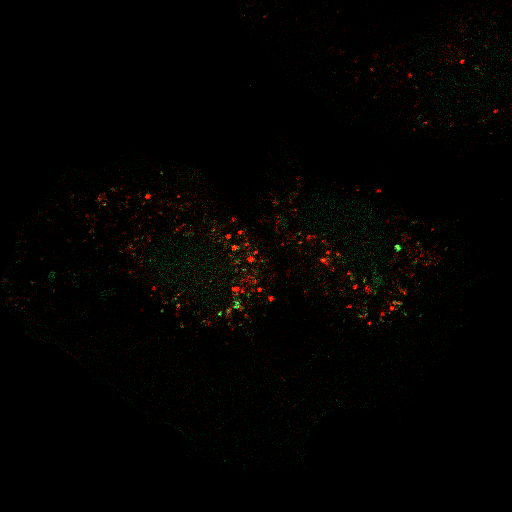

Supplement: Supplementary file 5 — Source data Fig. 3 [file 44319_2026_829_MOESM5_ESM.zip › Figure 3/E/SCARB1-sh1/MERGE.tif]

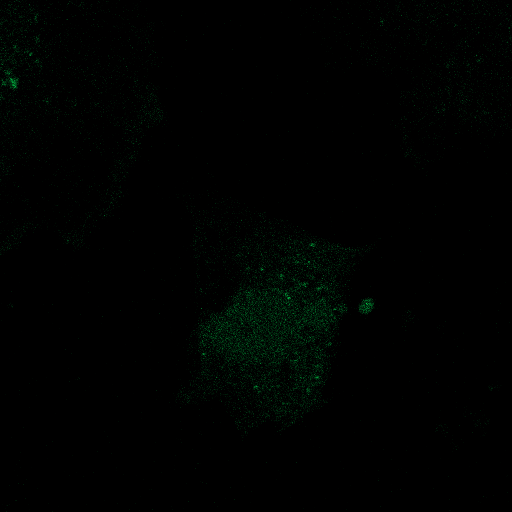

Supplement: Supplementary file 5 — Source data Fig. 3 [file 44319_2026_829_MOESM5_ESM.zip › Figure 3/E/SCARB1-sh2/GFP.tif]

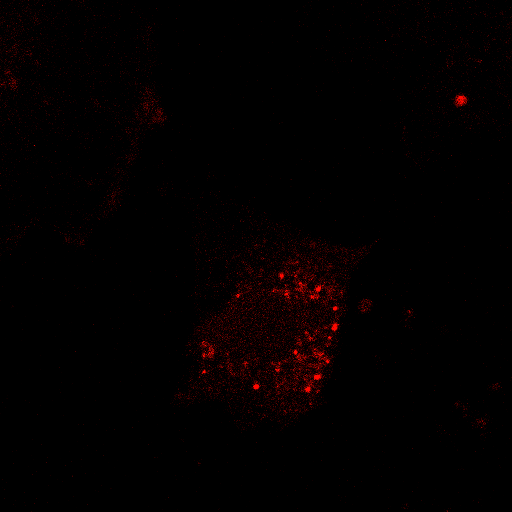

Supplement: Supplementary file 5 — Source data Fig. 3 [file 44319_2026_829_MOESM5_ESM.zip › Figure 3/E/SCARB1-sh2/mCherry.tif]

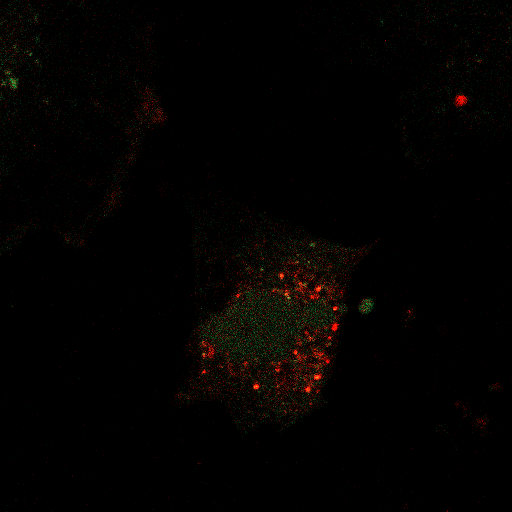

Supplement: Supplementary file 5 — Source data Fig. 3 [file 44319_2026_829_MOESM5_ESM.zip › Figure 3/E/SCARB1-sh2/MERGE.tif]

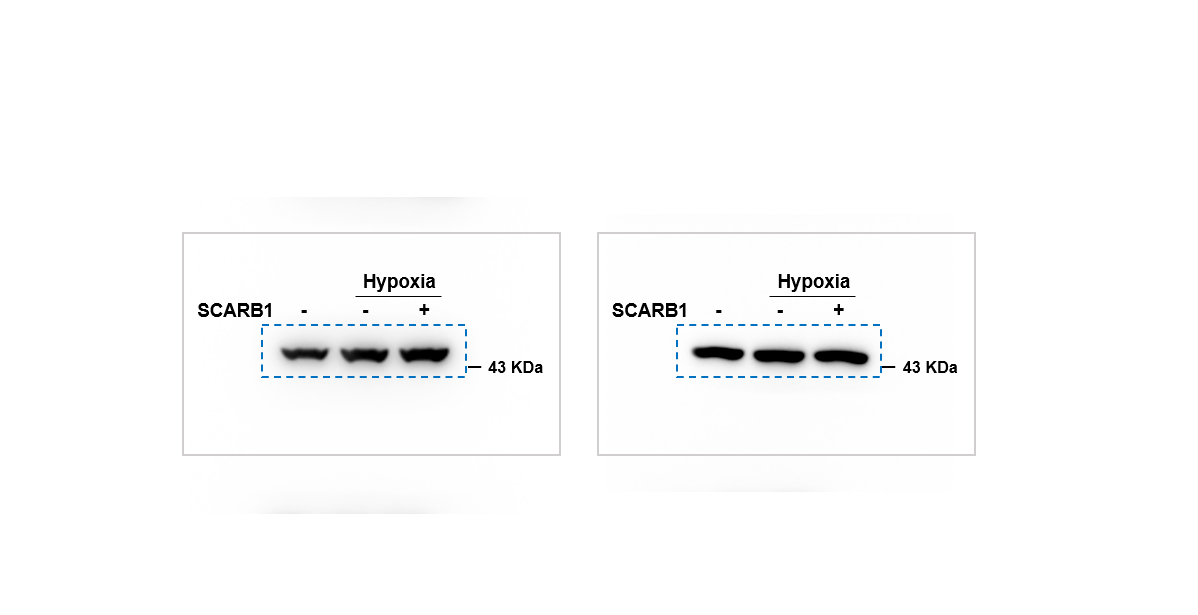

Supplement: Supplementary file 5 — Source data Fig. 3 [file 44319_2026_829_MOESM5_ESM.zip › Figure 3/F/ACTIN.png]

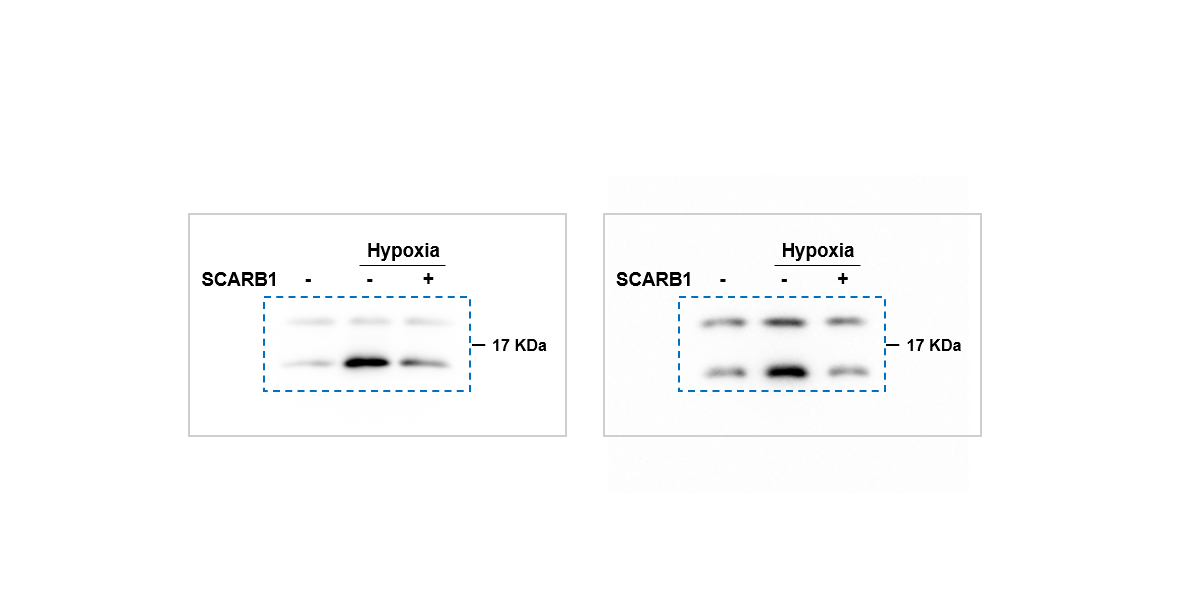

Supplement: Supplementary file 5 — Source data Fig. 3 [file 44319_2026_829_MOESM5_ESM.zip › Figure 3/F/LC3.png]

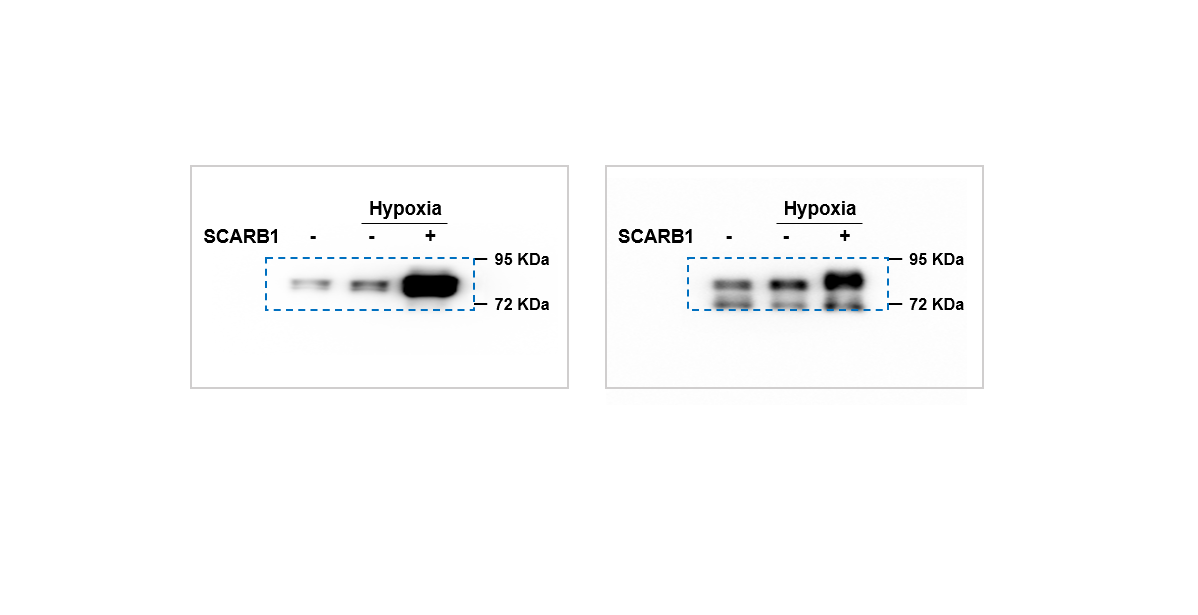

Supplement: Supplementary file 5 — Source data Fig. 3 [file 44319_2026_829_MOESM5_ESM.zip › Figure 3/F/SCARB1.png]

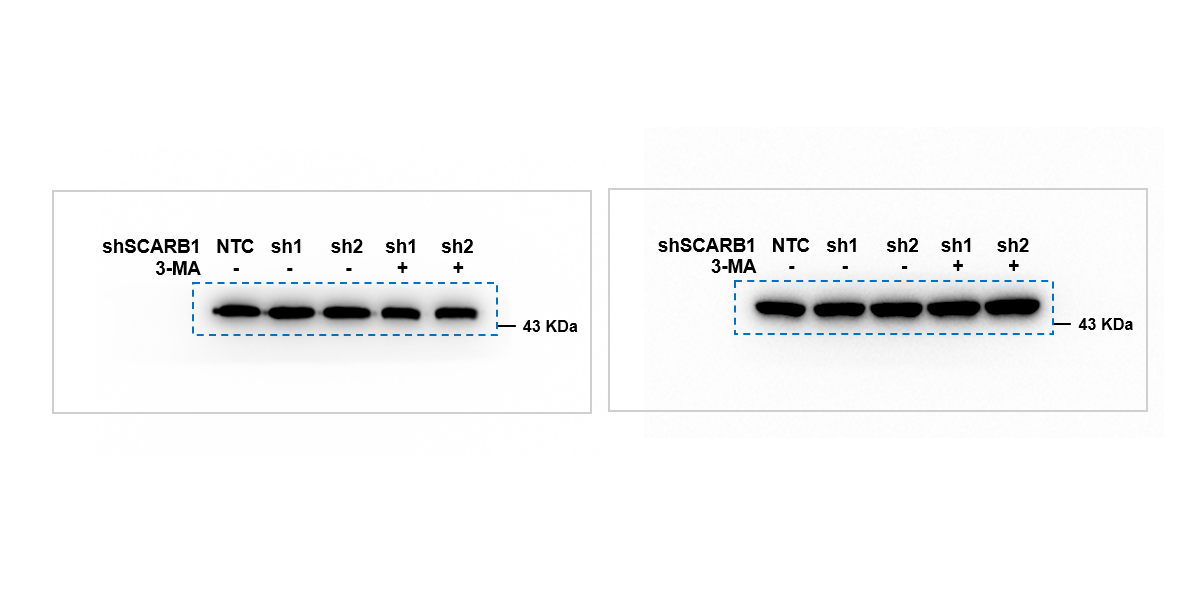

Supplement: Supplementary file 5 — Source data Fig. 3 [file 44319_2026_829_MOESM5_ESM.zip › Figure 3/G/ACTIN.png]

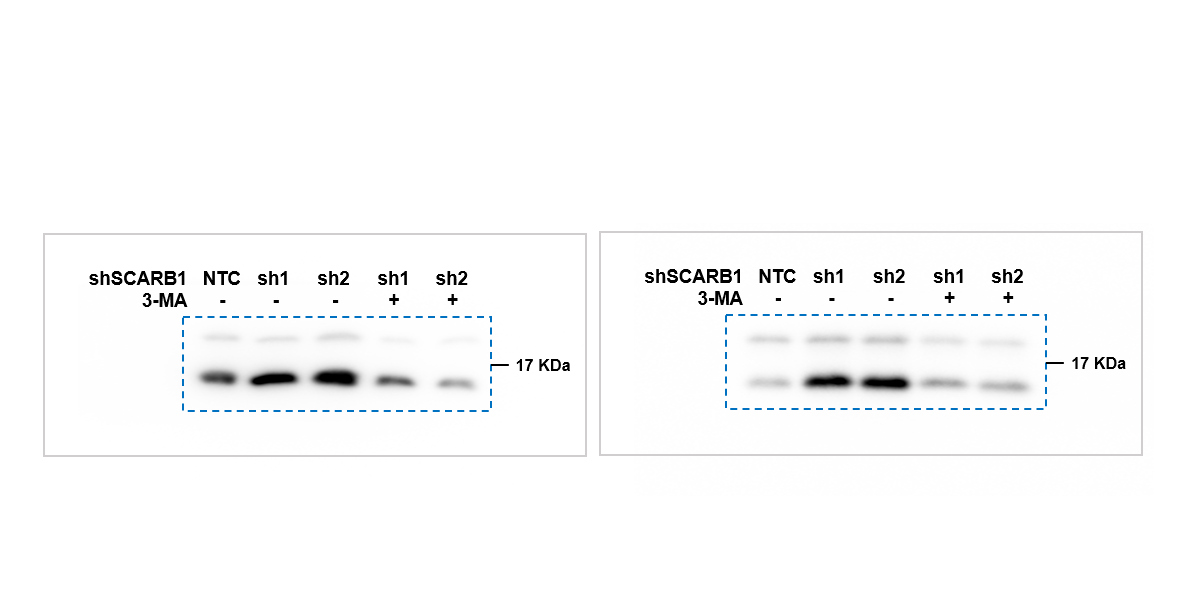

Supplement: Supplementary file 5 — Source data Fig. 3 [file 44319_2026_829_MOESM5_ESM.zip › Figure 3/G/LC3.png]

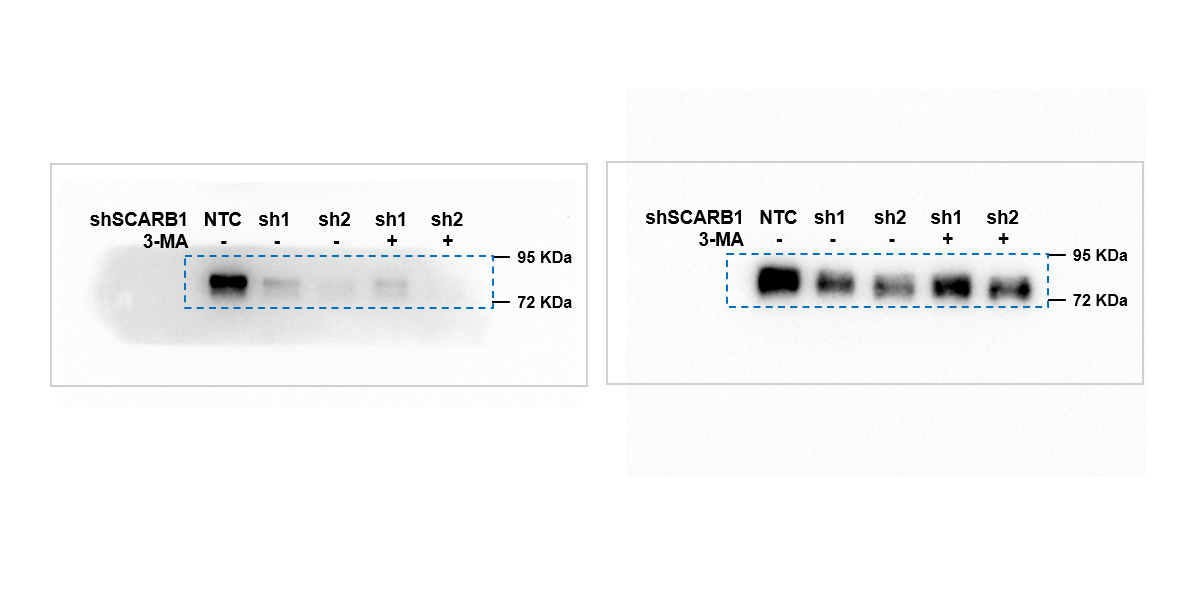

Supplement: Supplementary file 5 — Source data Fig. 3 [file 44319_2026_829_MOESM5_ESM.zip › Figure 3/G/SCARB1.png]

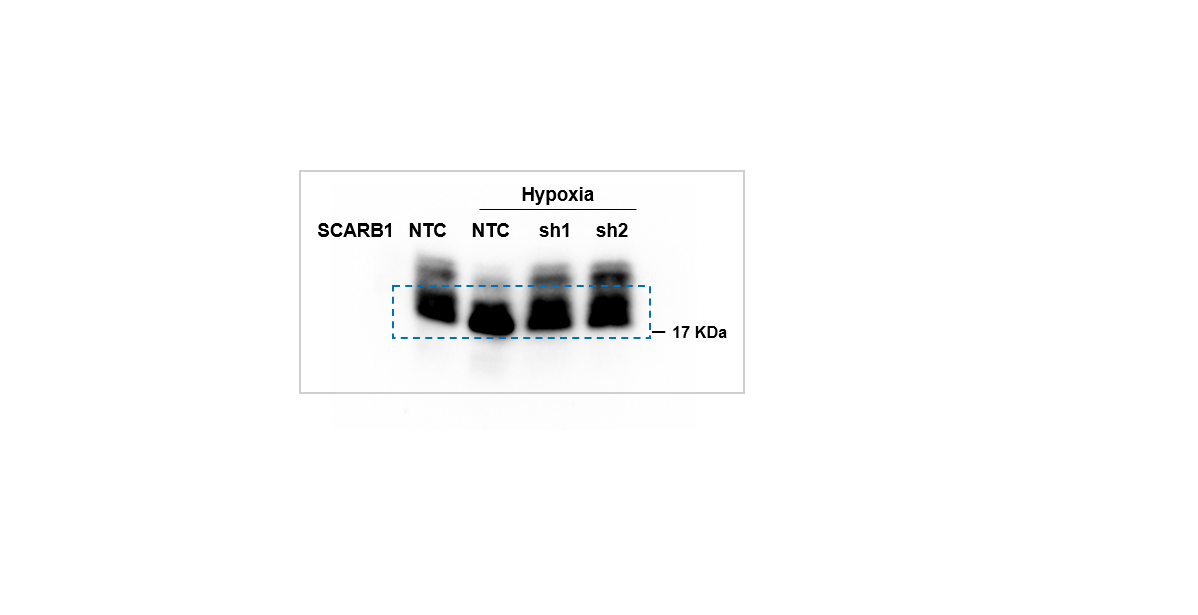

Supplement: Supplementary file 6 — Source data Fig. 4 [file 44319_2026_829_MOESM6_ESM.zip › Figure 4/A/4EBP1.png]

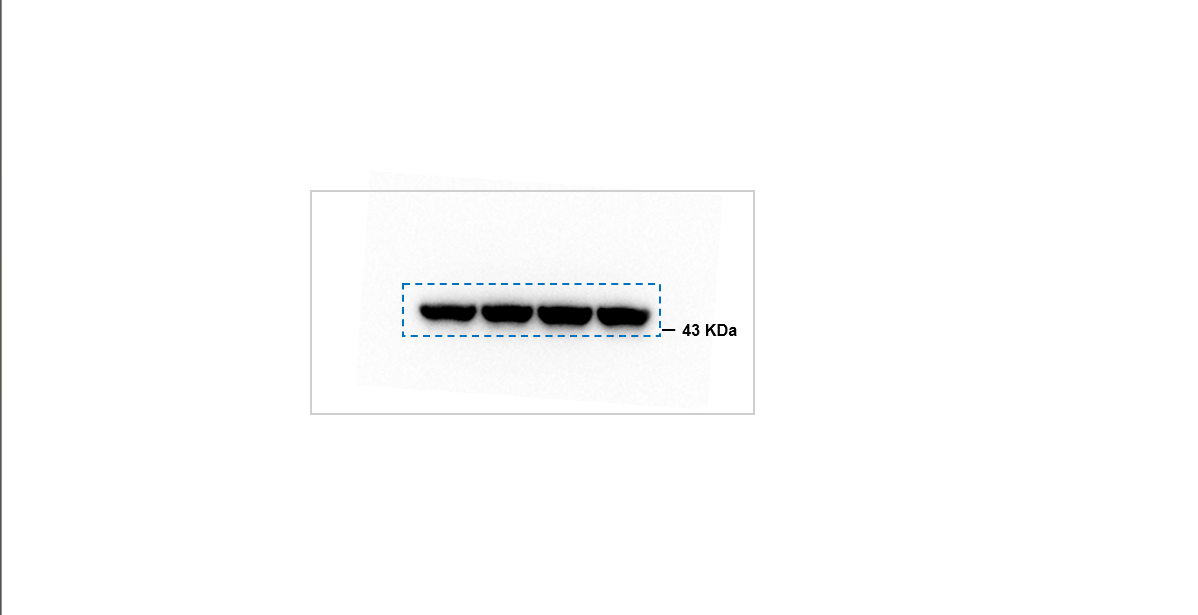

Supplement: Supplementary file 6 — Source data Fig. 4 [file 44319_2026_829_MOESM6_ESM.zip › Figure 4/A/ACTIN.png]

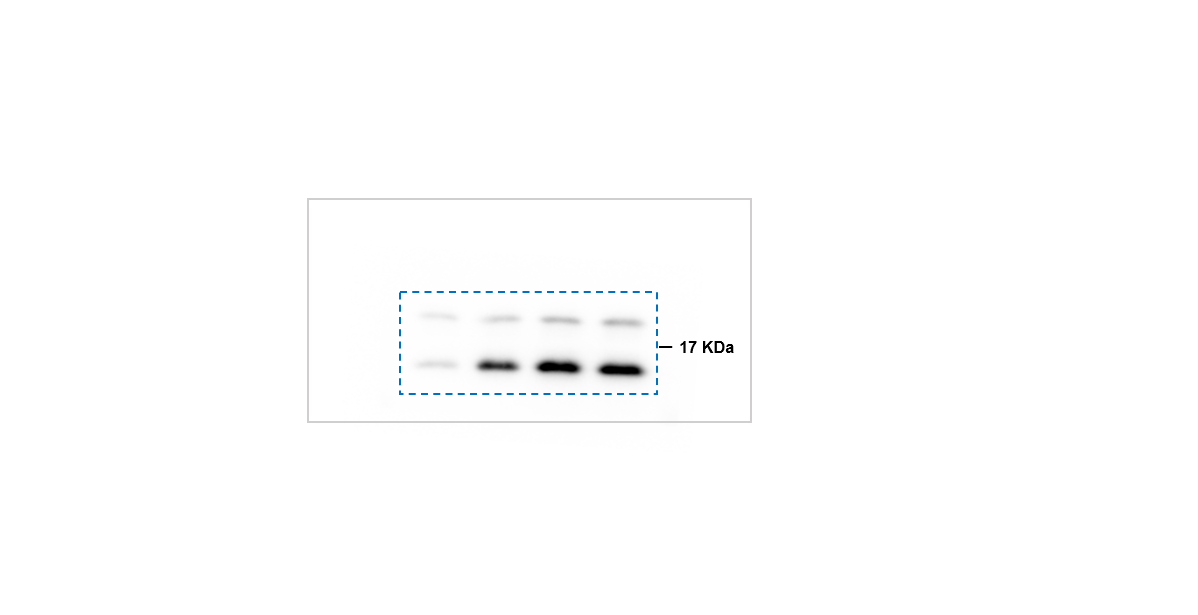

Supplement: Supplementary file 6 — Source data Fig. 4 [file 44319_2026_829_MOESM6_ESM.zip › Figure 4/A/LC3.png]

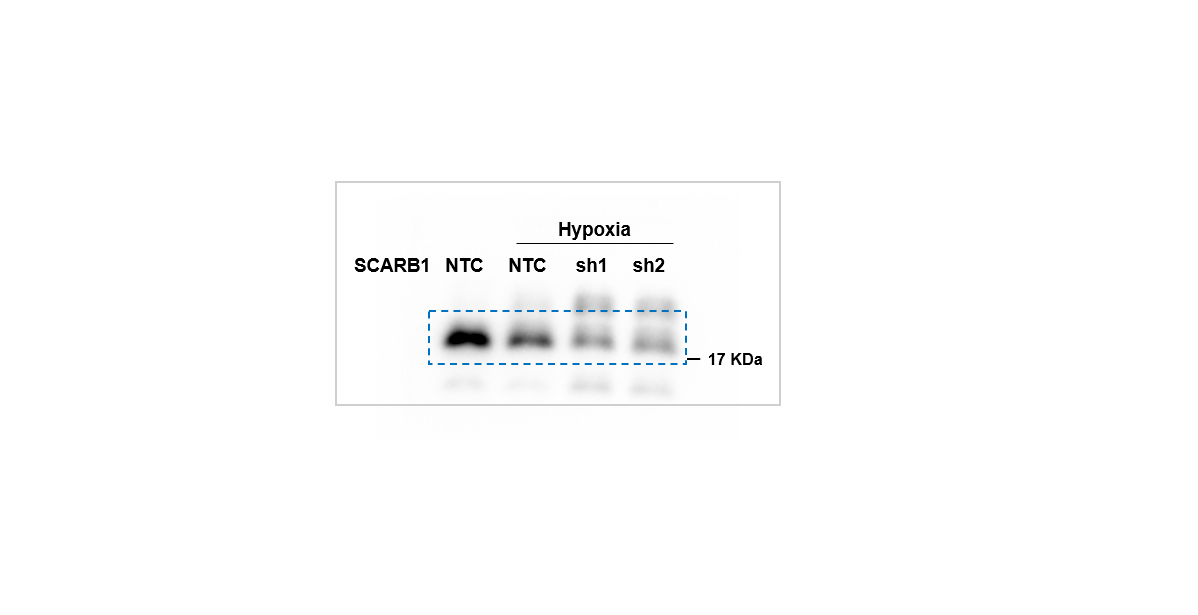

Supplement: Supplementary file 6 — Source data Fig. 4 [file 44319_2026_829_MOESM6_ESM.zip › Figure 4/A/p-4EBP1.png]

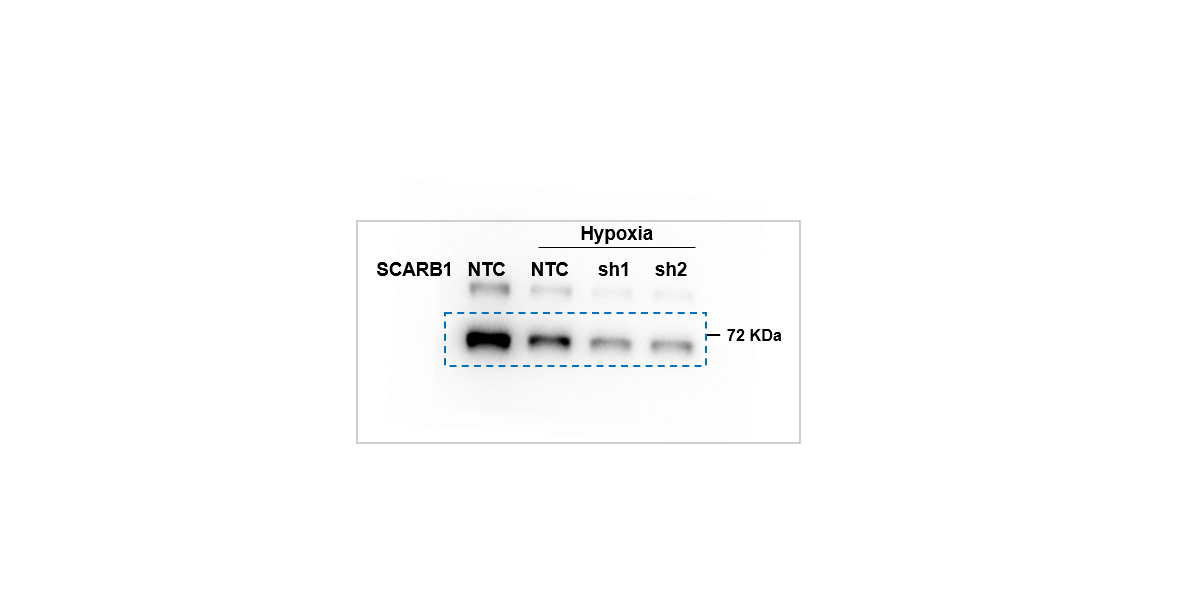

Supplement: Supplementary file 6 — Source data Fig. 4 [file 44319_2026_829_MOESM6_ESM.zip › Figure 4/A/p-S6K.png]

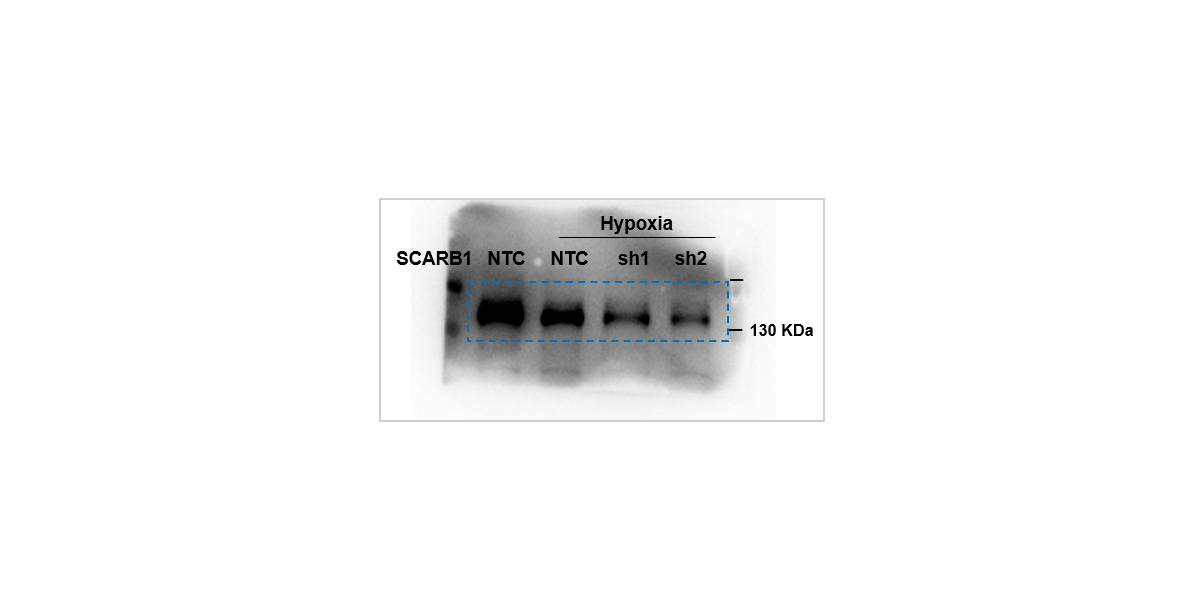

Supplement: Supplementary file 6 — Source data Fig. 4 [file 44319_2026_829_MOESM6_ESM.zip › Figure 4/A/p-ULK.png]

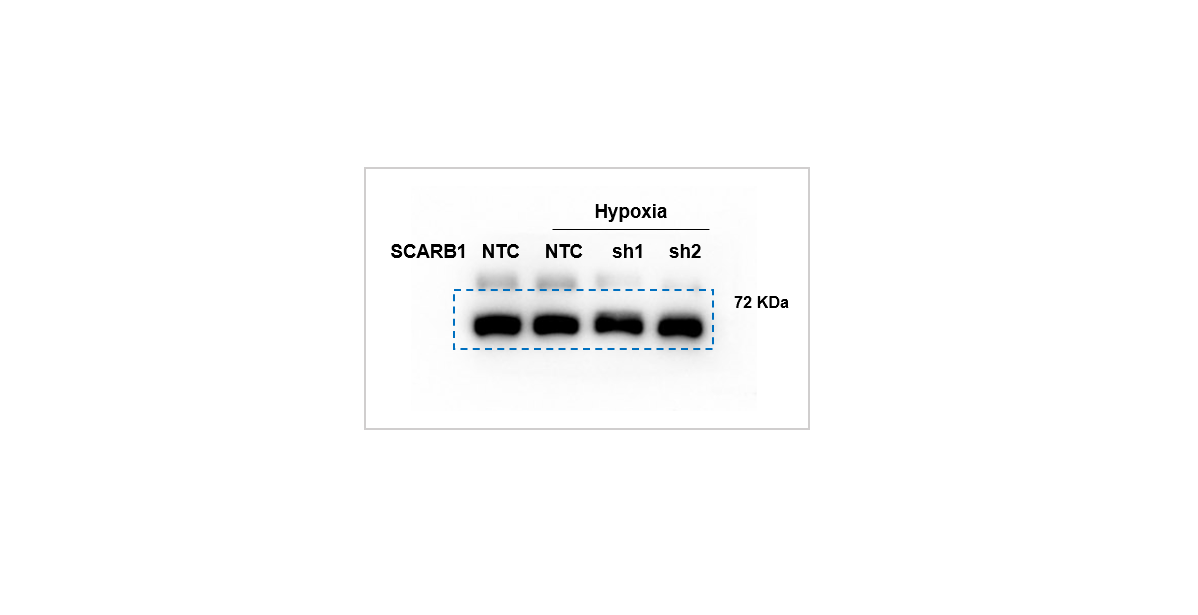

Supplement: Supplementary file 6 — Source data Fig. 4 [file 44319_2026_829_MOESM6_ESM.zip › Figure 4/A/S6K.png]

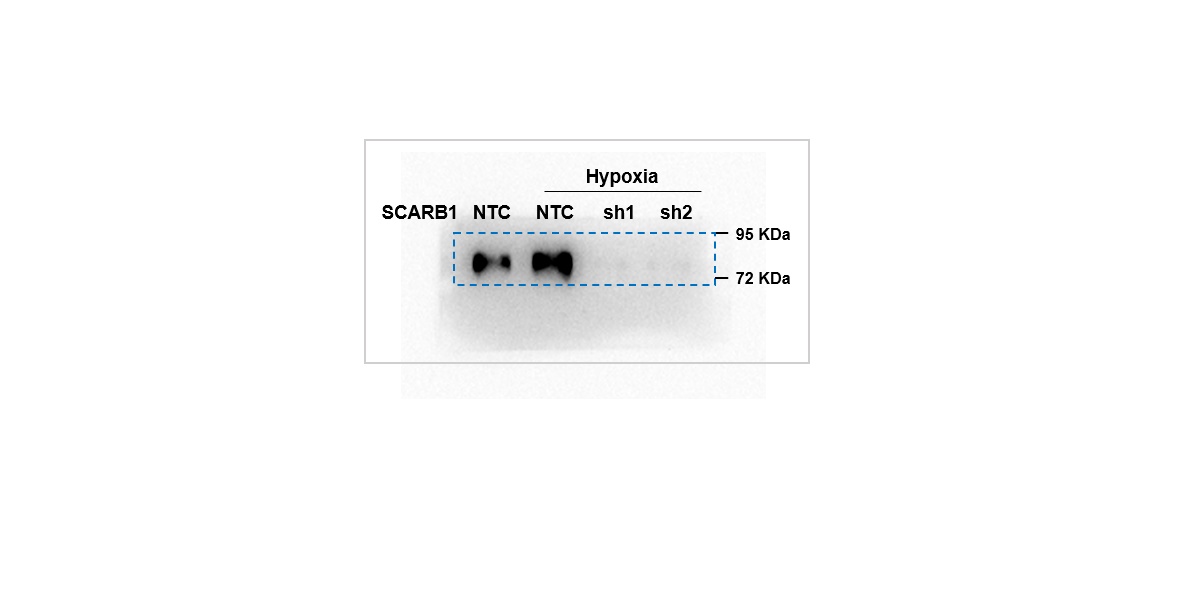

Supplement: Supplementary file 6 — Source data Fig. 4 [file 44319_2026_829_MOESM6_ESM.zip › Figure 4/A/SCARB1.png]

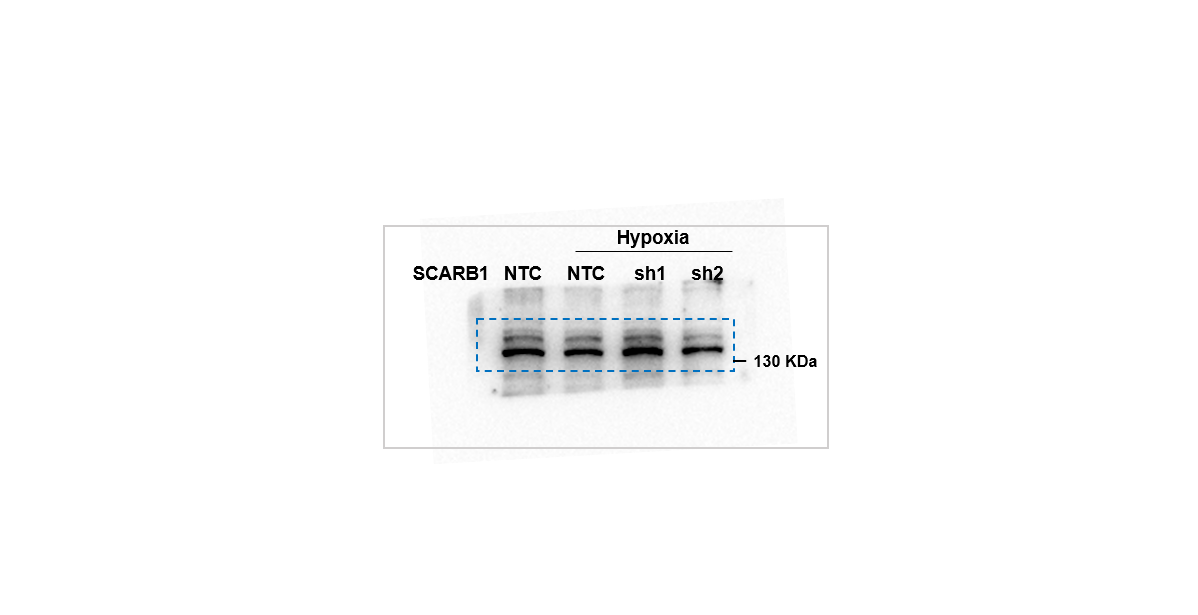

Supplement: Supplementary file 6 — Source data Fig. 4 [file 44319_2026_829_MOESM6_ESM.zip › Figure 4/A/ULK.png]

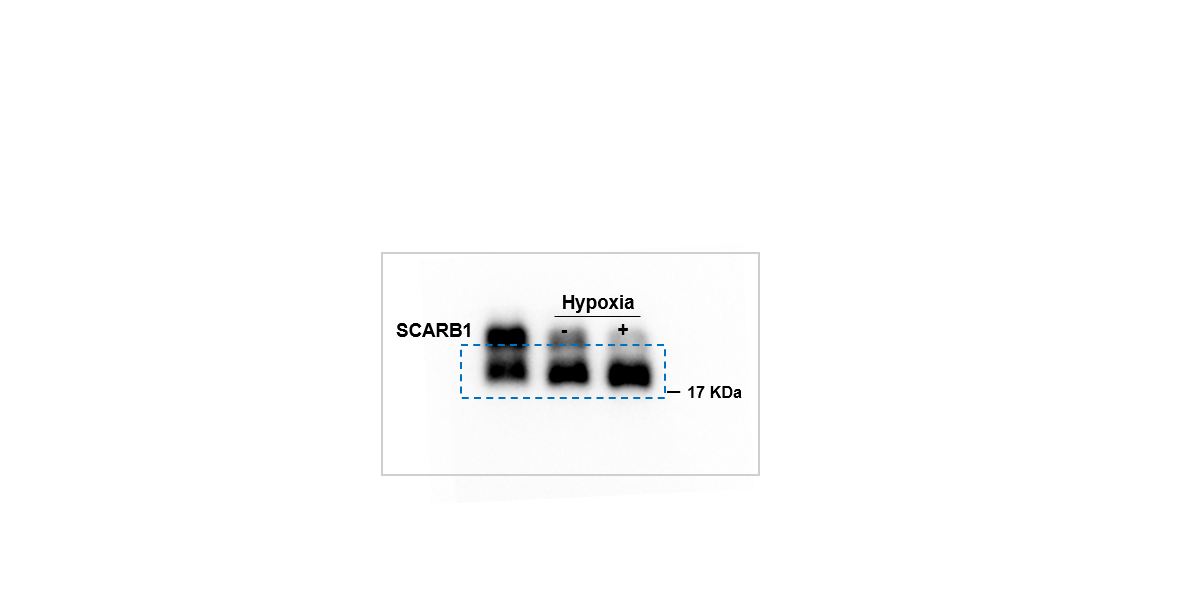

Supplement: Supplementary file 6 — Source data Fig. 4 [file 44319_2026_829_MOESM6_ESM.zip › Figure 4/B/4EBP1.png]

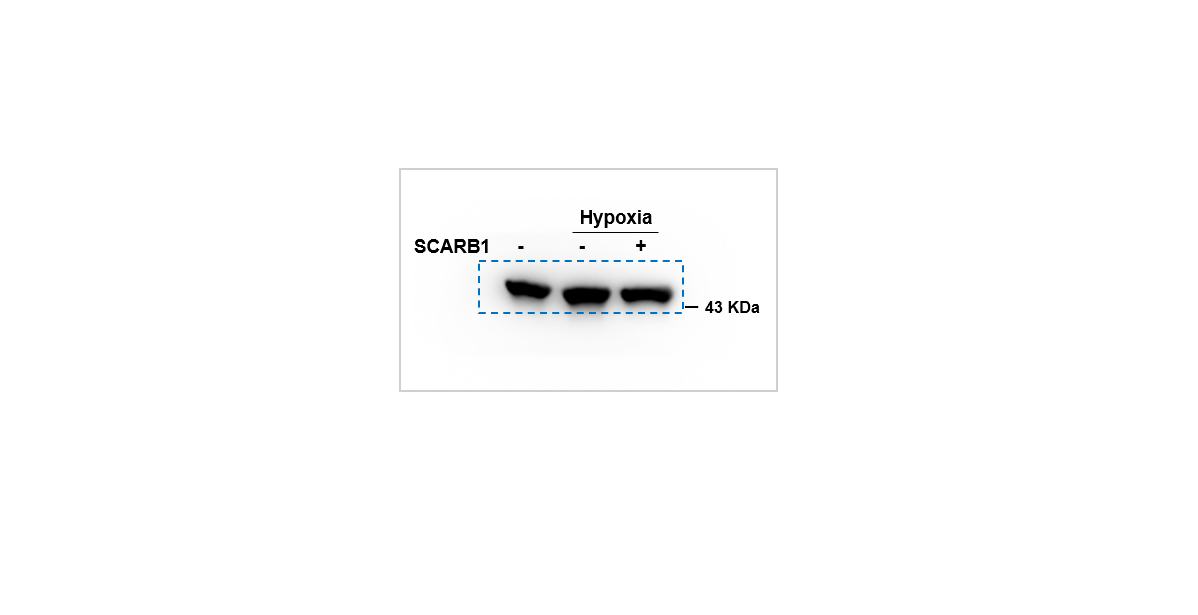

Supplement: Supplementary file 6 — Source data Fig. 4 [file 44319_2026_829_MOESM6_ESM.zip › Figure 4/B/ACTIN.png]

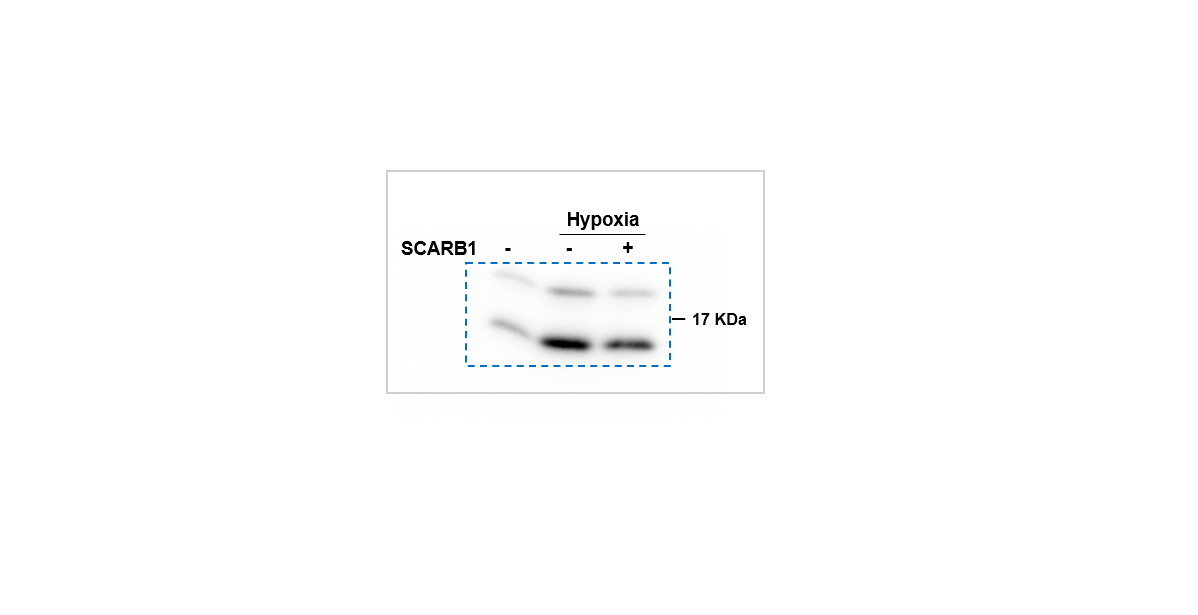

Supplement: Supplementary file 6 — Source data Fig. 4 [file 44319_2026_829_MOESM6_ESM.zip › Figure 4/B/LC3.png]

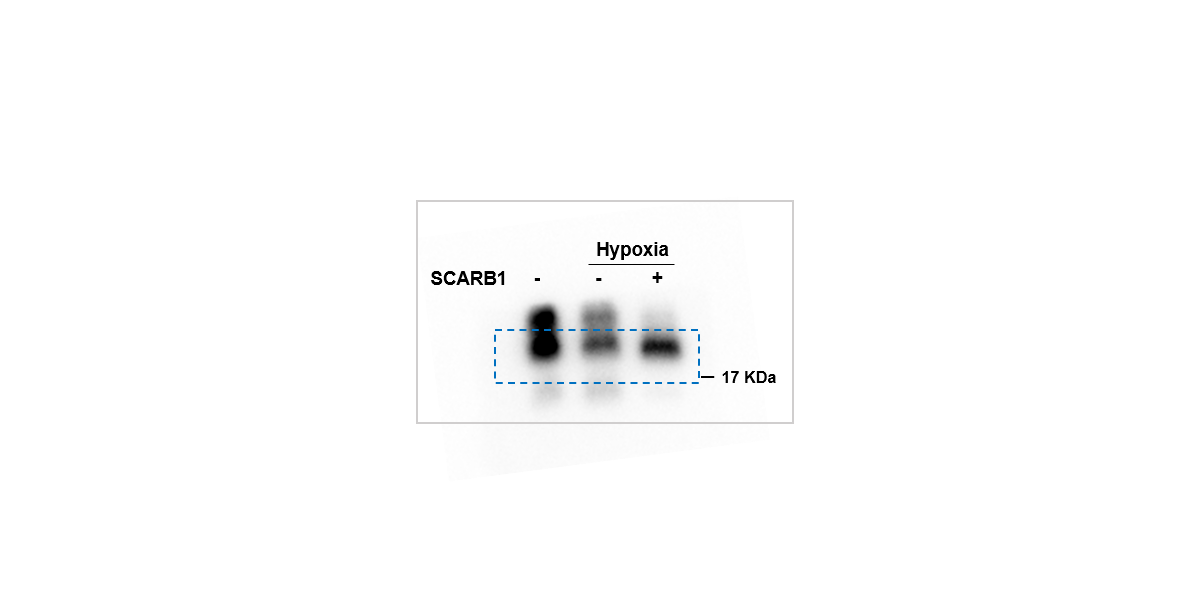

Supplement: Supplementary file 6 — Source data Fig. 4 [file 44319_2026_829_MOESM6_ESM.zip › Figure 4/B/p-4EBP1.png]

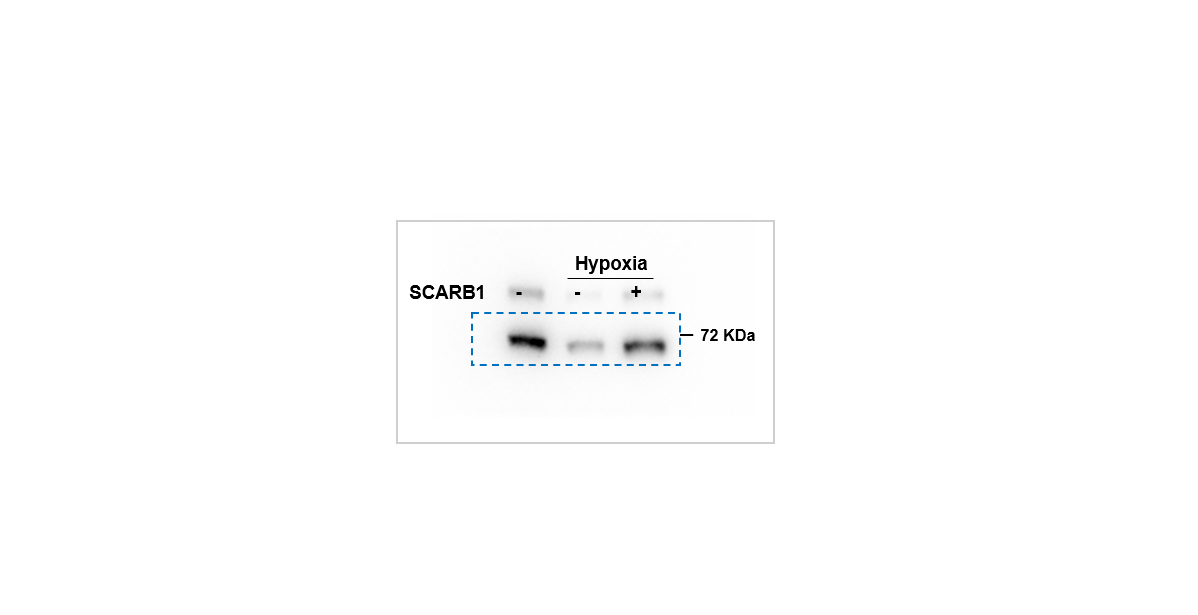

Supplement: Supplementary file 6 — Source data Fig. 4 [file 44319_2026_829_MOESM6_ESM.zip › Figure 4/B/p-S6K.png]

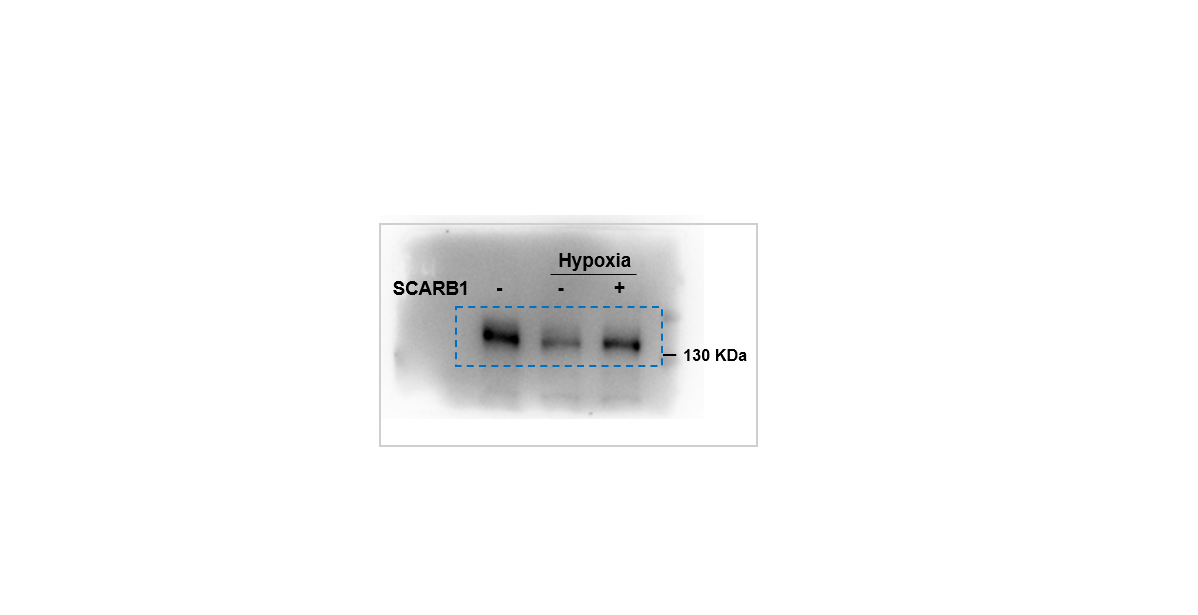

Supplement: Supplementary file 6 — Source data Fig. 4 [file 44319_2026_829_MOESM6_ESM.zip › Figure 4/B/p-ULK.png]

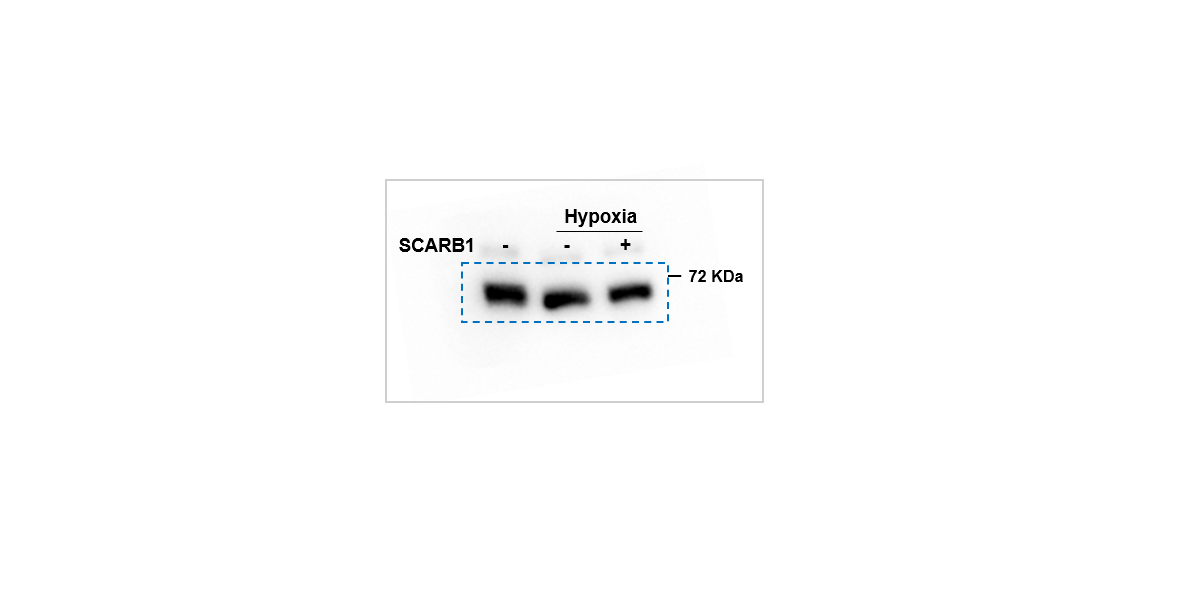

Supplement: Supplementary file 6 — Source data Fig. 4 [file 44319_2026_829_MOESM6_ESM.zip › Figure 4/B/S6K.png]

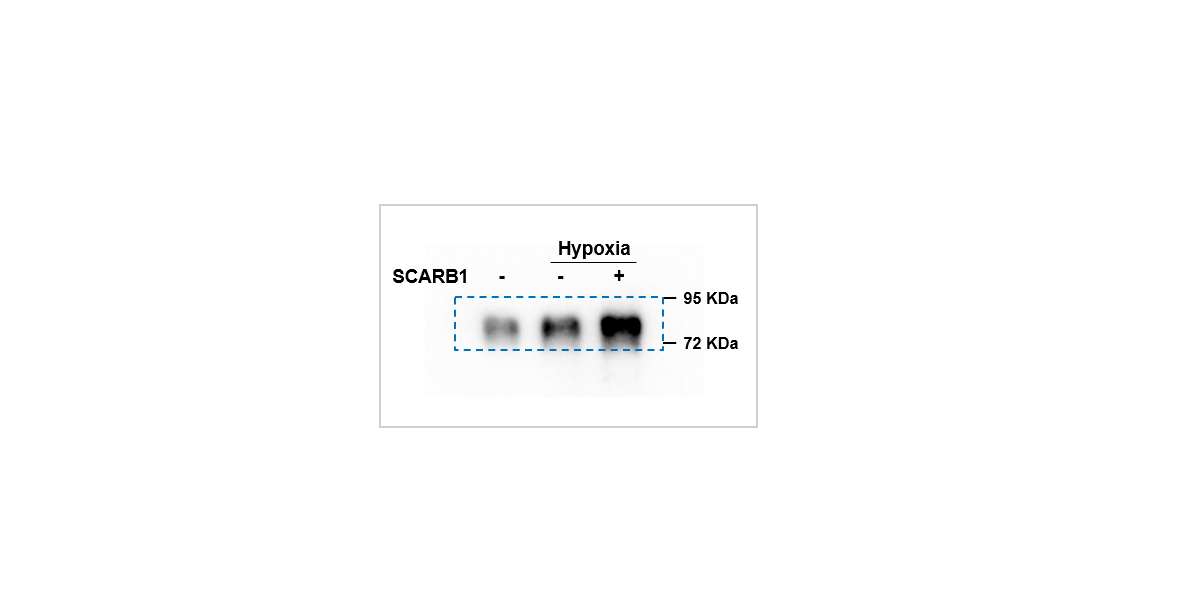

Supplement: Supplementary file 6 — Source data Fig. 4 [file 44319_2026_829_MOESM6_ESM.zip › Figure 4/B/SCARB1.png]

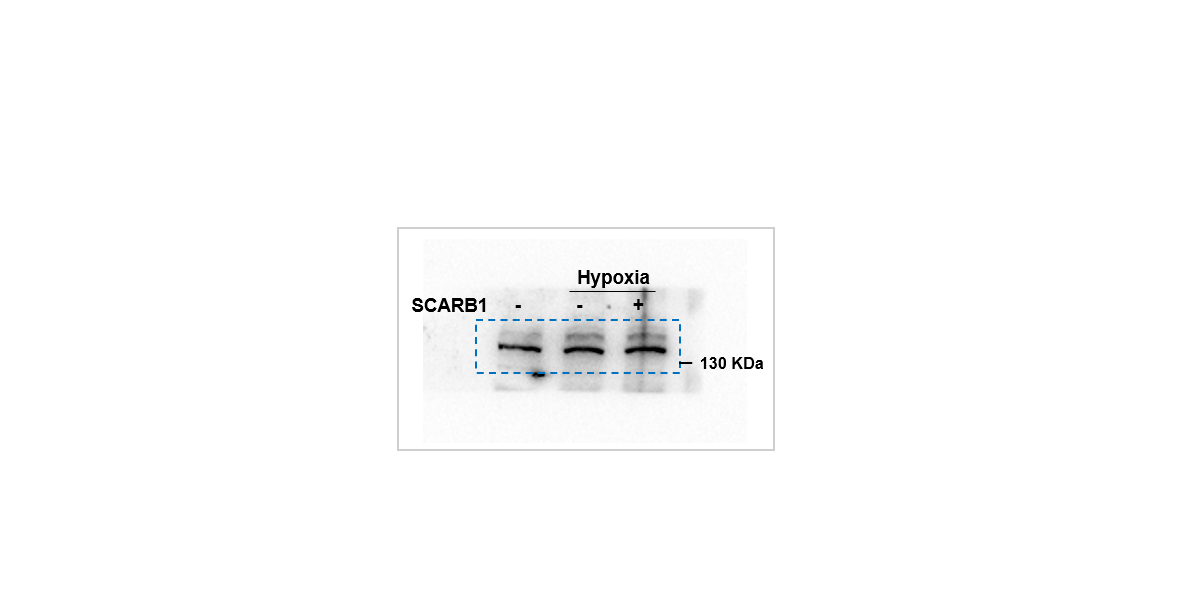

Supplement: Supplementary file 6 — Source data Fig. 4 [file 44319_2026_829_MOESM6_ESM.zip › Figure 4/B/ULK.png]

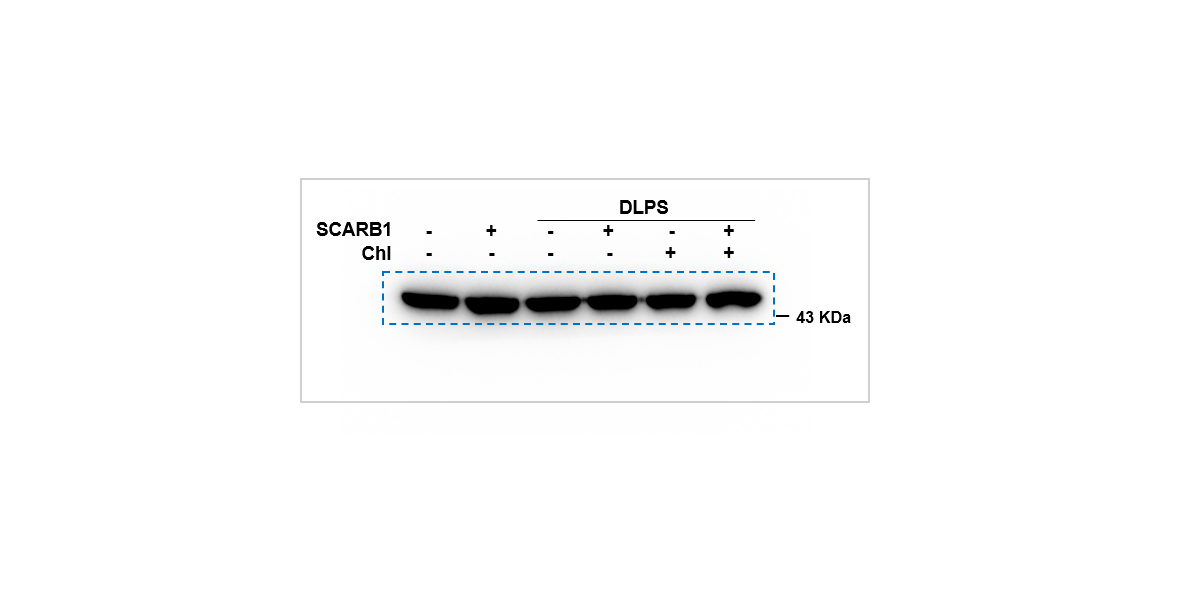

Supplement: Supplementary file 6 — Source data Fig. 4 [file 44319_2026_829_MOESM6_ESM.zip › Figure 4/C/actin.png]

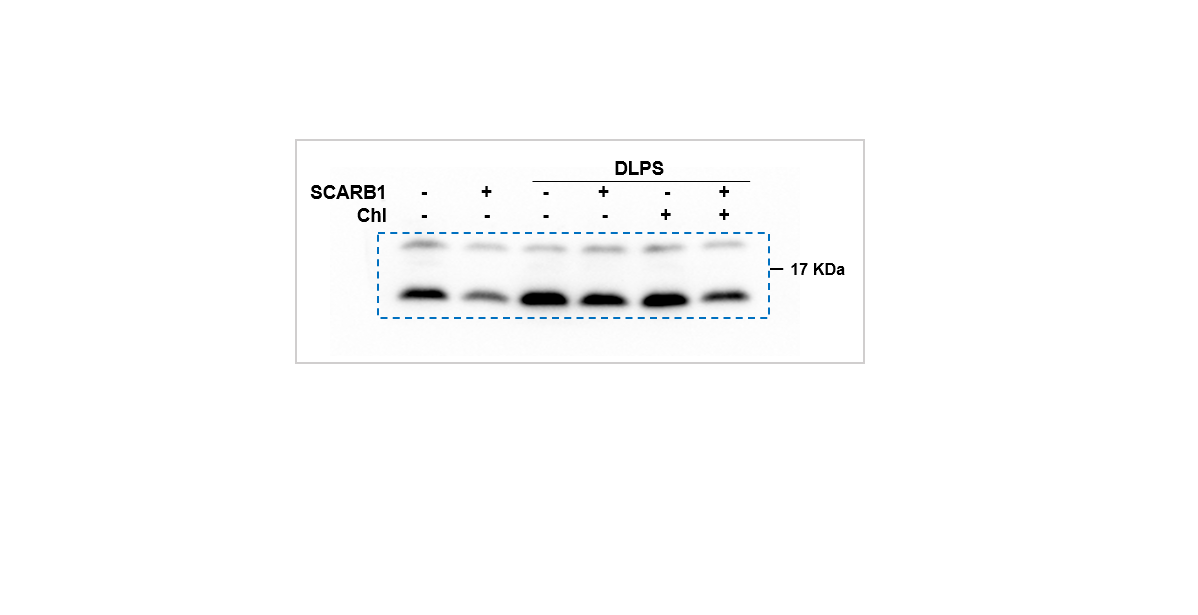

Supplement: Supplementary file 6 — Source data Fig. 4 [file 44319_2026_829_MOESM6_ESM.zip › Figure 4/C/LC3.png]

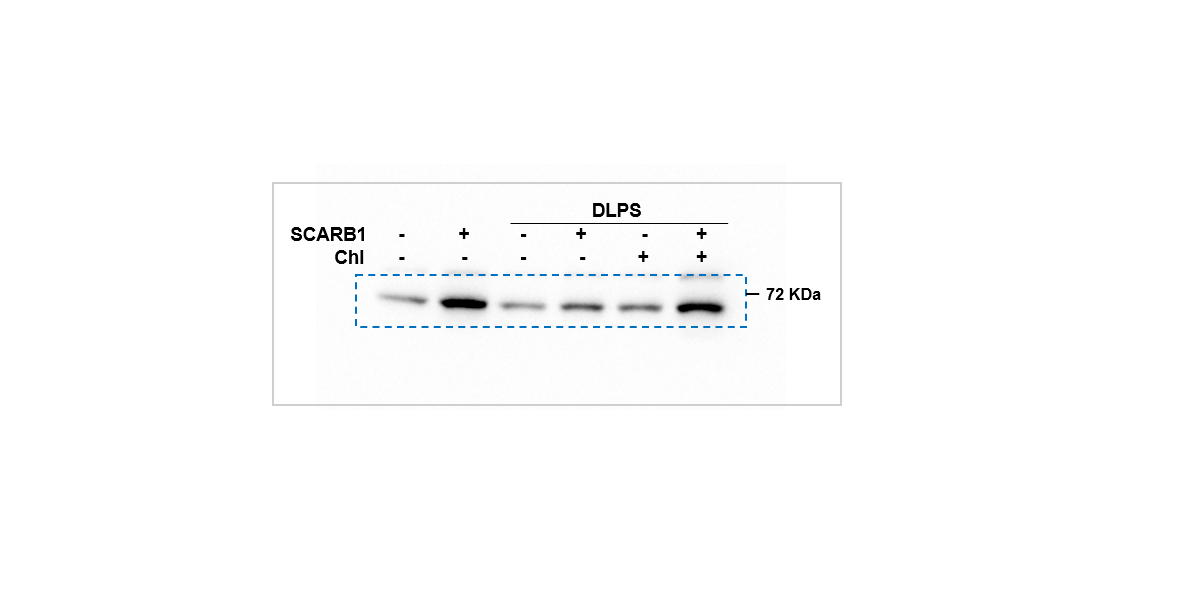

Supplement: Supplementary file 6 — Source data Fig. 4 [file 44319_2026_829_MOESM6_ESM.zip › Figure 4/C/p-S6K.png]

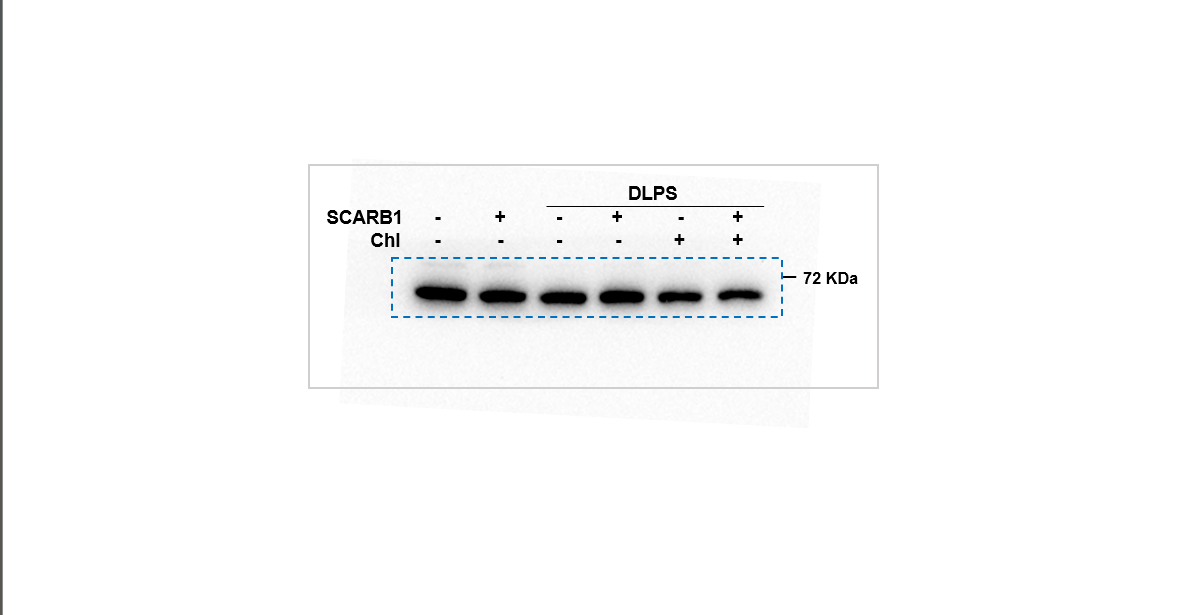

Supplement: Supplementary file 6 — Source data Fig. 4 [file 44319_2026_829_MOESM6_ESM.zip › Figure 4/C/S6K.png]

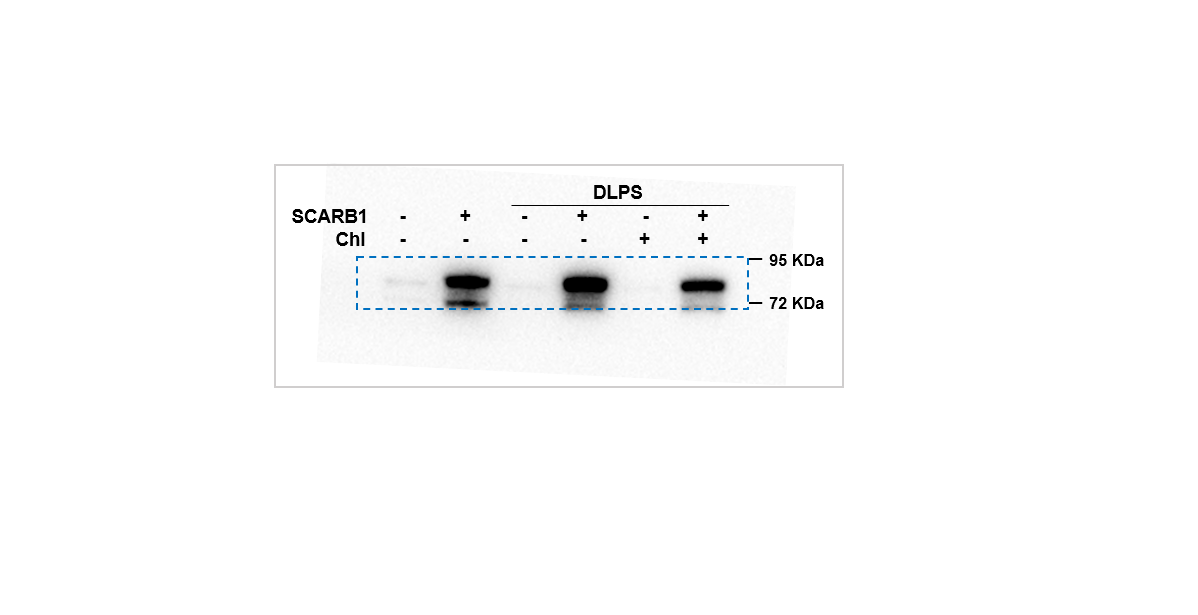

Supplement: Supplementary file 6 — Source data Fig. 4 [file 44319_2026_829_MOESM6_ESM.zip › Figure 4/C/SCARB1.png]

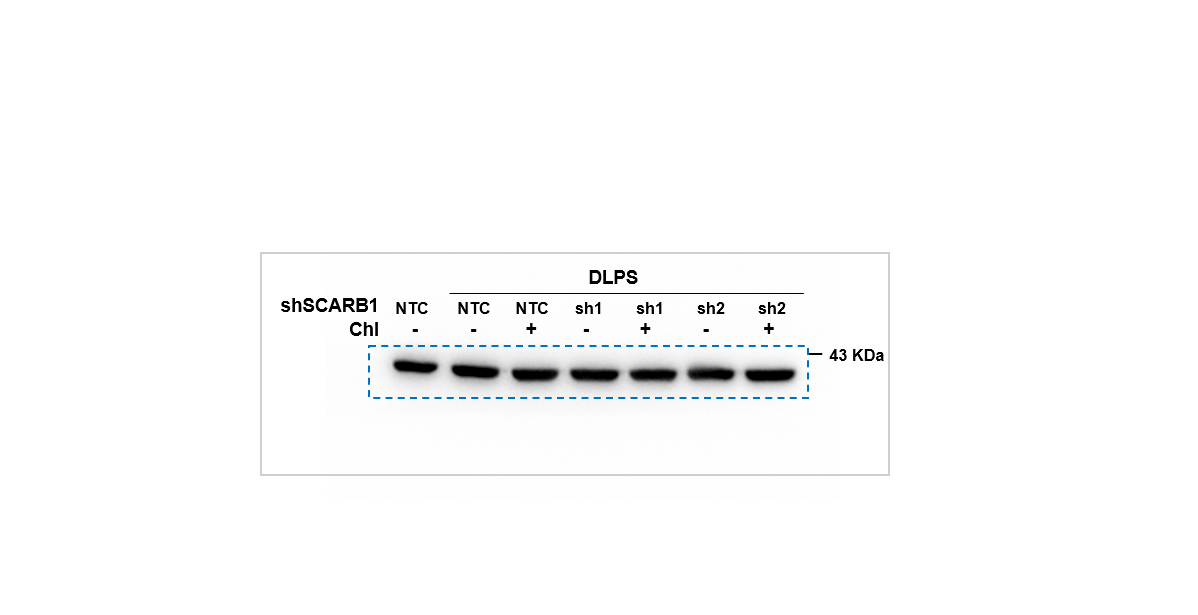

Supplement: Supplementary file 6 — Source data Fig. 4 [file 44319_2026_829_MOESM6_ESM.zip › Figure 4/D/ACTIN.png]

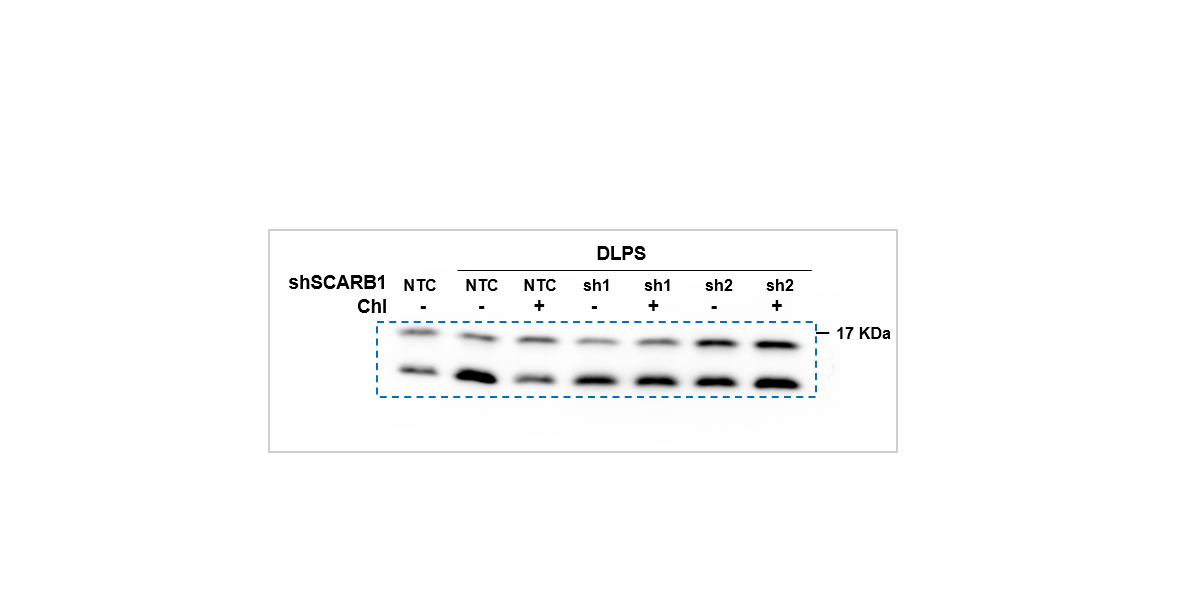

Supplement: Supplementary file 6 — Source data Fig. 4 [file 44319_2026_829_MOESM6_ESM.zip › Figure 4/D/LC3.png]

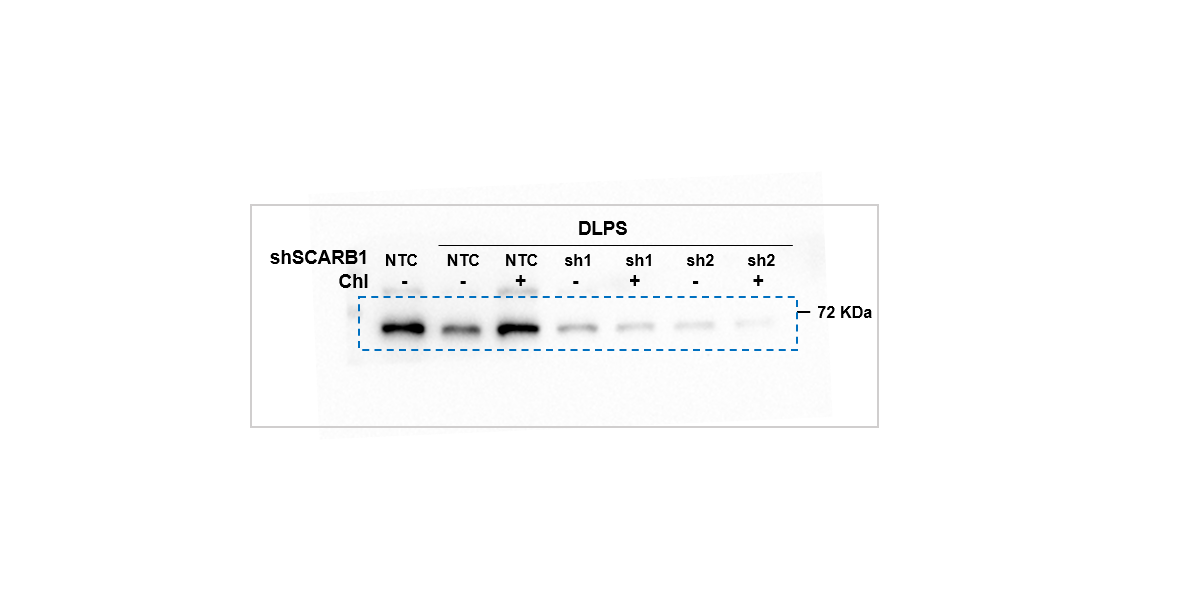

Supplement: Supplementary file 6 — Source data Fig. 4 [file 44319_2026_829_MOESM6_ESM.zip › Figure 4/D/p-S6K.png]

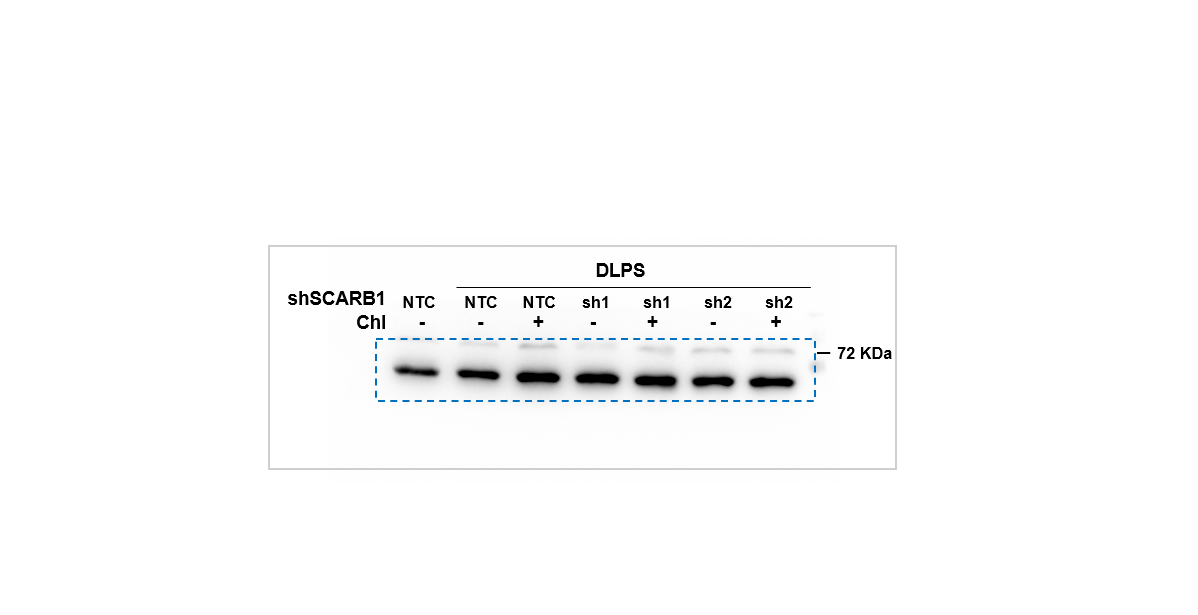

Supplement: Supplementary file 6 — Source data Fig. 4 [file 44319_2026_829_MOESM6_ESM.zip › Figure 4/D/S6K.png]

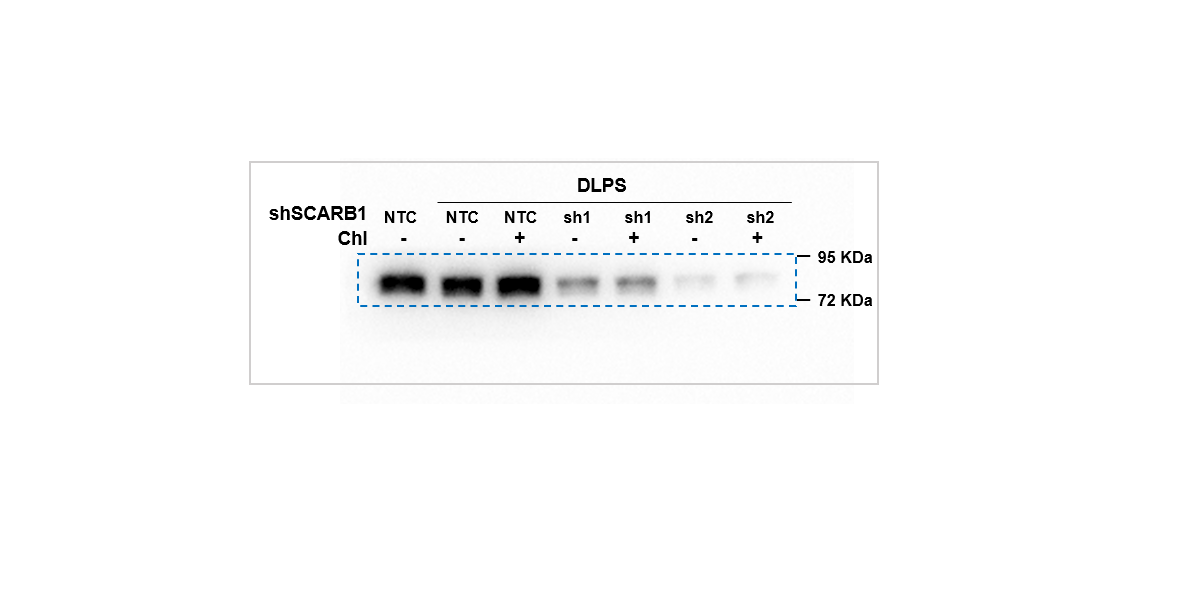

Supplement: Supplementary file 6 — Source data Fig. 4 [file 44319_2026_829_MOESM6_ESM.zip › Figure 4/D/SCARB1.png]

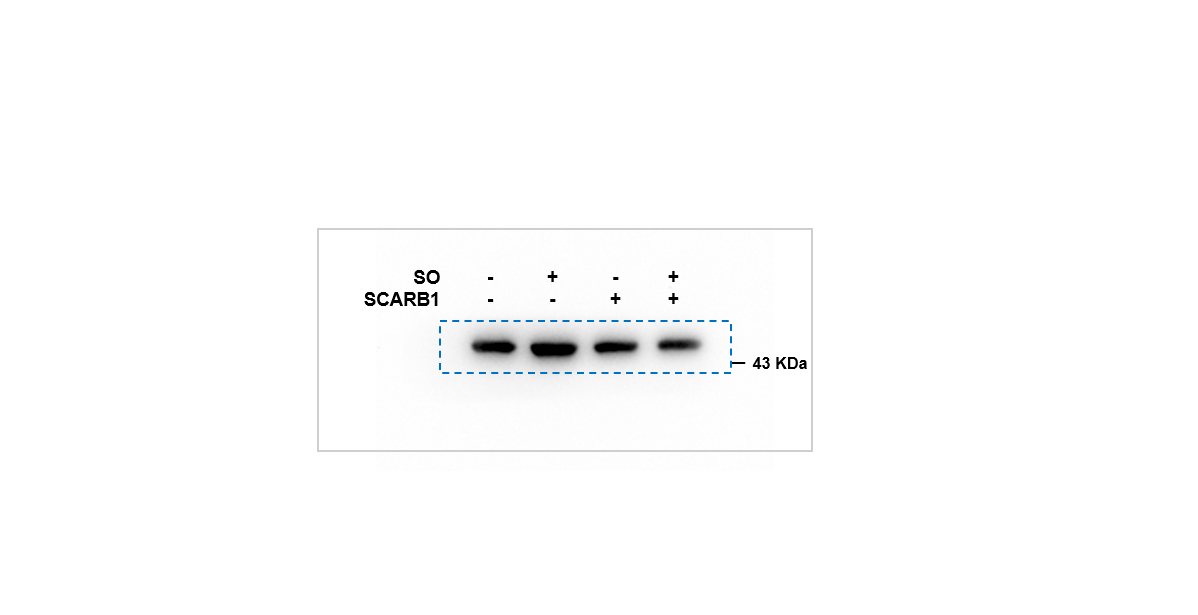

Supplement: Supplementary file 6 — Source data Fig. 4 [file 44319_2026_829_MOESM6_ESM.zip › Figure 4/E/ACTIN.png]

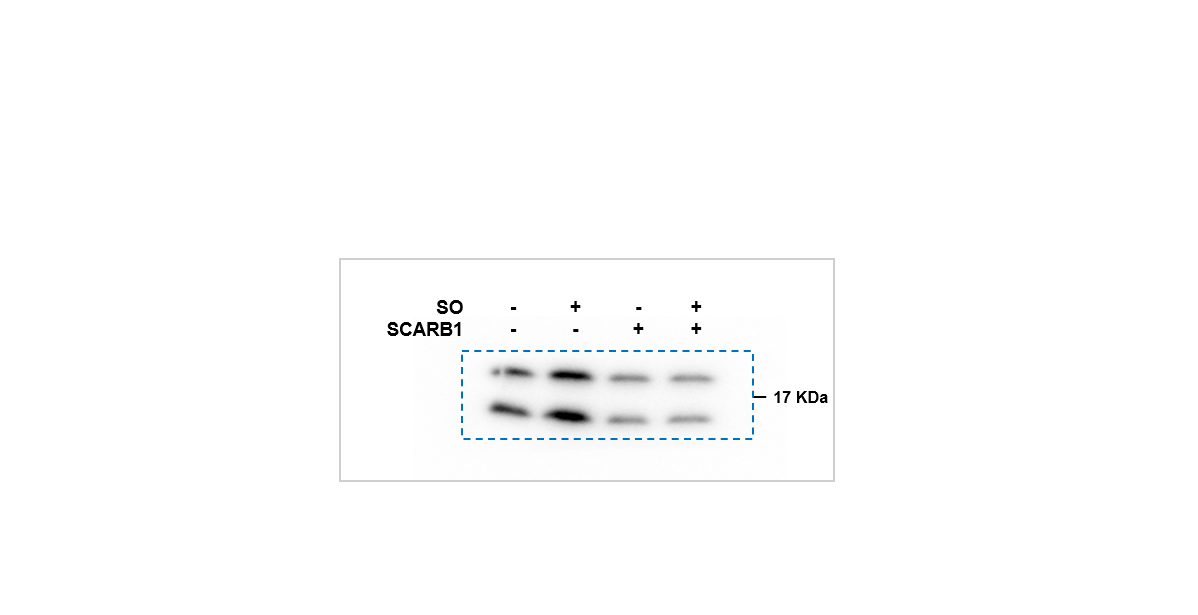

Supplement: Supplementary file 6 — Source data Fig. 4 [file 44319_2026_829_MOESM6_ESM.zip › Figure 4/E/LC3.png]

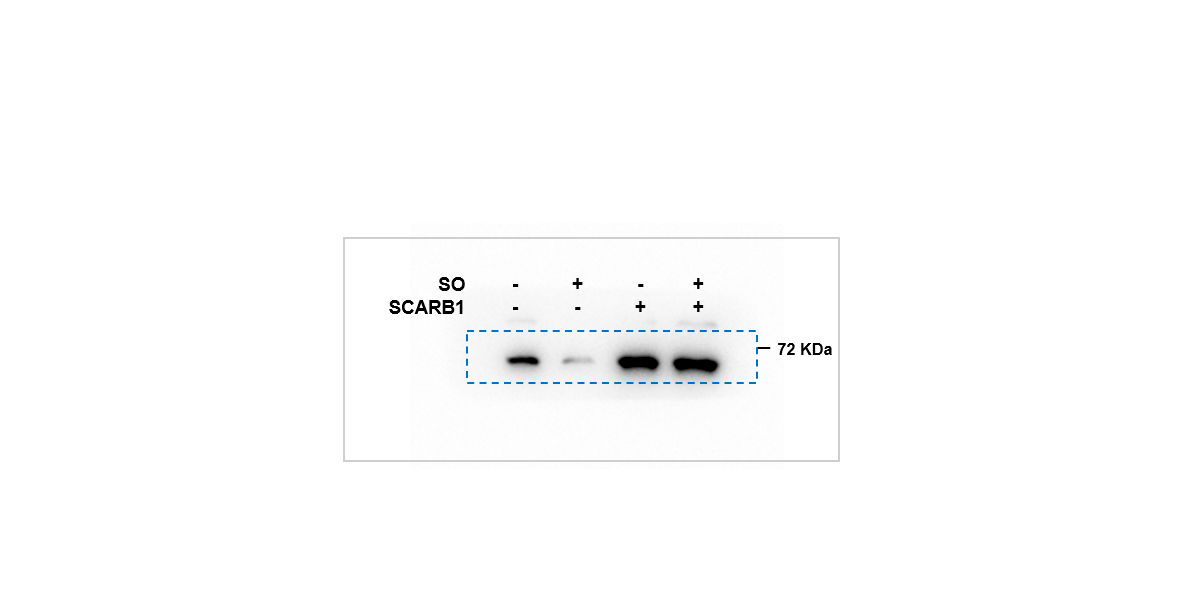

Supplement: Supplementary file 6 — Source data Fig. 4 [file 44319_2026_829_MOESM6_ESM.zip › Figure 4/E/p-S6K.png]

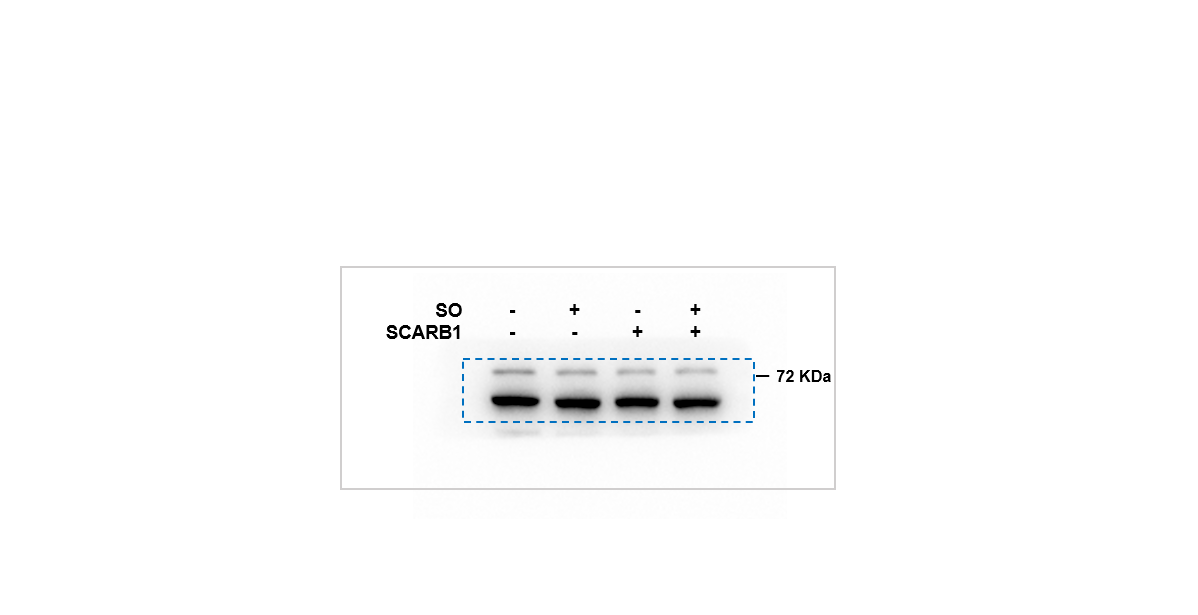

Supplement: Supplementary file 6 — Source data Fig. 4 [file 44319_2026_829_MOESM6_ESM.zip › Figure 4/E/S6K.png]

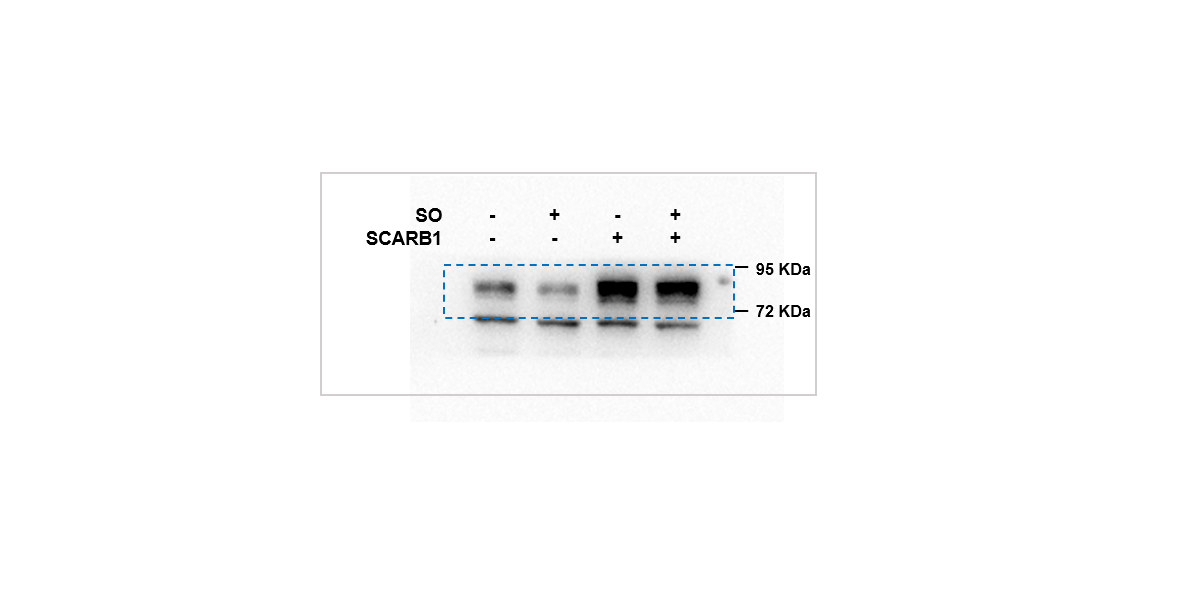

Supplement: Supplementary file 6 — Source data Fig. 4 [file 44319_2026_829_MOESM6_ESM.zip › Figure 4/E/SCARB1.png]

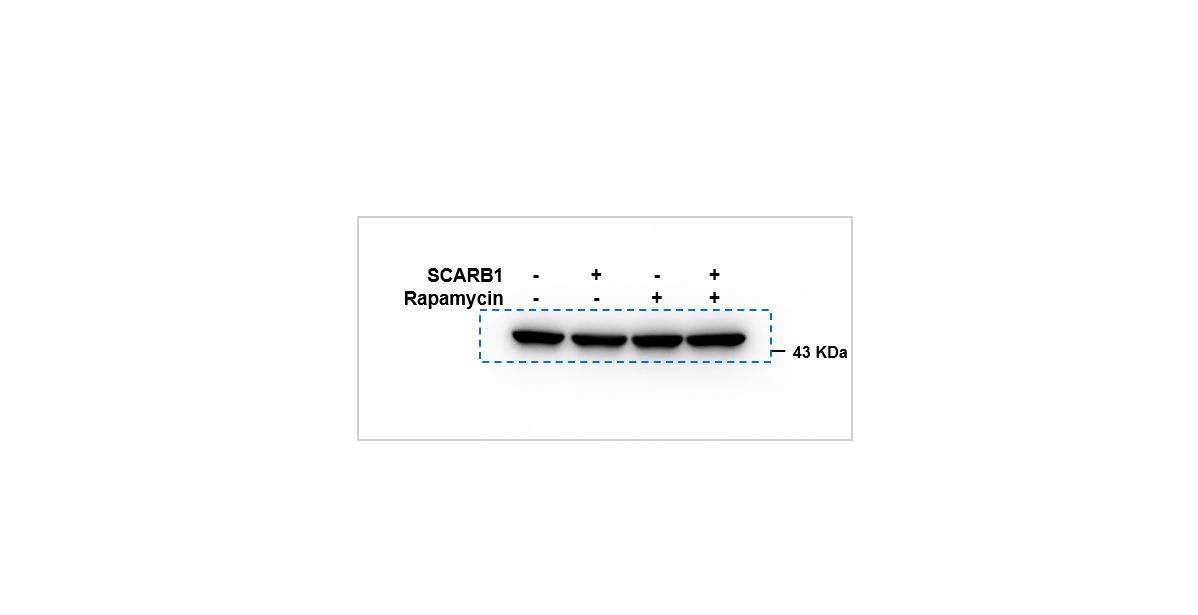

Supplement: Supplementary file 6 — Source data Fig. 4 [file 44319_2026_829_MOESM6_ESM.zip › Figure 4/F/ACTIN.png]

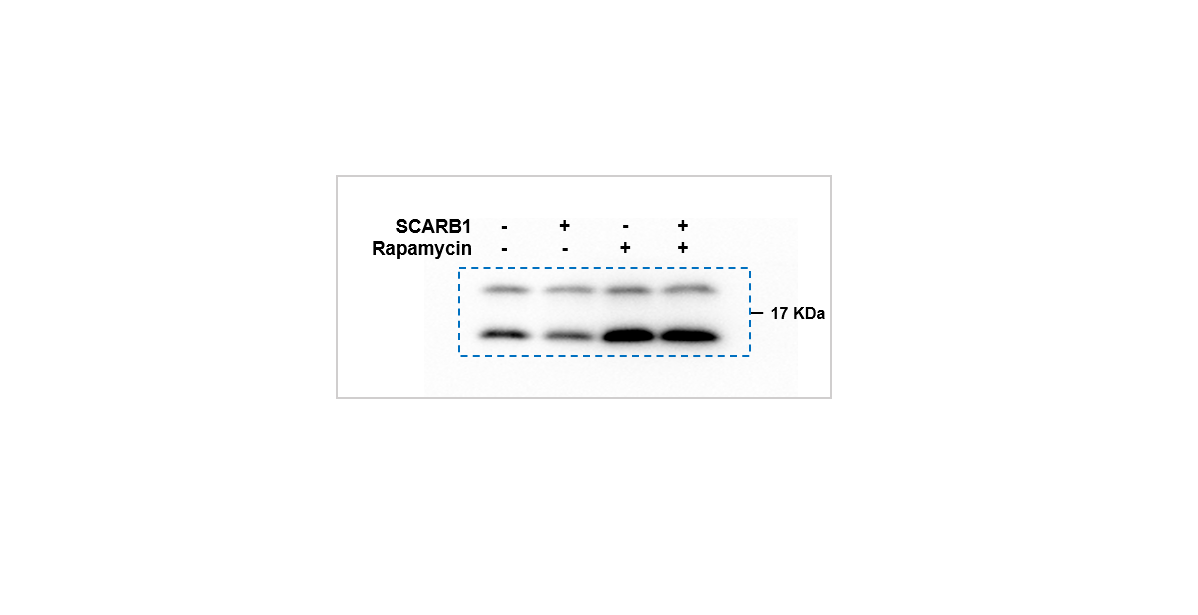

Supplement: Supplementary file 6 — Source data Fig. 4 [file 44319_2026_829_MOESM6_ESM.zip › Figure 4/F/LC3.png]

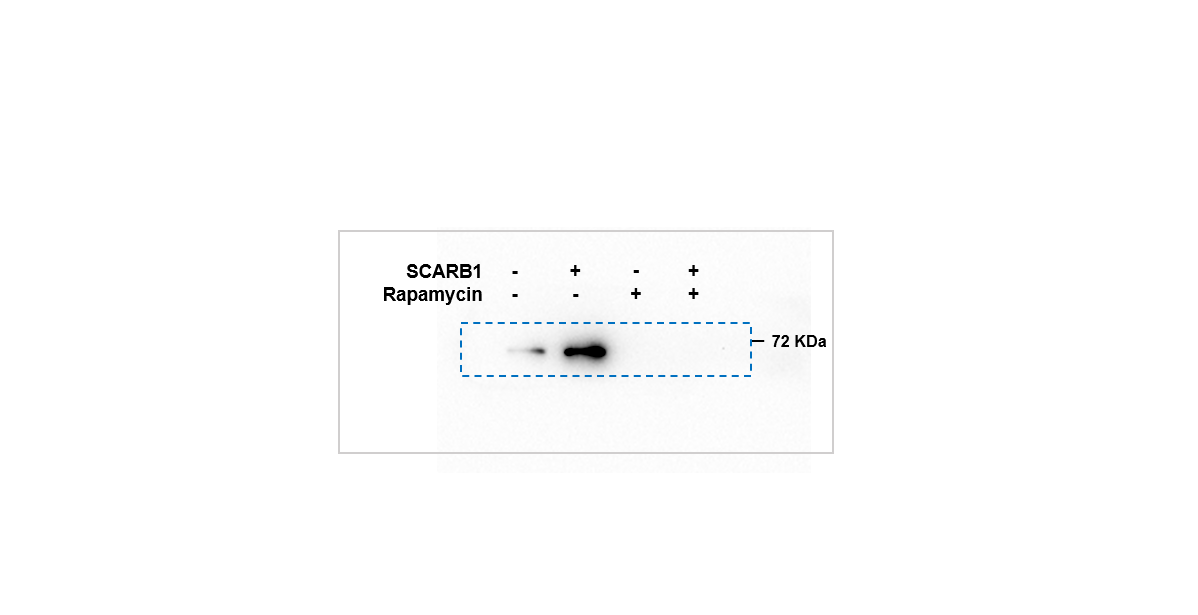

Supplement: Supplementary file 6 — Source data Fig. 4 [file 44319_2026_829_MOESM6_ESM.zip › Figure 4/F/p-S6K.png]
