# Supplementary figures and images for: Lactate-mediated cholesterol uptake promotes liver cancer progression via the SCARB1-autophagy axis (part 2 of 2)
Source: EMBO Rep. 2026 Jun 10;27(14):4141–65. doi: 10.1038/s44319-026-00829-x (PMC13400630; doi:10.1038/s44319-026-00829-x)

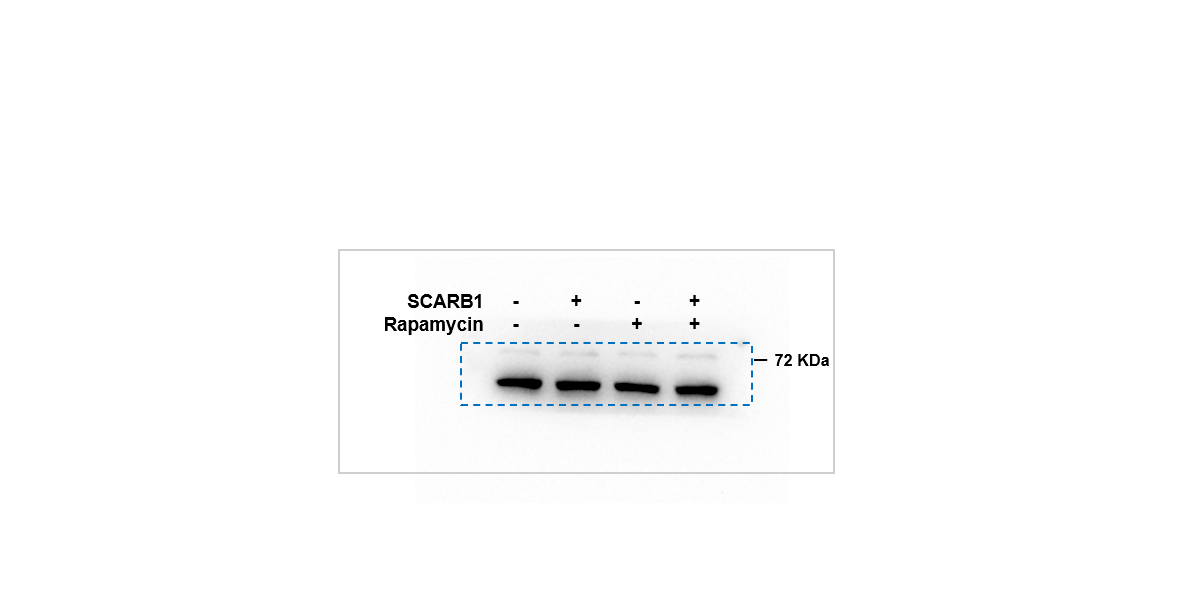

Supplement: Supplementary file 6 — Source data Fig. 4 [file 44319_2026_829_MOESM6_ESM.zip › Figure 4/F/S6K.png]

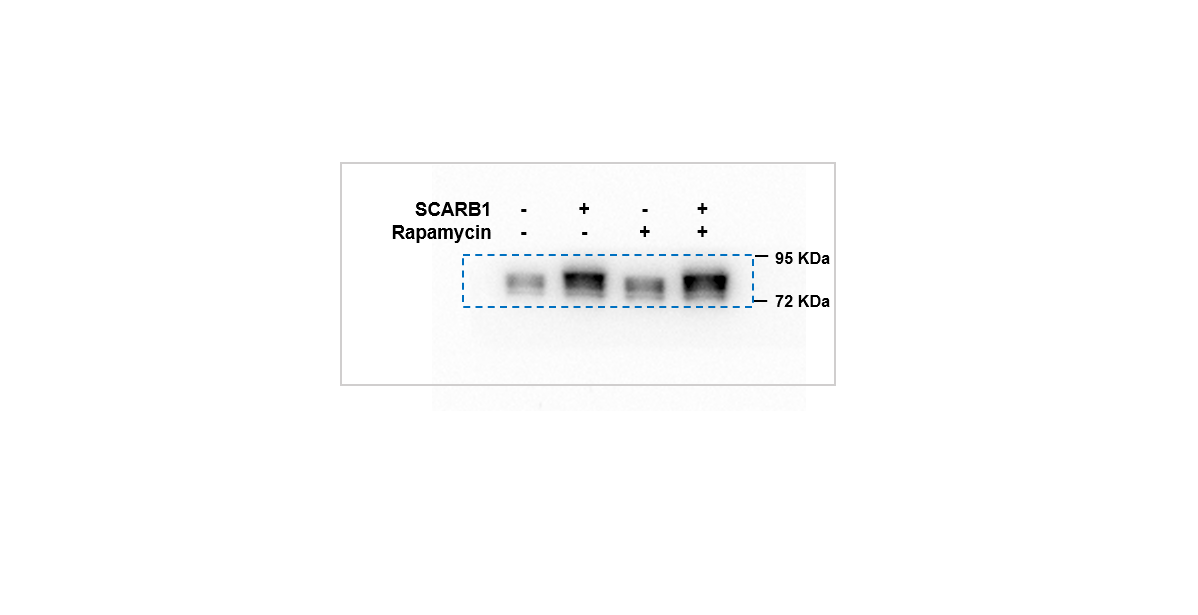

Supplement: Supplementary file 6 — Source data Fig. 4 [file 44319_2026_829_MOESM6_ESM.zip › Figure 4/F/SCARB1.png]

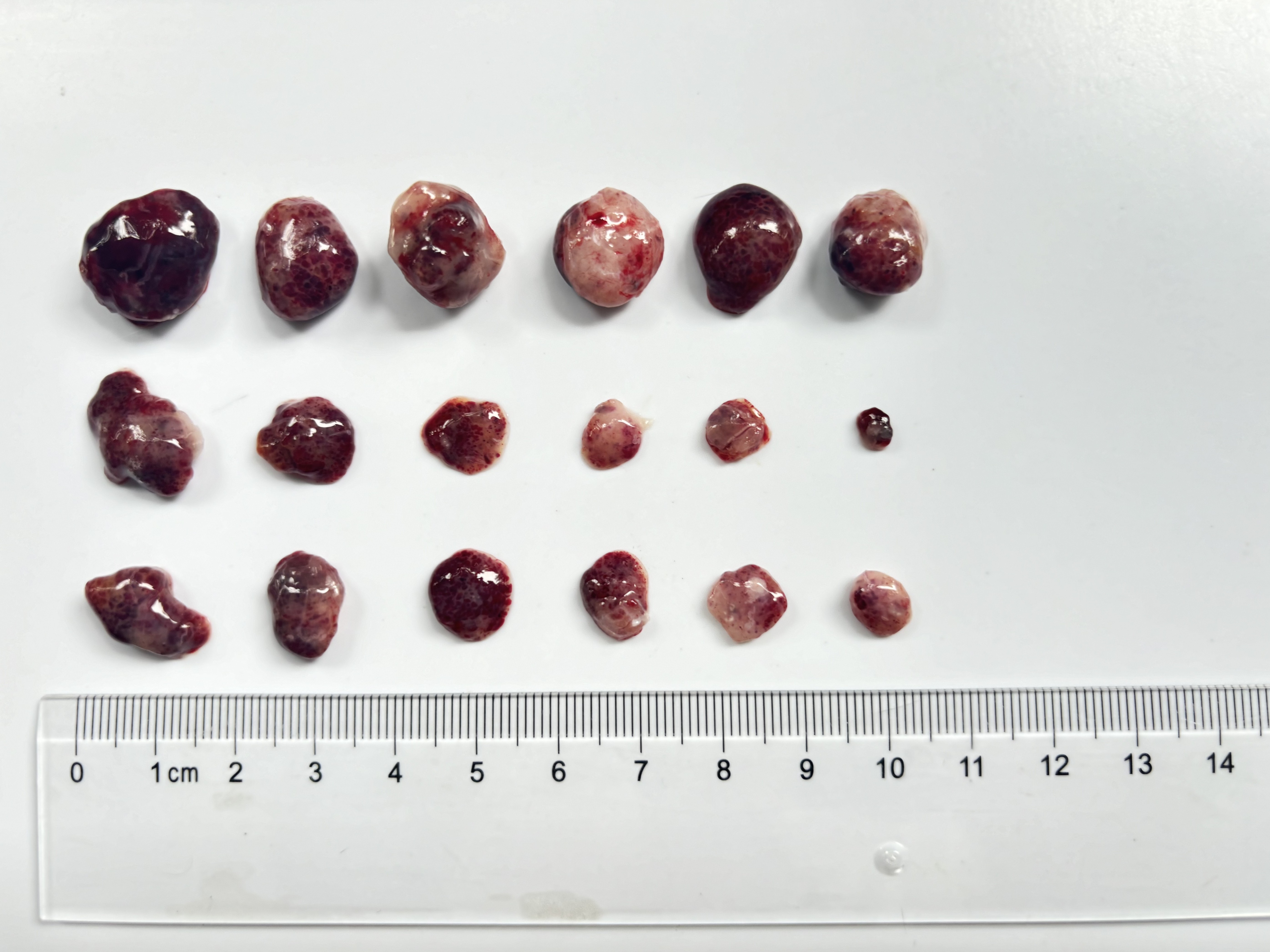

Supplement: Supplementary file 7 — Source data Fig. 5 [file 44319_2026_829_MOESM7_ESM.zip › Figure 5/A/TUMOR IMAGE.tif]

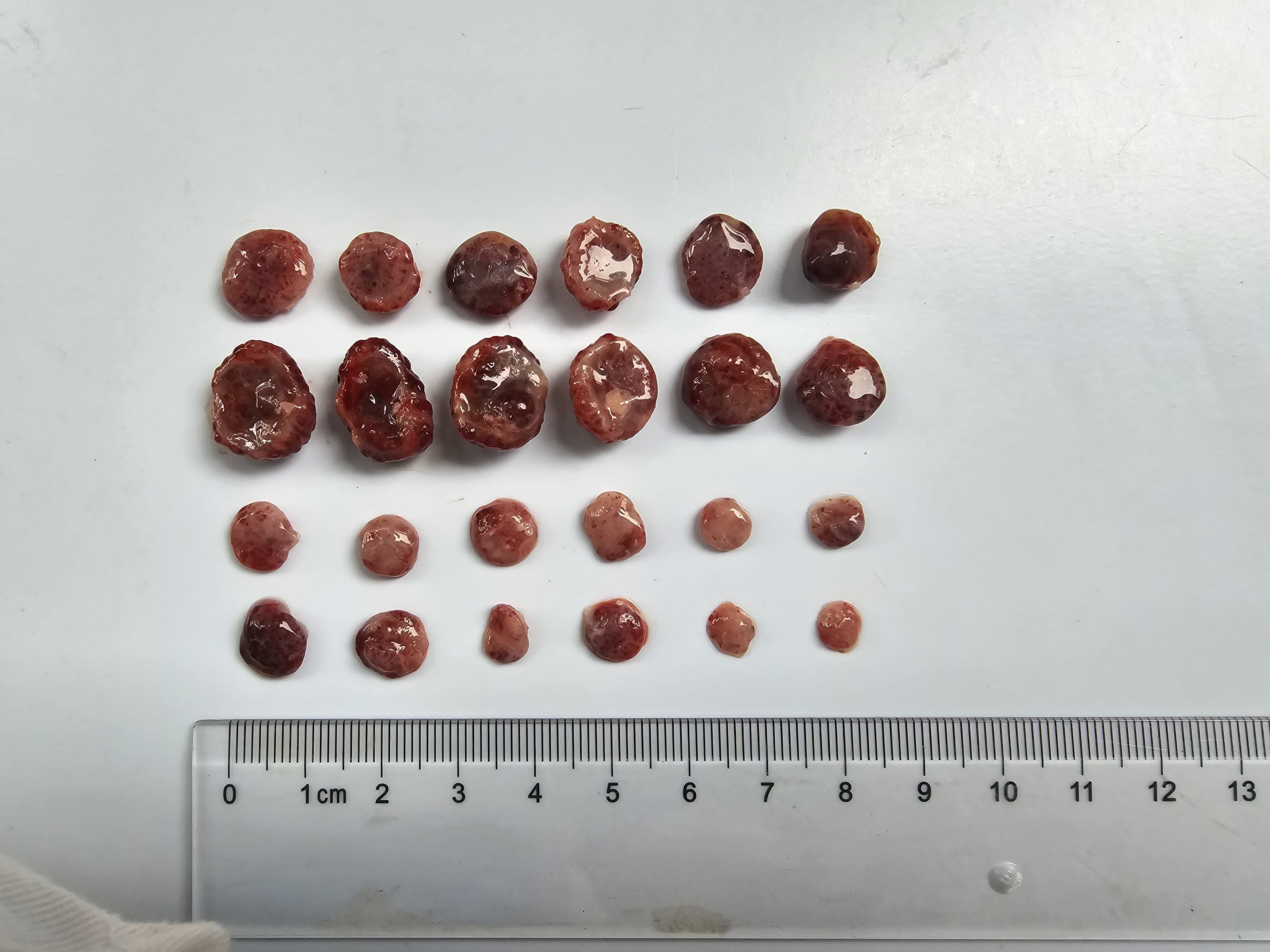

Supplement: Supplementary file 7 — Source data Fig. 5 [file 44319_2026_829_MOESM7_ESM.zip › Figure 5/D/TUMOR IMAGE.tif]

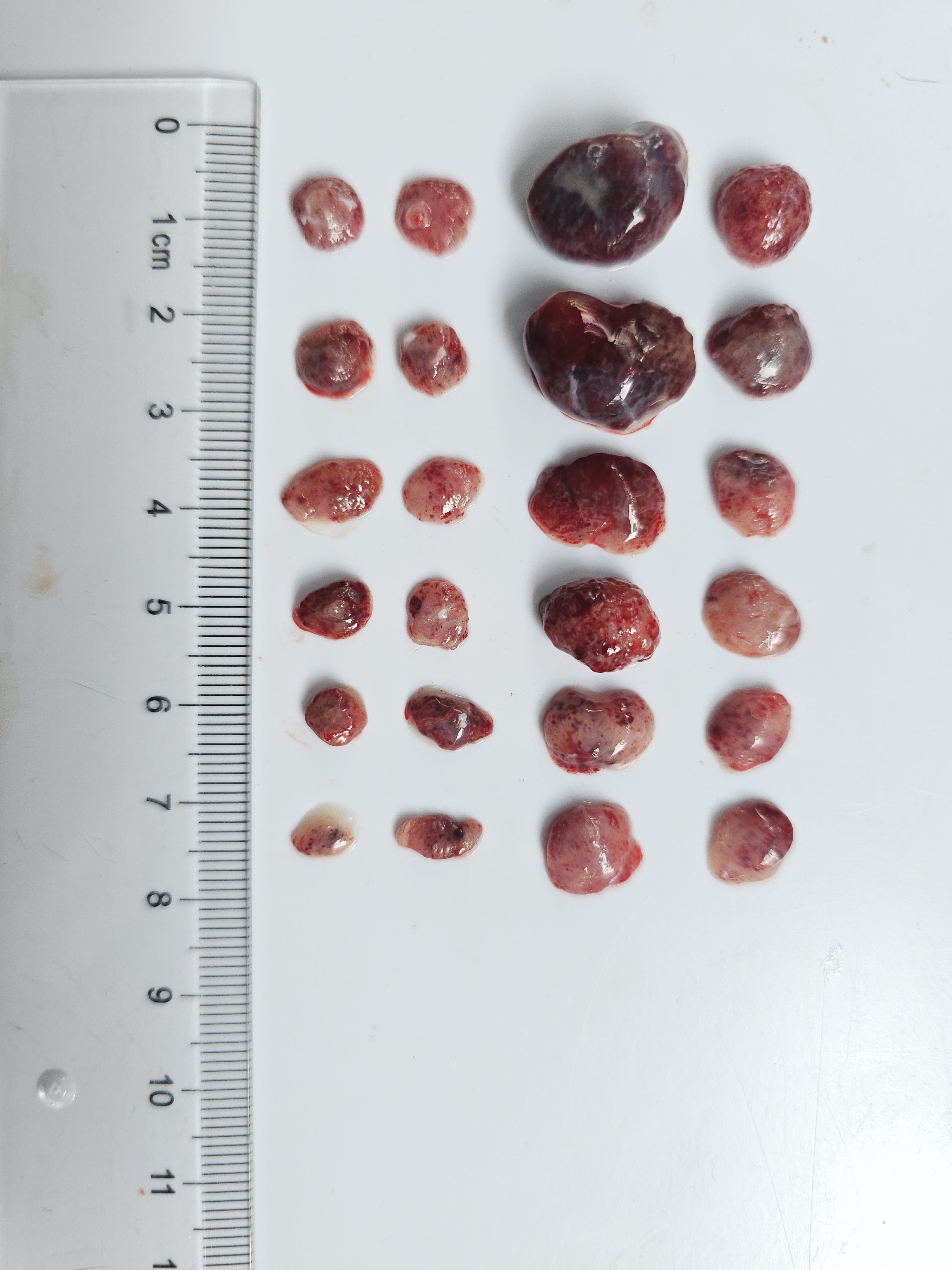

Supplement: Supplementary file 7 — Source data Fig. 5 [file 44319_2026_829_MOESM7_ESM.zip › Figure 5/E/TUMOR IMAGE.jpg]

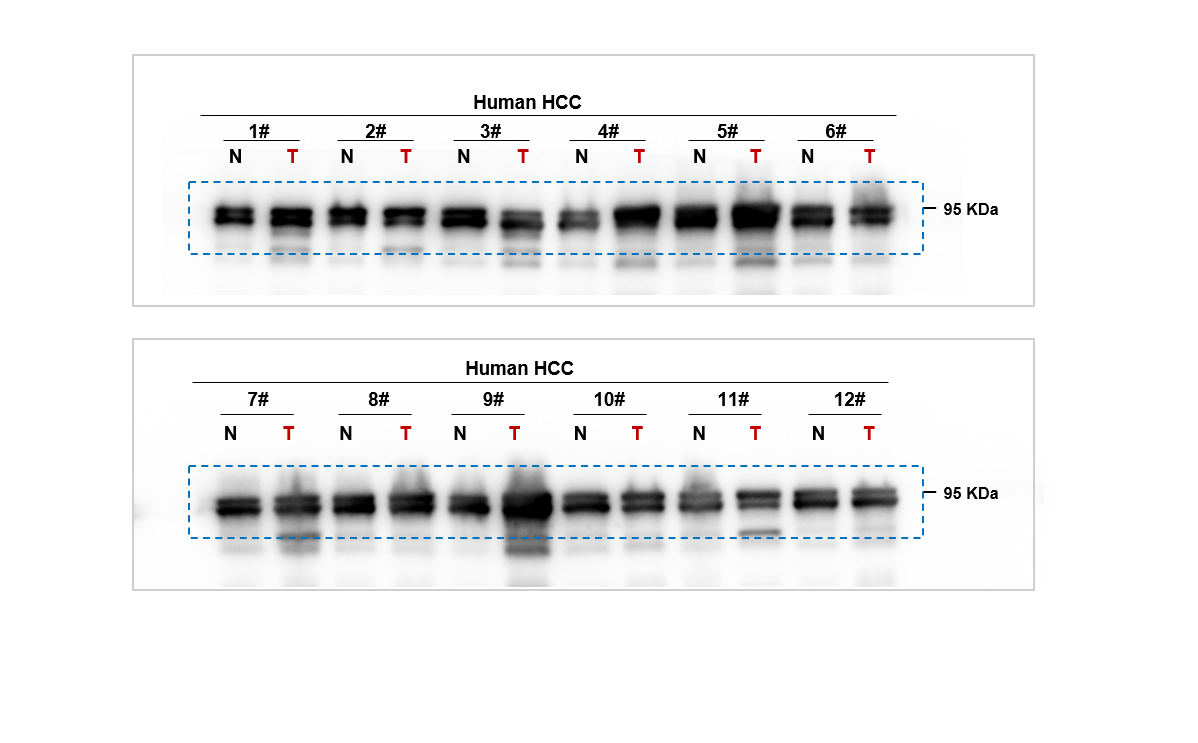

Supplement: Supplementary file 7 — Source data Fig. 5 [file 44319_2026_829_MOESM7_ESM.zip › Figure 5/F/Calnexin.png]

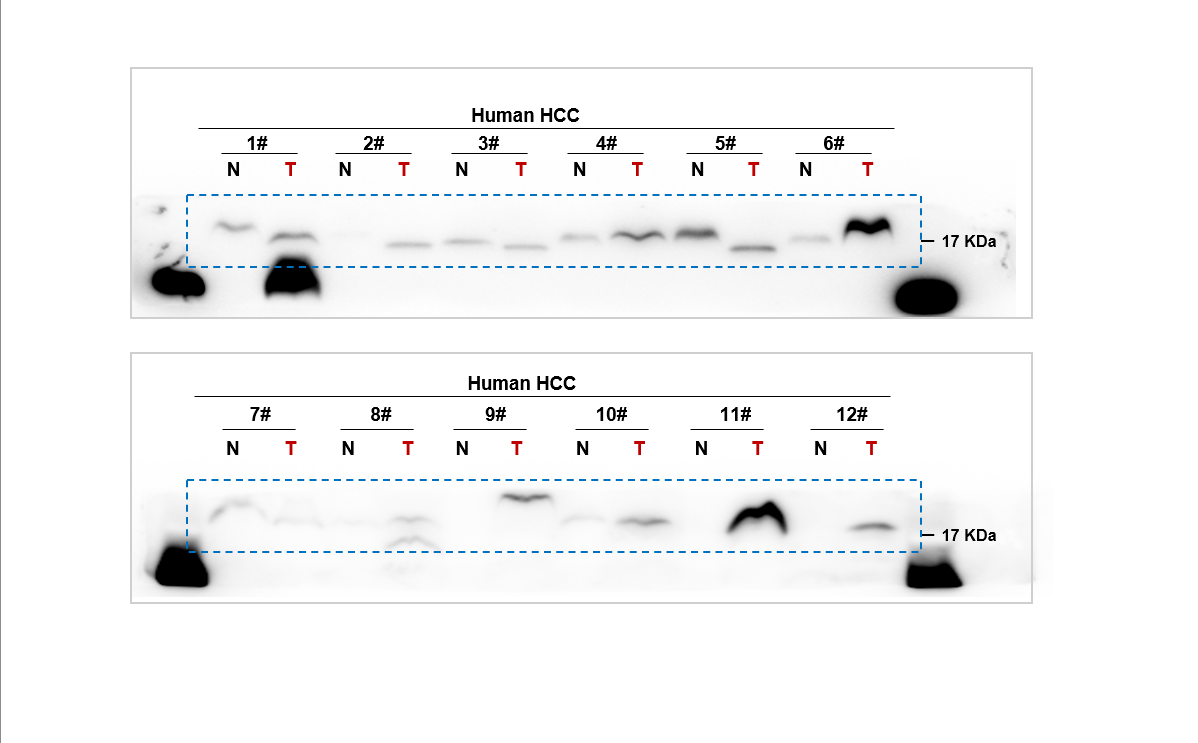

Supplement: Supplementary file 7 — Source data Fig. 5 [file 44319_2026_829_MOESM7_ESM.zip › Figure 5/F/H3.png]

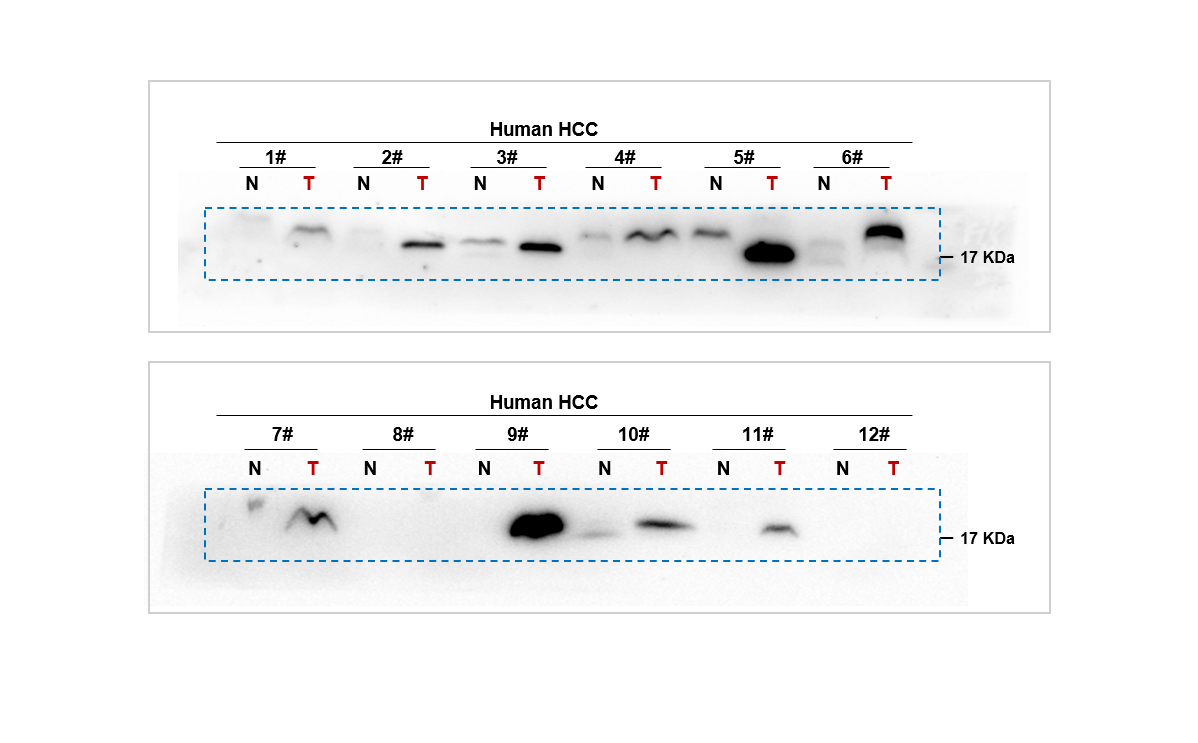

Supplement: Supplementary file 7 — Source data Fig. 5 [file 44319_2026_829_MOESM7_ESM.zip › Figure 5/F/H3K18la.png]

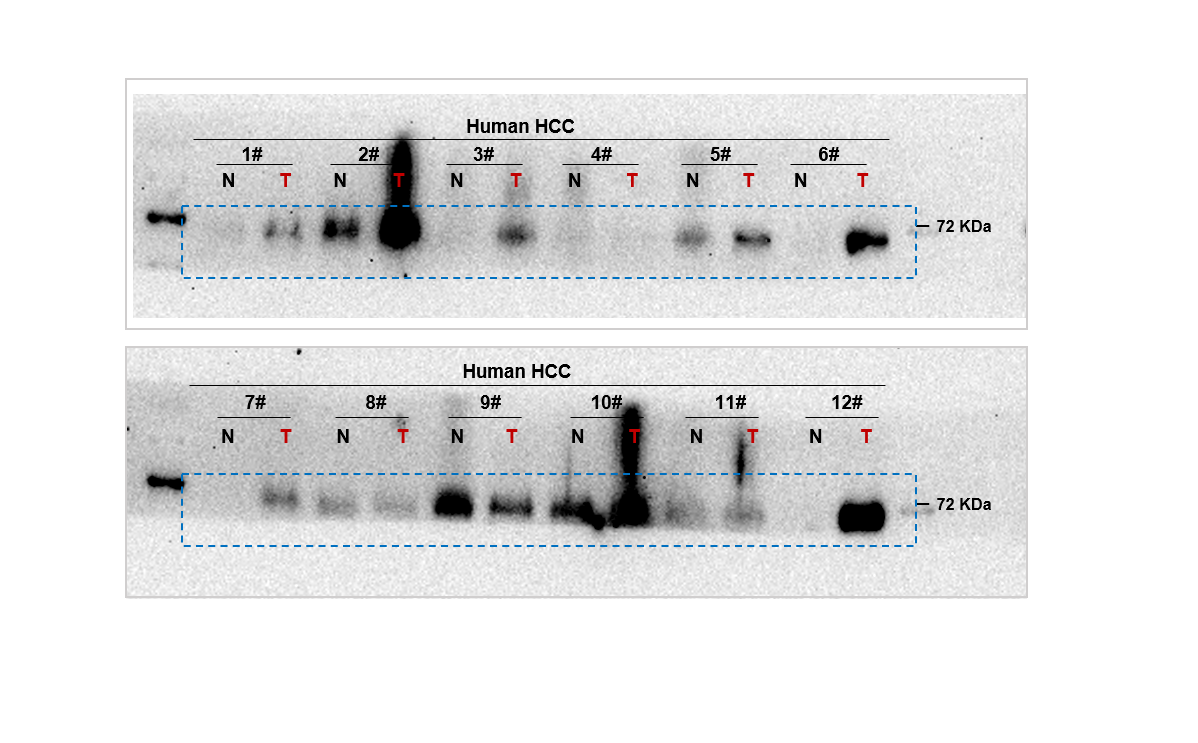

Supplement: Supplementary file 7 — Source data Fig. 5 [file 44319_2026_829_MOESM7_ESM.zip › Figure 5/F/p-S6K.png]

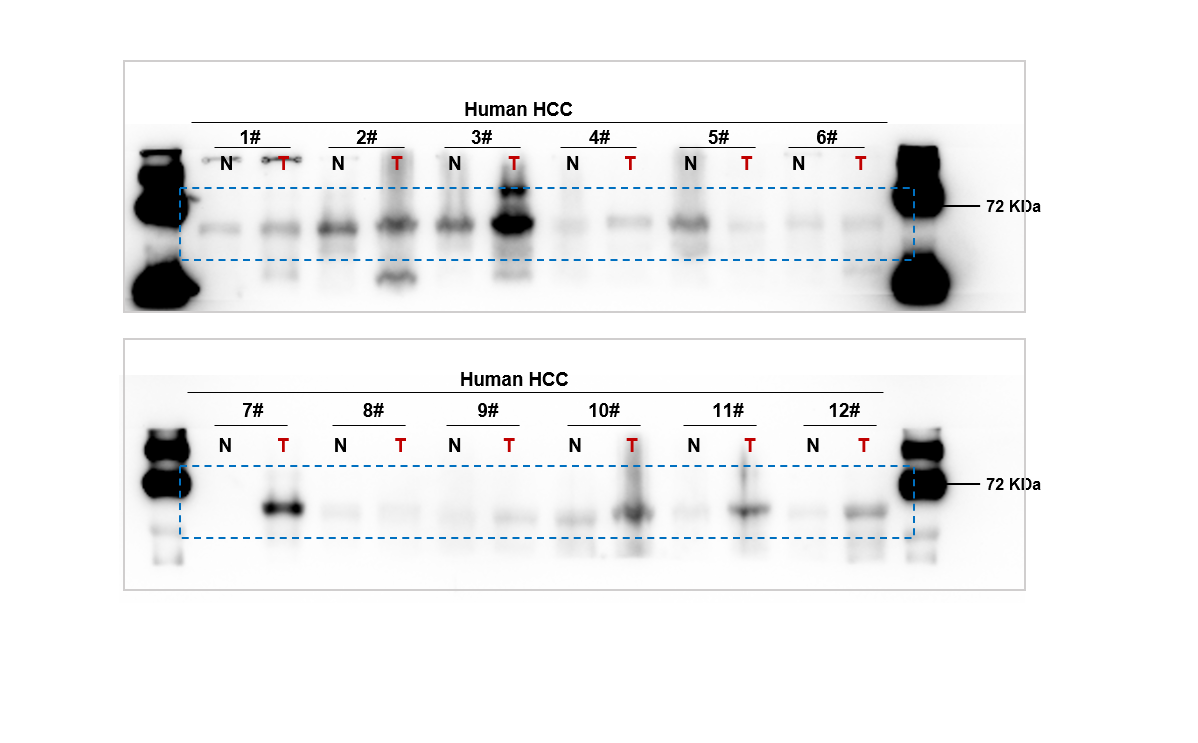

Supplement: Supplementary file 7 — Source data Fig. 5 [file 44319_2026_829_MOESM7_ESM.zip › Figure 5/F/S6K.png]

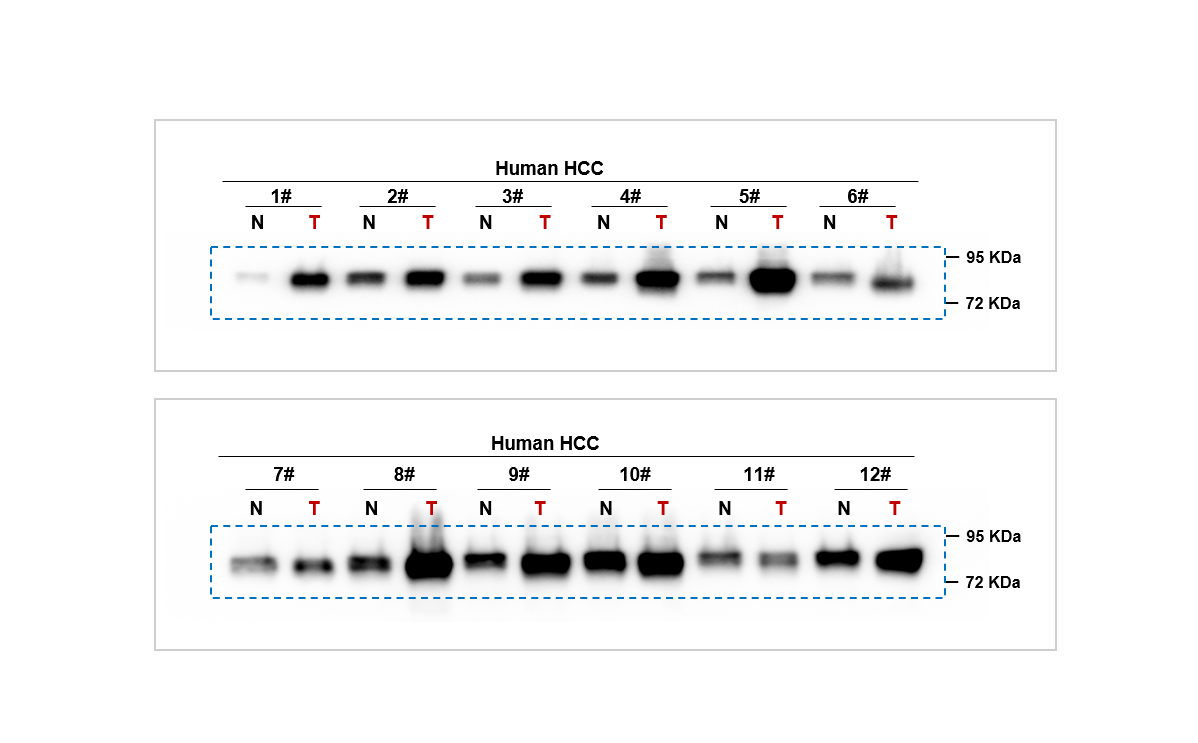

Supplement: Supplementary file 7 — Source data Fig. 5 [file 44319_2026_829_MOESM7_ESM.zip › Figure 5/F/SCARB1.png]

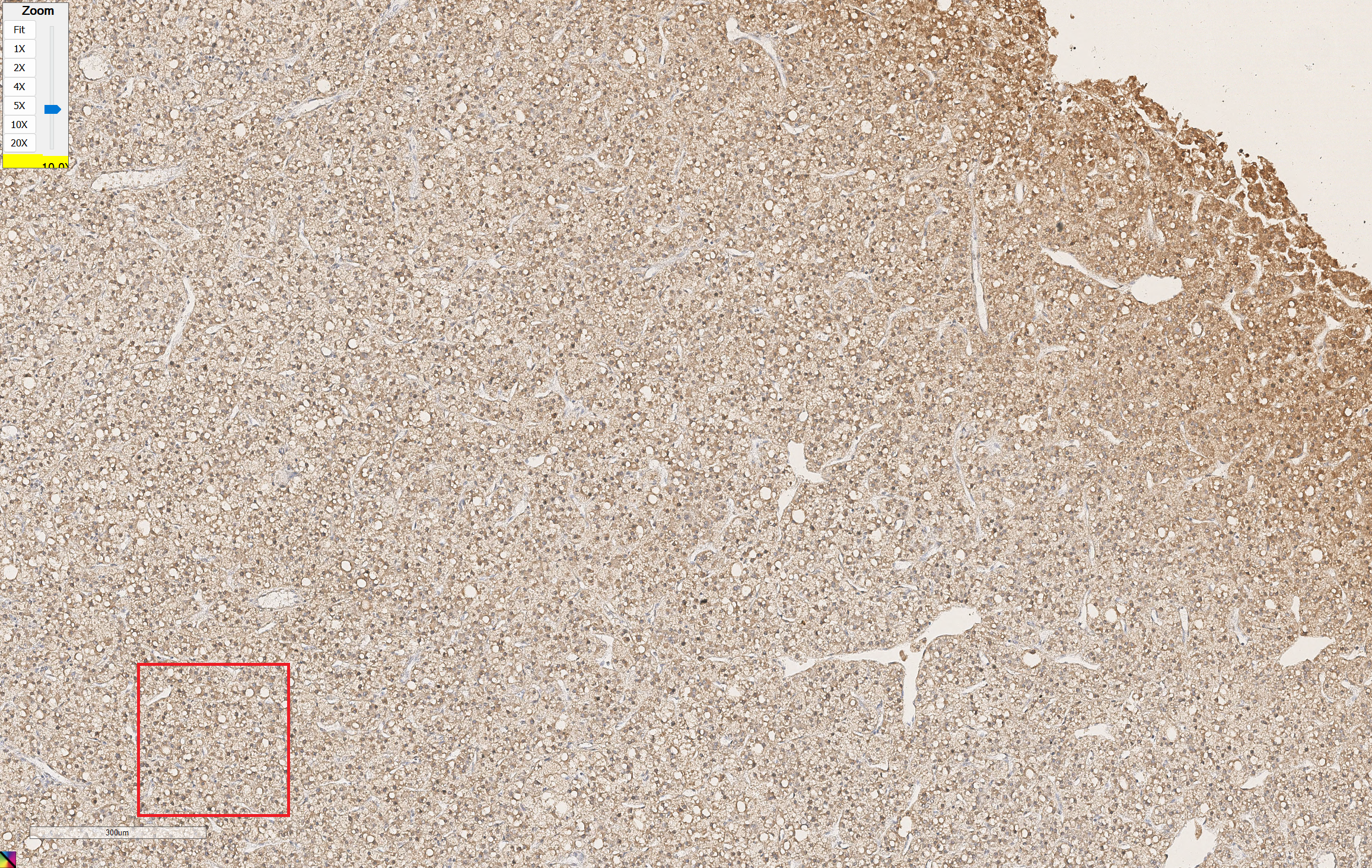

Supplement: Supplementary file 7 — Source data Fig. 5 [file 44319_2026_829_MOESM7_ESM.zip › Figure 5/G/IHC/High-H3K18la-#1.tif]

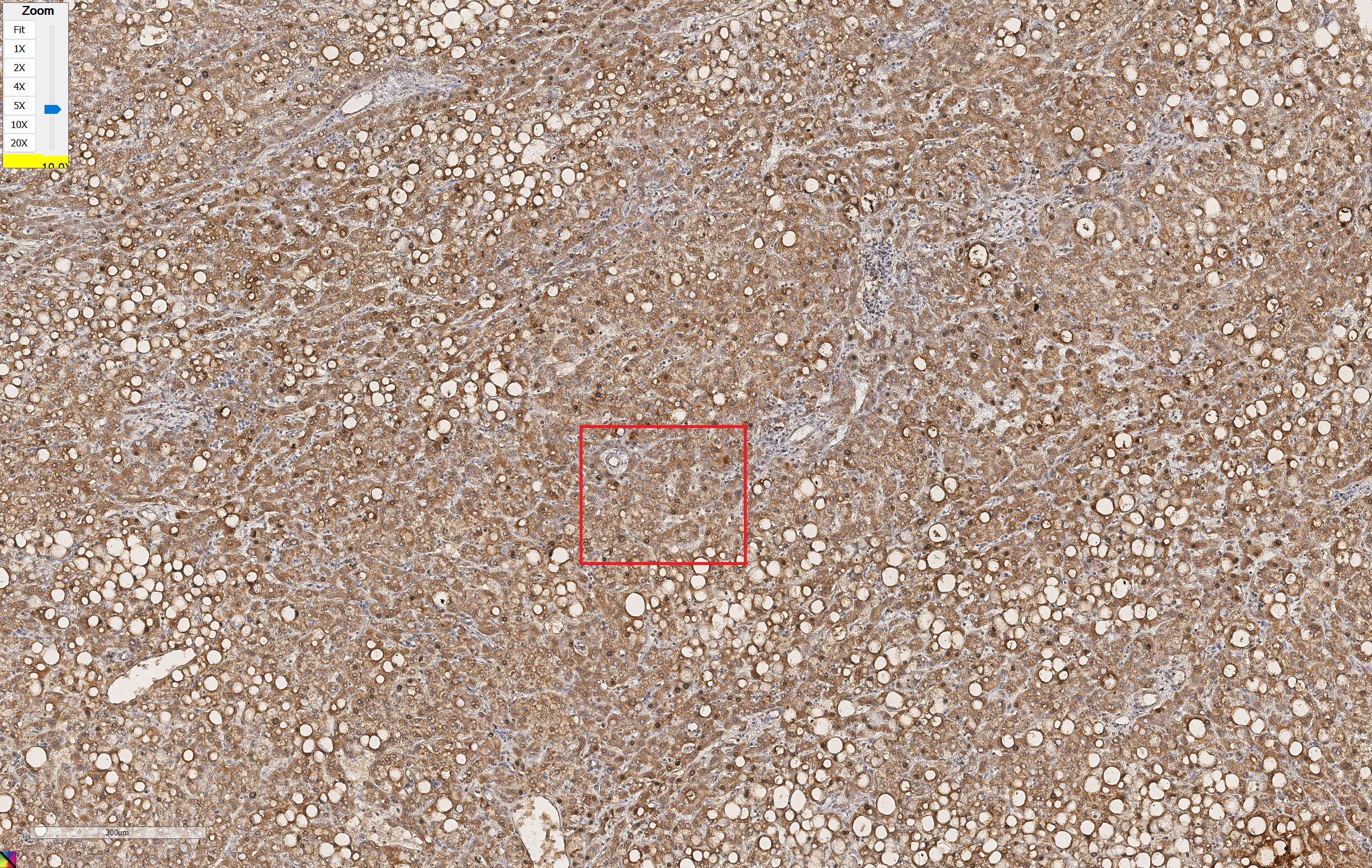

Supplement: Supplementary file 7 — Source data Fig. 5 [file 44319_2026_829_MOESM7_ESM.zip › Figure 5/G/IHC/High-H3K18la-#2.tif]

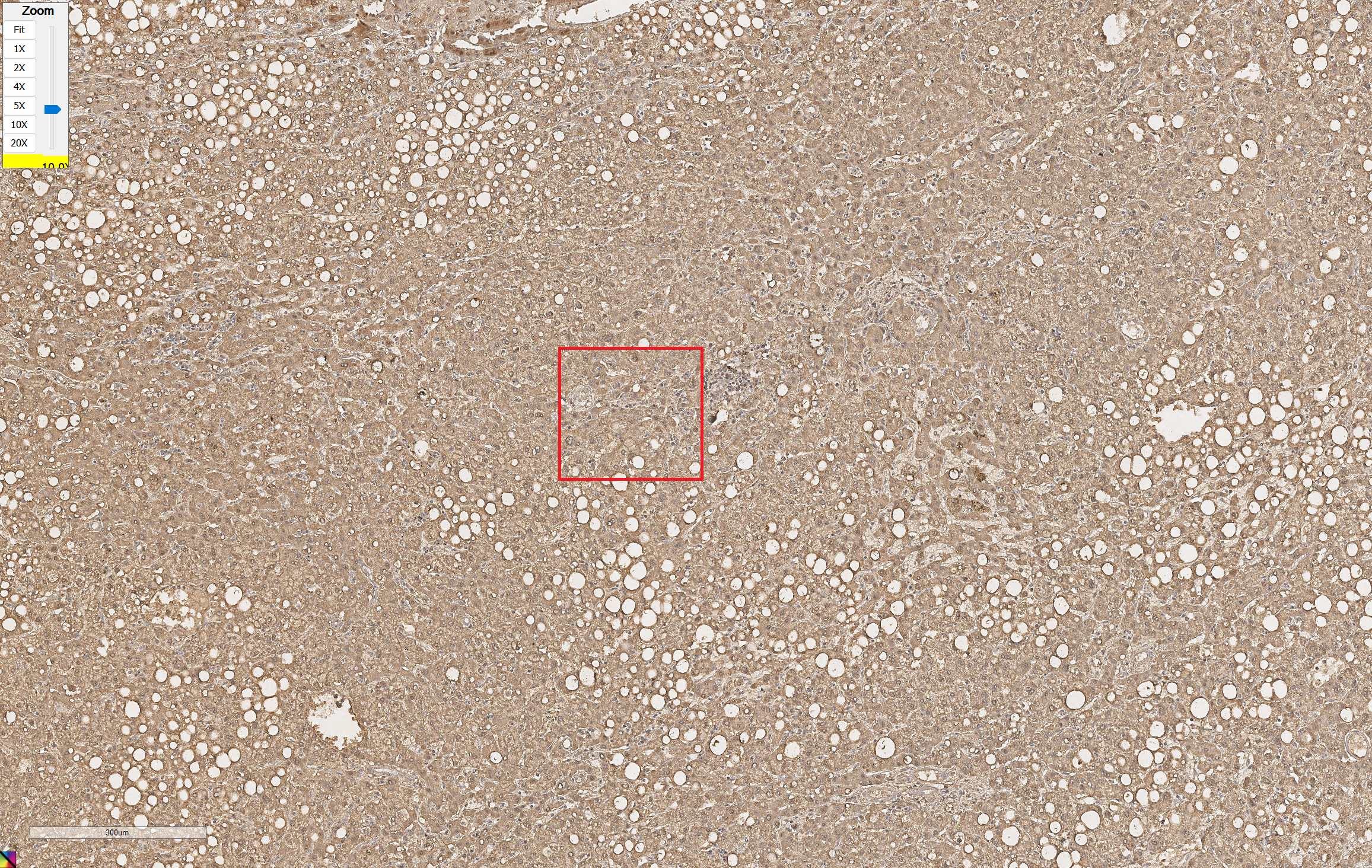

Supplement: Supplementary file 7 — Source data Fig. 5 [file 44319_2026_829_MOESM7_ESM.zip › Figure 5/G/IHC/High-LDHA-#2.tif]

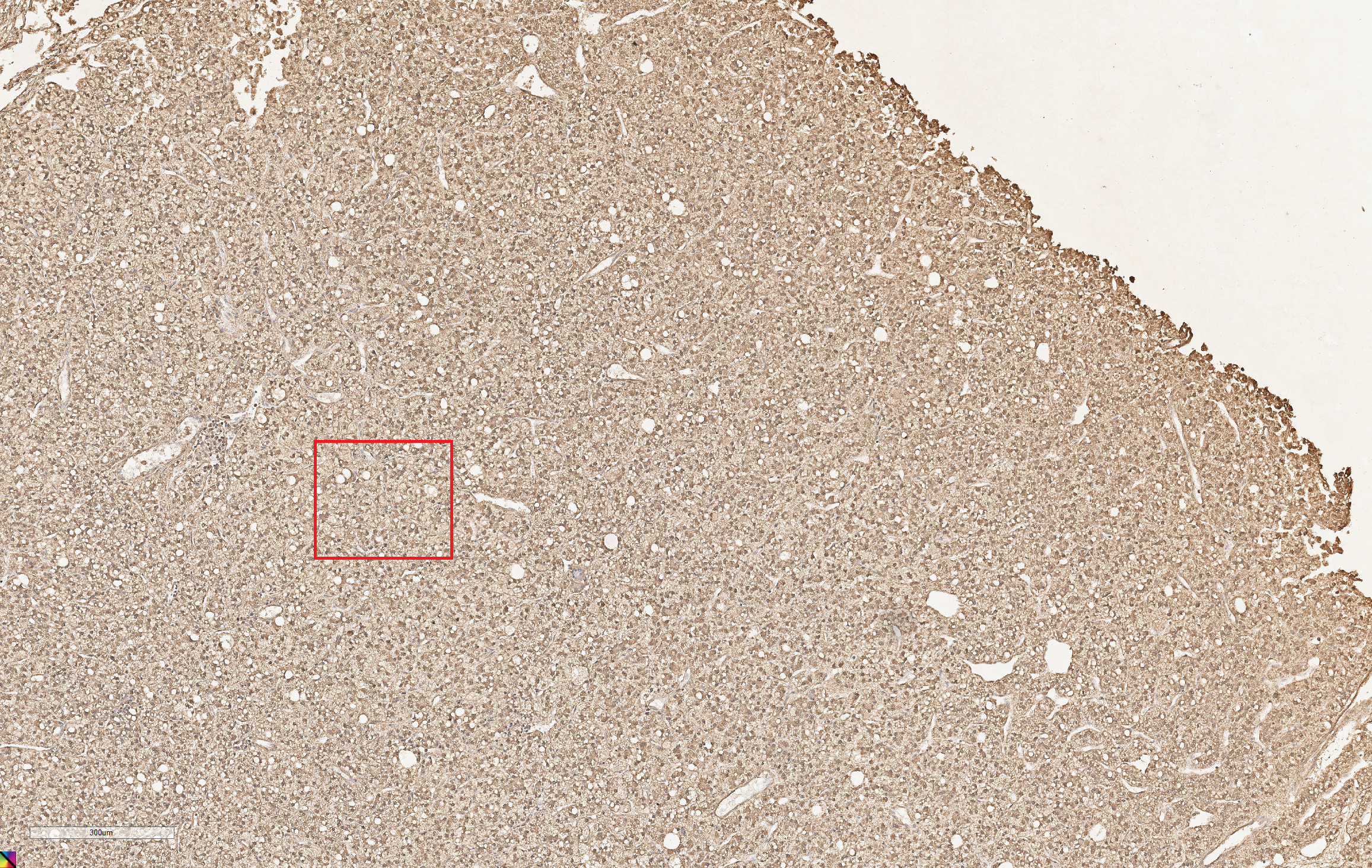

Supplement: Supplementary file 7 — Source data Fig. 5 [file 44319_2026_829_MOESM7_ESM.zip › Figure 5/G/IHC/High-LDHAla-#1.tif]

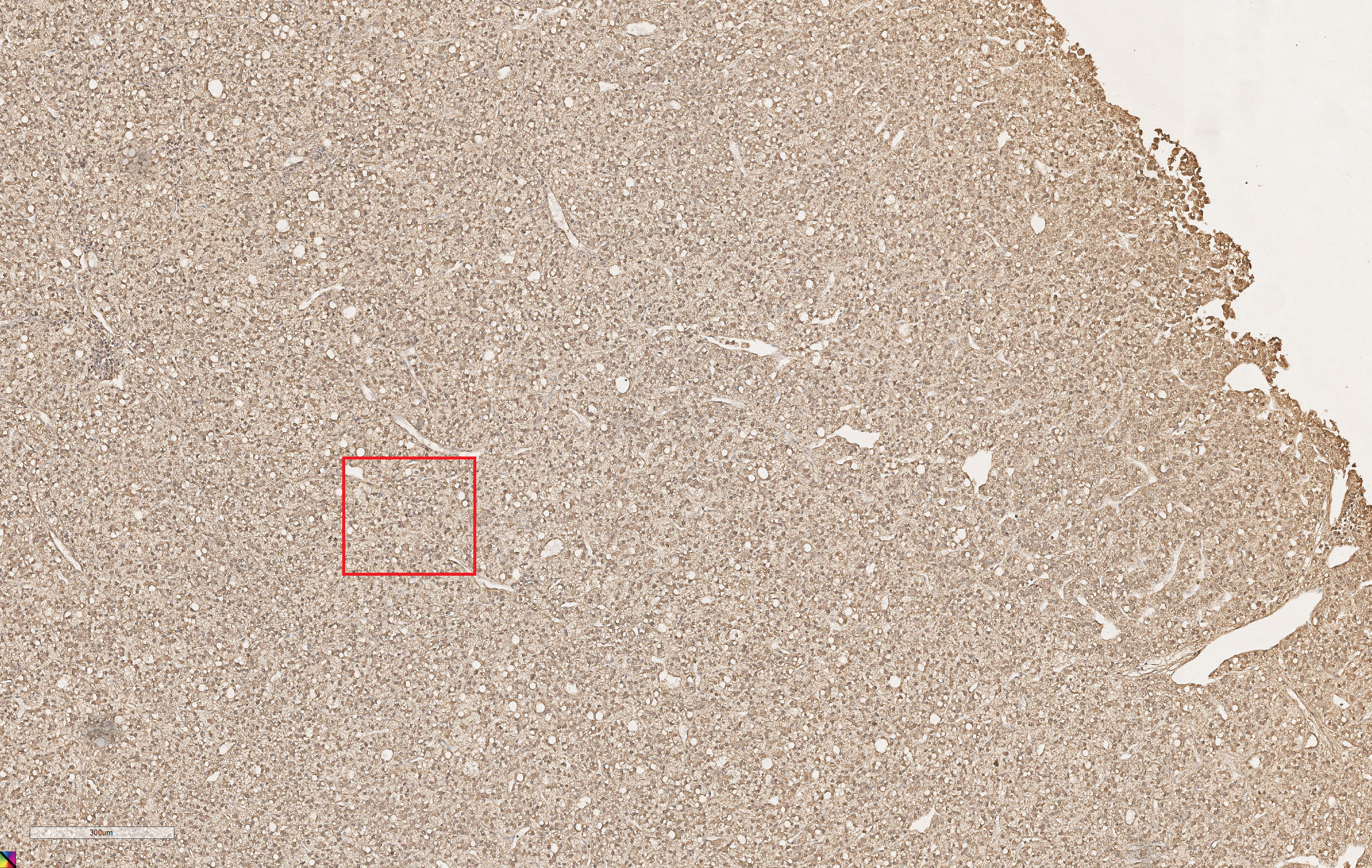

Supplement: Supplementary file 7 — Source data Fig. 5 [file 44319_2026_829_MOESM7_ESM.zip › Figure 5/G/IHC/High-p-S6K-#1.tif]

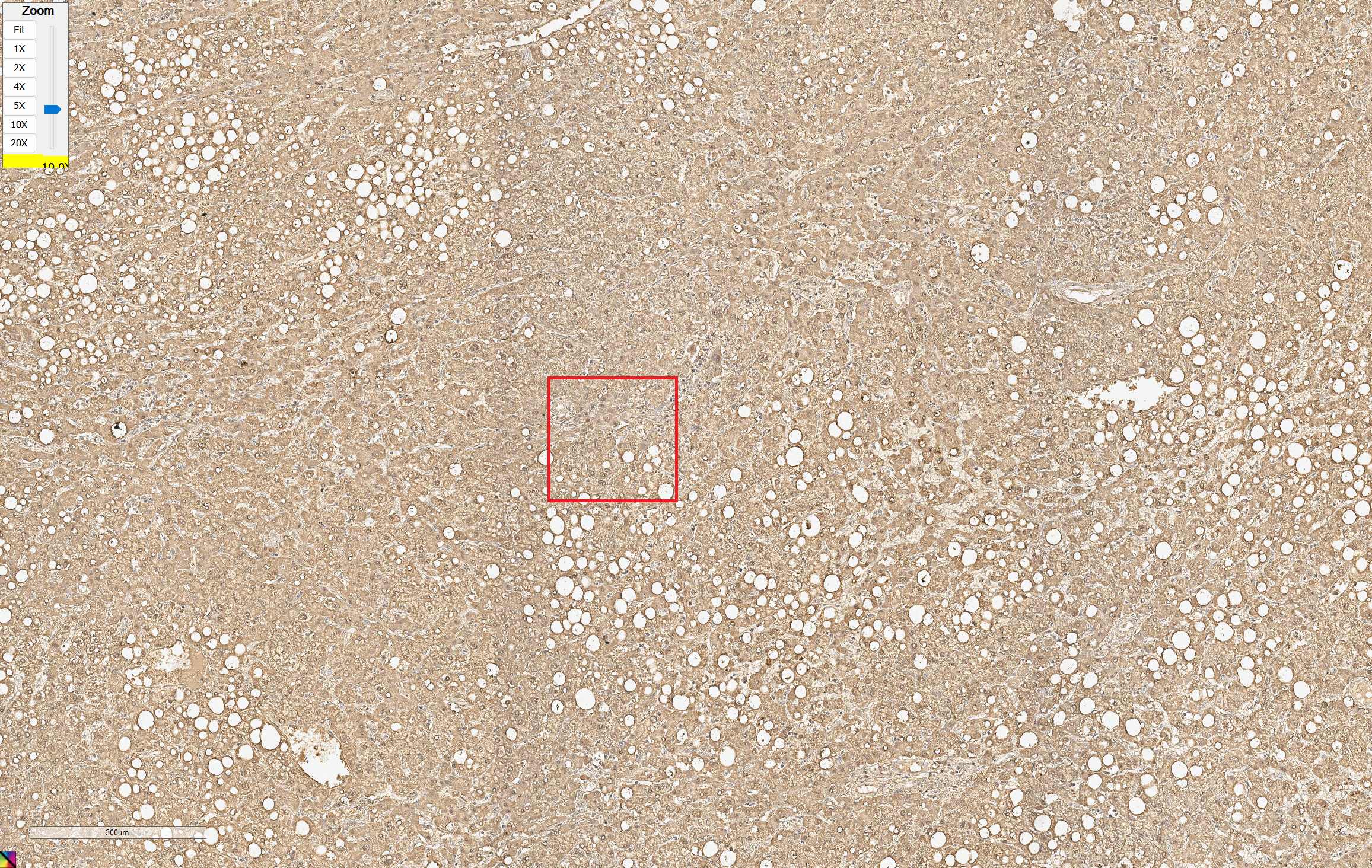

Supplement: Supplementary file 7 — Source data Fig. 5 [file 44319_2026_829_MOESM7_ESM.zip › Figure 5/G/IHC/High-p-S6K-#2.tif]

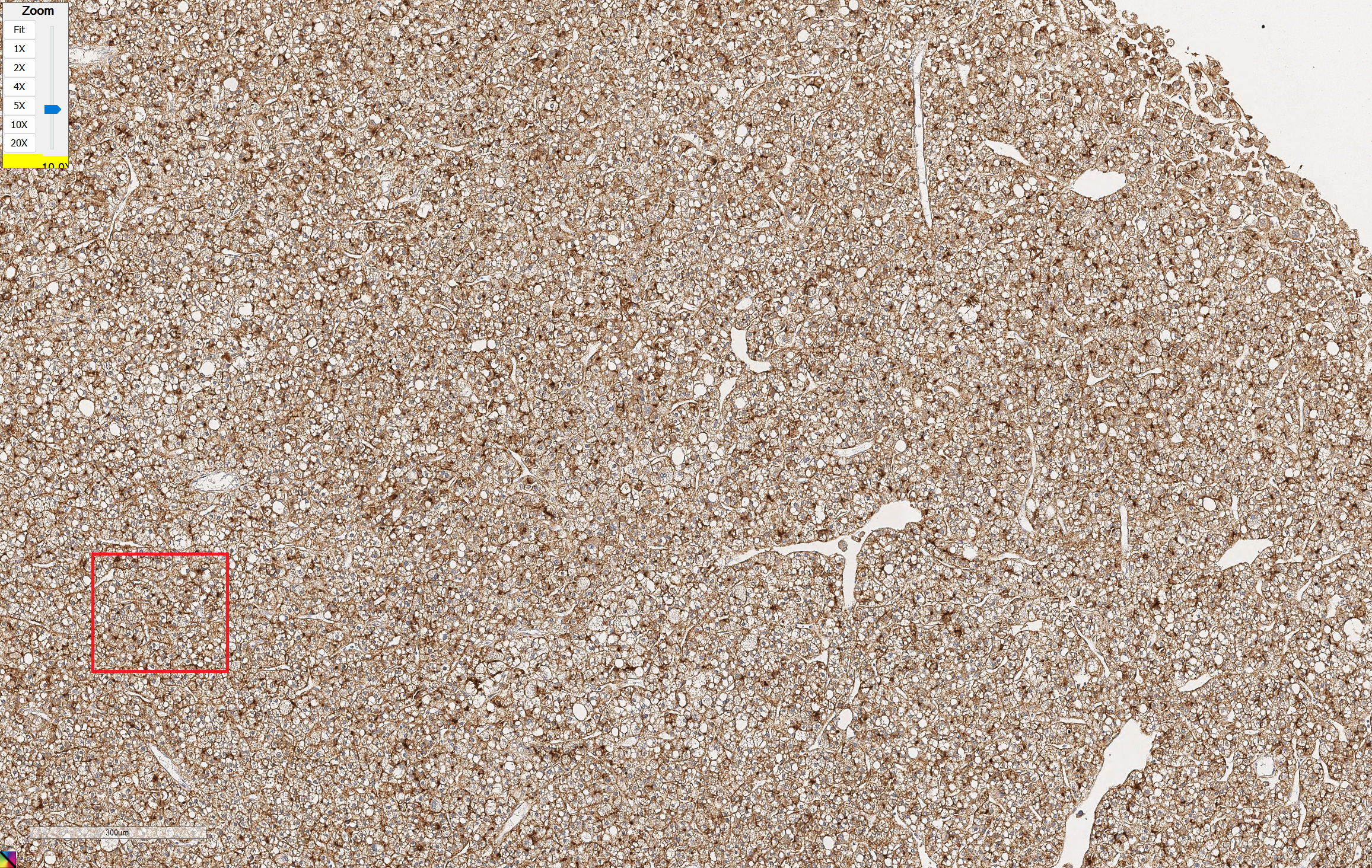

Supplement: Supplementary file 7 — Source data Fig. 5 [file 44319_2026_829_MOESM7_ESM.zip › Figure 5/G/IHC/High-SCARB1-#1.tif]

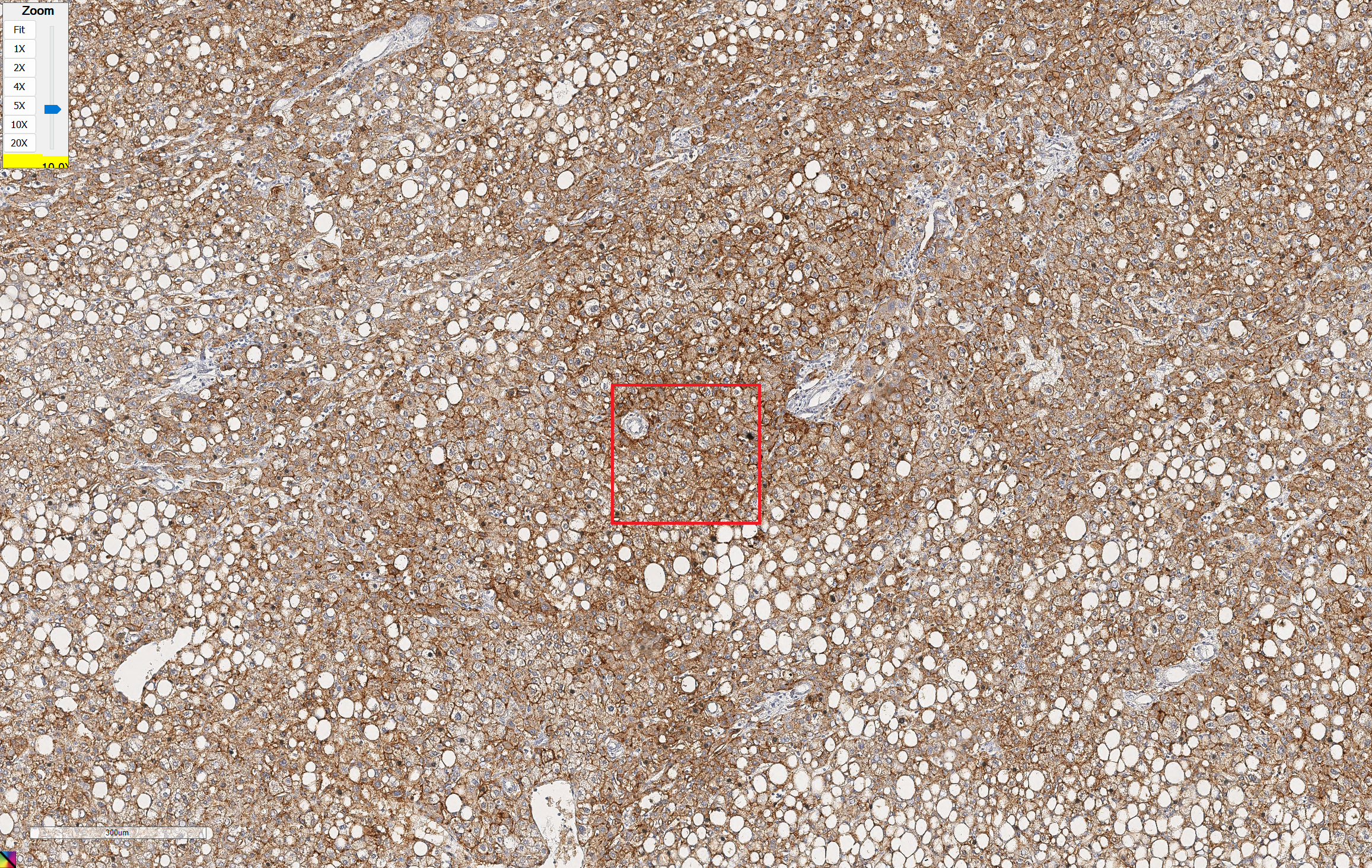

Supplement: Supplementary file 7 — Source data Fig. 5 [file 44319_2026_829_MOESM7_ESM.zip › Figure 5/G/IHC/High-SCARB1-#2.tif]

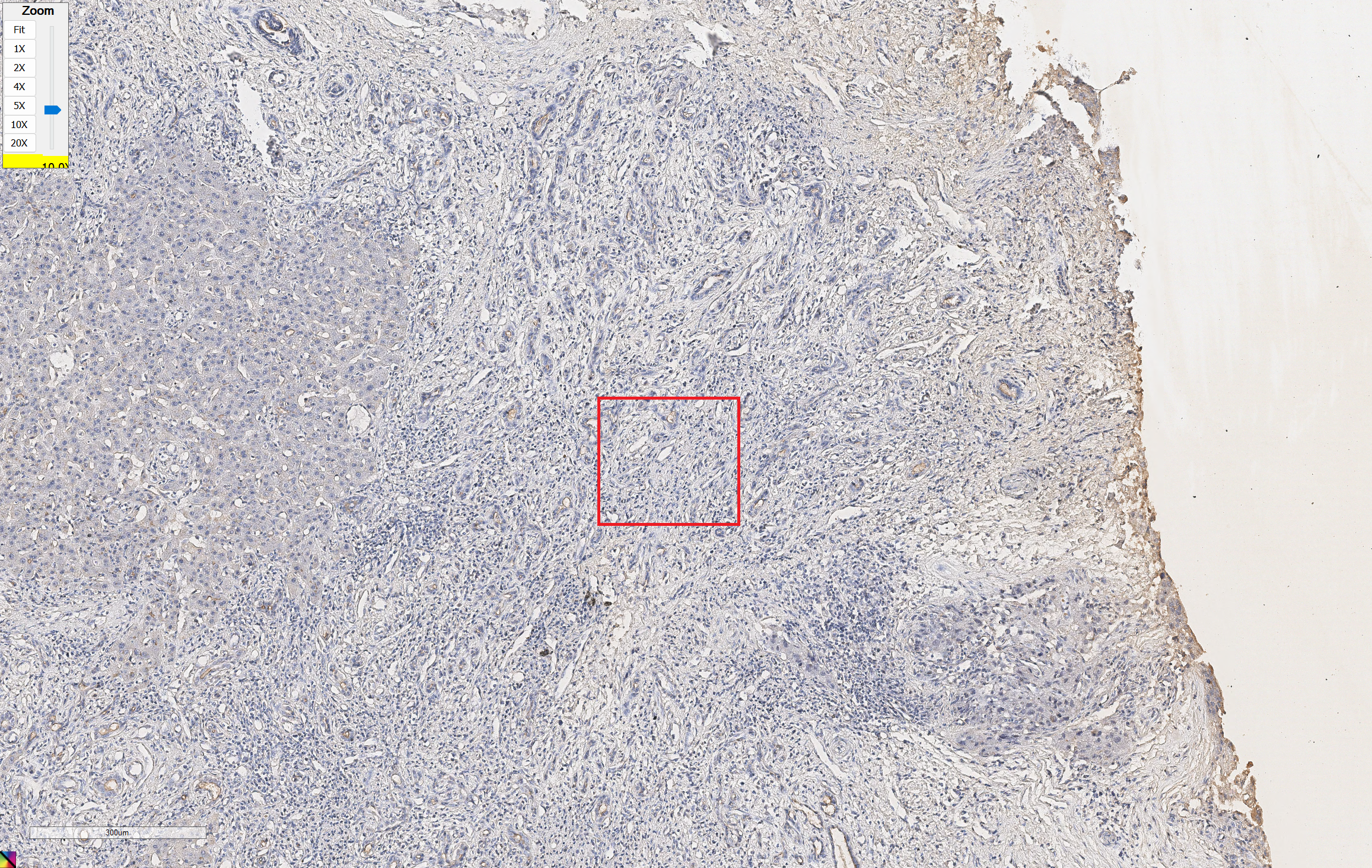

Supplement: Supplementary file 7 — Source data Fig. 5 [file 44319_2026_829_MOESM7_ESM.zip › Figure 5/G/IHC/Low-H3K18la-#1.tif]

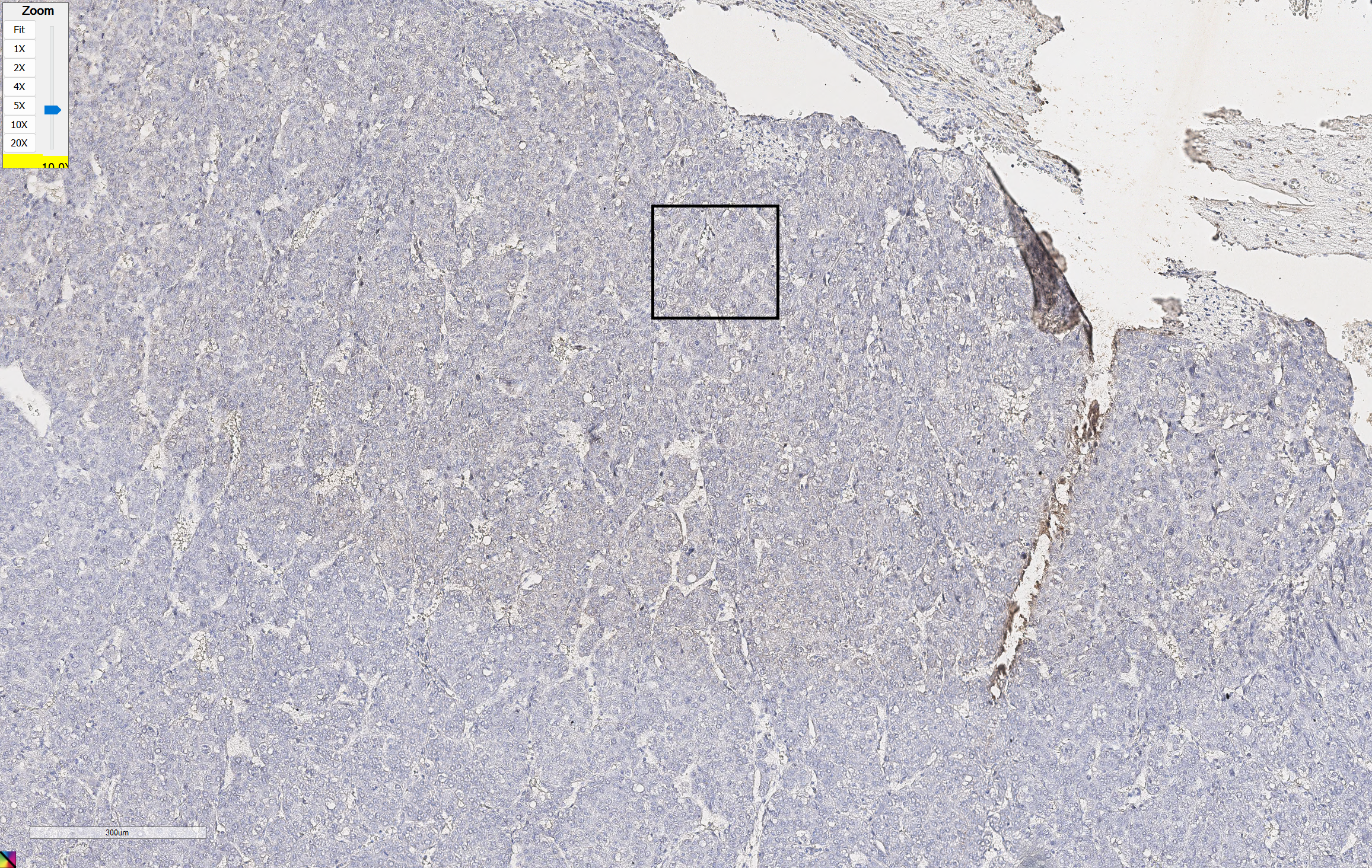

Supplement: Supplementary file 7 — Source data Fig. 5 [file 44319_2026_829_MOESM7_ESM.zip › Figure 5/G/IHC/Low-H3K18la-#2.tif]

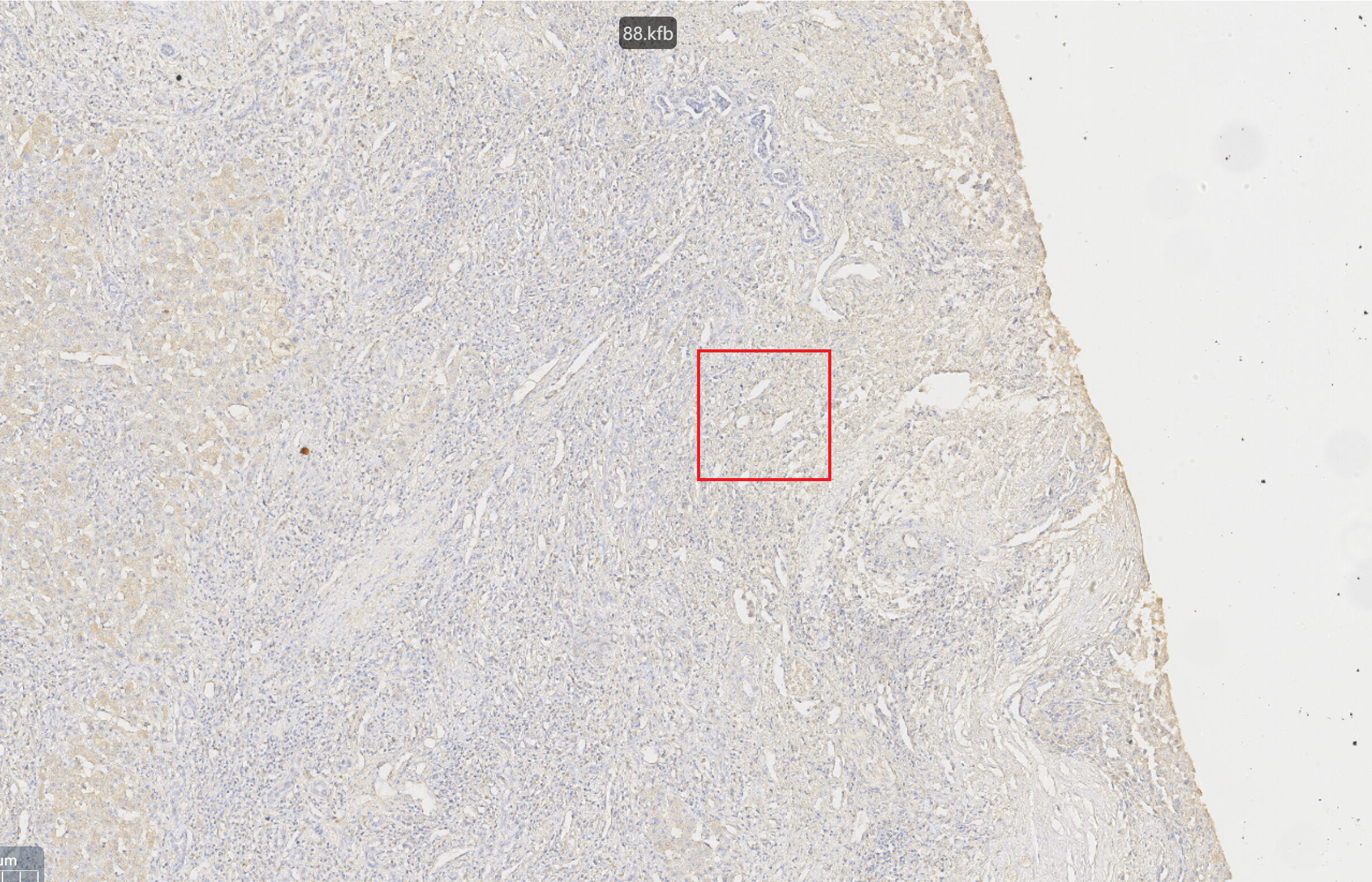

Supplement: Supplementary file 7 — Source data Fig. 5 [file 44319_2026_829_MOESM7_ESM.zip › Figure 5/G/IHC/Low-LDHA-#1.tif]

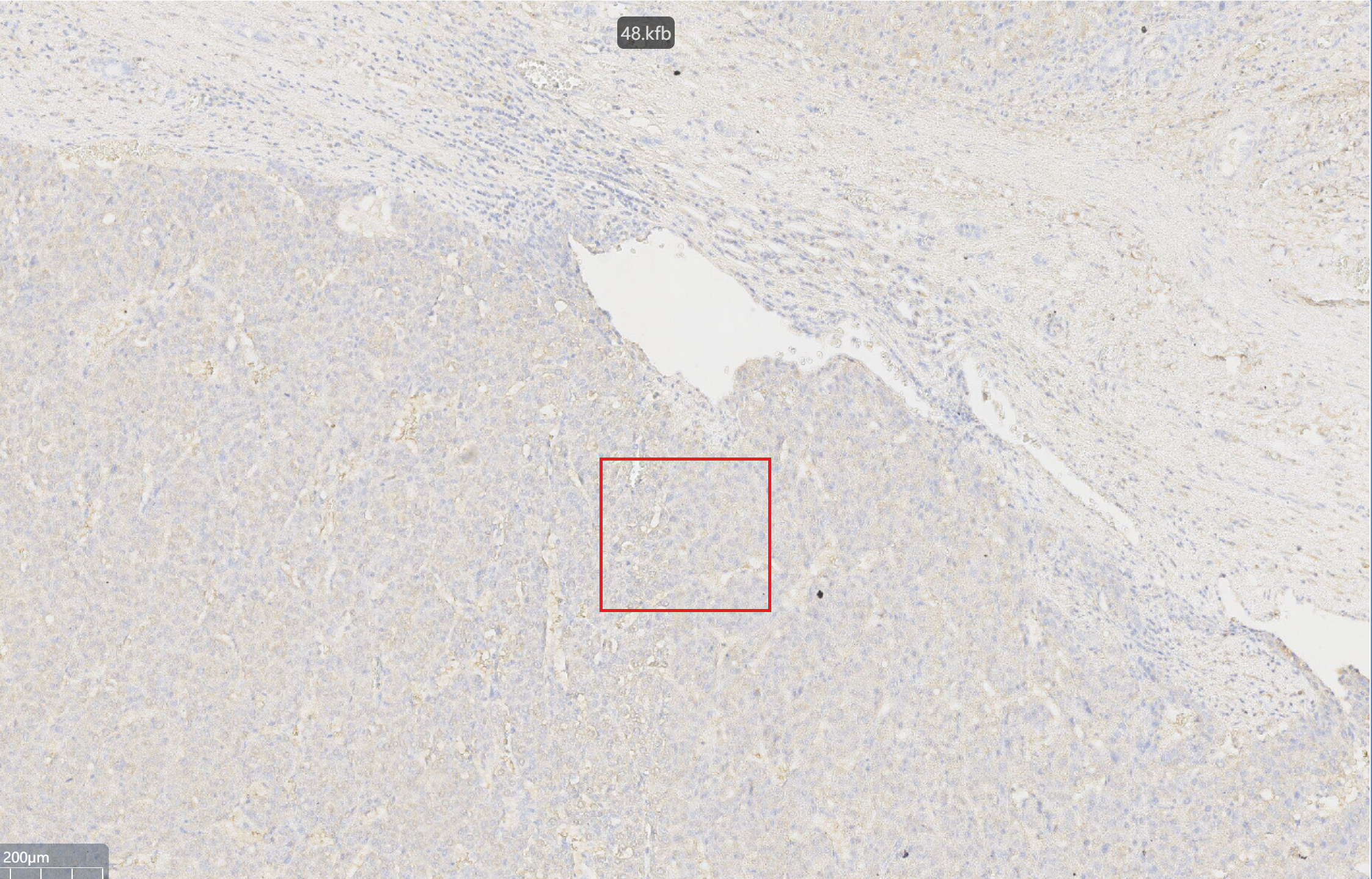

Supplement: Supplementary file 7 — Source data Fig. 5 [file 44319_2026_829_MOESM7_ESM.zip › Figure 5/G/IHC/Low-LDHA-#2.tif]

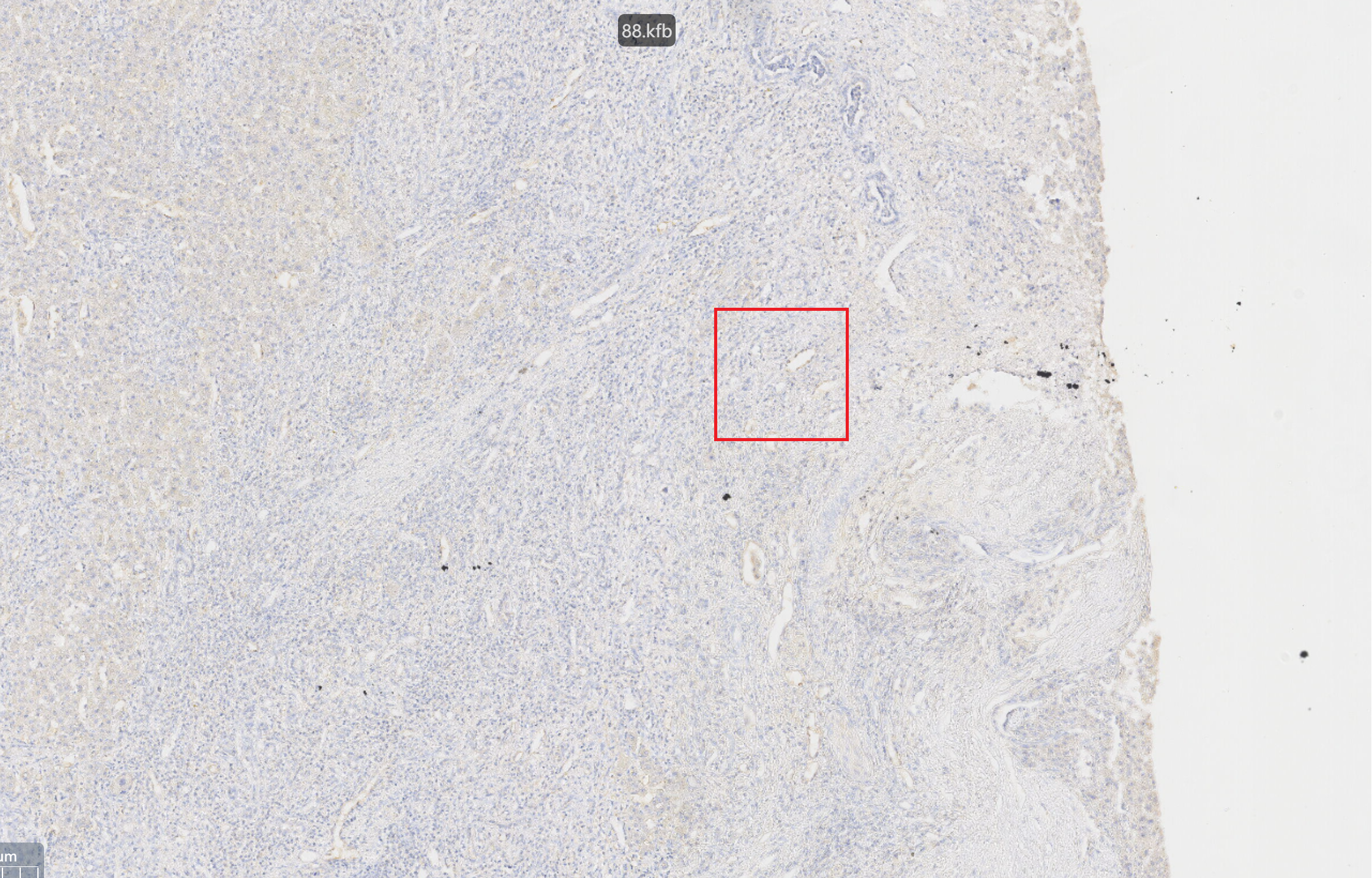

Supplement: Supplementary file 7 — Source data Fig. 5 [file 44319_2026_829_MOESM7_ESM.zip › Figure 5/G/IHC/Low-p-S6K-#1.tif]

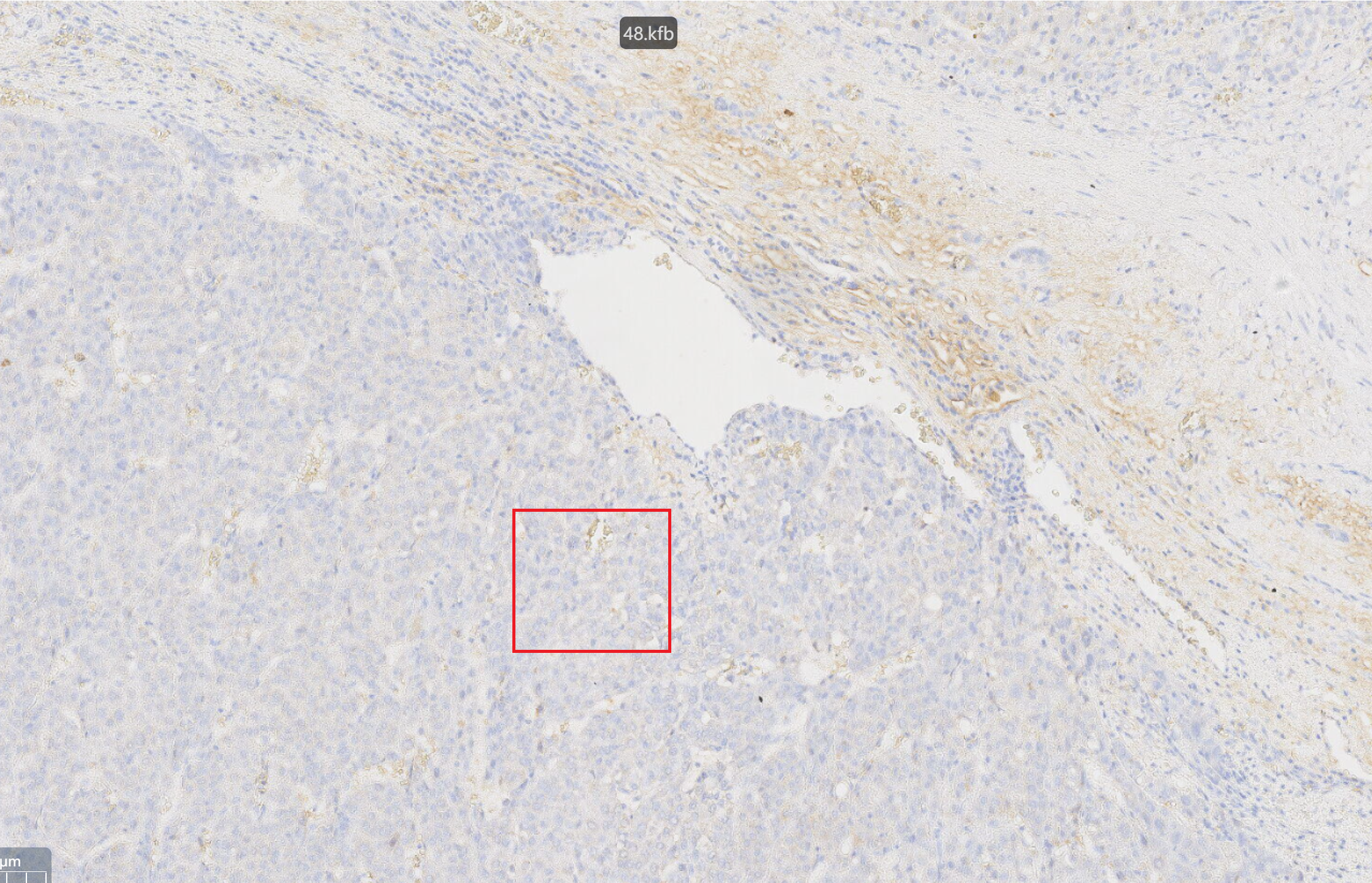

Supplement: Supplementary file 7 — Source data Fig. 5 [file 44319_2026_829_MOESM7_ESM.zip › Figure 5/G/IHC/Low-p-S6K-#2.tif]

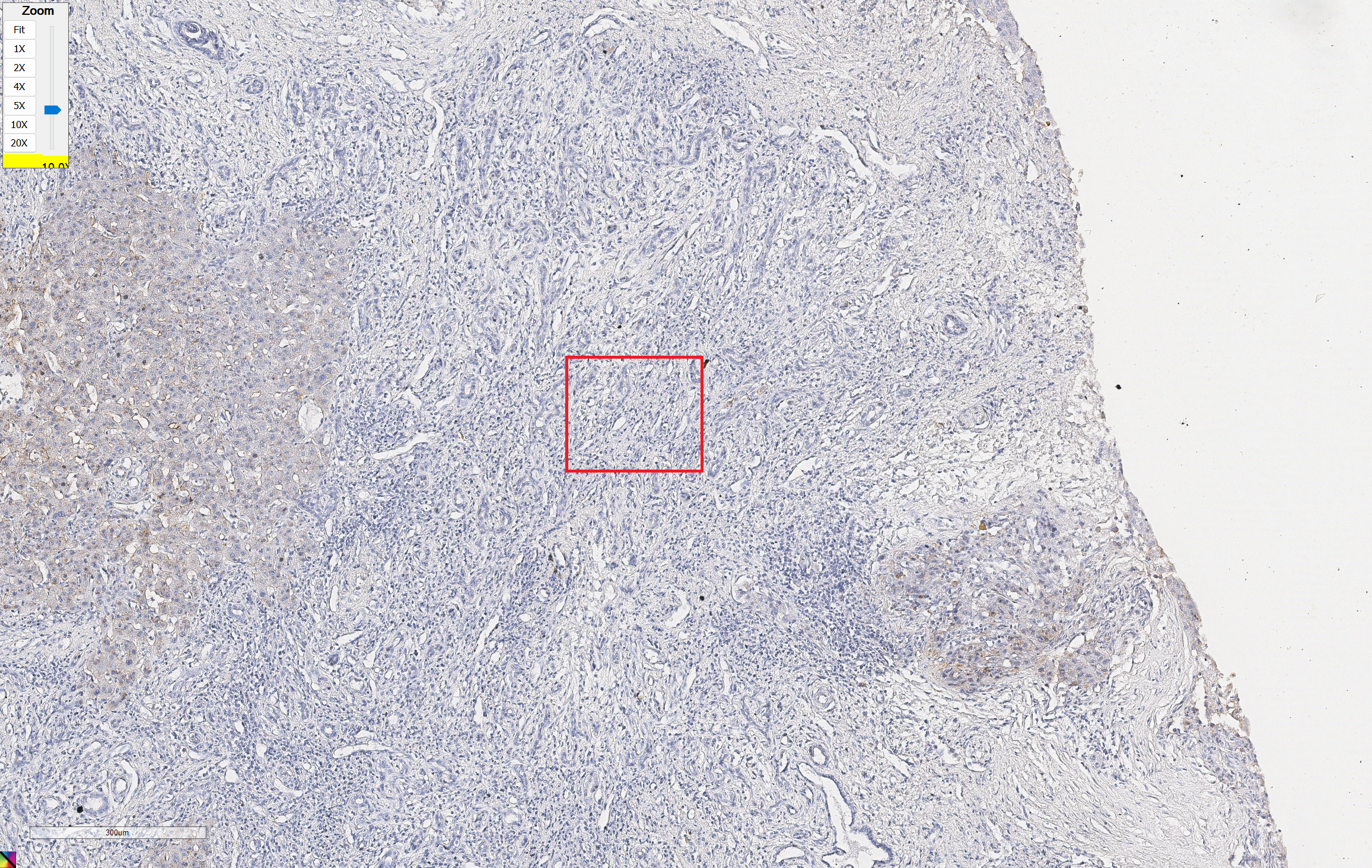

Supplement: Supplementary file 7 — Source data Fig. 5 [file 44319_2026_829_MOESM7_ESM.zip › Figure 5/G/IHC/Low-SCARB1-#1.tif]

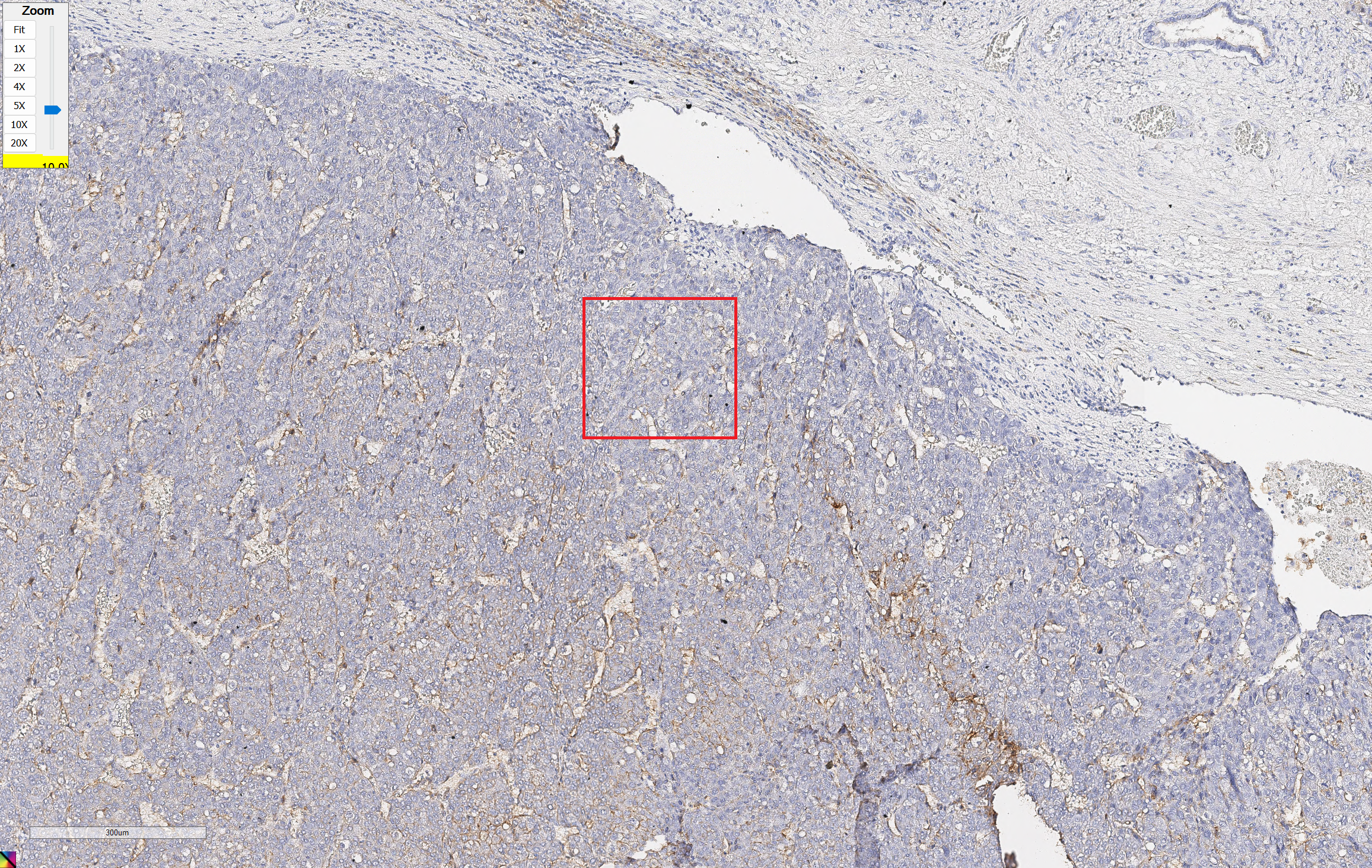

Supplement: Supplementary file 7 — Source data Fig. 5 [file 44319_2026_829_MOESM7_ESM.zip › Figure 5/G/IHC/Low-SCARB1-#2.tif]

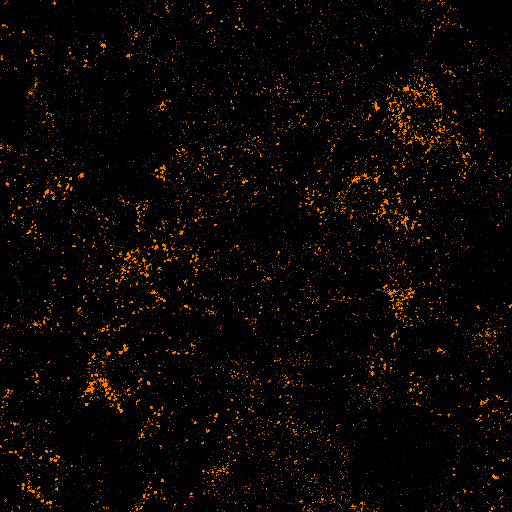

Supplement: Supplementary file 8 — Figure EV1 Source Data [file 44319_2026_829_MOESM8_ESM.zip › Figure EV1/EV 1/EV 1C/EV1C/DiI-HDL/H/DiI.tif]

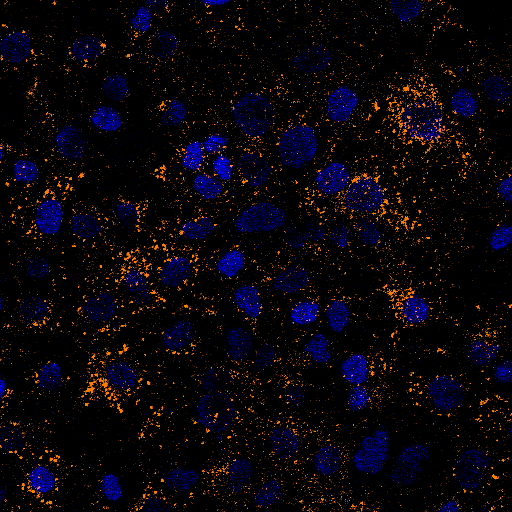

Supplement: Supplementary file 8 — Figure EV1 Source Data [file 44319_2026_829_MOESM8_ESM.zip › Figure EV1/EV 1/EV 1C/EV1C/DiI-HDL/H/MERGE.tif]

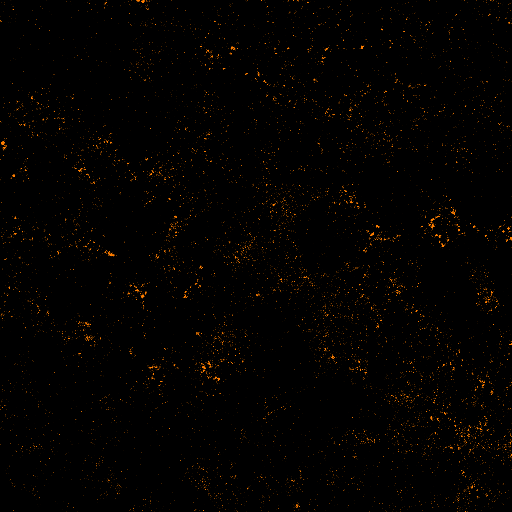

Supplement: Supplementary file 8 — Figure EV1 Source Data [file 44319_2026_829_MOESM8_ESM.zip › Figure EV1/EV 1/EV 1C/EV1C/DiI-HDL/H+SO/DiI.tif]

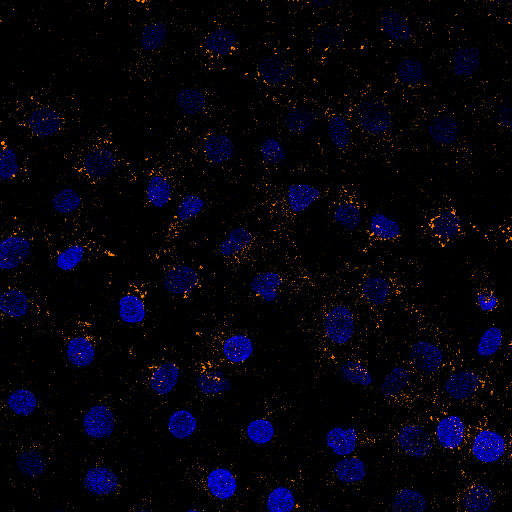

Supplement: Supplementary file 8 — Figure EV1 Source Data [file 44319_2026_829_MOESM8_ESM.zip › Figure EV1/EV 1/EV 1C/EV1C/DiI-HDL/H+SO/MERGE.tif]

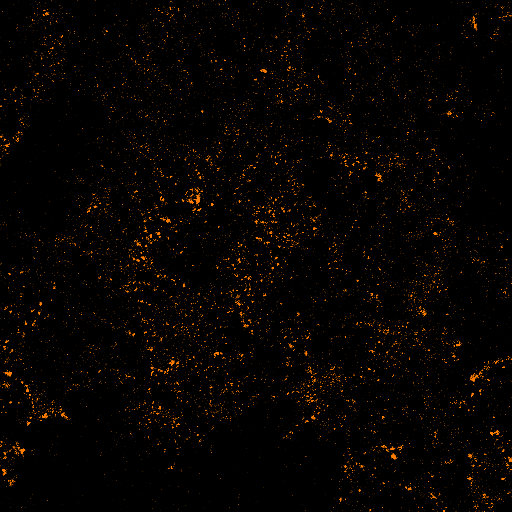

Supplement: Supplementary file 8 — Figure EV1 Source Data [file 44319_2026_829_MOESM8_ESM.zip › Figure EV1/EV 1/EV 1C/EV1C/DiI-HDL/N/DiI.tif]

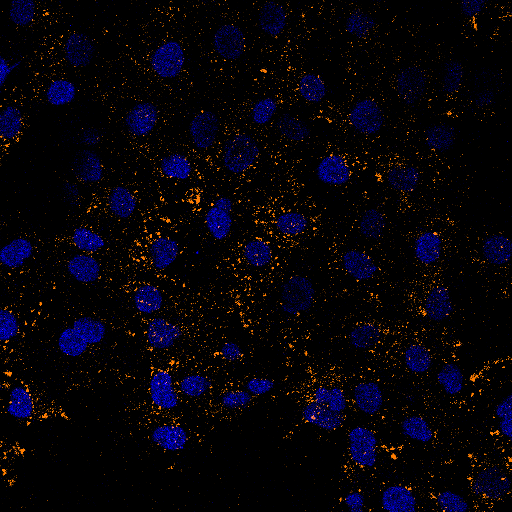

Supplement: Supplementary file 8 — Figure EV1 Source Data [file 44319_2026_829_MOESM8_ESM.zip › Figure EV1/EV 1/EV 1C/EV1C/DiI-HDL/N/MERGE.tif]

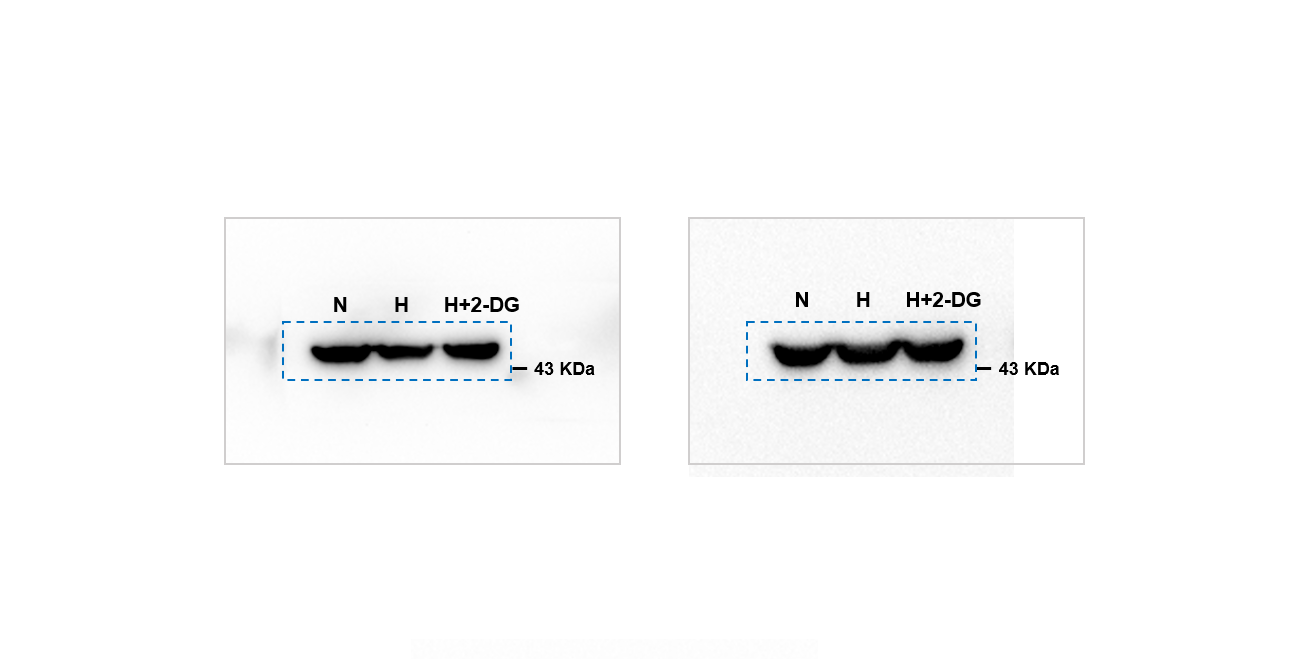

Supplement: Supplementary file 8 — Figure EV1 Source Data [file 44319_2026_829_MOESM8_ESM.zip › Figure EV1/EV 1/EV 1D/western blot/ACTIN.png]

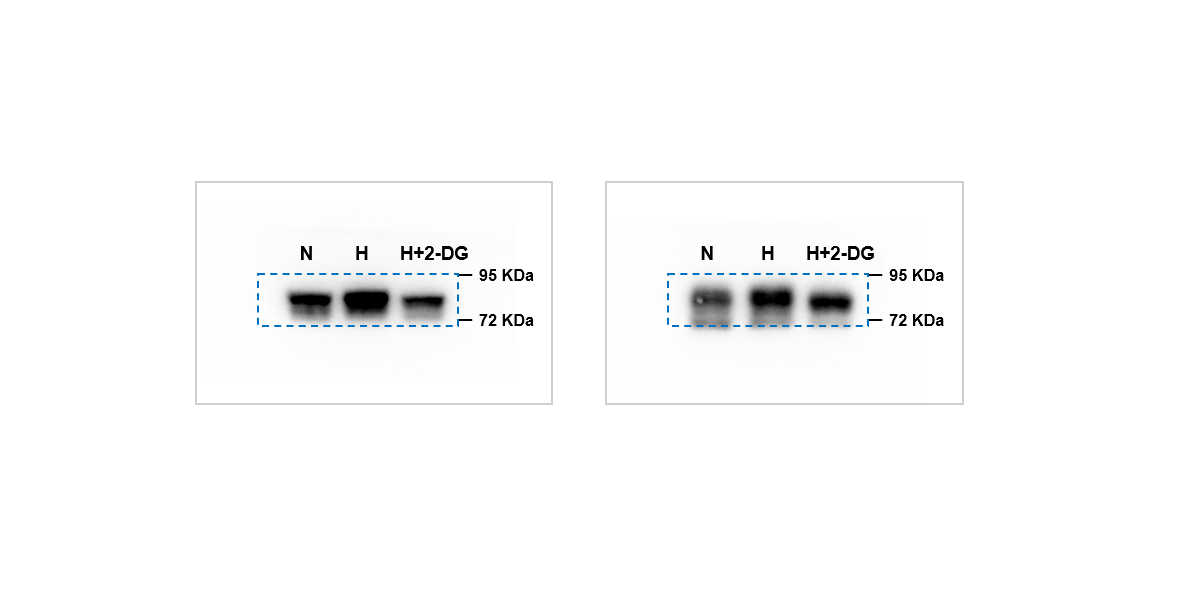

Supplement: Supplementary file 8 — Figure EV1 Source Data [file 44319_2026_829_MOESM8_ESM.zip › Figure EV1/EV 1/EV 1D/western blot/SCARB1.png]

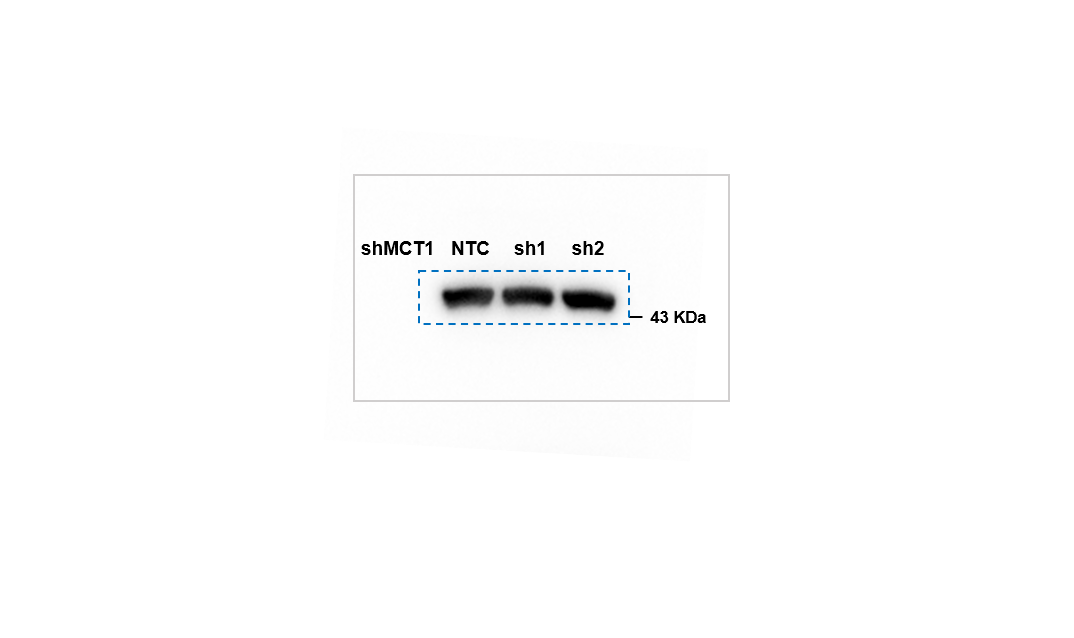

Supplement: Supplementary file 8 — Figure EV1 Source Data [file 44319_2026_829_MOESM8_ESM.zip › Figure EV1/EV 1/EV 1E/ACTIN.png]

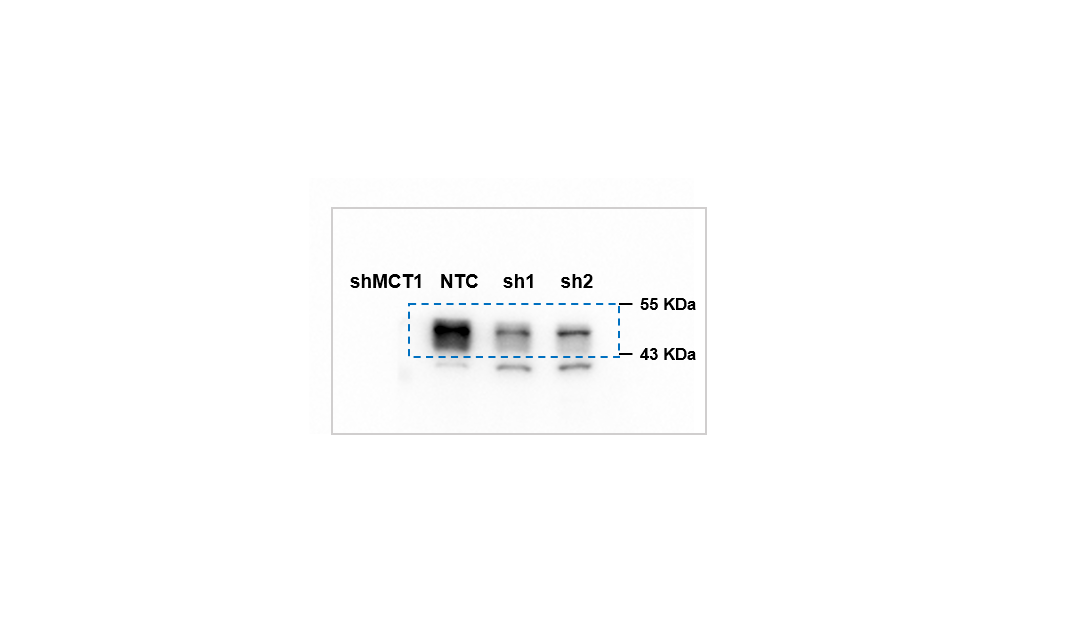

Supplement: Supplementary file 8 — Figure EV1 Source Data [file 44319_2026_829_MOESM8_ESM.zip › Figure EV1/EV 1/EV 1E/MCT1.png]

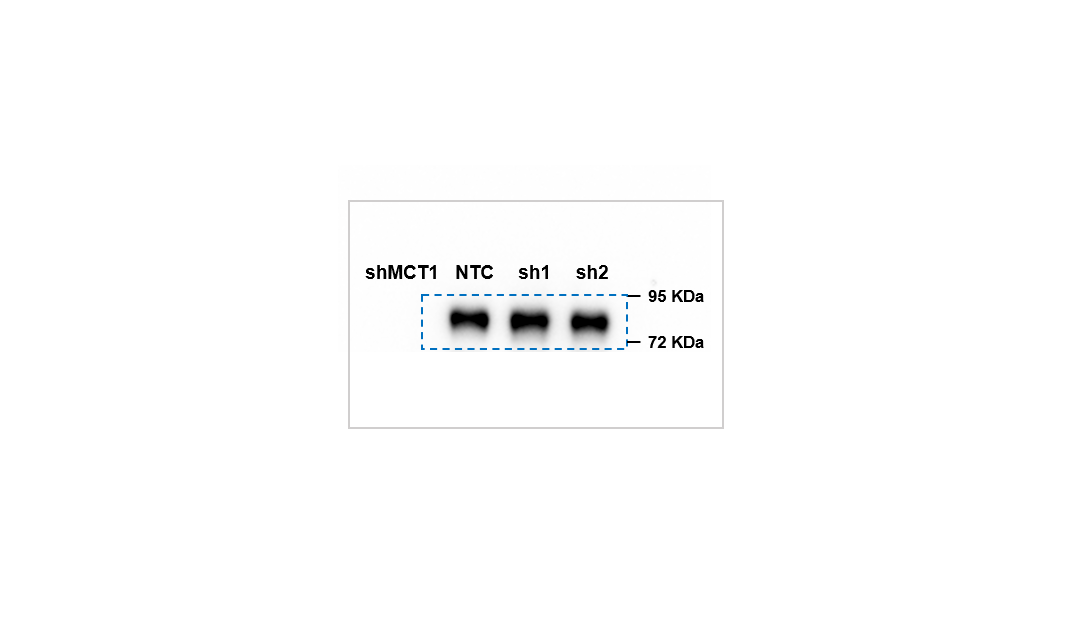

Supplement: Supplementary file 8 — Figure EV1 Source Data [file 44319_2026_829_MOESM8_ESM.zip › Figure EV1/EV 1/EV 1E/SCARB1.png]

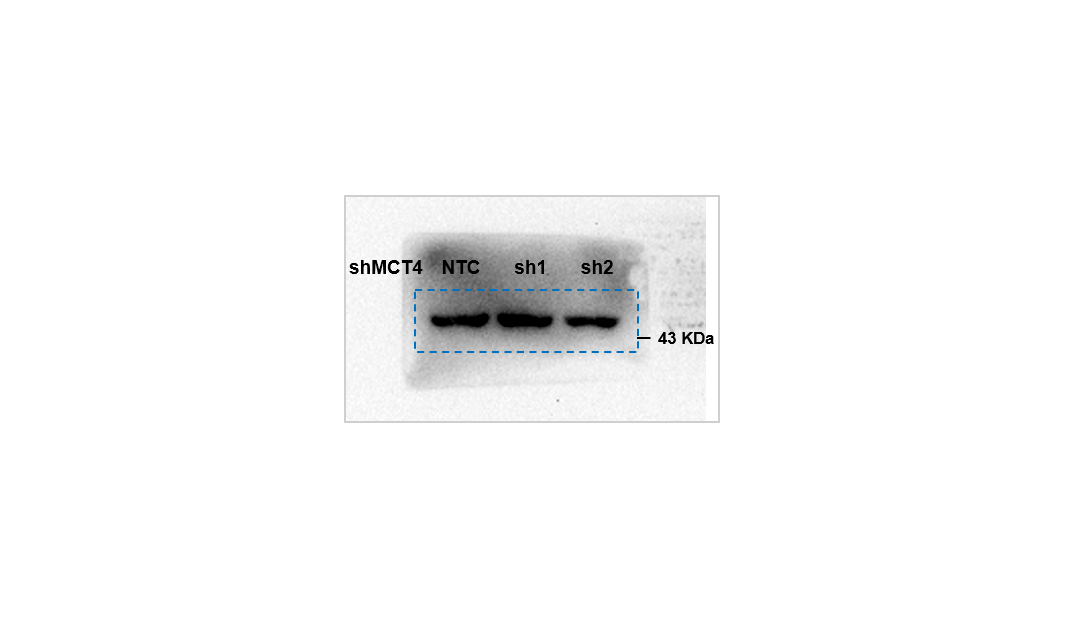

Supplement: Supplementary file 8 — Figure EV1 Source Data [file 44319_2026_829_MOESM8_ESM.zip › Figure EV1/EV 1/EV 1F/ACTIN.png]

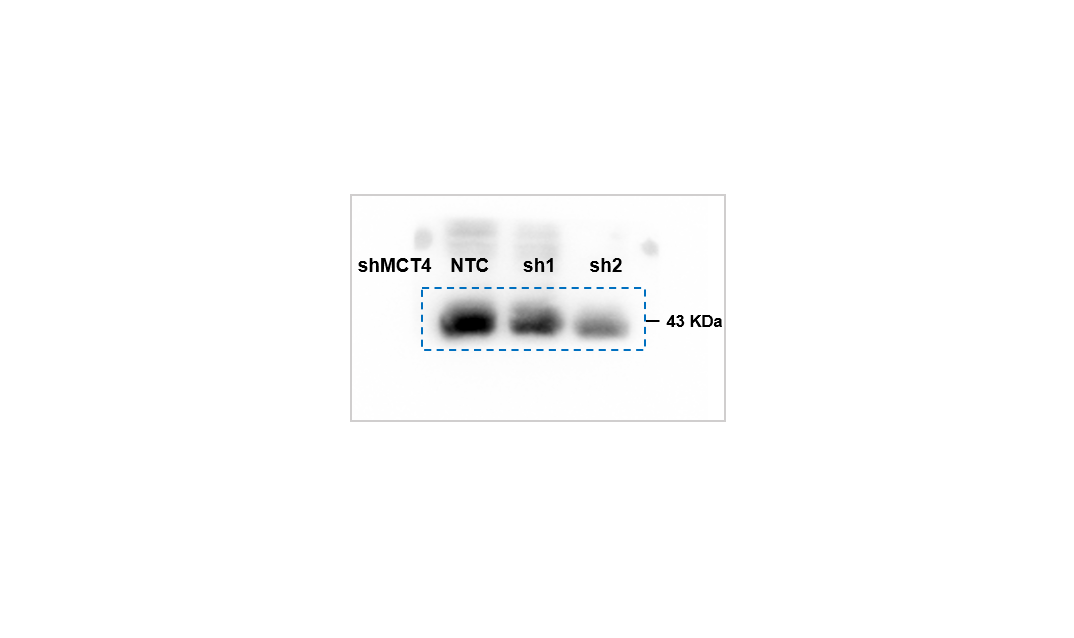

Supplement: Supplementary file 8 — Figure EV1 Source Data [file 44319_2026_829_MOESM8_ESM.zip › Figure EV1/EV 1/EV 1F/MCT4.png]

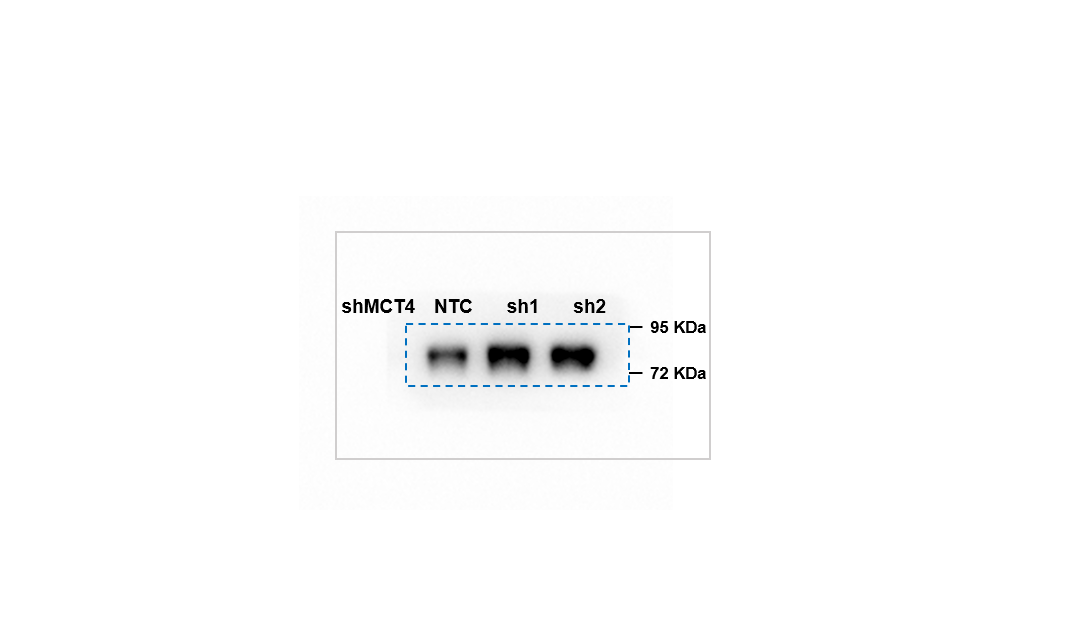

Supplement: Supplementary file 8 — Figure EV1 Source Data [file 44319_2026_829_MOESM8_ESM.zip › Figure EV1/EV 1/EV 1F/SCARB1.png]

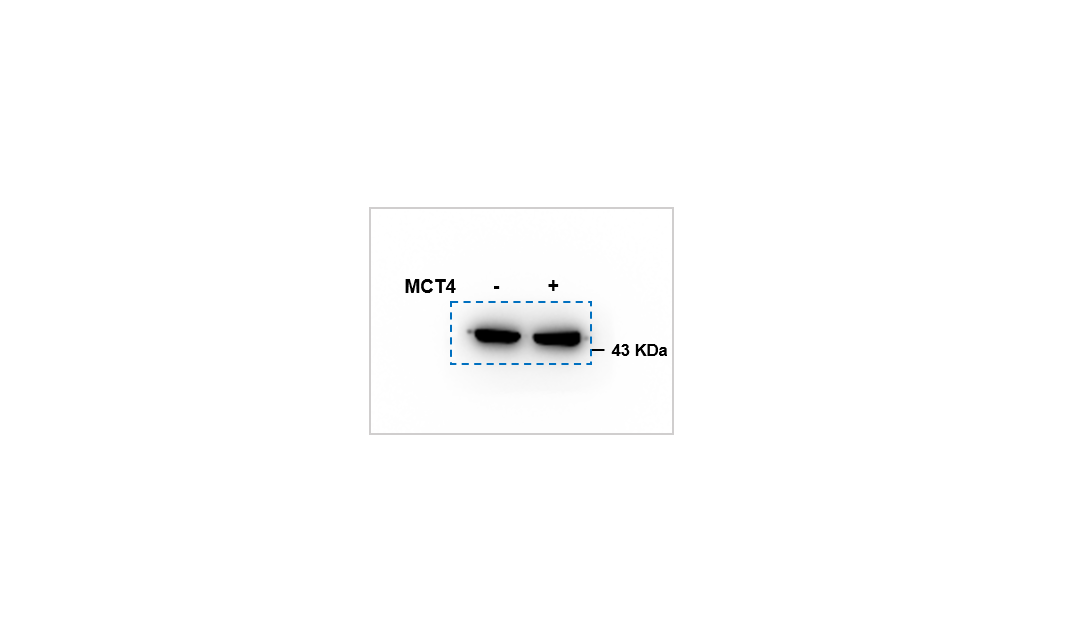

Supplement: Supplementary file 8 — Figure EV1 Source Data [file 44319_2026_829_MOESM8_ESM.zip › Figure EV1/EV 1/EV 1G/ACTIN.png]

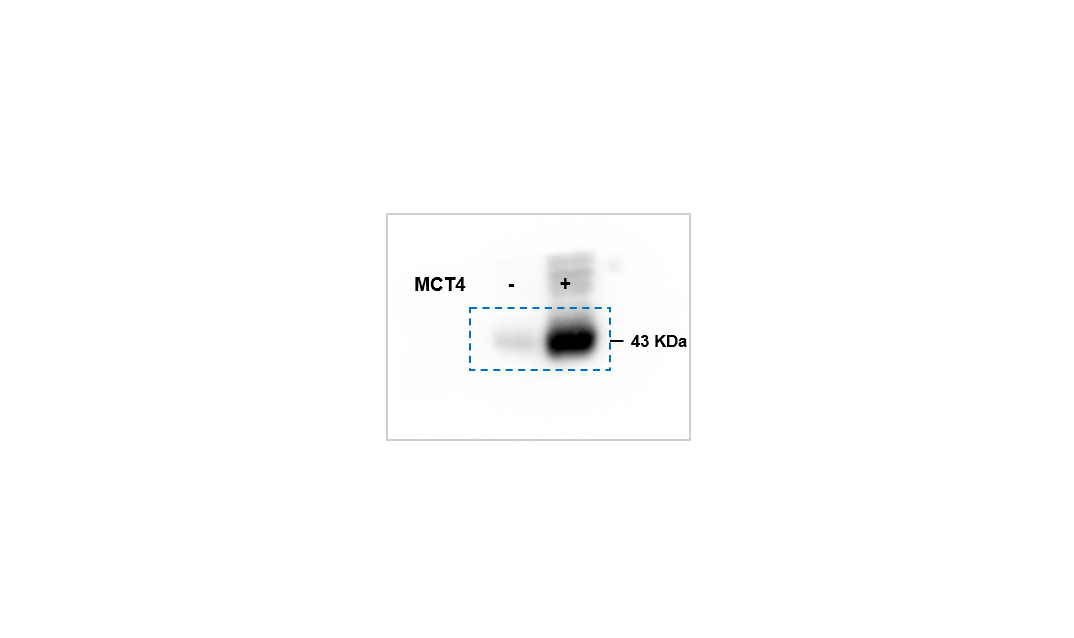

Supplement: Supplementary file 8 — Figure EV1 Source Data [file 44319_2026_829_MOESM8_ESM.zip › Figure EV1/EV 1/EV 1G/MCT4.png]

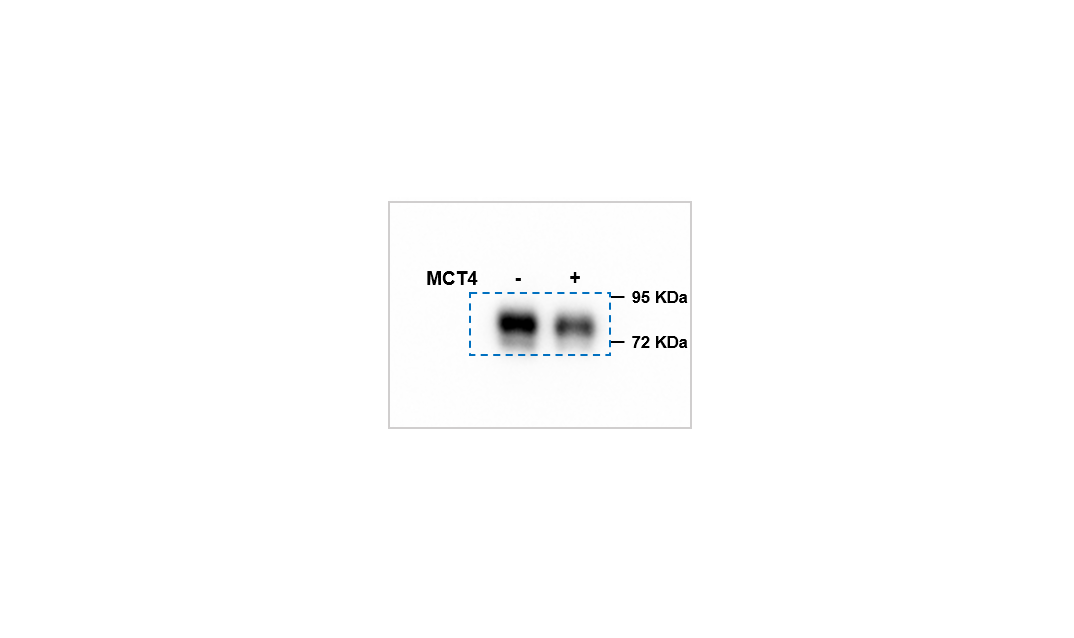

Supplement: Supplementary file 8 — Figure EV1 Source Data [file 44319_2026_829_MOESM8_ESM.zip › Figure EV1/EV 1/EV 1G/SCARB1.png]

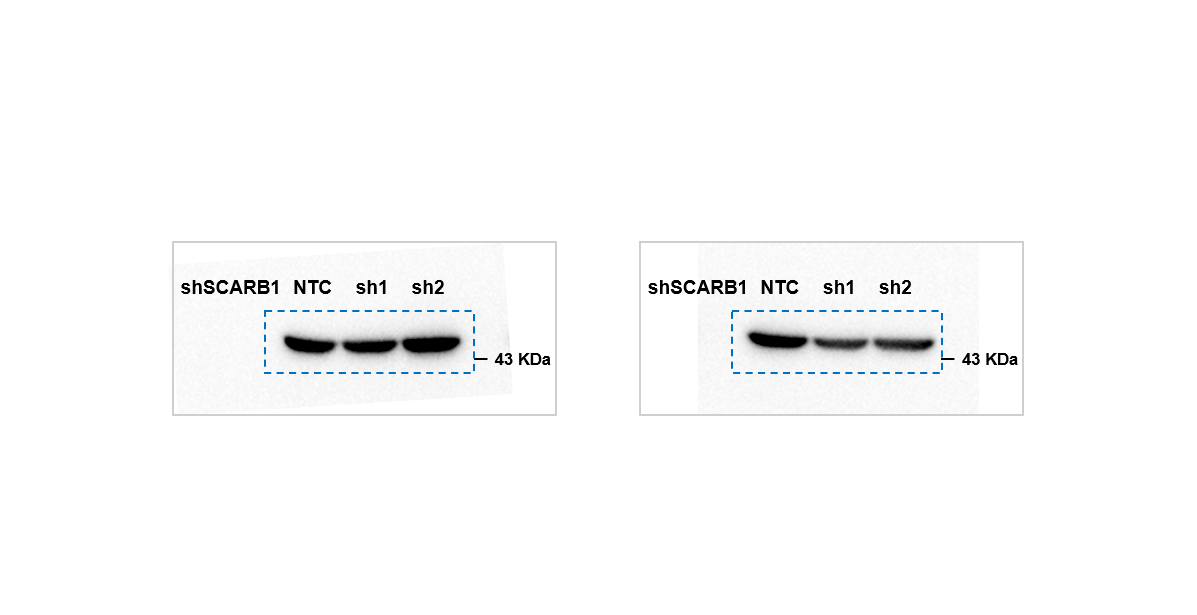

Supplement: Supplementary file 8 — Figure EV1 Source Data [file 44319_2026_829_MOESM8_ESM.zip › Figure EV1/EV 1/EV 1H/KD/ACTIN.png]

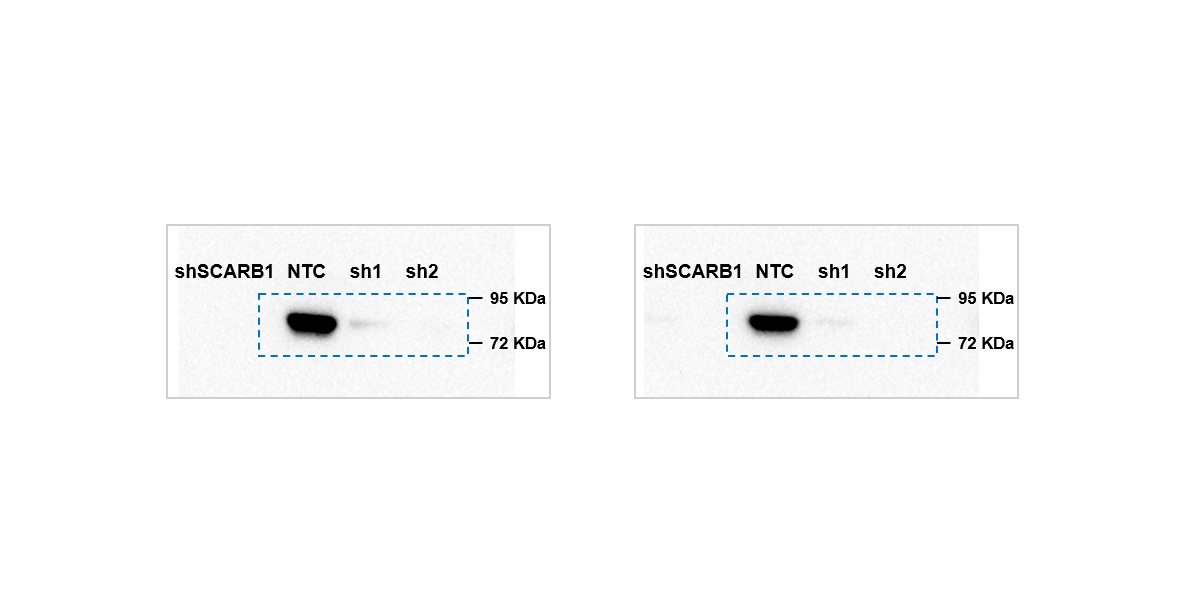

Supplement: Supplementary file 8 — Figure EV1 Source Data [file 44319_2026_829_MOESM8_ESM.zip › Figure EV1/EV 1/EV 1H/KD/SCARB1.png]

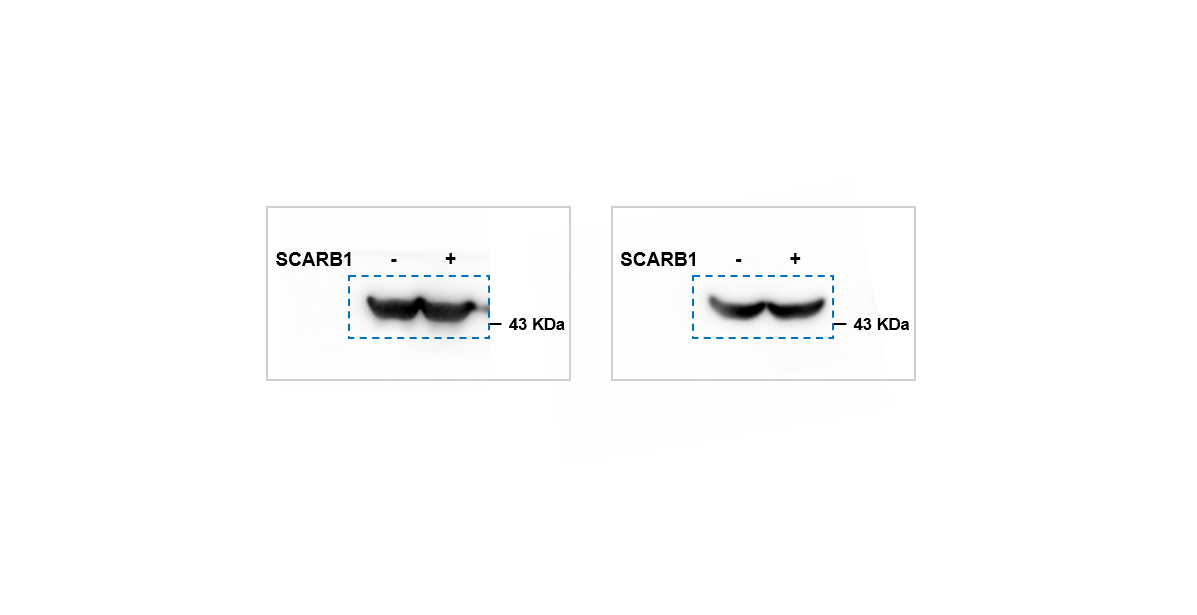

Supplement: Supplementary file 8 — Figure EV1 Source Data [file 44319_2026_829_MOESM8_ESM.zip › Figure EV1/EV 1/EV 1H/OE/ACTIN.png]

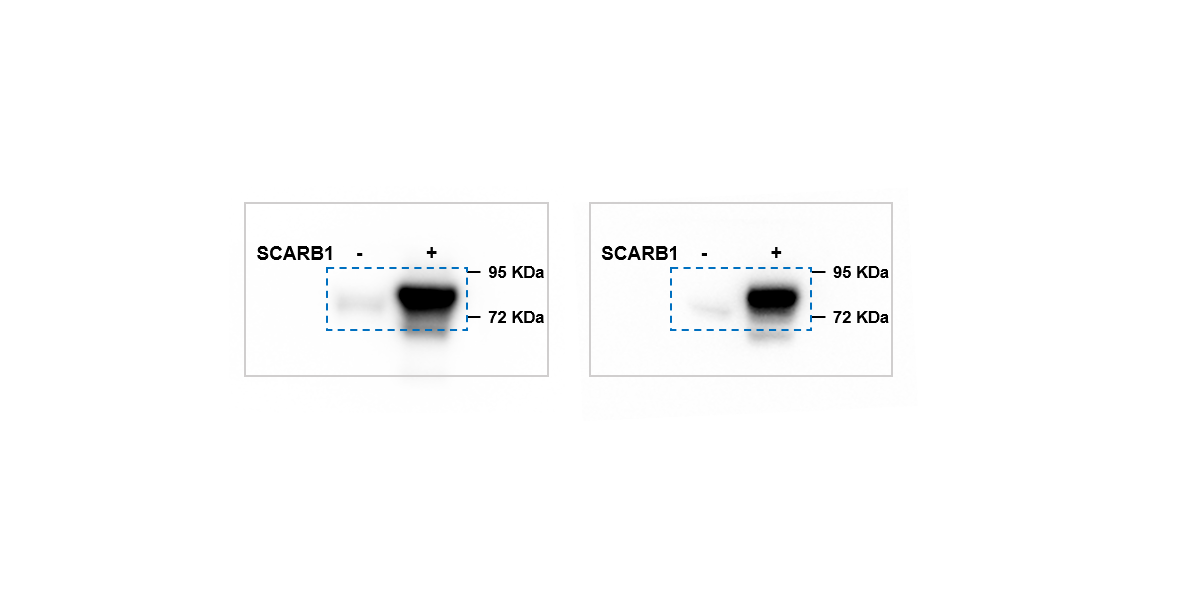

Supplement: Supplementary file 8 — Figure EV1 Source Data [file 44319_2026_829_MOESM8_ESM.zip › Figure EV1/EV 1/EV 1H/OE/SCARB1.png]

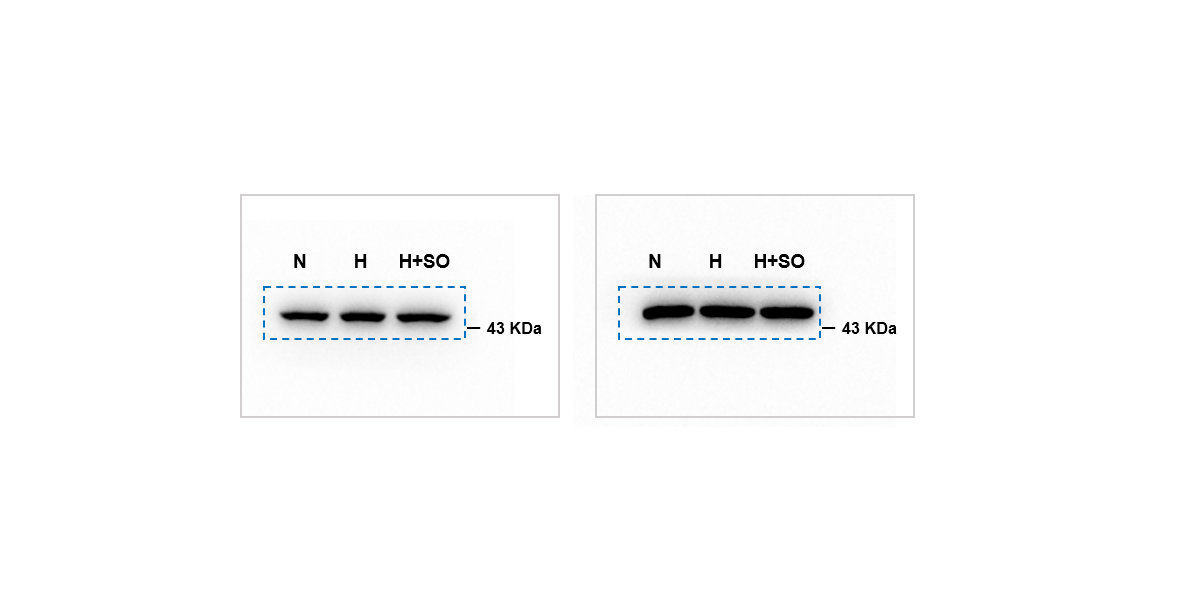

Supplement: Supplementary file 9 — Figure EV2 Source Data [file 44319_2026_829_MOESM9_ESM.zip › Figure EV2/EV2/A/ACTIN.png]

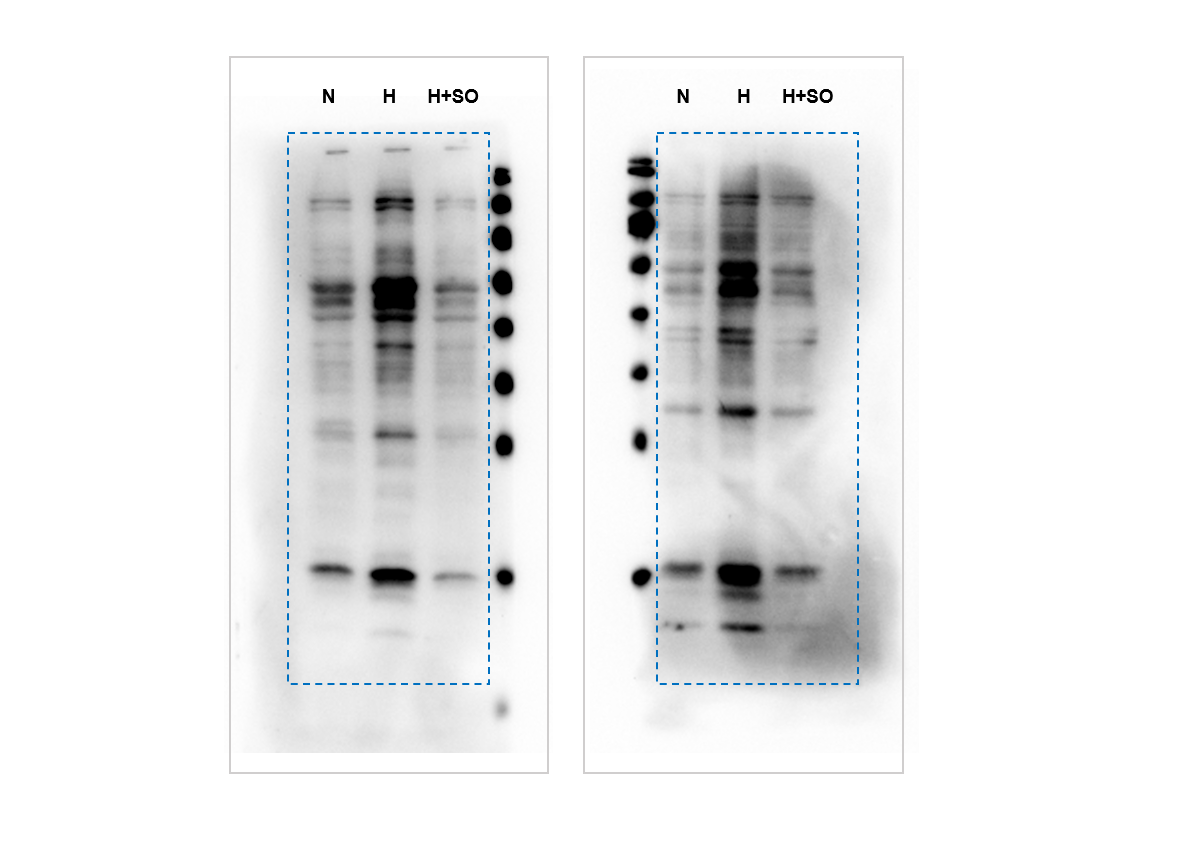

Supplement: Supplementary file 9 — Figure EV2 Source Data [file 44319_2026_829_MOESM9_ESM.zip › Figure EV2/EV2/A/Pan-Kla.png]

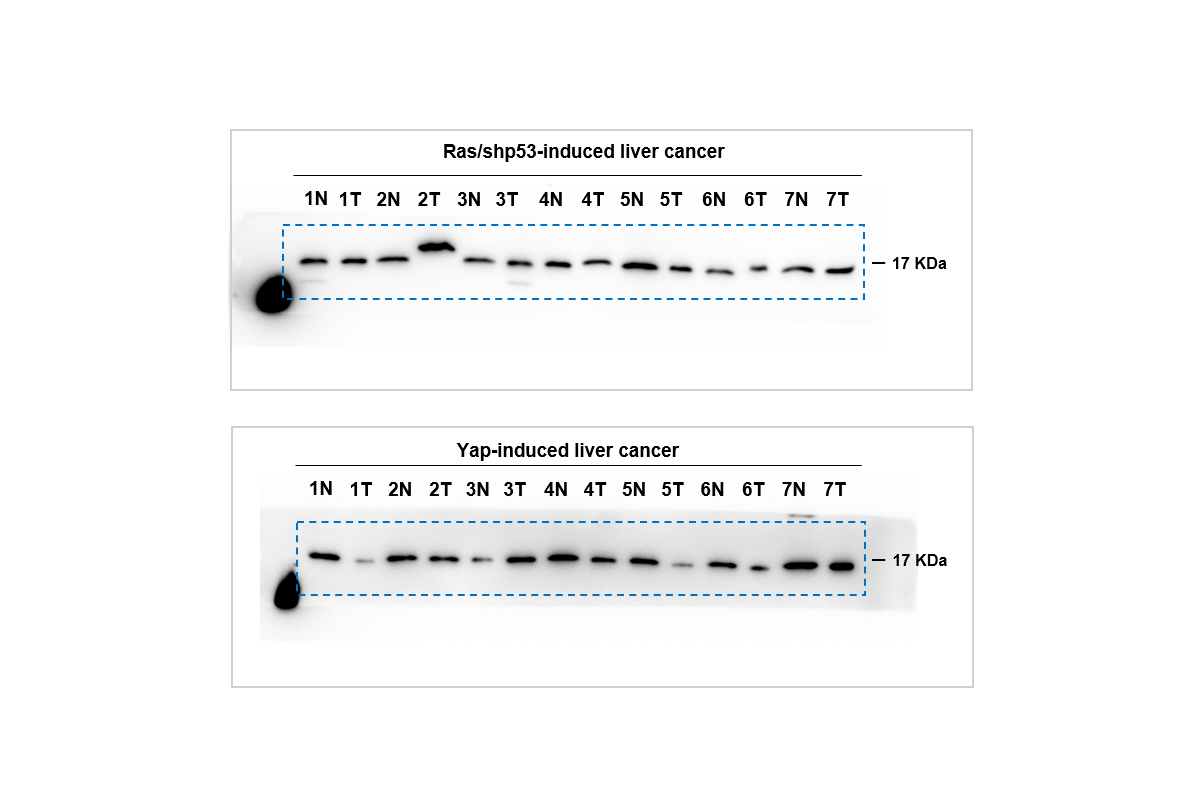

Supplement: Supplementary file 9 — Figure EV2 Source Data [file 44319_2026_829_MOESM9_ESM.zip › Figure EV2/EV2/B/H3.png]

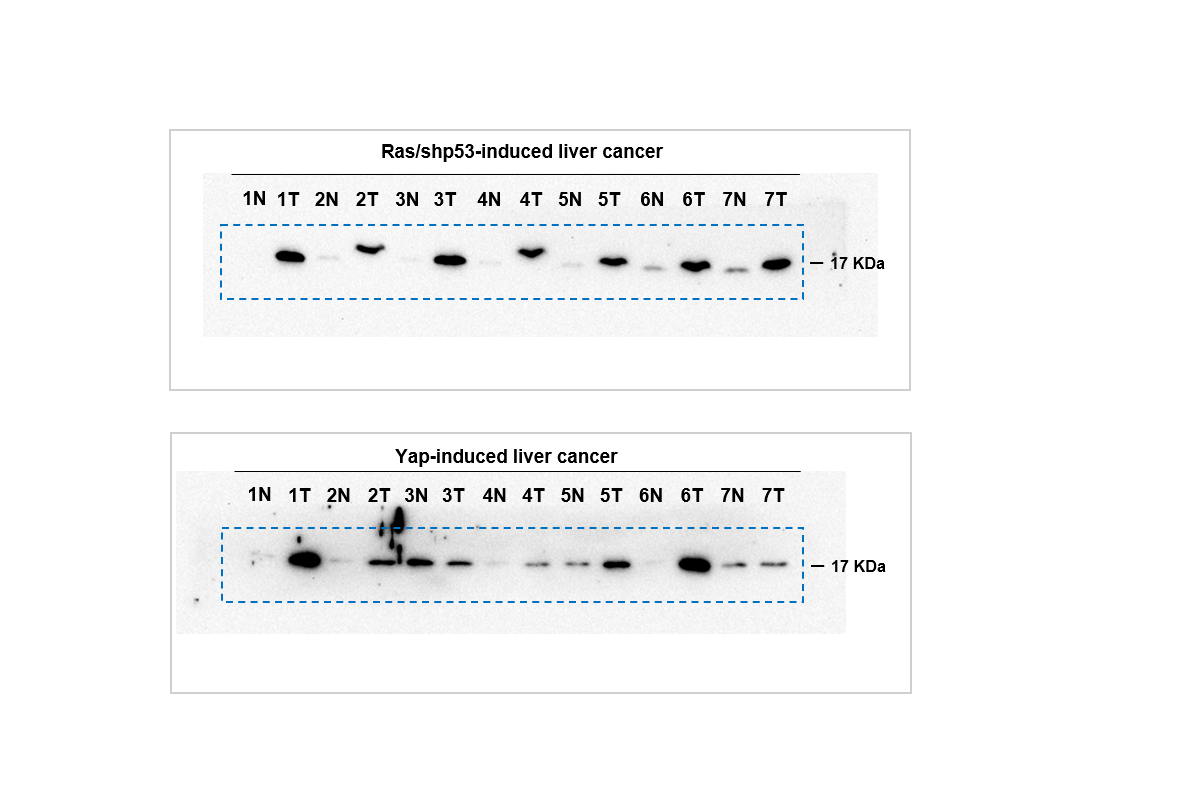

Supplement: Supplementary file 9 — Figure EV2 Source Data [file 44319_2026_829_MOESM9_ESM.zip › Figure EV2/EV2/B/H3K18la.png]

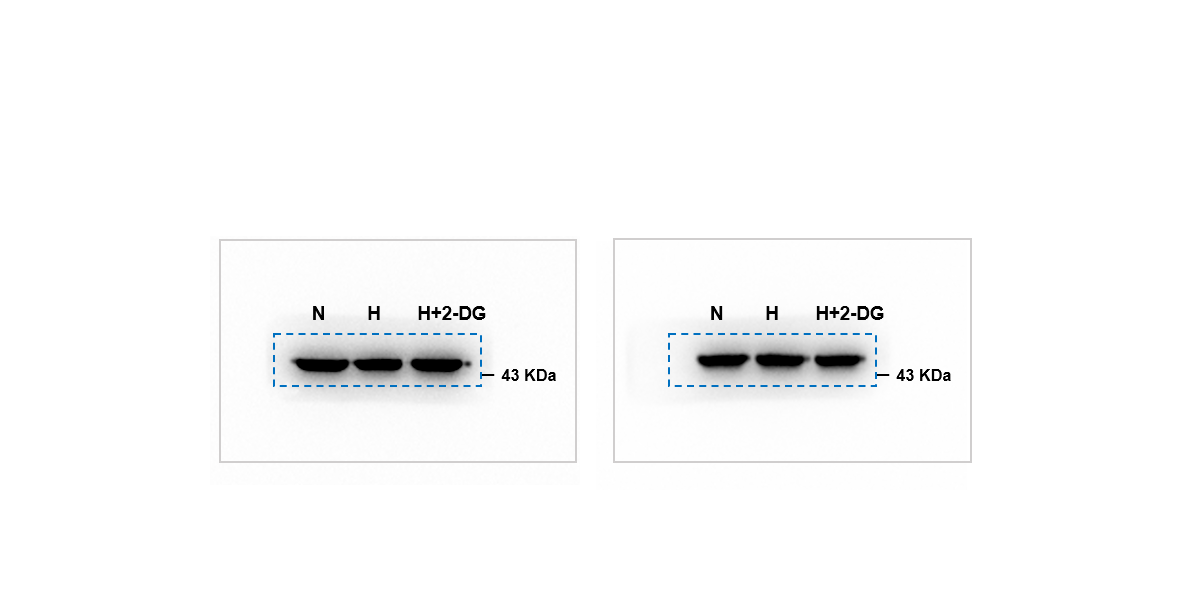

Supplement: Supplementary file 9 — Figure EV2 Source Data [file 44319_2026_829_MOESM9_ESM.zip › Figure EV2/EV2/C/ACTIN.png]

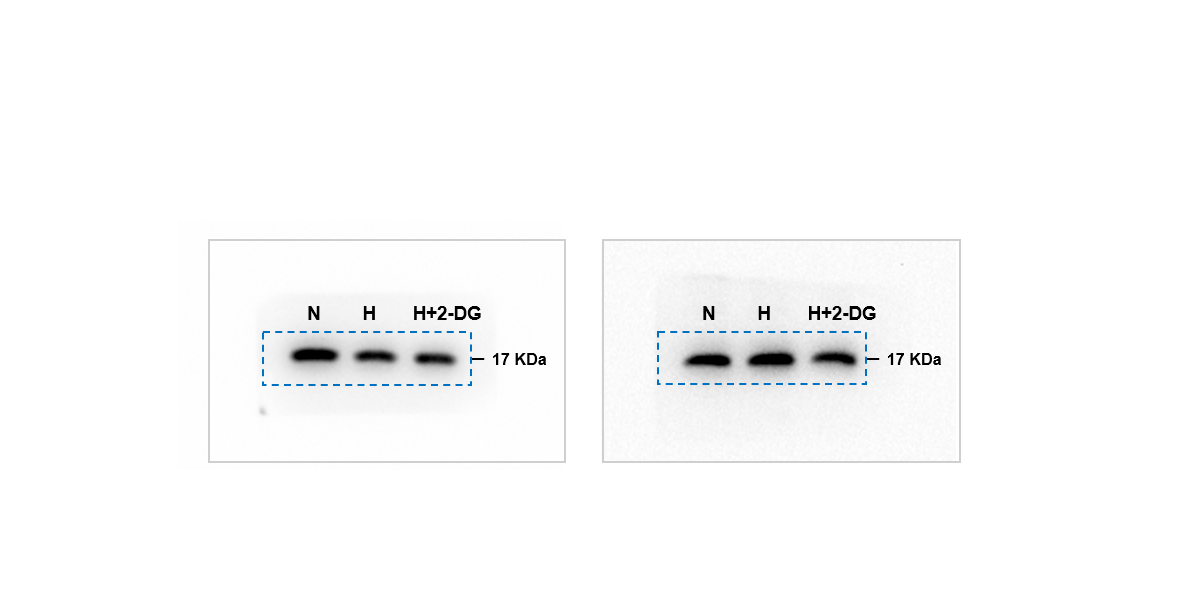

Supplement: Supplementary file 9 — Figure EV2 Source Data [file 44319_2026_829_MOESM9_ESM.zip › Figure EV2/EV2/C/H3.png]

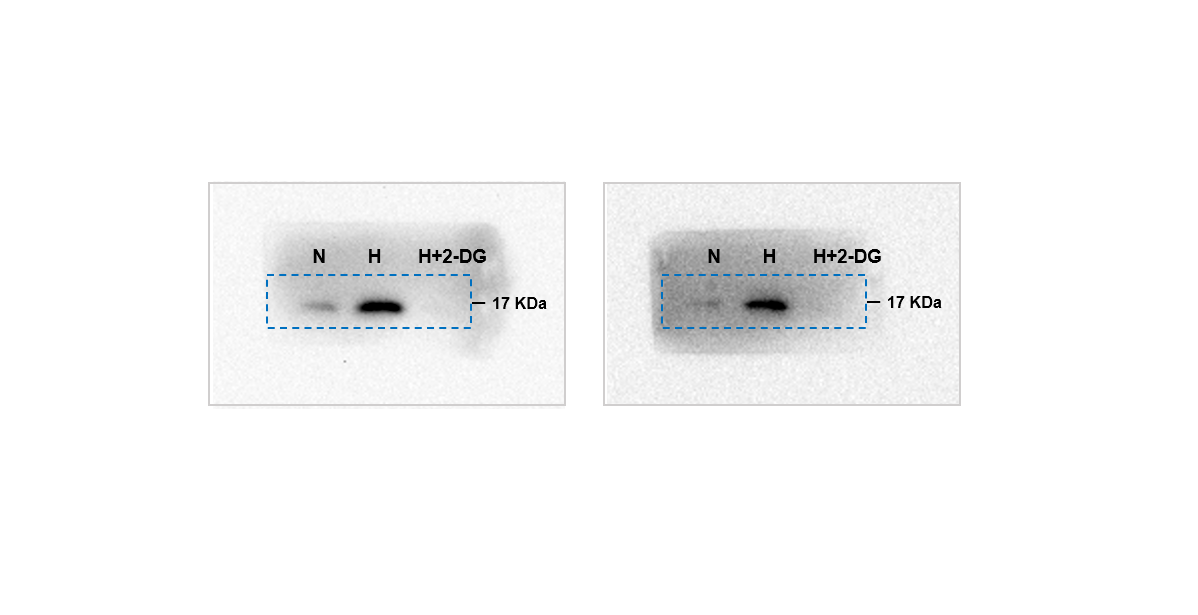

Supplement: Supplementary file 9 — Figure EV2 Source Data [file 44319_2026_829_MOESM9_ESM.zip › Figure EV2/EV2/C/H3K18la.png]

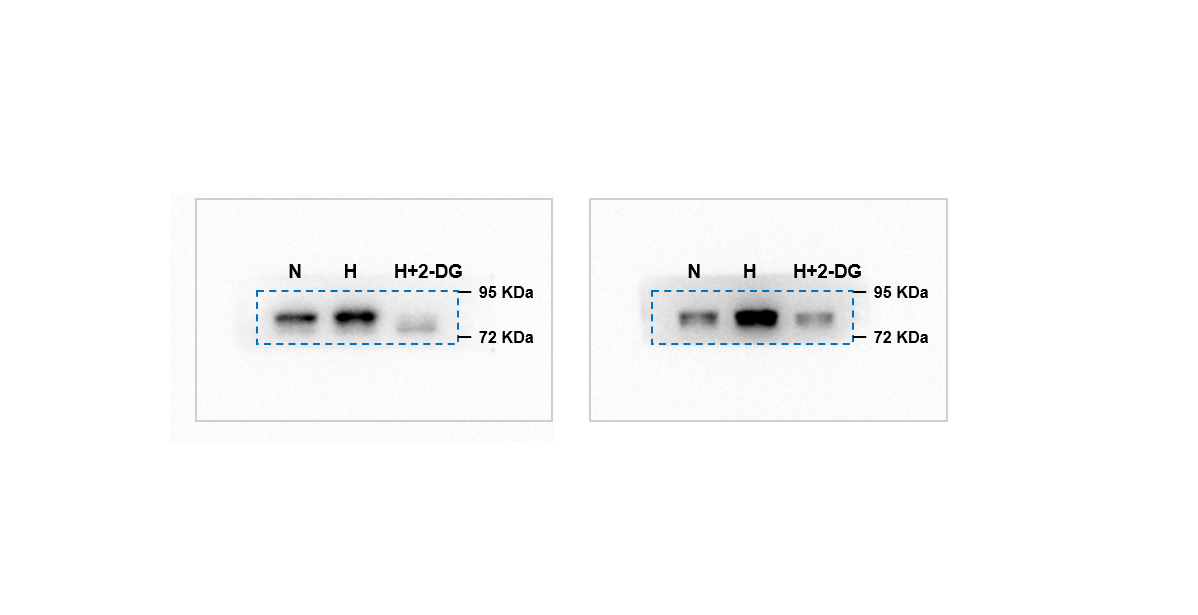

Supplement: Supplementary file 9 — Figure EV2 Source Data [file 44319_2026_829_MOESM9_ESM.zip › Figure EV2/EV2/C/SCARB1.png]

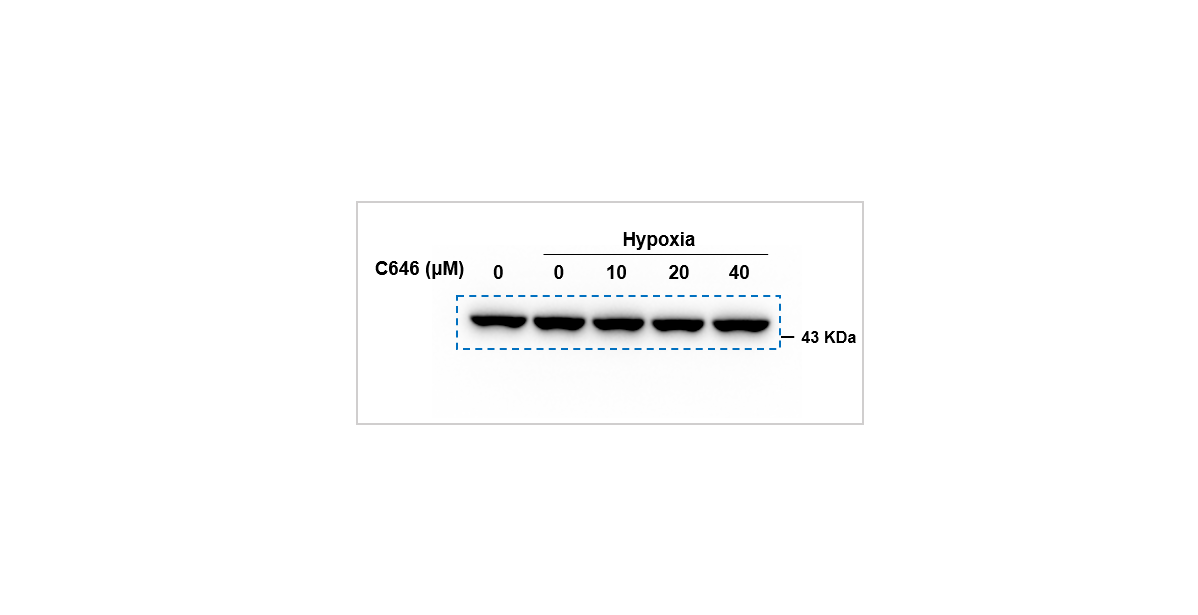

Supplement: Supplementary file 9 — Figure EV2 Source Data [file 44319_2026_829_MOESM9_ESM.zip › Figure EV2/EV2/D/ACTIN.png]

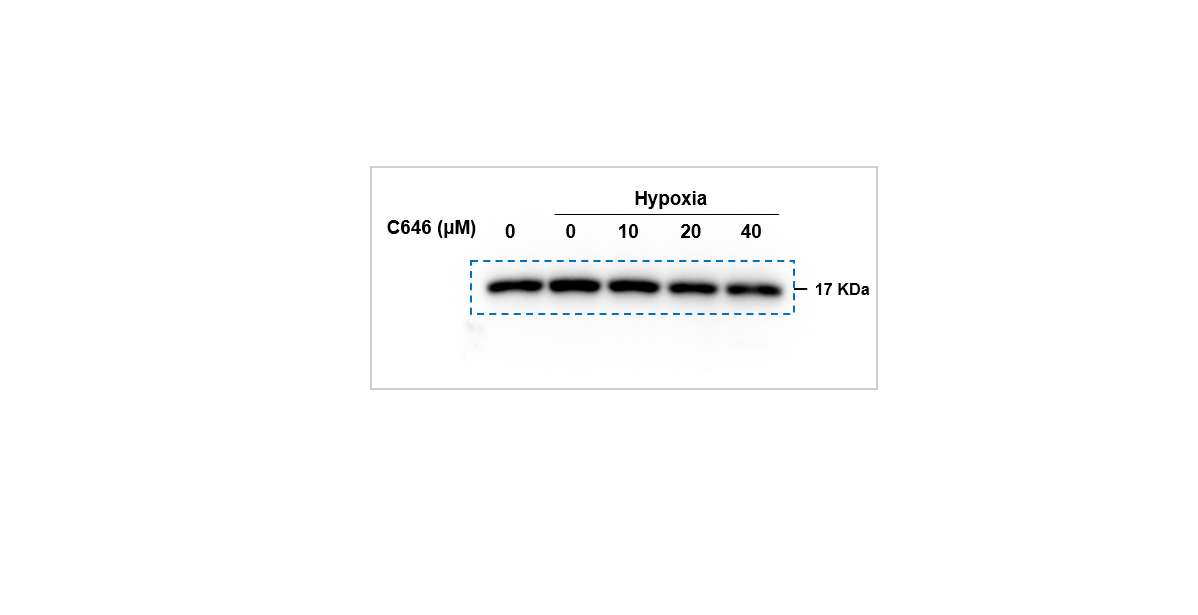

Supplement: Supplementary file 9 — Figure EV2 Source Data [file 44319_2026_829_MOESM9_ESM.zip › Figure EV2/EV2/D/H3.png]

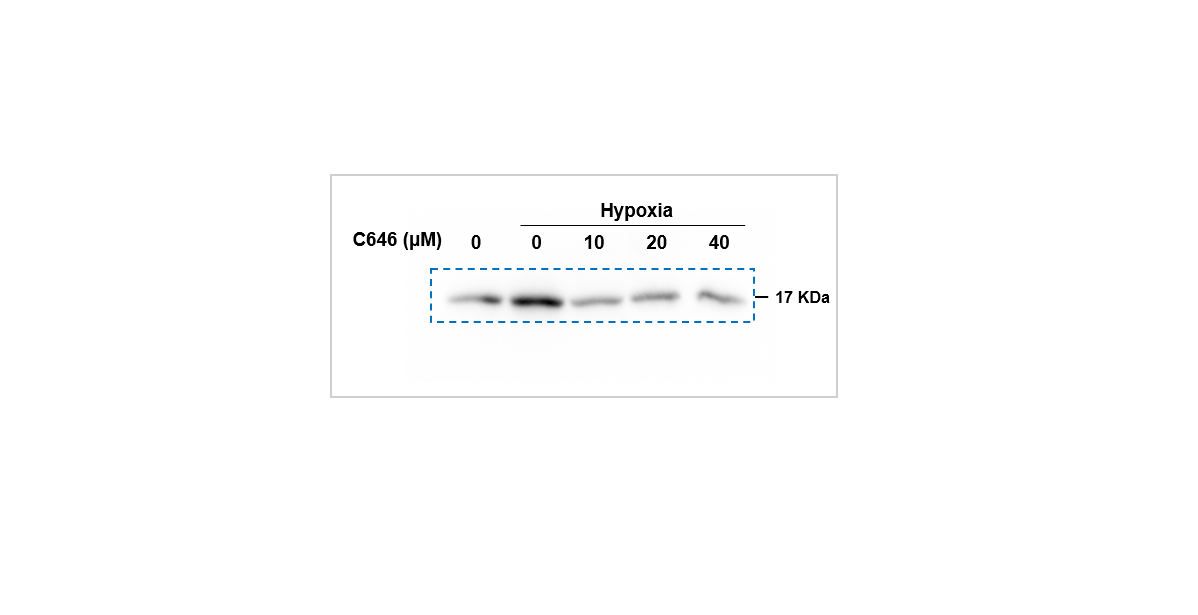

Supplement: Supplementary file 9 — Figure EV2 Source Data [file 44319_2026_829_MOESM9_ESM.zip › Figure EV2/EV2/D/H3K18la.png]

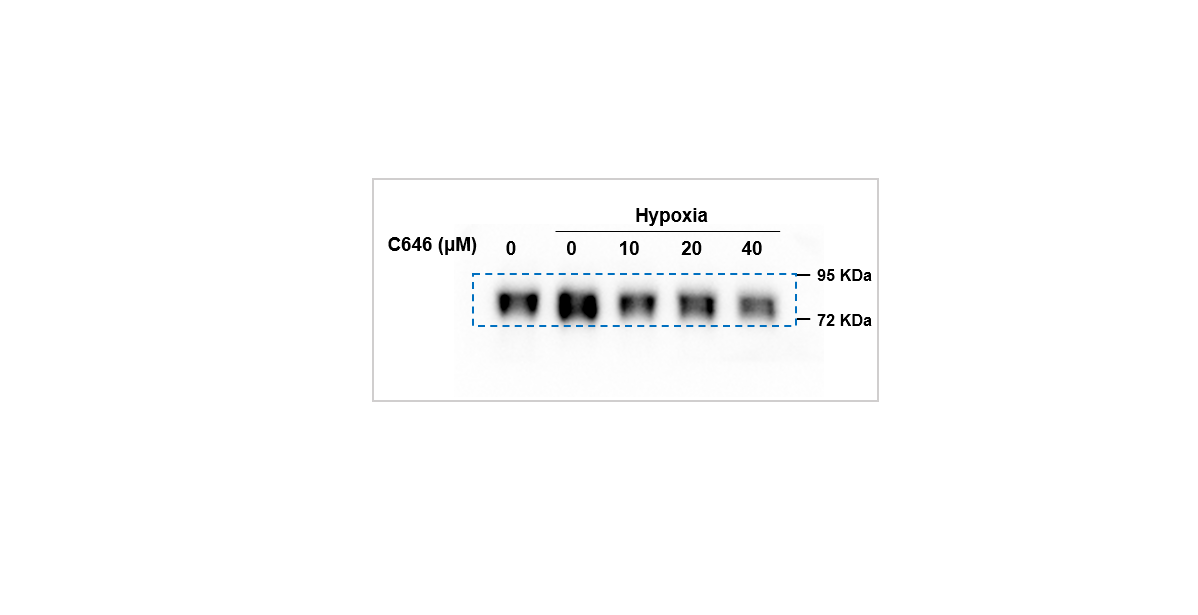

Supplement: Supplementary file 9 — Figure EV2 Source Data [file 44319_2026_829_MOESM9_ESM.zip › Figure EV2/EV2/D/SCARB1.png]

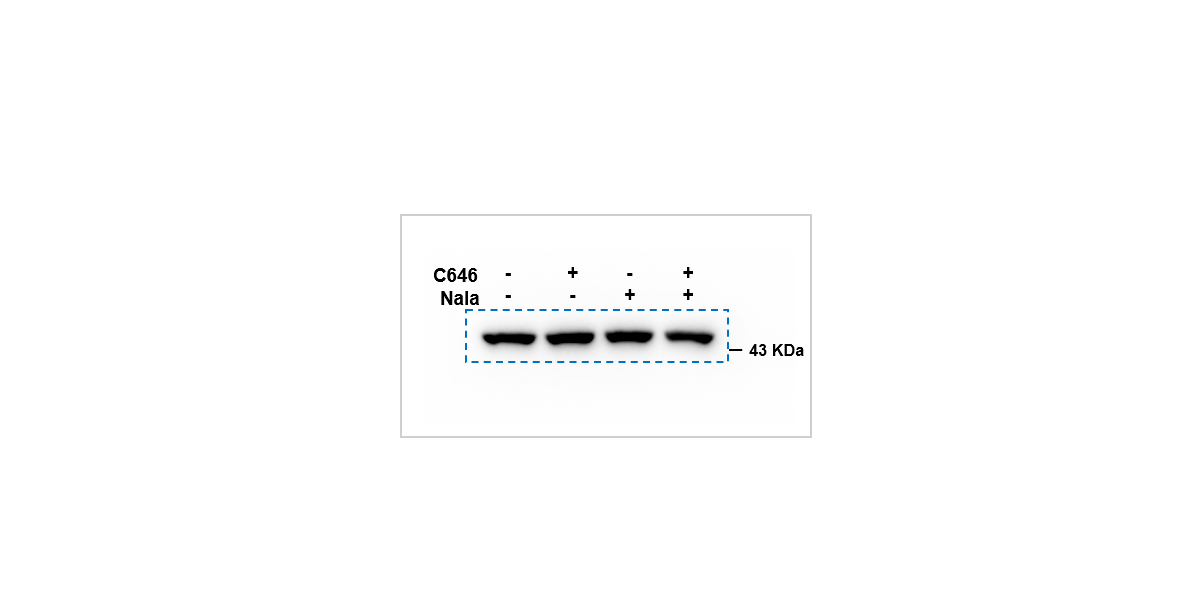

Supplement: Supplementary file 9 — Figure EV2 Source Data [file 44319_2026_829_MOESM9_ESM.zip › Figure EV2/EV2/E/ACTIN.png]

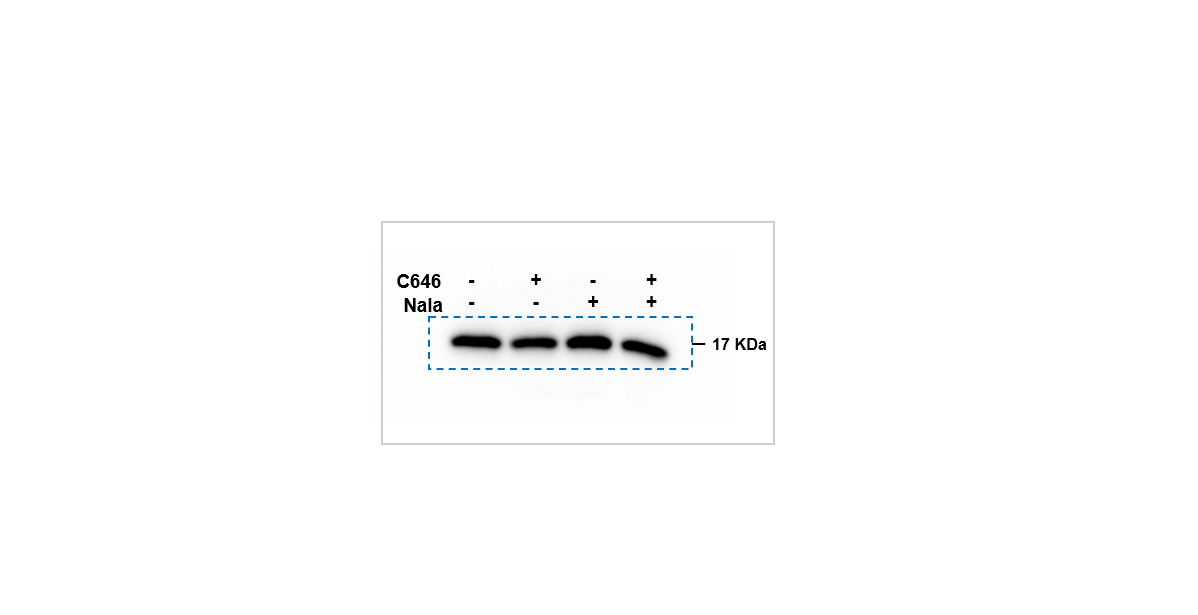

Supplement: Supplementary file 9 — Figure EV2 Source Data [file 44319_2026_829_MOESM9_ESM.zip › Figure EV2/EV2/E/H3.png]

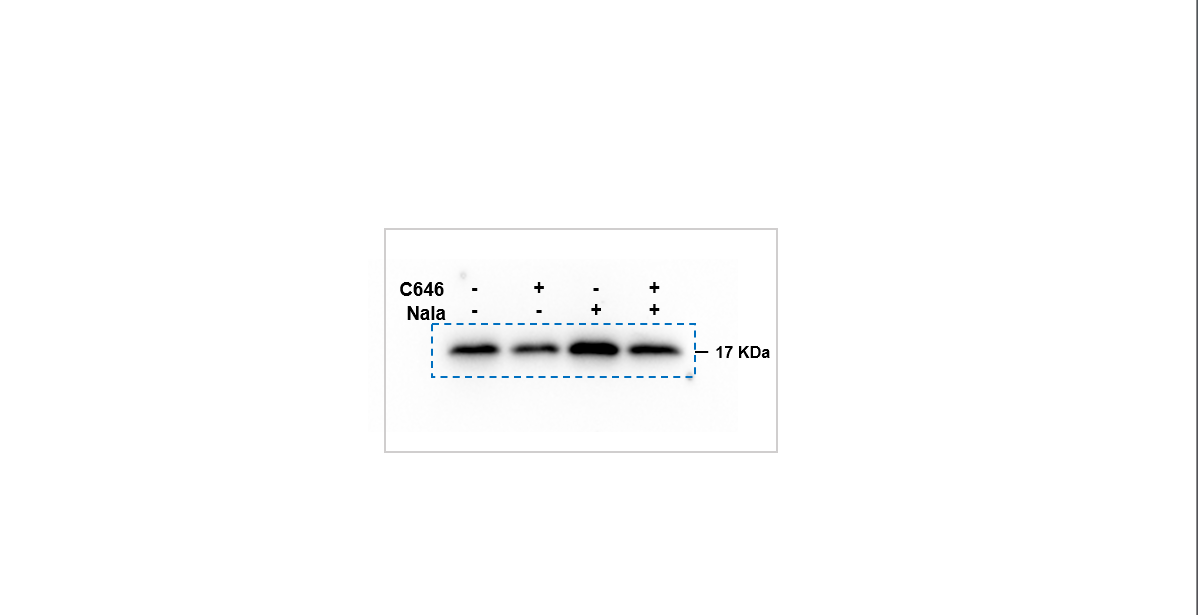

Supplement: Supplementary file 9 — Figure EV2 Source Data [file 44319_2026_829_MOESM9_ESM.zip › Figure EV2/EV2/E/H3K18la.png]

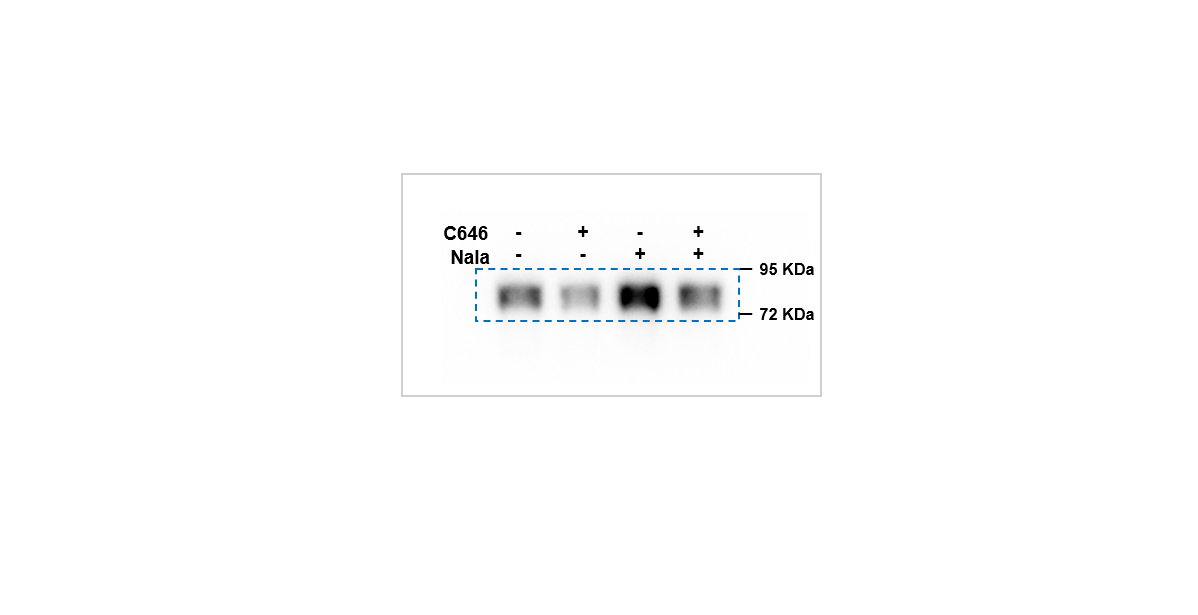

Supplement: Supplementary file 9 — Figure EV2 Source Data [file 44319_2026_829_MOESM9_ESM.zip › Figure EV2/EV2/E/SCARB1.png]

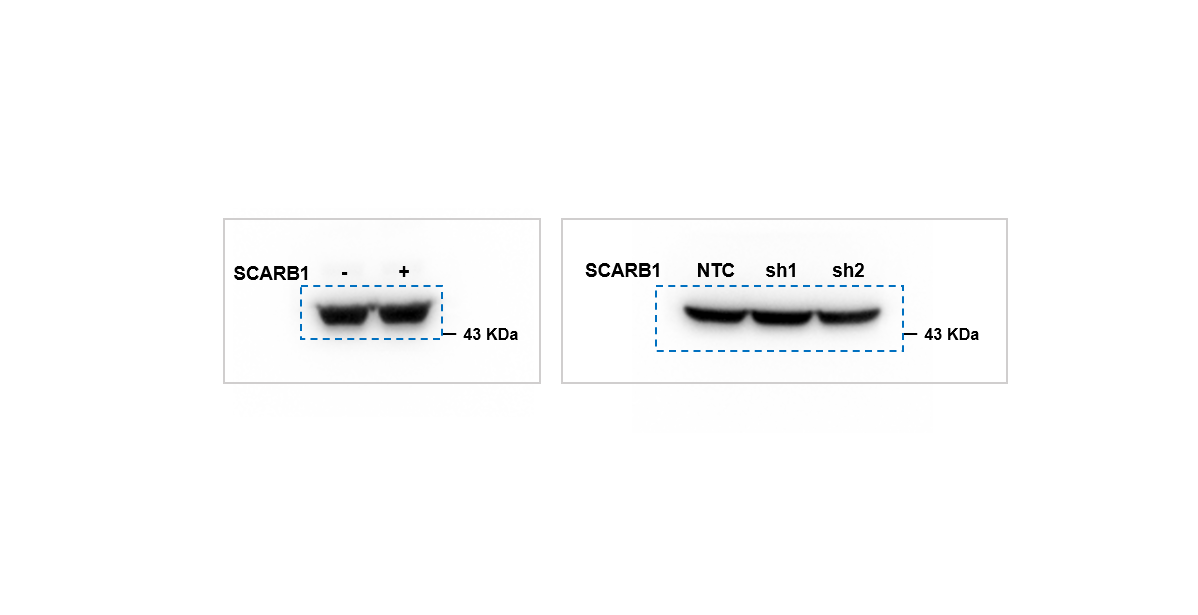

Supplement: Supplementary file 10 — Figure EV3 Source Data [file 44319_2026_829_MOESM10_ESM.zip › Figure EV3/EV3/C/ACTIN.png]

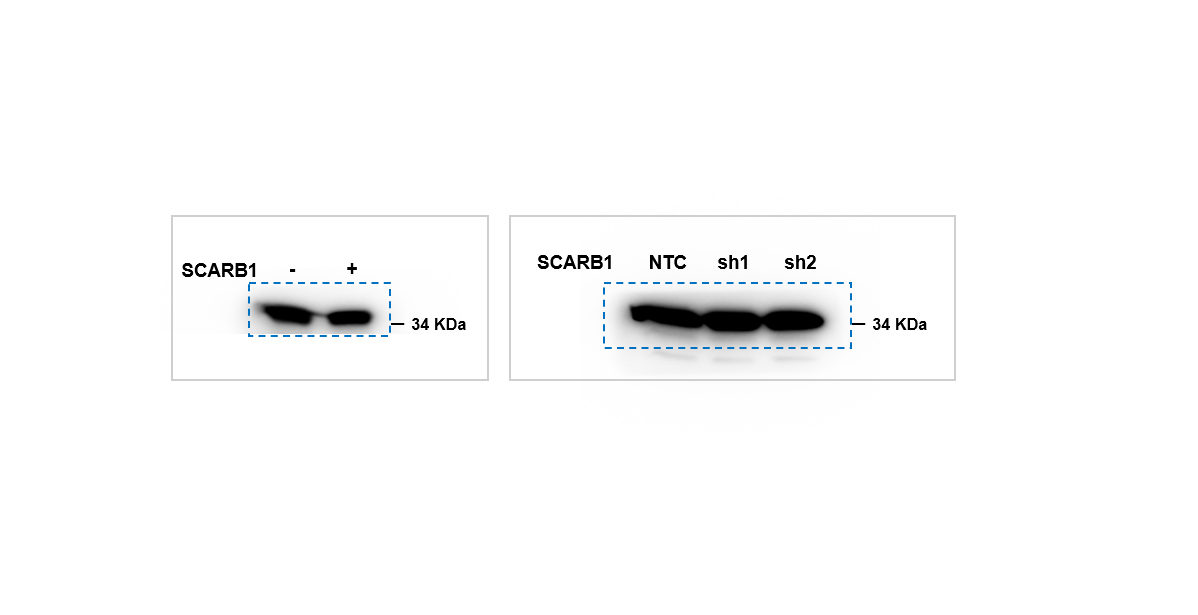

Supplement: Supplementary file 10 — Figure EV3 Source Data [file 44319_2026_829_MOESM10_ESM.zip › Figure EV3/EV3/C/Caspase 3.png]

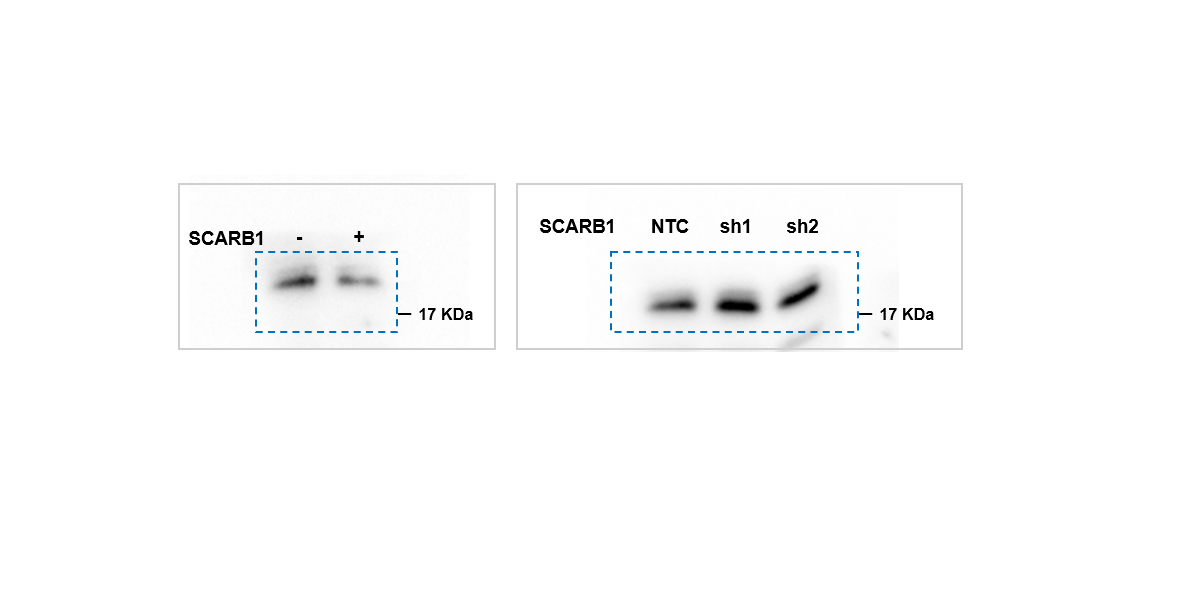

Supplement: Supplementary file 10 — Figure EV3 Source Data [file 44319_2026_829_MOESM10_ESM.zip › Figure EV3/EV3/C/Cleaved Caspase 3.png]

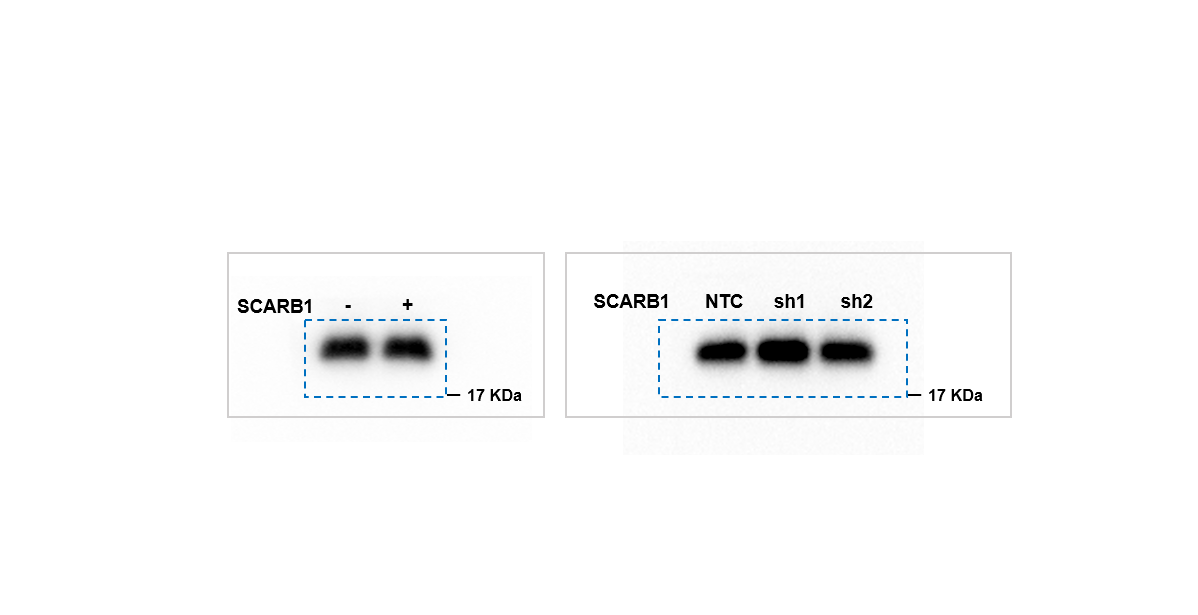

Supplement: Supplementary file 10 — Figure EV3 Source Data [file 44319_2026_829_MOESM10_ESM.zip › Figure EV3/EV3/C/GPX4.png]

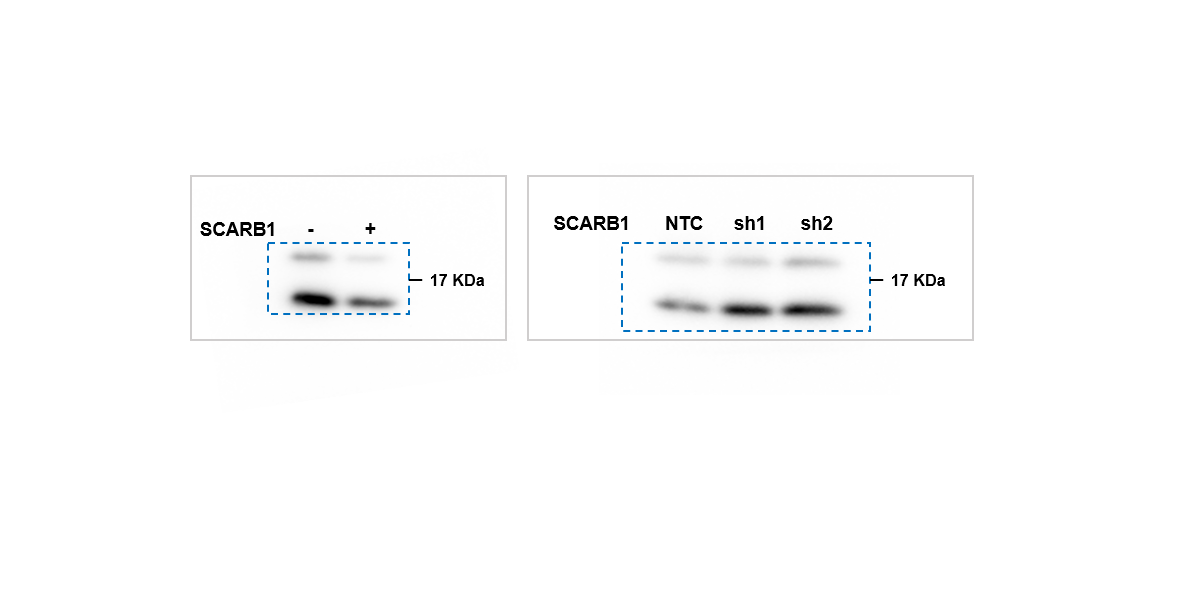

Supplement: Supplementary file 10 — Figure EV3 Source Data [file 44319_2026_829_MOESM10_ESM.zip › Figure EV3/EV3/C/LC3.png]

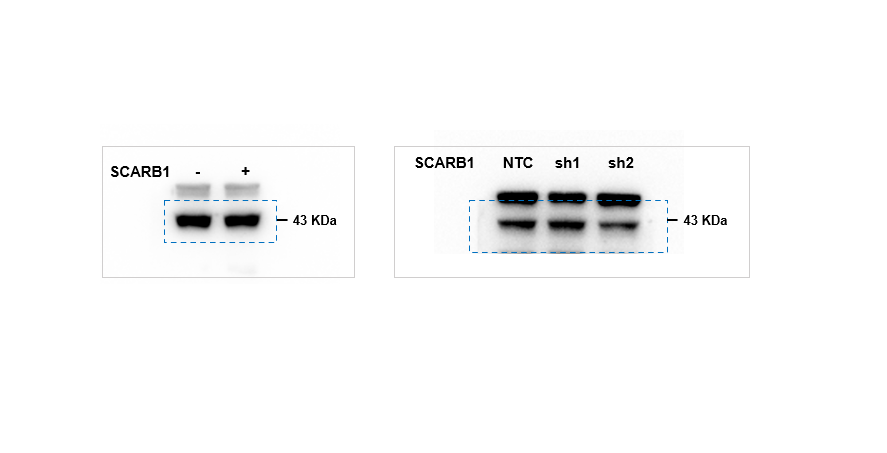

Supplement: Supplementary file 10 — Figure EV3 Source Data [file 44319_2026_829_MOESM10_ESM.zip › Figure EV3/EV3/C/MLKL.png]

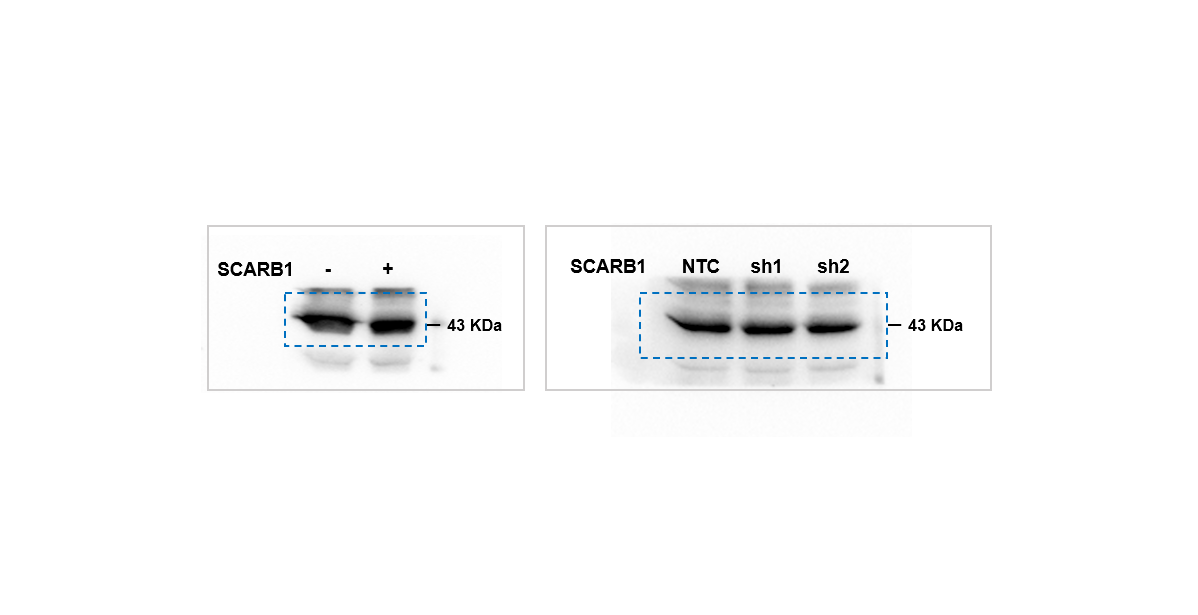

Supplement: Supplementary file 10 — Figure EV3 Source Data [file 44319_2026_829_MOESM10_ESM.zip › Figure EV3/EV3/C/p-MLKL.png]

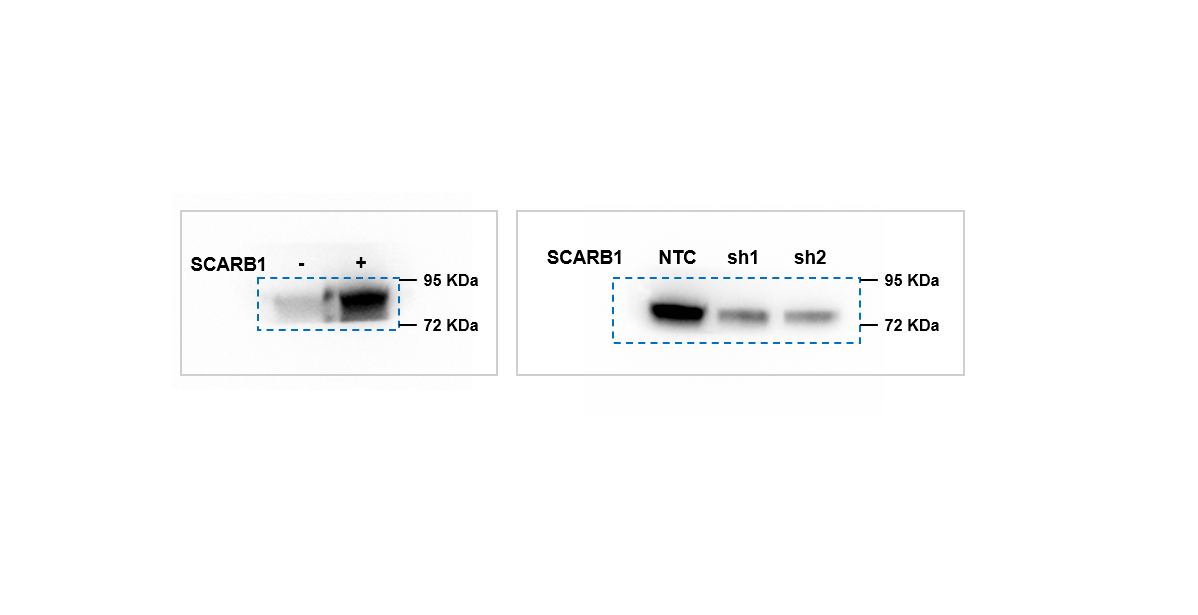

Supplement: Supplementary file 10 — Figure EV3 Source Data [file 44319_2026_829_MOESM10_ESM.zip › Figure EV3/EV3/C/SCARB1.png]

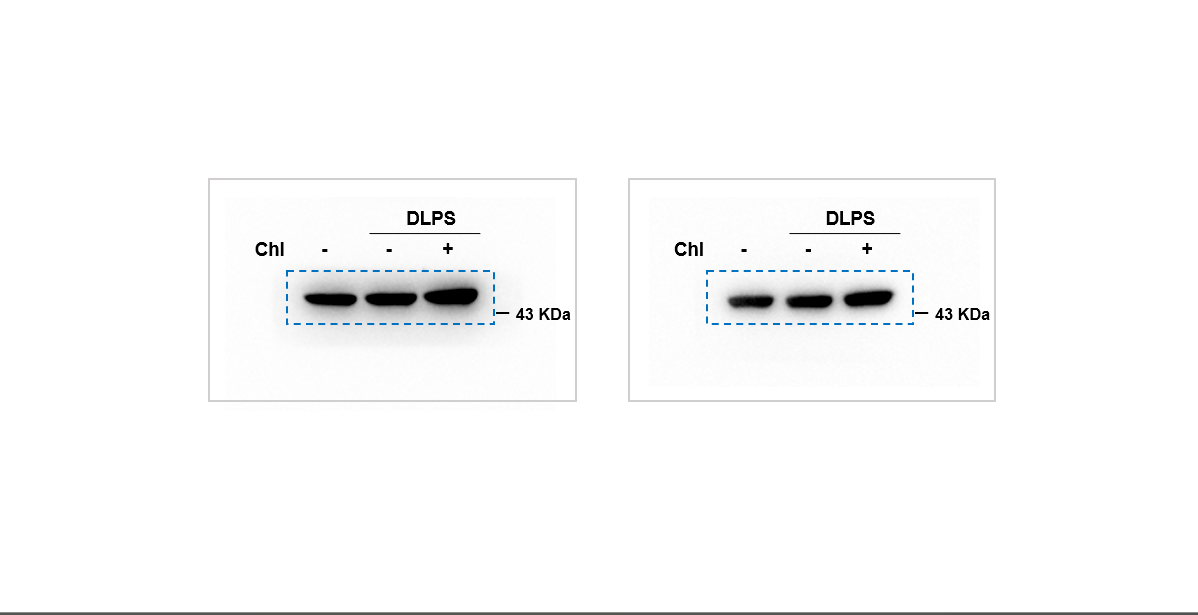

Supplement: Supplementary file 11 — Figure EV4 Source Data [file 44319_2026_829_MOESM11_ESM.zip › Figure EV4/EV4/A/ACTIN.png]

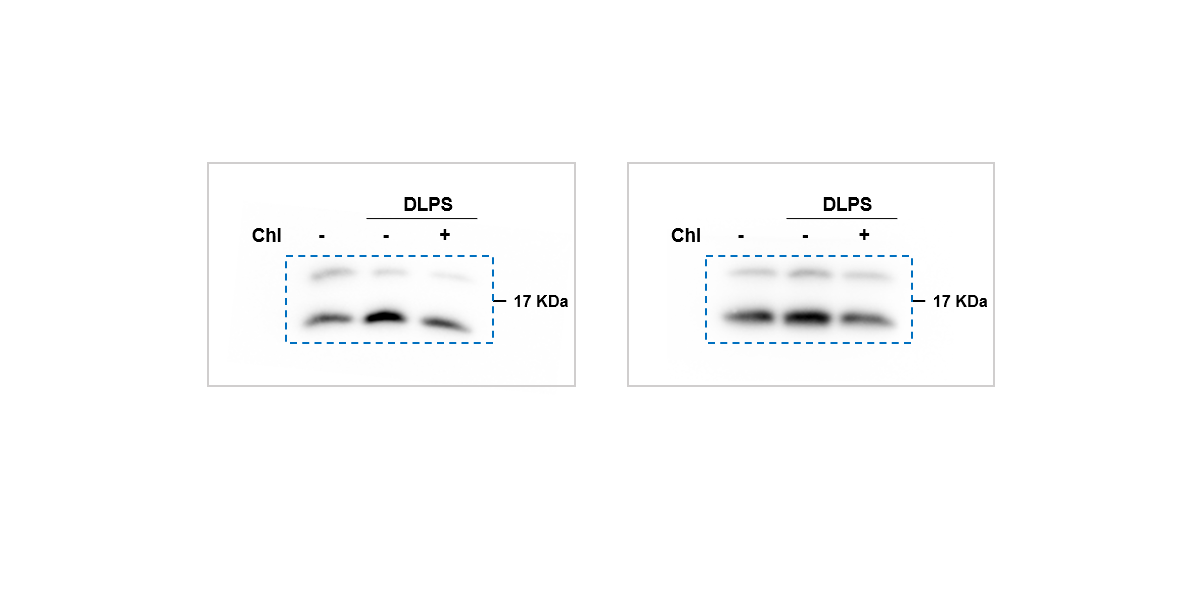

Supplement: Supplementary file 11 — Figure EV4 Source Data [file 44319_2026_829_MOESM11_ESM.zip › Figure EV4/EV4/A/LC3.png]

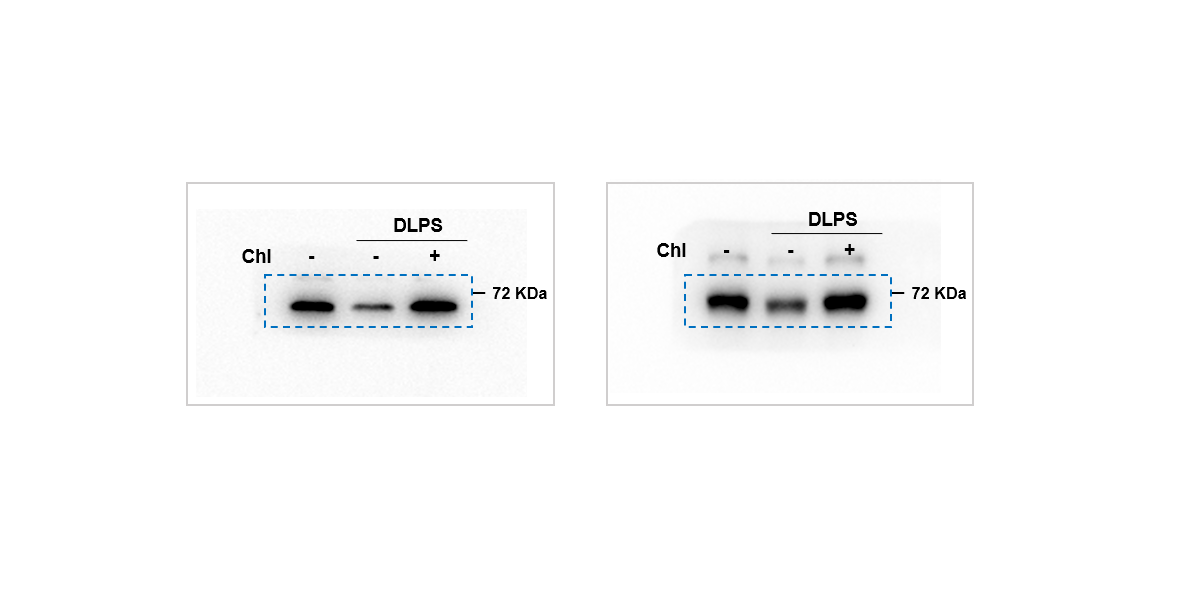

Supplement: Supplementary file 11 — Figure EV4 Source Data [file 44319_2026_829_MOESM11_ESM.zip › Figure EV4/EV4/A/p-S6K.png]

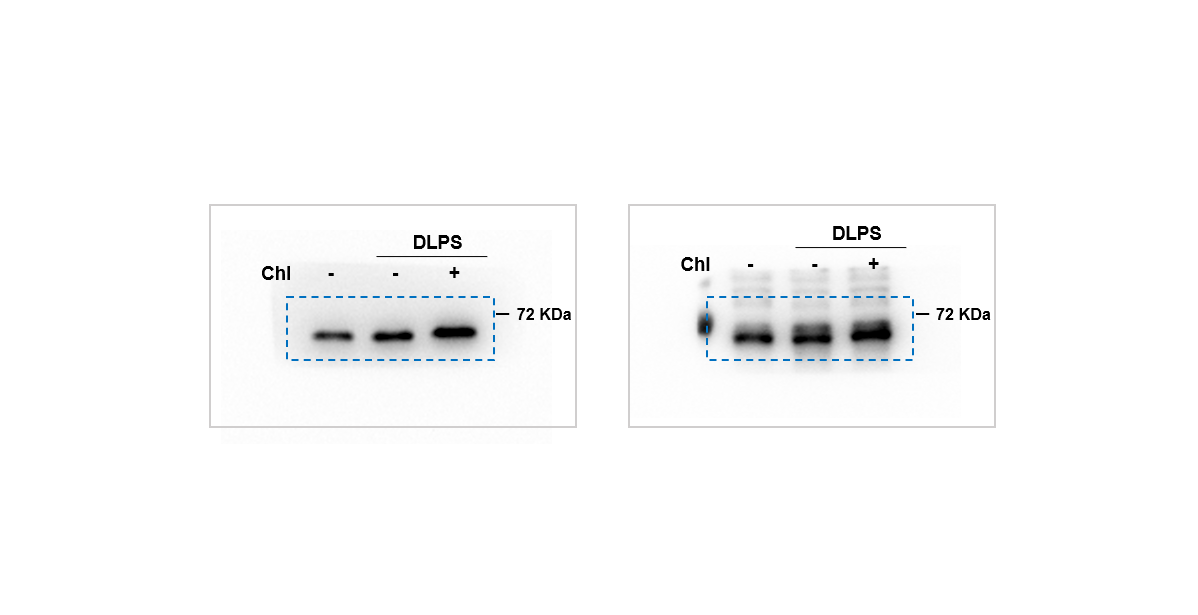

Supplement: Supplementary file 11 — Figure EV4 Source Data [file 44319_2026_829_MOESM11_ESM.zip › Figure EV4/EV4/A/S6K.png]

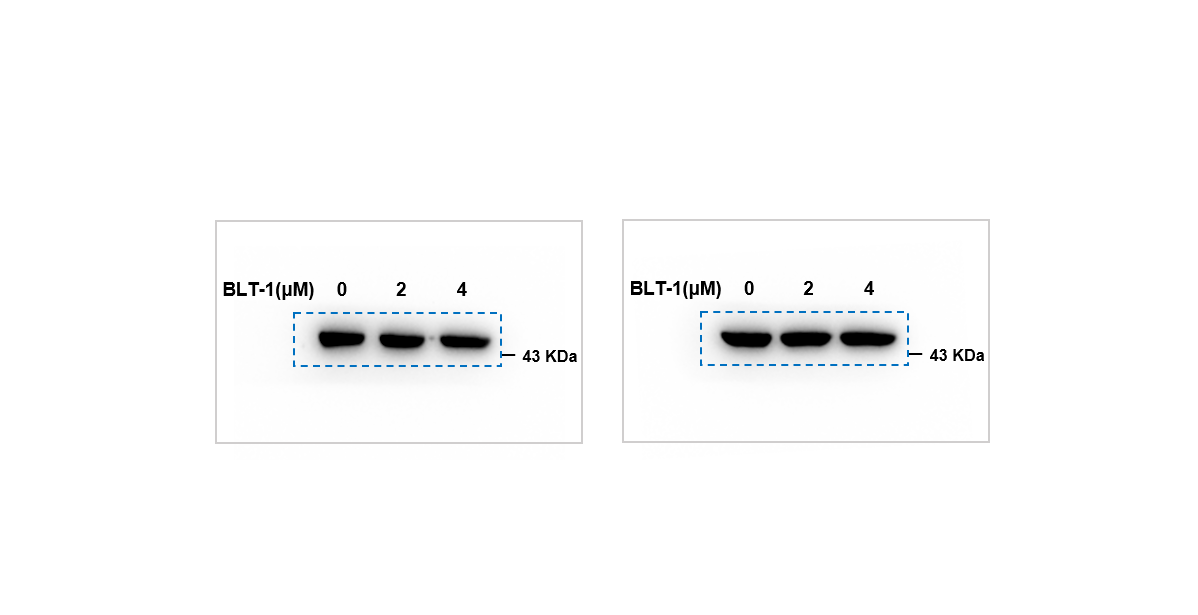

Supplement: Supplementary file 11 — Figure EV4 Source Data [file 44319_2026_829_MOESM11_ESM.zip › Figure EV4/EV4/B/ACTIN.png]

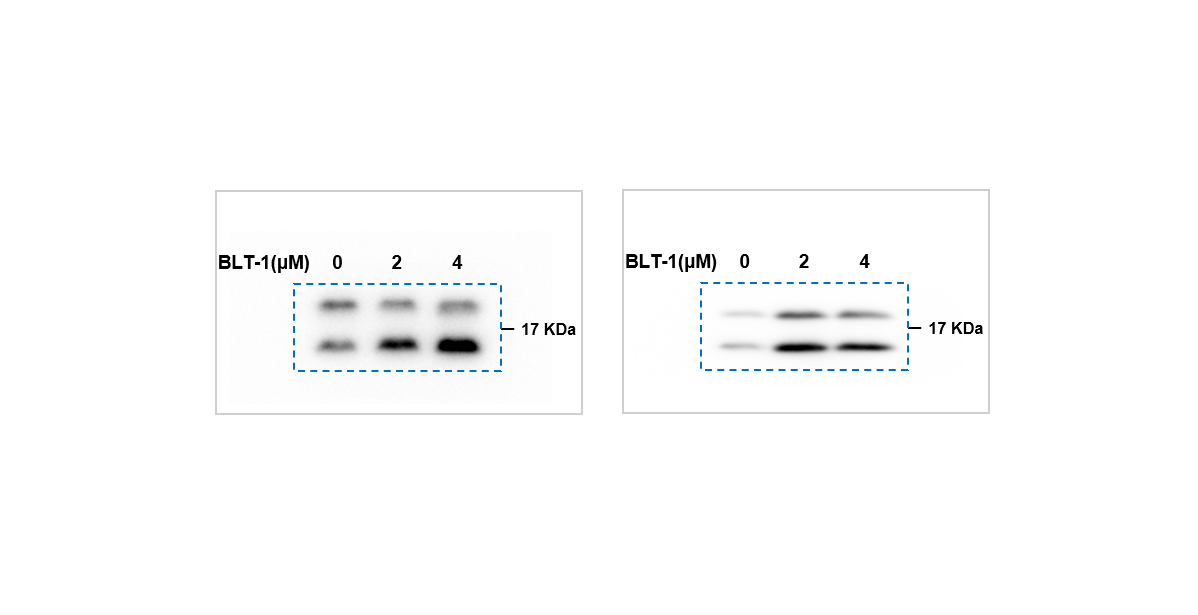

Supplement: Supplementary file 11 — Figure EV4 Source Data [file 44319_2026_829_MOESM11_ESM.zip › Figure EV4/EV4/B/LC3.png]

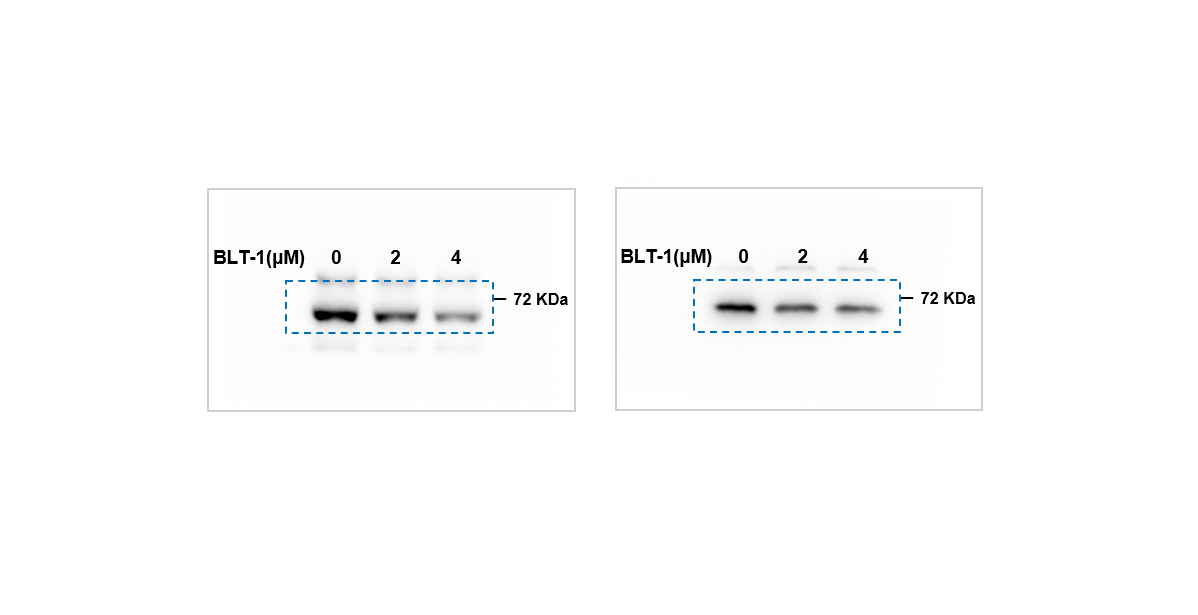

Supplement: Supplementary file 11 — Figure EV4 Source Data [file 44319_2026_829_MOESM11_ESM.zip › Figure EV4/EV4/B/p-S6K.png]

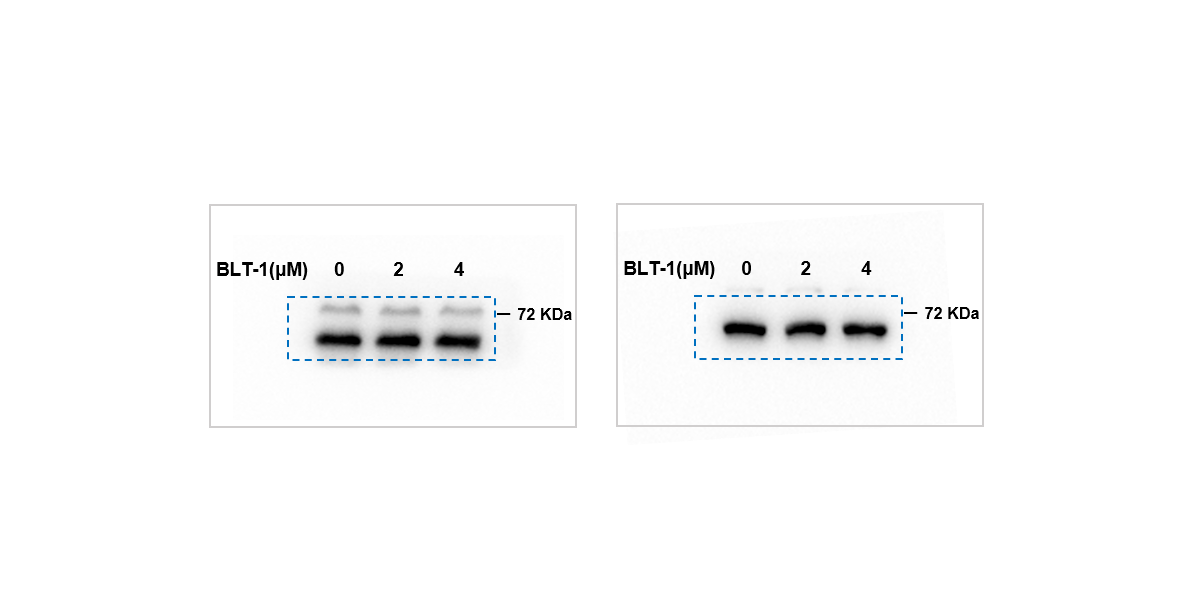

Supplement: Supplementary file 11 — Figure EV4 Source Data [file 44319_2026_829_MOESM11_ESM.zip › Figure EV4/EV4/B/S6K.png]

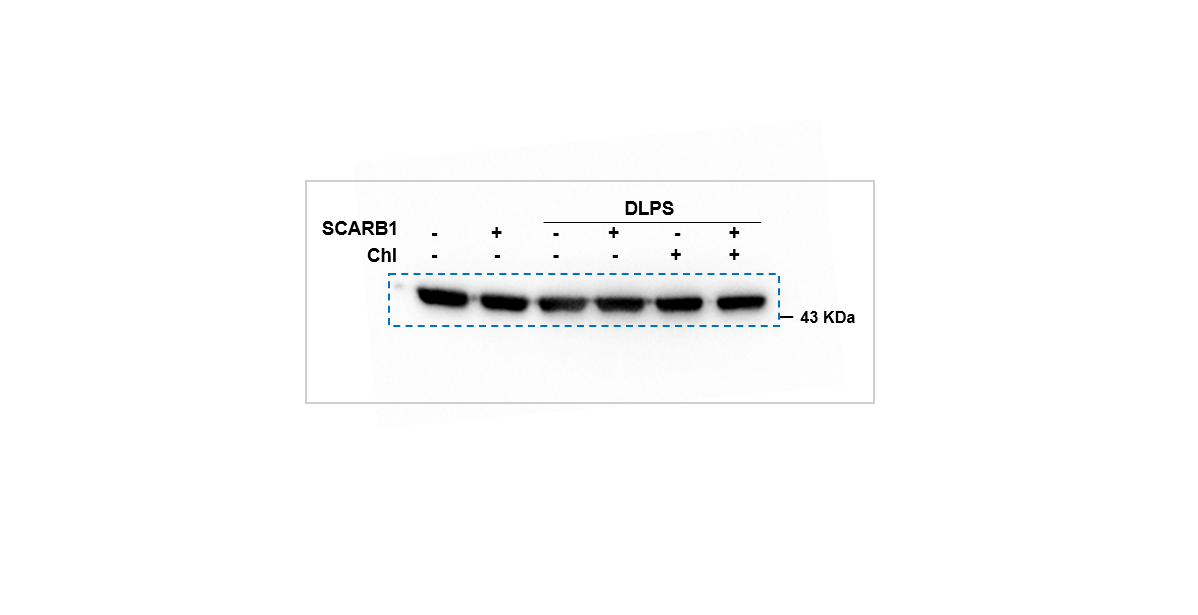

Supplement: Supplementary file 11 — Figure EV4 Source Data [file 44319_2026_829_MOESM11_ESM.zip › Figure EV4/EV4/C/ACTIN.png]

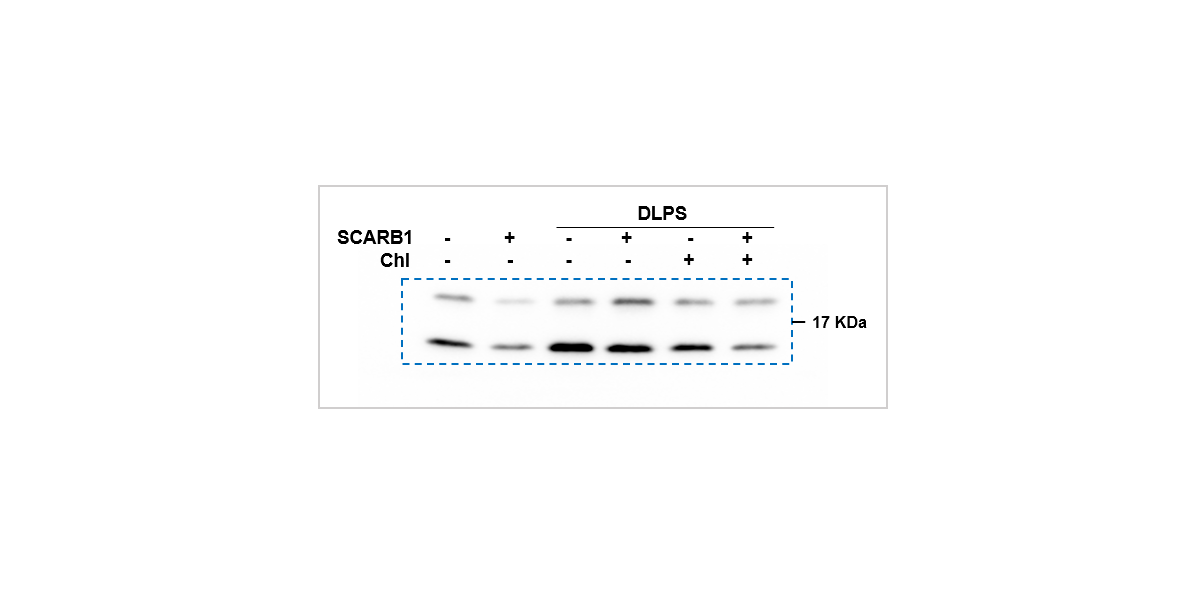

Supplement: Supplementary file 11 — Figure EV4 Source Data [file 44319_2026_829_MOESM11_ESM.zip › Figure EV4/EV4/C/LC3.png]

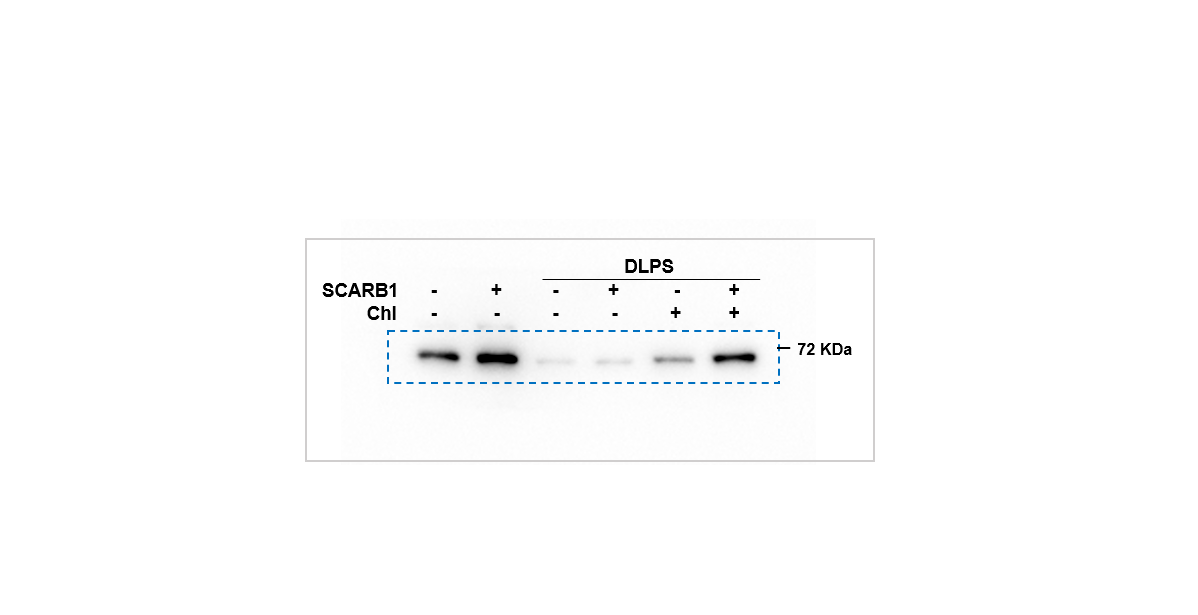

Supplement: Supplementary file 11 — Figure EV4 Source Data [file 44319_2026_829_MOESM11_ESM.zip › Figure EV4/EV4/C/p-S6K.png]

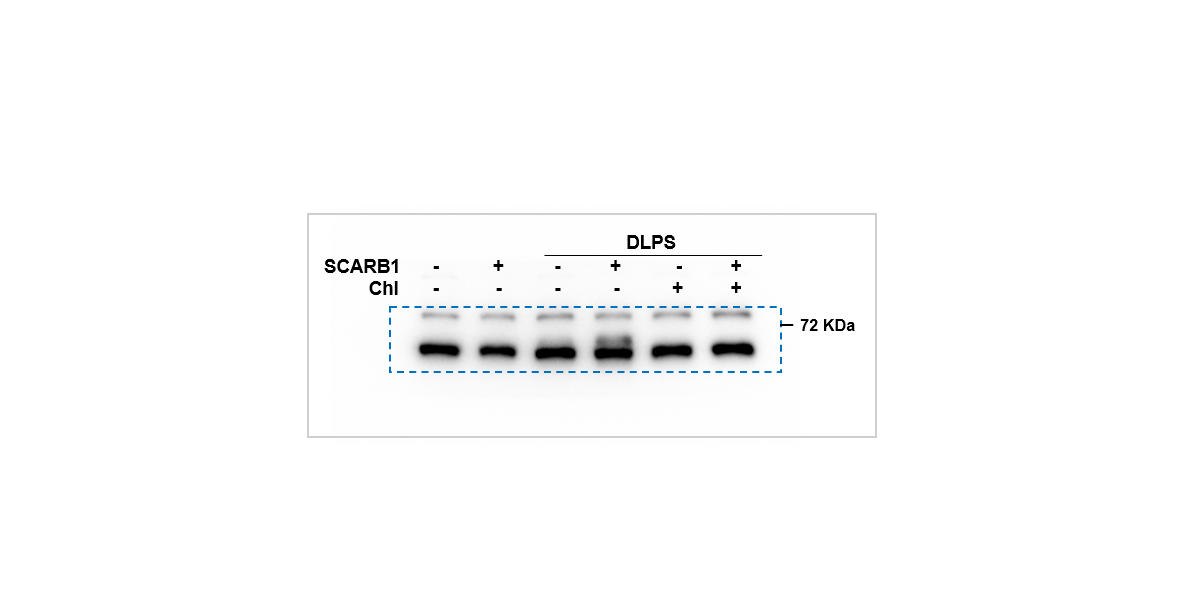

Supplement: Supplementary file 11 — Figure EV4 Source Data [file 44319_2026_829_MOESM11_ESM.zip › Figure EV4/EV4/C/S6K.png]

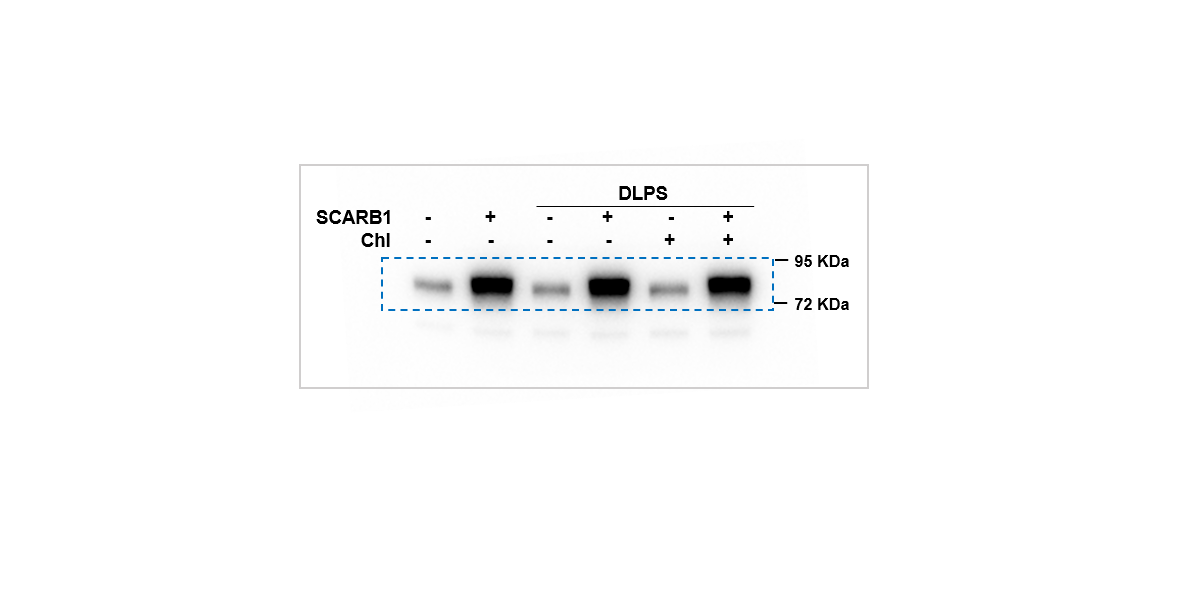

Supplement: Supplementary file 11 — Figure EV4 Source Data [file 44319_2026_829_MOESM11_ESM.zip › Figure EV4/EV4/C/SCARB1.png]

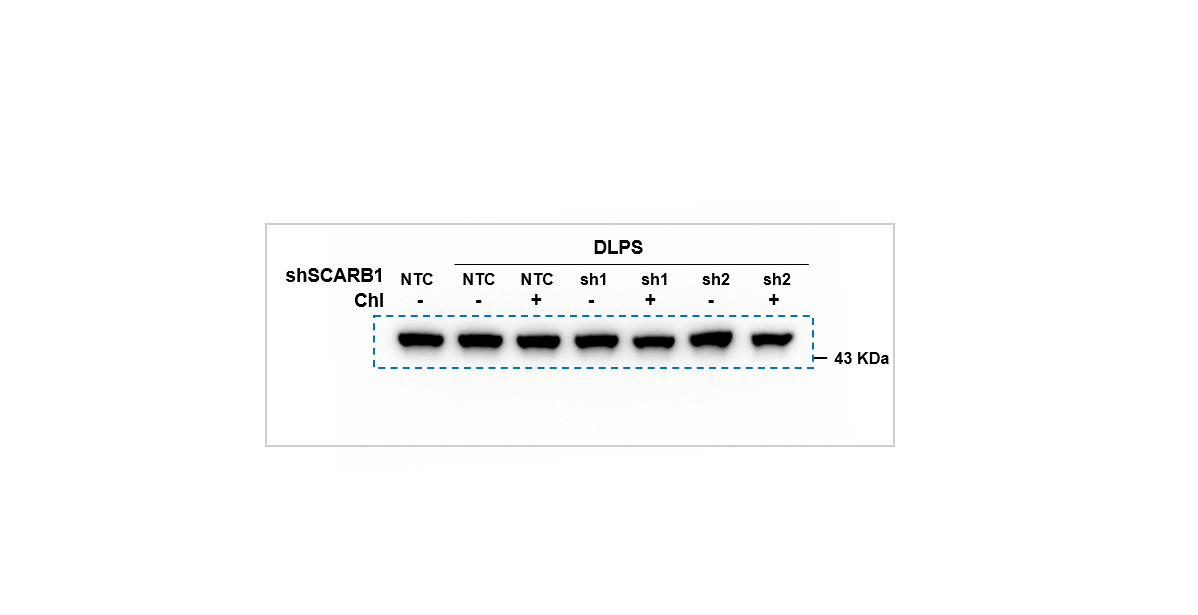

Supplement: Supplementary file 11 — Figure EV4 Source Data [file 44319_2026_829_MOESM11_ESM.zip › Figure EV4/EV4/D/ACTIN.png]

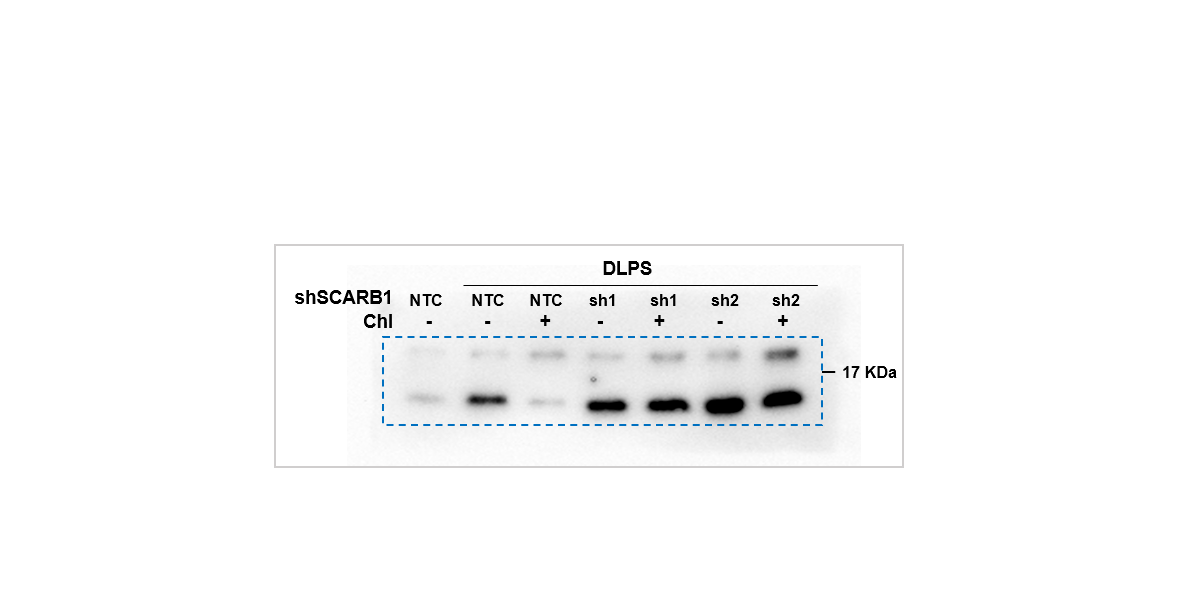

Supplement: Supplementary file 11 — Figure EV4 Source Data [file 44319_2026_829_MOESM11_ESM.zip › Figure EV4/EV4/D/LC3.png]

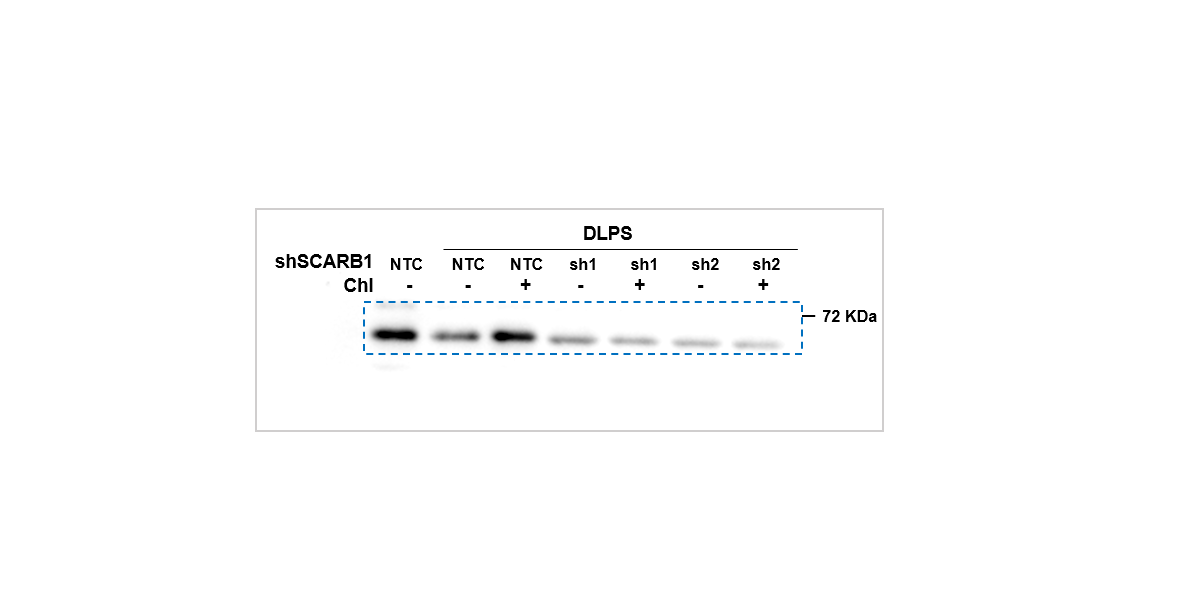

Supplement: Supplementary file 11 — Figure EV4 Source Data [file 44319_2026_829_MOESM11_ESM.zip › Figure EV4/EV4/D/p-S6K.png]

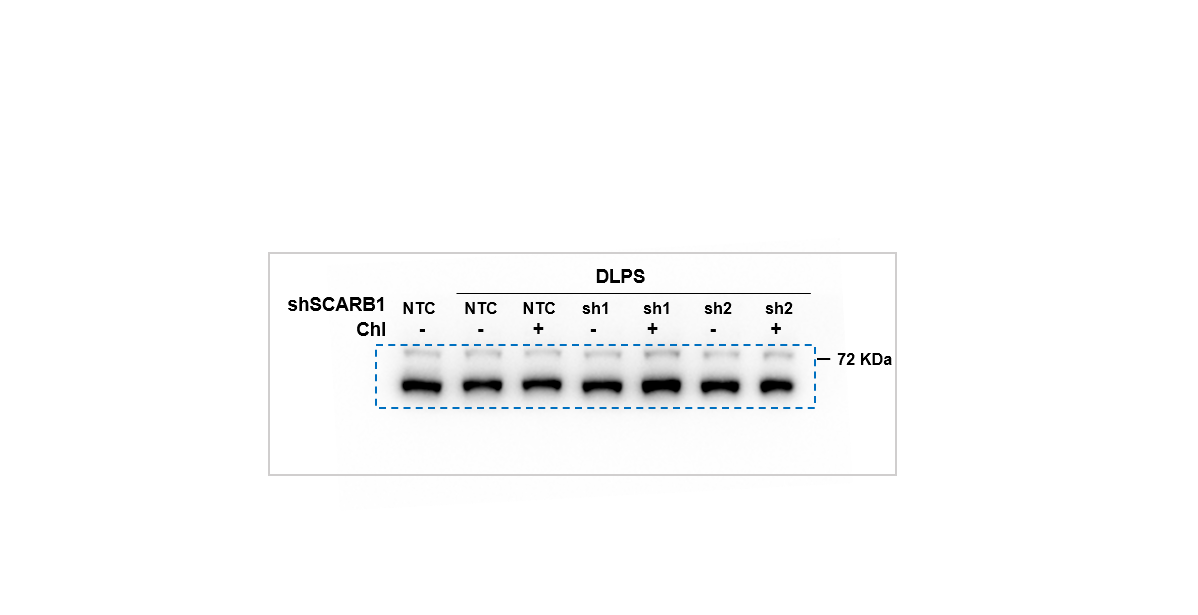

Supplement: Supplementary file 11 — Figure EV4 Source Data [file 44319_2026_829_MOESM11_ESM.zip › Figure EV4/EV4/D/S6K.png]

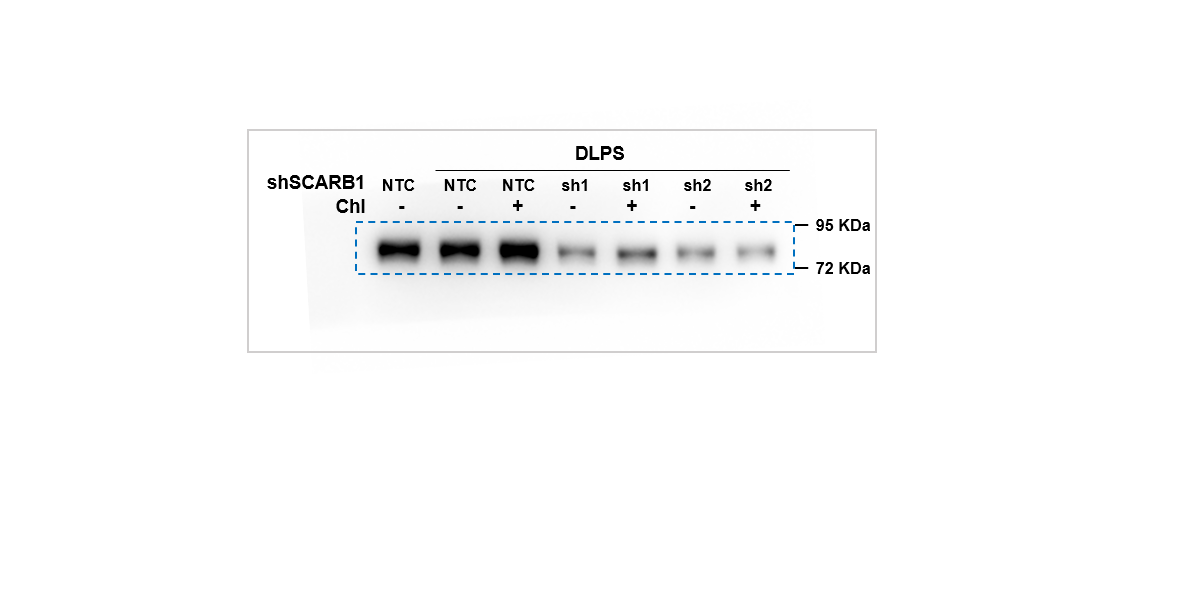

Supplement: Supplementary file 11 — Figure EV4 Source Data [file 44319_2026_829_MOESM11_ESM.zip › Figure EV4/EV4/D/SCARB1.png]

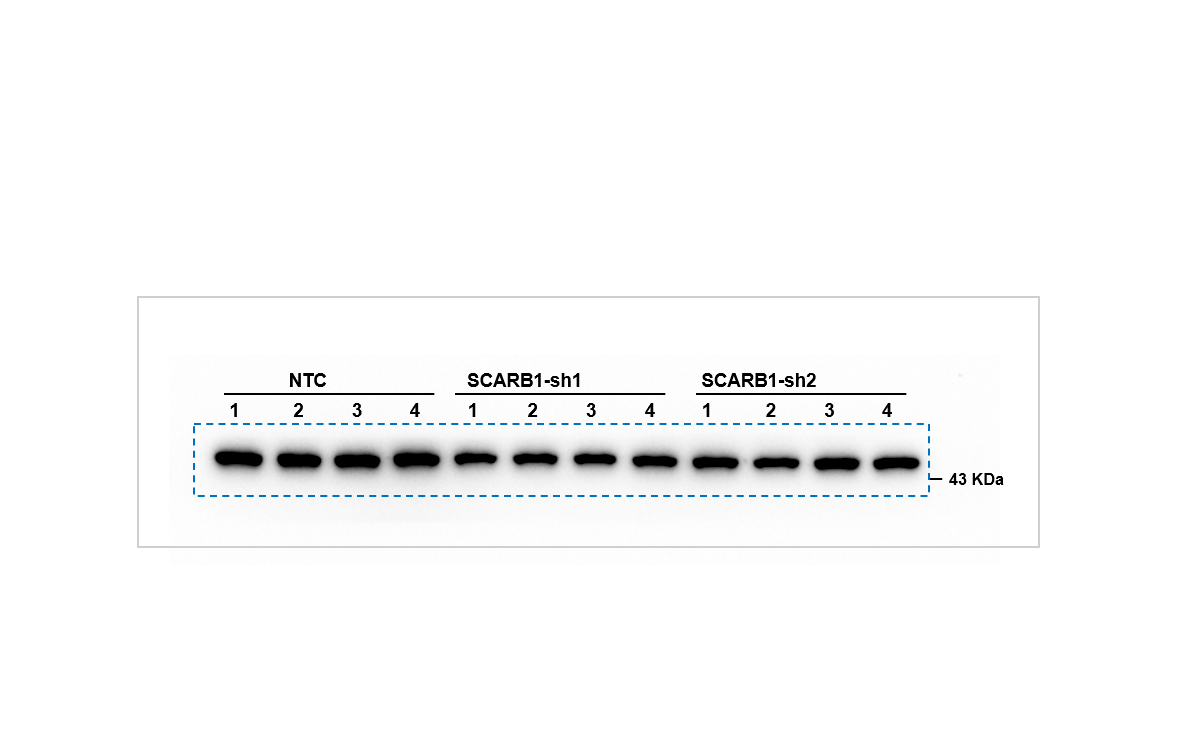

Supplement: Supplementary file 12 — Figure EV5 Source Data [file 44319_2026_829_MOESM12_ESM.zip › Figure EV5/EV/A/ACTIN.png]

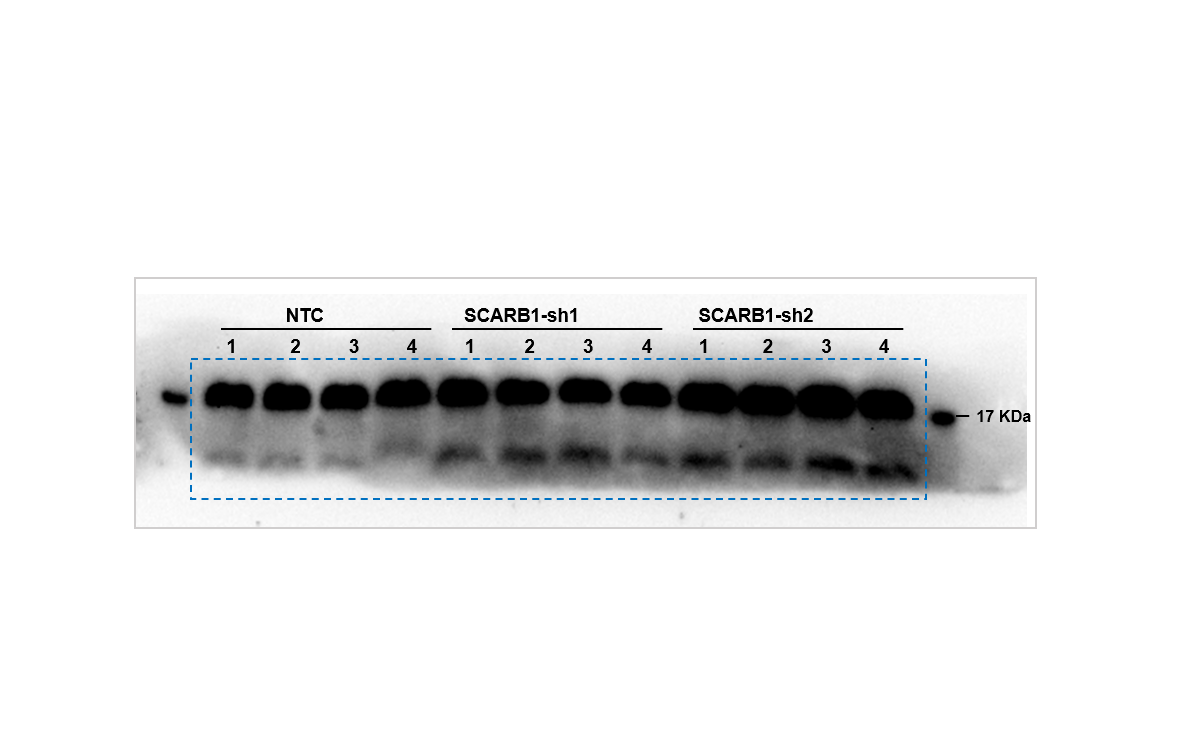

Supplement: Supplementary file 12 — Figure EV5 Source Data [file 44319_2026_829_MOESM12_ESM.zip › Figure EV5/EV/A/LC3.png]

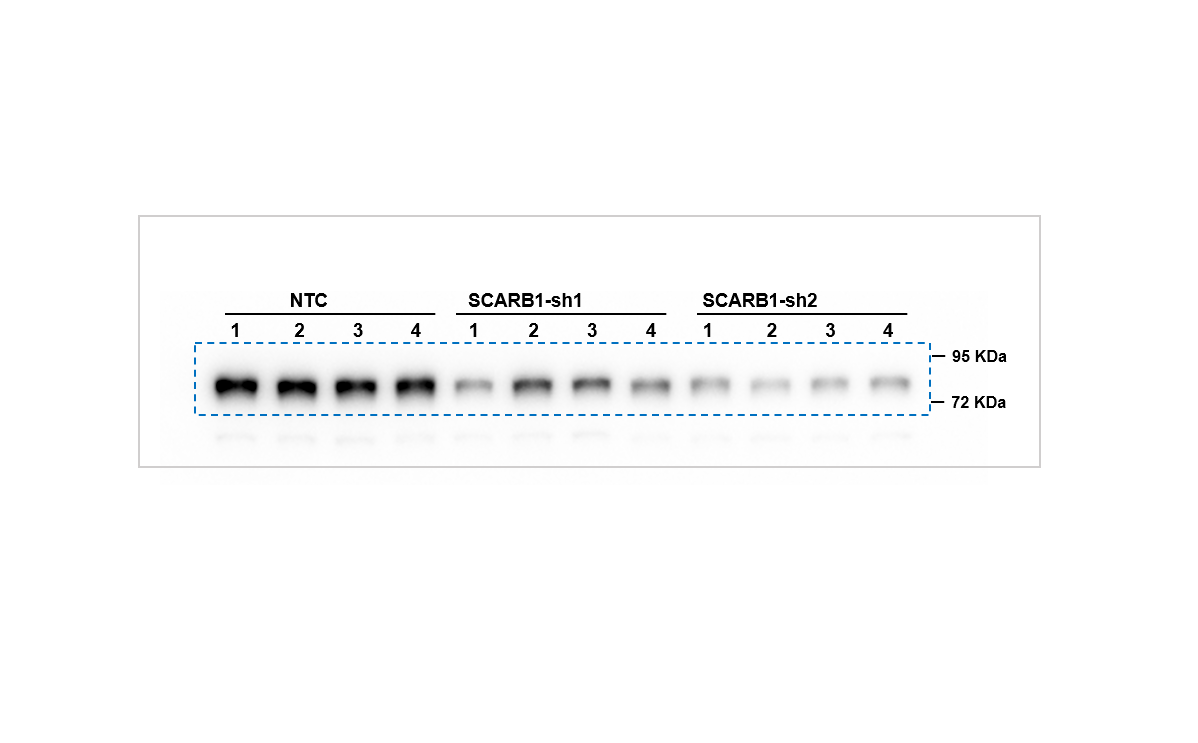

Supplement: Supplementary file 12 — Figure EV5 Source Data [file 44319_2026_829_MOESM12_ESM.zip › Figure EV5/EV/A/SCARB1.png]
