# Supplementary material for: Lactate-mediated cholesterol uptake promotes liver cancer progression via the SCARB1-autophagy axis
Source: EMBO Rep. 2026 Jun 10;27(14):4141–65. doi: 10.1038/s44319-026-00829-x (PMC13400630; doi:10.1038/s44319-026-00829-x)
Supplement: Supplementary file 13 — Expanded View Figures [file 44319_2026_829_MOESM13_ESM.pdf]

Expanded View Figures

Figure EV1. Lactate upregulates SCARB1 to increase cholesterol uptake under hypoxia.

(**A**) Hep3B and PLC cells were cultured under normoxia (N), hypoxia (H), or hypoxia with 10 mM 2-DG (H + 2-DG) for 48 h. Cellular cholesterol content was measured. H vs. N: $P = 2.4e\text{-}5$; H vs. H + 2-DG: $P = 3.0e\text{-}6$. (**B**) mRNA levels of cholesterol metabolism-related genes were determined by RT–qPCR in Hep3B cells cultured under normoxia, hypoxia, or hypoxia with 20 mM sodium oxamate (H + SO) for 48 h. H vs. N: $P = 3.8e\text{-}6$; H vs. H + SO: $P = 2.6e\text{-}5$. (**C**) Hep3B cells were cultured under normoxia, hypoxia, or hypoxia with 20 mM sodium oxamate (H + SO) for 48 h, followed by measurement of DiI–HDL uptake. Scale bar: 50 μm. $P = 1.1e\text{-}6$. (**D**) Hep3B and PLC cells were cultured under normoxia, hypoxia, or hypoxia with 10 mM 2-DG for 48 h. SCARB1 expression was detected by western blotting and qRT–PCR. H vs. N: $P = 4.8e\text{-}5$ (Hep3B) and 1.1e-5 (PLC); H vs. H + 2-DG: $P = 1.5e\text{-}5$ (Hep3B) and 5.2e-5 (PLC). (**E**) Western blot analysis of SCARB1 protein expression in Hep3B cells expressing MCT1 knockdown. (**F**, **G**) Western blot analysis of SCARB1 protein expression in Hep3B cells expressing MCT4 knockdown (**F**) or overexpression (**G**). (**H**) Western blot analysis of SCARB1 expression in Hep3B and PLC cells with SCARB1 overexpression or knockdown. Immunoblots are representative of three independent experiments (**D**, **H**). Data are presented as the mean ± s.d. of three independent experiments. Statistical significance was determined by one-way ANOVA (**A**, **C**, **D**) and two-way ANOVA (**B**). Source data are available online for this figure.

© The Author(s)

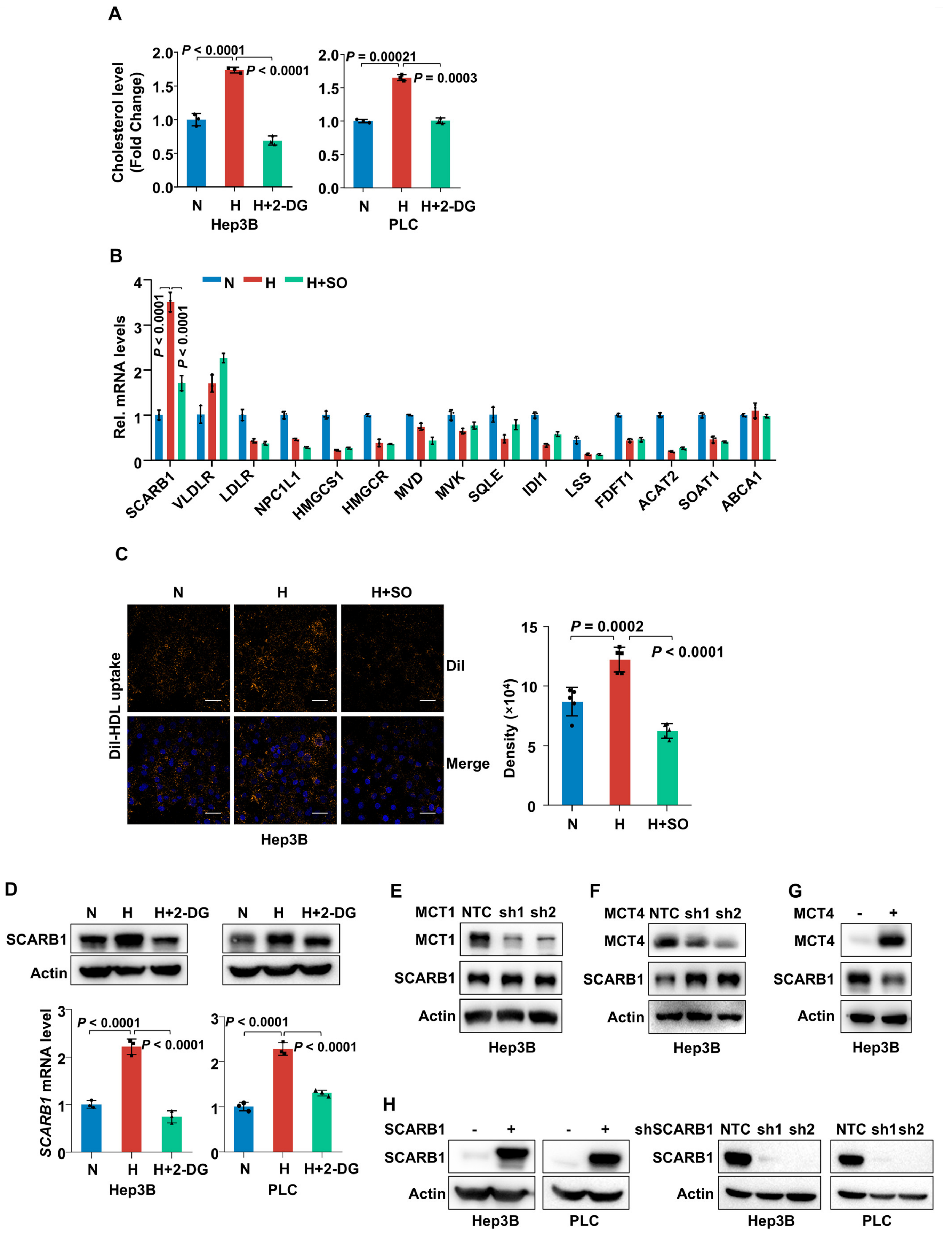
A
Cholesterol level (Fold Change)
P < 0.0001
P < 0.0001
N H H+2-DG
Hep3B
P = 0.00021
P = 0.0003
N H H+2-DG
PLC
B
N H H+SO
Rel. mRNA levels
P < 0.0001
P < 0.0001
SCARB1 VLDLR LDLR NPC1L1 HMGCS1 HMGCR MVD MVK SQLE IDI1 LSS FDFT1 ACAT2 SOAT1 ABCA1
C
N H H+SO
DiI-HDL uptake
DiI
Merge
Hep3B
P = 0.0002
P < 0.0001
Density (×10⁴)
N H H+SO
D
N H H+2-DG
SCARB1
Actin
SCARB1 mRNA level
P < 0.0001
P < 0.0001
N H H+2-DG
Hep3B
PLC
E
MCT1 NTC sh1 sh2
MCT1
SCARB1
Actin
Hep3B
F
MCT4 NTC sh1 sh2
MCT4
SCARB1
Actin
Hep3B
G
MCT4 - +
MCT4
SCARB1
Actin
Hep3B
H
SCARB1 - + - +
SCARB1
Actin
Hep3B PLC
shSCARB1 NTC sh1 sh2
NTC sh1 sh2
SCARB1
Actin
Hep3B PLC

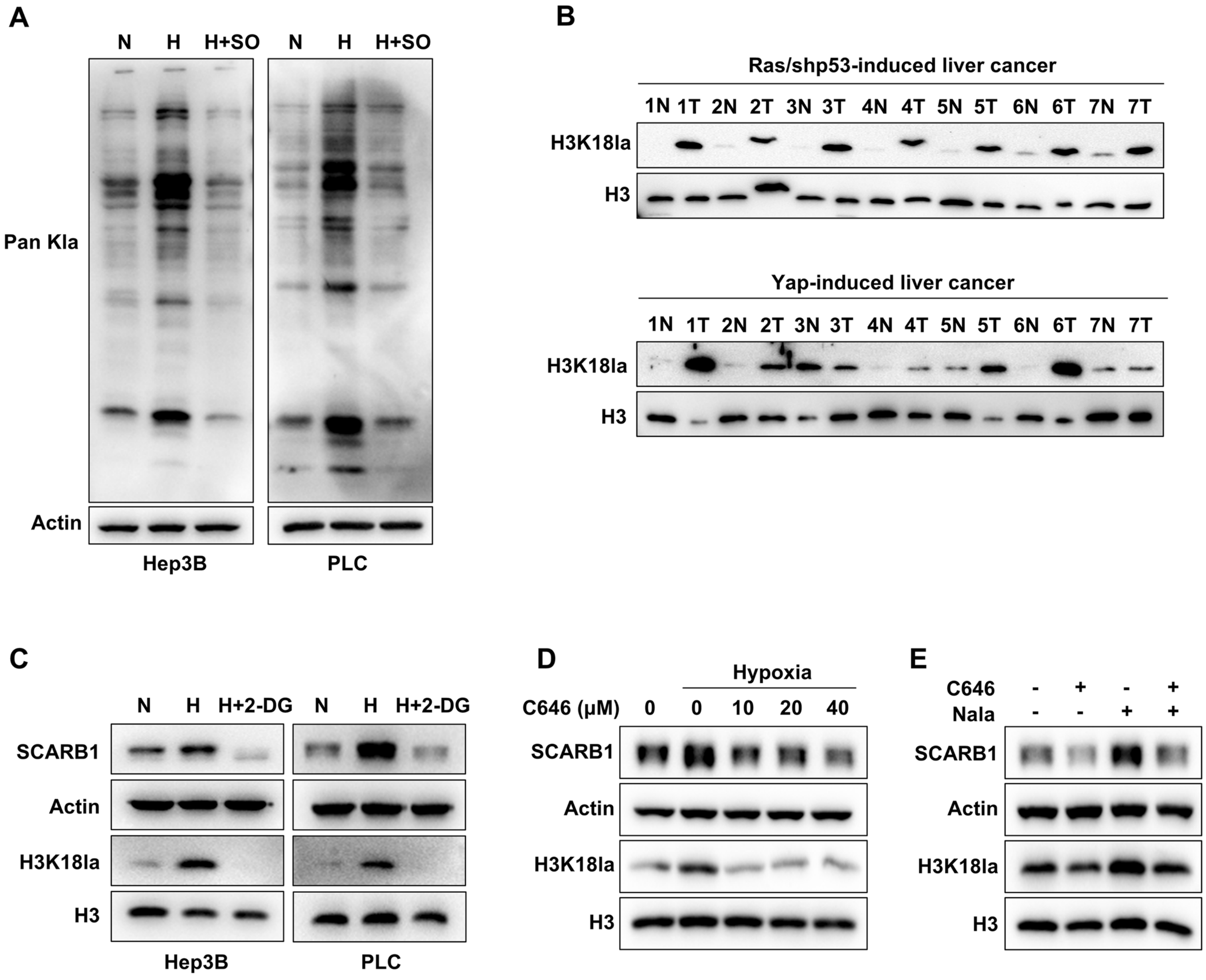

Figure EV2. Histone lactylation activates SCARB1 transcription.

(**A**) Western blot analysis of panlactylation in Hep3B and PLC cells cultured under normoxia, hypoxia, or hypoxia with 20 mM sodium oxamate for 48 h. (**B**) Western blot analysis of H3K18la expression in paired adjacent noncancerous liver tissues (N) and cancerous liver tissues (T) in YAP-5SA- or Ras/shp53-induced mouse HCC. (**C**) Western blot analysis of H3K18la and SCARB1 expression in Hep3B and PLC cells cultured under normoxia, hypoxia, or hypoxia with 10 mM 2-DG for 48 h. Immunoblots are representative of three independent experiments (**A**, **C**). (**D**) Western blot analysis of H3K18la and SCARB1 expression in Hep3B cells treated with the p300 inhibitor C646 and cultured under normoxia or hypoxia for 48 h. (**E**) Western blot analysis of H3K18la and SCARB1 expression in Hep3B cells treated with 40 μM C646, 20 mM sodium lactate, or a combination of sodium lactate and C646 and cultured under hypoxia for 48 h. Immunoblots are representative of three independent experiments (**A**, **C–E**). Source data are available online for this figure.

 © The Author(s)

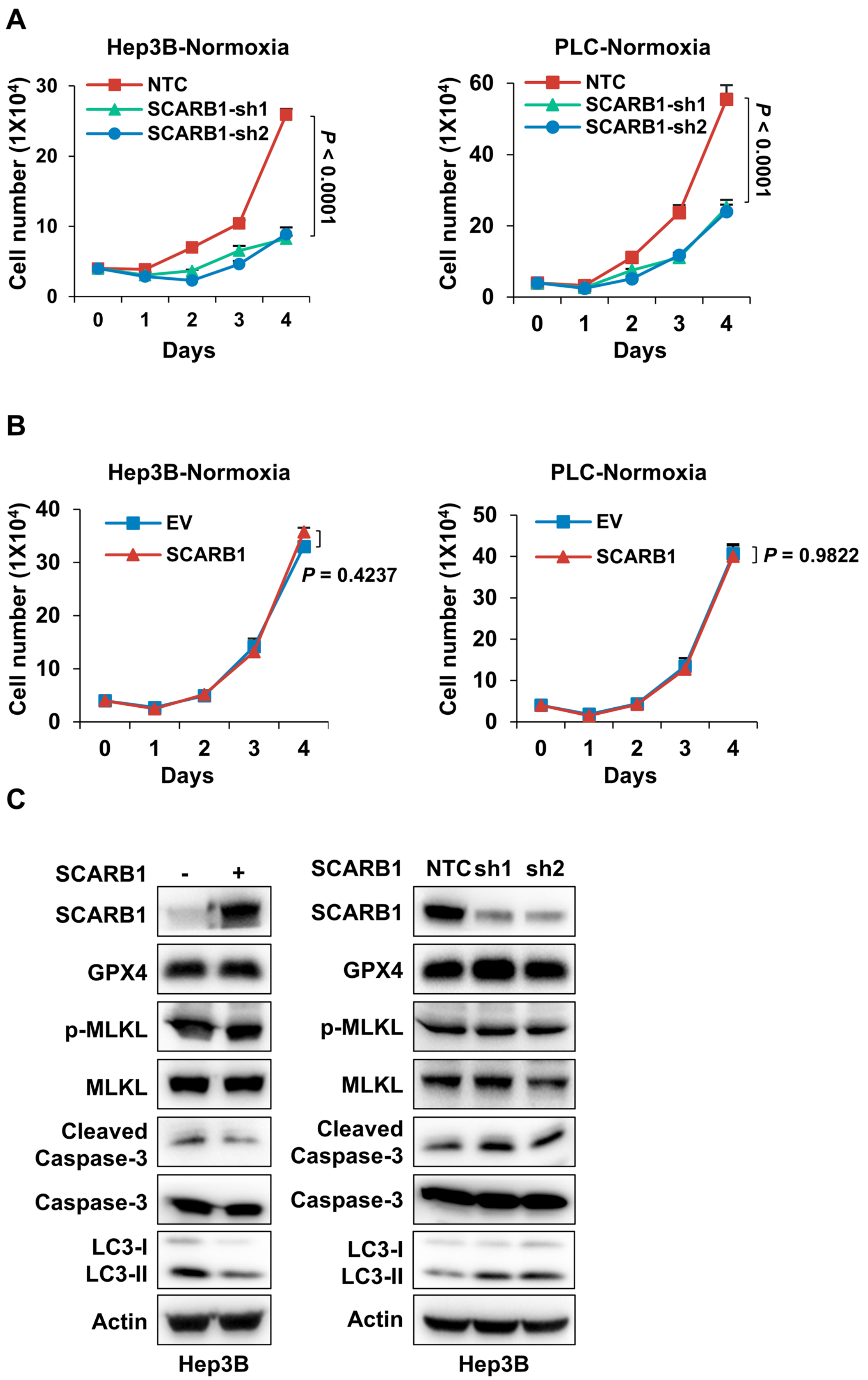
A
Hep3B-Normoxia
Cell number (1X10^4)
30
20
10
0
NTC
SCARB1-sh1
SCARB1-sh2
P < 0.0001
0 1 2 3 4
Days
PLC-Normoxia
60
40
20
0
NTC
SCARB1-sh1
SCARB1-sh2
P < 0.0001
B
Hep3B-Normoxia
40
30
EV
SCARB1
P = 0.4237
PLC-Normoxia
50
EV
SCARB1
P = 0.9822
C
SCARB1 - +
SCARB1
GPX4
p-MLKL
MLKL
Cleaved Caspase-3
Caspase-3
LC3-I
LC3-II
Actin
Hep3B
SCARB1 NTC sh1 sh2

Figure EV3. SCARB1 promotes cell growth by inhibiting autophagy.

(**A**, **B**) Growth curves were generated for Hep3B and PLC cells expressing shNTC or shSCARB1 (**A**) and for cells expressing SCARB1 (**B**) under normoxia. (**A**) NTC vs. shSCARB1#1: $P = 3.5e\text{-}11$ (Hep3B) and 4.6e-11 (PLC); NTC vs. shSCARB1#2: $P = 1.3e\text{-}15$ (Hep3B) and 1.6e-10 (PLC). (**C**) Western blot analysis of GPX4, phosphor-MLKL, MLKL, cleaved caspase-3, Caspase-3, and LC3-I/II in Hep3B cells expressing SCARB1 overexpression or knockdown under hypoxia. Data are presented as the mean ± s.d. of three independent experiments. Statistical significance was determined by two-way ANOVA (**A**, **B**).Source data are available online for this figure.

 © The Author(s)

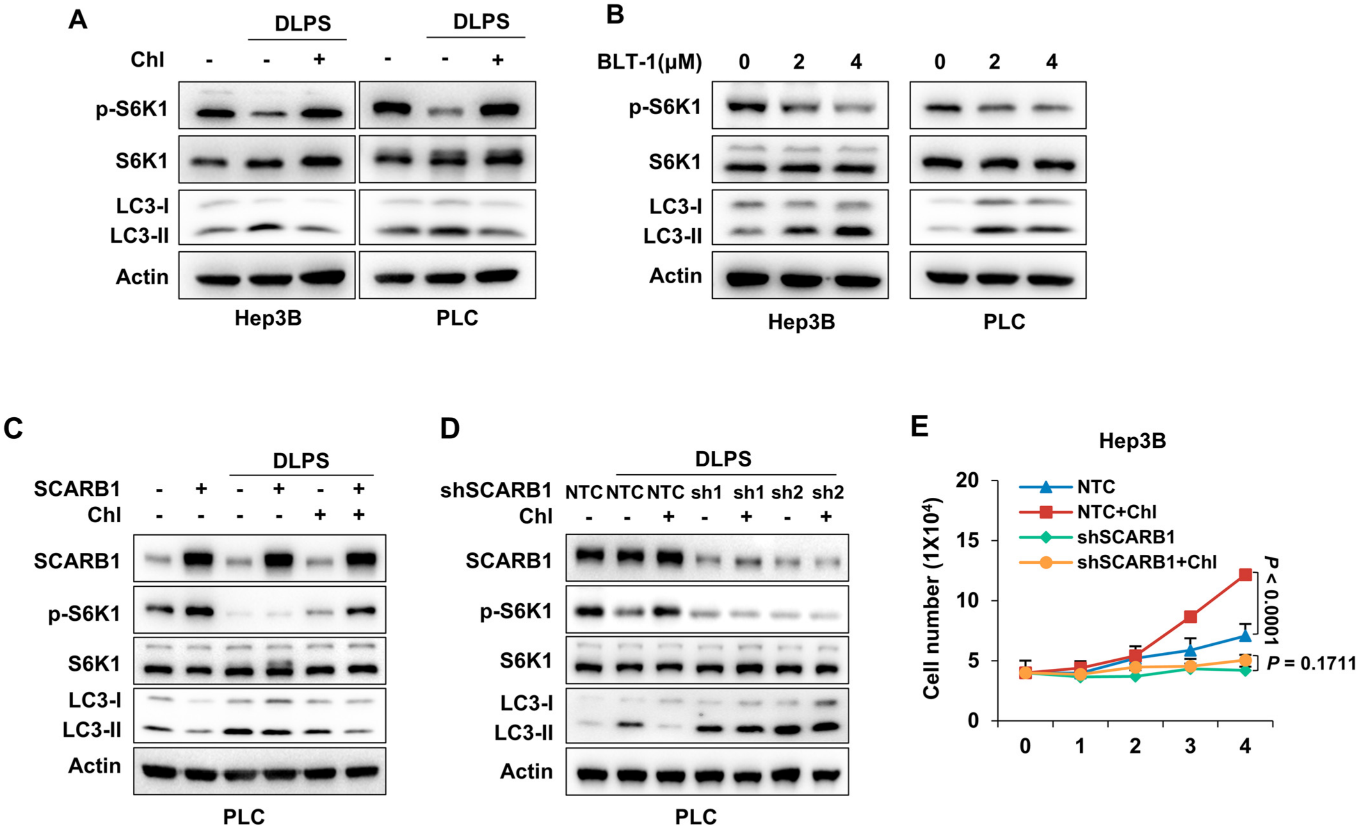

Figure EV4. SCARB1 activates mTORC1 signalling through cholesterol uptake.

(**A**) Western blot analysis of SCARB1, p-S6K1, S6K1, and LC3-I/II in Hep3B cells cultured in 10% FBS or 10% DLPS in the absence or presence of 10 μg/mL cholesterol for 2 h under hypoxia. (**B**) Western blot analysis of SCARB1, p-S6K1, S6K1, and LC3-I/II in Hep3B and PLC cells treated with BLT-1 under hypoxia for 48 h. (**C**, **D**) PLC cells expressing SCARB1 (**C**) or shSCARB1 (**D**) were cultured in 10% FBS or 10% DLPS in the absence or presence of 10 μg/mL cholesterol under hypoxia. SCARB1, p-S6K1, S6K1, and LC3-I/II protein levels were detected by Western blotting. (**E**) Growth curves were generated for Hep3B cells expressing shNTC or shSCARB1 and treated with or without 10 μg/mL cholesterol under hypoxia. $P = 4.3e\text{-}10$. Immunoblots are representative of three independent experiments (**A**–**D**). Data are presented as the mean ± s.d. of three independent experiments. Statistical significance was determined by two-way ANOVA (**E**). Source data are available online for this figure.

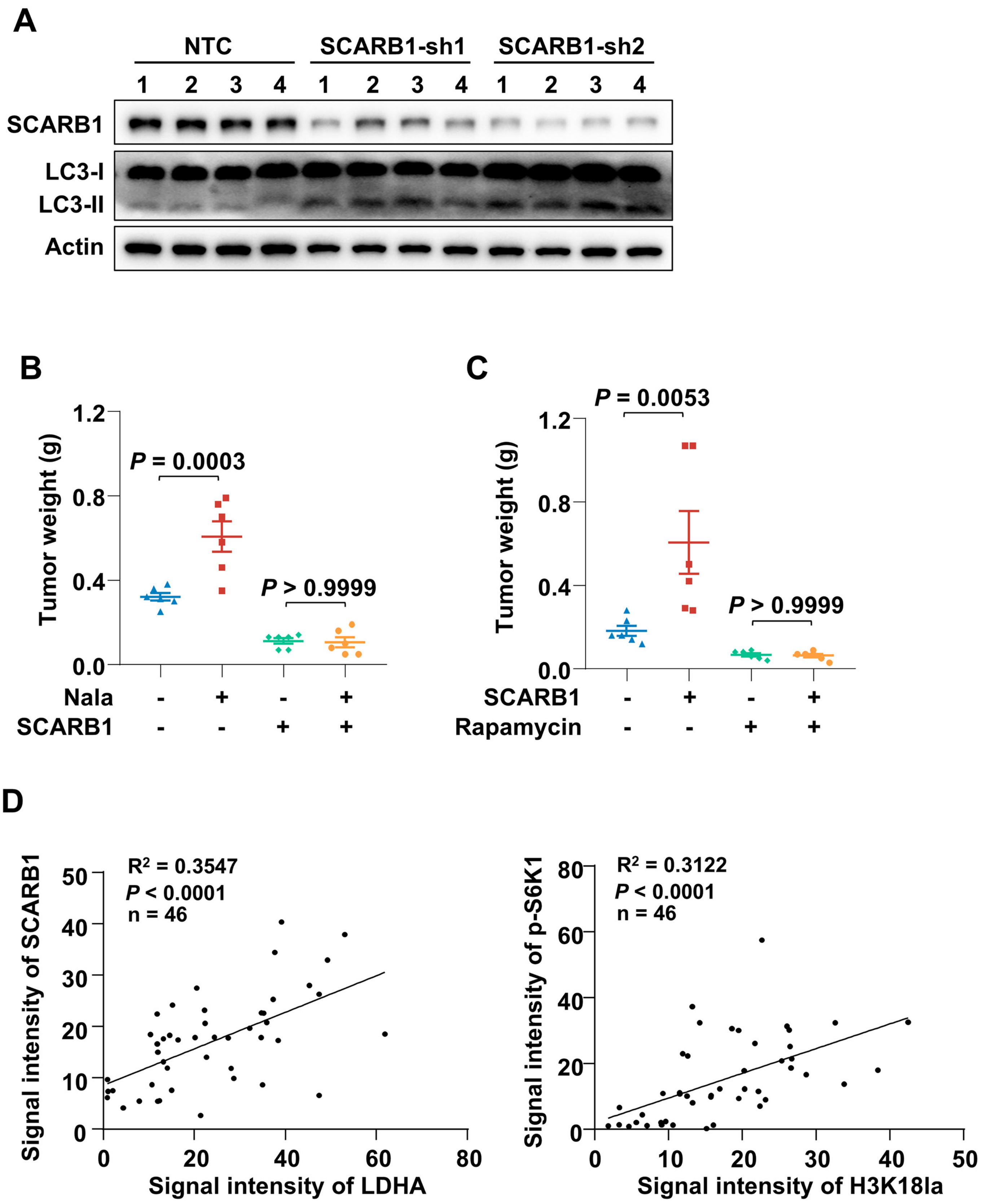
A
NTC
SCARB1-sh1
SCARB1-sh2
1 2 3 4 1 2 3 4 1 2 3 4
SCARB1
LC3-I
LC3-II
Actin
B
Tumor weight (g)
P = 0.0003
P > 0.9999
Nala
SCARB1
C
Tumor weight (g)
P = 0.0053
P > 0.9999
SCARB1
Rapamycin
D
R² = 0.3547
P < 0.0001
n = 46
Signal intensity of SCARB1
Signal intensity of LDHA
R² = 0.3122
P < 0.0001
n = 46
Signal intensity of p-S6K1
Signal intensity of H3K18la

 © The Author(s)

Figure EV5. SCARB1 promotes tumour progression in vivo.

(**A**) Protein levels of SCARB1 and LC3-I/II were detected by Western blot using lysates from four independent tumours of each group, as shown in Fig. 5A. (**B**) Hep3B cells expressing shNTC or shSCARB1 were injected subcutaneously into nude mice, and tumour mass was measured at the end of the experiment. (**C**) Hep3B cells expressing EV or SCARB1 were injected subcutaneously into nude mice, and tumour mass was measured at the end of the experiment. (**D**) Analysis of the correlation between the protein levels of LDHA and SCARB1, as well as H3K18la and p-S6K1 in HCC specimens. Pearson correlation analyses were performed. $P = 1.3e-5$ (left), $P = 5.4e-5$ (right). Data are presented as the mean ± s.e.m (**B**, **C**). Statistical significance was determined by one-way ANOVA (**B**, **C**). Source data are available online for this figure.

© The Author(s)
