# Supplementary material for: Lactate-mediated cholesterol uptake promotes liver cancer progression via the SCARB1-autophagy axis
Source: EMBO Rep. 2026 Jun 10;27(14):4141–65. doi: 10.1038/s44319-026-00829-x (PMC13400630; doi:10.1038/s44319-026-00829-x)
Supplement: Supplementary file 2 — Peer Review File [file 44319_2026_829_MOESM2_ESM.pdf]

EMBO reports

Lactate-mediated cholesterol uptake promotes liver cancer progression via the SCARB1-autophagy axis

Jiamin Chen, Mingyue Zhao, Guanxu Ji, Kecheng Liu, Shengqi Shen, Shi-Ting Li, Jin Cai, Linchong Sun, Jin Li, Ping Gao, and Tong Zhang

Corresponding author(s): Tong Zhang (tzhang11@mail.ustc.edu.cn) , Ping Gao (pgao2@ustc.edu.cn), Jin Li (JL@gzlab.ac.cn)

Review Timeline:

Submission Date:	24th Sep 25
Editorial Decision:	20th Nov 25
Revision Received:	17th Feb 26
Editorial Decision:	7th Apr 26
Revision Received:	14th Apr 26
Accepted:	22nd May 26

Editor: Kurt Weir

Transaction Report:

(Note: With the exception of the correction of typographical or spelling errors that could be a source of ambiguity, letters and reports are not edited. Depending on transfer agreements, referee reports obtained elsewhere may or may not be included in this compilation. Referee reports are anonymous unless the Referee chooses to sign their reports.)

1st Editorial Decision **20th Nov 2025**

Dear Prof. Gao

Thank you for the submission of your research manuscript to our journal. We have now received the full set of referee reports that is copied below.

As you will see, the referees acknowledge that the findings are interesting and that the conclusions are overall supported by the data presented but they also raise a number of concerns and have suggestions how to further strengthen the data, particularly focused on situating the mechanism described in your paper within the relevant literature.

Given these constructive comments, we would like to invite you to revise your manuscript with the understanding that the referee concerns (as detailed above and in their reports) must be fully addressed and their suggestions taken on board. Please address all referee concerns in a complete point-by-point response. Acceptance of the manuscript will depend on a positive outcome of a second round of review. It is EMBO Reports policy to allow a single round of revision only and acceptance or rejection of the manuscript will therefore depend on the completeness of your responses included in the next, final version of the manuscript.

We realize that it is difficult to revise to a specific deadline. In the interest of protecting the conceptual advance provided by the work, we recommend a revision within 3 months (February 20th). Please discuss the revision progress ahead of this time with the editor if you require more time to complete the revisions.

I am also happy to discuss the revision further via e-mail or a video call, if you wish.

You can either publish the study as a short report or as a full article. For short reports, the revised manuscript should not exceed 27,000 characters (including spaces but excluding materials & methods and references) and 5 main plus 5 expanded view figures. The results and discussion sections must further be combined, which will help to shorten the manuscript text by eliminating some redundancy that is inevitable when discussing the same experiments twice. For a normal article there are no length limitations, but it should have more than 5 main figures and the results and discussion sections must be separate. In both cases, the entire materials and methods must be included in the main manuscript file.

================
IMPORTANT NOTE:

We perform an initial quality control of all revised manuscripts before re-review. Your manuscript will FAIL this control and the handling will be delayed IN CASE the following APPLIES:

1) A data availability section providing access to data deposited in public databases is missing. If you have not deposited any data, please add a sentence to the data availability section that explains that.

2) Your manuscript contains statistics and error bars based on n=2. Please use scatter blots in these cases. No statistics should be calculated if n=2.

When submitting your revised manuscript, please carefully review the instructions that follow below. Failure to include requested items will delay the evaluation of your revision.

================

When submitting your revised manuscript, we will require:

1) a .docx formatted version of the manuscript text (including legends for main figures, EV figures and tables). Please make sure that the changes are highlighted to be clearly visible.

2) individual production quality figure files as .eps, .tif, .jpg (one file per figure).
Please download our Figure Preparation Guidelines (figure preparation pdf) from our Author Guidelines pages https://www.embopress.org/page/journal/14693178/authorguide for more info on how to prepare your figures.

3) a .docx formatted letter INCLUDING the reviewers' reports and your detailed point-by-point responses to their comments. As part of the EMBO Press transparent editorial process, the point-by-point response is part of the Review Process File (RPF), which will be published alongside your paper.

4) a complete author checklist, which you can download from our author guidelines (<https://www.embopress.org/page/journal/14693178/authorguide>). Please insert information in the checklist that is also

reflected in the manuscript. The completed author checklist will also be part of the RPF.

5) Please note that all corresponding authors are required to supply an ORCID ID for their name upon submission of a revised manuscript (<https://orcid.org/>). Please find instructions on how to link your ORCID ID to your account in our manuscript tracking system in our Author guidelines (<https://www.embopress.org/page/journal/14693178/authorguide#authorshipguidelines>)

6) We replaced Supplementary Information with Expanded View (EV) Figures and Tables that are collapsible/expandable online. A maximum of 5 EV Figures can be typeset. EV Figures should be cited as 'Figure EV1, Figure EV2" etc... in the text and their respective legends should be included in the main text after the legends of regular figures.

- For the figures that you do NOT wish to display as Expanded View figures, they should be bundled together with their legends in a single PDF file called *Appendix*, which should start with a short Table of Content. Appendix figures should be referred to in the main text as: "Appendix Figure S1, Appendix Figure S2" etc. See detailed instructions regarding expanded view here: <https://www.embopress.org/page/journal/14693178/authorguide#expandedview>

- Additional Tables/Datasets should be labeled and referred to as Table EV1, Dataset EV1, etc. Legends have to be provided in a separate tab in case of .xls files. Alternatively, the legend can be supplied as a separate text file (README) and zipped together with the Table/Dataset file.

7) Before submitting your revision, primary datasets (and computer code, where appropriate) produced in this study need to be deposited in an appropriate public database (see < https://www.embopress.org/page/journal/14693178/authorguide#dataavailability>).

Please remember to provide a reviewer password if the datasets are not yet public.

The accession numbers and database should be listed in a formal "Data Availability " section (placed after Materials & Method) that follows the model below (see also < https://www.embopress.org/page/journal/14693178/authorguide#dataavailability>). Please note that the Data Availability Section is restricted to new primary data that are part of this study.

Data availability

The datasets (and computer code) produced in this study are available in the following databases:

- RNA-Seq data: Gene Expression Omnibus GSE46843 (https://www.ncbi.nlm.nih.gov/geo/query/acc.cgi?acc=GSE46843)
- [data type]: [name of the resource] [accession number/identifier/doi] ([URL or identifiers.org/DATABASE:ACCESSION])

*** Note - All links should resolve to a page where the data can be accessed. ***

8) At EMBO Press we ask authors to provide source data for the main manuscript figures. You will receive a separate email with instructions for providing source data with your revised manuscript, including how to upload and organize the files.

Additional information on source data and instruction on how to label the files are available <https://www.embopress.org/page/journal/14693178/authorguide#sourcedata>

9) The journal requires a statement specifying whether or not authors have competing interests (defined as all potential or actual interests that could be perceived to influence the presentation or interpretation of an article). In case of competing interests, this must be specified in your disclosure statement. Further information: https://www.embopress.org/competing-interests

10) Figure legends and data quantification:
The following points must be specified in each figure legend:

- the name of the statistical test used to generate error bars and P values,
- the EXACT p-values,
- the number (n) of independent experiments (please specify technical or biological replicates) underlying each data point,
- the nature of the bars and error bars (s.d., s.e.m.)

- If the data are obtained from n {less than or equal to} 5, show the individual data points in addition to the SD or SEM.
- If the data are obtained from n {less than or equal to} 2, use scatter blots showing the individual data points.

Discussion of statistical methodology can be reported in the materials and methods section, but figure legends should contain a basic description of n, P and the test applied.

See also the guidelines for figure legend preparation:

https://www.embopress.org/page/journal/14693178/authorguide#figureformat

- Please also include scale bars in all microscopy images and define their size in the legend, not the image.

11) Our journal encourages inclusion of *data citations in the reference list* to directly cite datasets that were re-used and obtained from public databases. Data citations in the article text are distinct from normal bibliographical citations and should directly link to the database records from which the data can be accessed. In the main text, data citations are formatted as follows: "Data ref: Smith et al, 2001" or "Data ref: NCBI Sequence Read Archive PRJNA342805, 2017". In the Reference list, data citations must be labeled with "[DATASET]". A data reference must provide the database name, accession number/identifiers and a resolvable link to the landing page from which the data can be accessed at the end of the reference. Further instructions are available at <https://www.embopress.org/page/journal/14693178/authorguide#referencesformat>.

12) All Materials and Methods need to be described in the main text using our 'Structured Methods' format. According to this format, the Methods section includes a Reagents and Tools Table (listing key reagents, experimental models, software and relevant equipment and including their sources and relevant identifiers) followed by a Methods and Protocols section describing the methods, ideally using a step-by-step protocol format. The aim is to facilitate adoption of the methodologies across labs. Please download and fill our Reagents and Tools Table template (.docx), which you can find in our author guidelines: https://www.embopress.org/page/journal/14693178/authorguide#structuredmethods.
When submitting your revised manuscript, please do not include the Reagents and Tools Table in the Methods section of the manuscript but upload it as a separate file choosing the file type "Reagent Table".
An example of a Method paper with Structured Methods can be found here: https://www.embopress.org/doi/10.15252/msb.20178071.

13) As part of the EMBO publication's Transparent Editorial Process, EMBO Reports publishes online a Review Process File to accompany accepted manuscripts. This File will be published in conjunction with your paper and will include the referee reports, your point-by-point response and all pertinent correspondence relating to the manuscript.

You are able to opt out of this by letting the editorial office know (emboreports@embo.org). If you do opt out, the Review Process File link will point to the following statement: "No Review Process File is available with this article, as the authors have chosen not to make the review process public in this case."

We would also welcome the submission of cover suggestions, or motifs to be used by our Graphics Illustrator in designing a cover.

I look forward to seeing a revised form of your manuscript when it is ready and please let me know if you have questions or comments regarding the revision.

Yours sincerely,

Kurt Weir
Editor
EMBO Reports

Referee #1:

Chen and collaborators propose in this article a comprehensive study of hypoxia-driven metabolic reprogramming, highlighting on a novel insight that involves cholesterol accumulation, the autophagy-restrictive mTORC1 pathway and histone lactylation, with an important focus on the receptor SCARB1. The work sustained thoroughly explains and validates the hypotheses and supports the biological conclusions. The rationale behind the study is well explained and well sustained by the literature. The paper results in itself well written and the images explain clearly the objectives of the study. The data support a significant value in novel cancer therapy. Although, there are a few minor points to remark.

- Fig. 1A could benefice of another control diagram of cholesterol accumulation in Hep3B and PLC cells in condition of normoxia at 24 and 48 hours to compare each time points.
- Fig. 2A, ChIP assays have determined that the transcription factor HIF-1 alfa does not directly regulate the overexpression of SCARB1. However, being HIF-1alfa a key marker of hypoxia, it could be of additional value to monitor it throughout the study as a control.
- Authors enlighted that SCARB1 is linked with proliferation in Hep3B and PLC in fig. 3A, B and cell death fig 3C. An analysis of the involvement of SCARB1 in migration and invasion under hypoxia and normoxia (using Boyden Chamber assay, motility assays...for example) could further validate the relevance of SCARB1 in the process.
- The immunohistochemistry assays in fig. 5G lack of the observation of some markers. It should be informative to observe in the tissue (especially in HCC specimens) pSK6, LDHA for example and their change of staining.

Referee #2:

Summary & Significance
This manuscript identifies a hypoxia-adaptation pathway in hepatocellular carcinoma (HCC) wherein lactate-driven H3K18 lactylation (H3K18la) upregulates SCARB1, enhancing exogenous cholesterol uptake. The resulting cholesterol accumulation activates mTORC1, suppressing excessive autophagy and thereby sustaining tumor growth under hypoxic stress.
The authors propose a coherent mechanistic bridge connecting tumor metabolism (lactate), epigenetic regulation (H3K18la), and lipid-signaling cross-talk (SCARB1-mTORC1-autophagy), and they highlight SCARB1 as a plausible therapeutic target.
The work is conceptually appealing and broadly relevant to cancer metabolism and hypoxia biology. However, several mechanistic gaps, missing controls, and contextual issues limit the manuscript's current strength.

Major Comments
1. Scope of lactate as a TCA fuel (Introduction)
The statement that lactate is an "irreplaceable and primary fuel for the TCA cycle" (Hui et al., 2017) overstates the case. Lactate is indeed a major circulating carbon source, but its contribution is tissue-, context-, and diet-dependent. Please temper the language and align more closely with the cited literature.
2. Clarity and flow on histone lactylation phrasing
The sentence "Beyond this, the discovery of histone lysine lactylation (Kla) provides a link between gene regulation and cell metabolism through dysmetabolic activity and epigenetic modifications..." is difficult to follow. Rephrase for clarity, for example: "The discovery of histone lysine lactylation (Kla) established a direct link between cellular metabolic state and gene regulation, with lactate acting as an epigenetic signal that shapes the tumor microenvironment."
3. Placing the SCARB1→mTORC1→autophagy axis in the context of prior literature
The proposed model contrasts with reports showing that SCARB1 can enhance autophagy in macrophages (PMID: 33661763) and that lactylation stabilizes TFEB, thereby promoting autophagy (PMID: 39196068). Please reconcile these findings. For example, by discussing potential cancer-type or hypoxia-specific dependencies, differential cholesterol thresholds, or distinct transcriptional programs (e.g., TFEB, PPARα).
Where feasible, supporting experiments (TFEB and PPARα levels {plus minus} SCARB1 or lactate manipulation) would clarify whether your pathway suppresses autophagy by overriding TFEB activation.
4. Mechanistic specificity of lactate source and entry
The current evidence rests largely on lactate synthesis blockade (LDHA knockdown, oxamate). To solidify the causal chain, the authors should also block lactate import (e.g., MCT1, MCT2 and/or MCT4 inhibition). Please specify whether culture media contained exogenous lactate or used dialyzed serum (regular fetal bovine serum that is not dialyzed contains lactate). This clarification is essential to demonstrate that intracellularly generated lactate, rather than extracellular sources, drives H3K18la → SCARB1 induction.
5. Rescue experiments with extracellular lactate
It would strengthen the model to show that exogenous lactate supplementation rescues SCARB1 expression in LDHA-deficient or LDHA-inhibited cells. A positive rescue would confirm that the regulatory axis depends on lactate itself rather than upstream glycolytic flux.
6. Mechanistic validation of H3K18la-mediated transcriptional activation
Although ChIP-qPCR indicates H3K18la enrichment at the SCARB1 promoter, functional evidence for transcriptional activation is lacking. Reporter assays using SCARB1 promoter constructs {plus minus} p300 inhibition or mutation of the H3K18 site would directly demonstrate transcriptional causality. In addition, testing p300 knockdown or catalytically inactive mutants would confirm that this acetyltransferase mediates lactylation-dependent SCARB1 activation.
7. Autophagy readouts and mTORC1 depth
The conclusions about autophagy rely mainly on LC3-II accumulation. Measuring autophagic flux (e.g., with bafilomycin A1 or tandem mCherry-GFP-LC3 reporters) would clarify whether changes reflect altered initiation or clearance. Likewise, include additional mTORC1 readouts (p-4EBP1, ULK1 S757) and time-course analyses to substantiate the directionality (SCARB1 → mTORC1 → autophagy suppression).
8. RNA-seq replication and cholesterol flux
RNA-seq was performed with "one replicate per condition," which undermines statistical confidence. Biological replicates are essential. Furthermore, cholesterol changes are inferred from total content rather than direct uptake assays. Tracer or fluorescent cholesterol uptake measurements would strengthen the claim that SCARB1 mediates enhanced import rather than synthesis.
9. Clinical correlation and prognostic analysis
The IHC data linking H3K18la and SCARB1 in 46 paired samples are encouraging but largely correlative. Including multivariate survival analysis (stage, grade, age) or confirming the survival trend in the study's own cohort, rather than relying solely on GEPIA, would increase translational significance.

Figure-Specific & Presentation Comments
10. Fig. 1A-C & EV1A - Normalization:
Express cholesterol levels relative to the 0 h time point for clarity.

11. Figure labeling and navigation:
Add figure and page numbers and define all abbreviations (e.g., NTC = non-targeting control).
12. Fig. 1D - Controls:
Include a clear normoxia control and discuss the persistent VLDLR upregulation (EV1C): is this statistically or biologically significant? Quantify if relevant.
13. Fig. 3C - Cell-death pathways:
Provide p-values and indicate markers used for apoptosis (e.g., cleaved CASP3), necroptosis (MLKL phosphorylation), ferroptosis (GPX4 loss), etc.
14. Fig. 3H - Partial rescue:
Discuss whether residual cell death reflects non-autophagic pathways; targeted inhibitors could clarify this.
15. Fig. 4A - Quantification:
Add band-intensity quantification (densitometry) for p-S6K and LC3-II to support representative blots.
16. Integration with TFEB literature:
Incorporate a short paragraph in the Discussion reconciling your model (lactate → SCARB1 → mTORC1 → autophagy suppression) with studies showing lactate- or lactylation-mediated TFEB activation. Distinguish between early adaptive versus chronic hypoxic responses, or nucleus-cytoplasm regulation differences.

Overall Assessment
The manuscript provides an appealing conceptual advance linking lactate-driven epigenetic reprogramming to cholesterol-dependent signaling in hypoxic HCC. Addressing the points above-particularly mechanistic validation of H3K18la transcriptional control, flux and replication rigor, and contextual reconciliation with prior autophagy literature-will substantially enhance the clarity, rigor, and translational relevance of this work.
With these improvements, the study would merit strong consideration for publication in EMBO Reports.

Referee #3:

In this manuscript the authors showed data that under hypoxia the cholesterol transporter SCARB1 is elevated to increase cholesterol uptake. They suggest that lactate produced under hypoxia is responsible for the elevation of SCRAB1 expression. They showed that histone H3K18 is lactylated at the SCRAB1 promoter, which could explain the transcriptional elevation of SCRAB1. Further they showed that SCRAB1 depletion impairs the proliferation and tumorigenesis of HCC cells. The authors claim that SCRAB1 is required for the activation of mTORC1 and inhibition of autophagy under hypoxia. However, it is known that hypoxia inhibits mTORC1 through the expression of REDD1. In addition, the authors showed that induction of SCRAB1 by hypoxia is not sufficient to activate mTORC1 and inhibits autophagy questioning the physiological significance of SCRAB1 overexpression. The authors also did not show the mechanism by which SCRAB1 and cholesterol activate mTORC1. Overall, there are several conceptual and specific concerns listed below:
Conceptual concerns:
1. The authors suggest that autophagy induced by mTORC1 inhibition is causing cell death. However, autophagy is in fact promoting survival under nutrient stress. Indeed, inhibition of mTORC1 and activation of autophagy by rapamycin does not induce cell death but rather attenuates cell proliferation.

2. Although depletion of SCRAB1 under hypoxia inhibits mTORC1 and induces autophagy (Fig. 4A), the elevated expression of SCRAB1 under hypoxia is not sufficient to prevent inhibition of mTORC1 and activation of autophagy under hypoxia (Fig. 3F and Fig. 4C). Therefore, the effect of physiological levels of SCARB1 on mTORC1 and autophagy under hypoxia are questionable.
Specific points:
1. The authors need to conduct ECAR and show how much lactate is elevated under hypoxia.
2. The authors need to show level and localization of SCARB1 by IF.
3. Fig. 1F, G- It appears that the Induced level of SCARB1 protein is much higher than the elevation of Scrab1 mRNA (especially in Fig. 1G) suggesting that there is a posttranscriptional effect on the level of SCRAB1 protein.
4. Unlike the authors' assessment SCARB1 is required for cell proliferation both under hypoxia and normoxia (Fig. EV3A). It is not clear why overexpression of SCRAB1 increases proliferation only under hypoxia as under these conditions it is elevated anyway.
5. Fig. 3C suggests that the effect of SCARB1 depletion on proliferation is mostly due to cell death (about 30% cell death). The authors should determine cell death also under normoxic conditions.
6. To clarify the effect of SCARB1 on cell proliferation versus cell death the authors should measure BrdU incorporation or Ki67 staining and cell death markers.
7. It is known that hypoxia inhibits mTORC1 by inducing the expression of REDD1. How is SCARB1 related to that? Does it override the effect of REDD1?
8. Fig. 4A - The authors should conduct the experiment under normoxia. It is not clear from the WB if mTORC1 is inhibited under hypoxia.
9. Fig. 5F- The authors should have H3 and S6K on the immunoblot.
10. Fig. EV4B- Inconsistent with earlier results overexpression of SCRAB1 did not affect mTORC1 and autophagy in normal medium.
11. Fig. EV4C- If comparing pS6K/S6K, mTORC1 activity is much higher in regular medium than in DLPS + Cholesterol, yet autophagy is inhibited.

Point-by-point response to the comments

Editor's Decision:

Thank you for the submission of your research manuscript to our journal. We have now received the full set of referee reports that is copied below.

As you will see, the referees acknowledge that the findings are interesting and that the conclusions are overall supported by the data presented but they also raise a number of concerns and have suggestions how to further strengthen the data, particularly focused on situating the mechanism described in your paper within the relevant literature.

Given these constructive comments, we would like to invite you to revise your manuscript with the understanding that the referee concerns (as detailed above and in their reports) must be fully addressed and their suggestions taken on board. Please address all referee concerns in a complete point-by-point response. Acceptance of the manuscript will depend on a positive outcome of a second round of review. It is EMBO Reports policy to allow a single round of revision only and acceptance or rejection of the manuscript will therefore depend on the completeness of your responses included in the next, final version of the manuscript.

We realize that it is difficult to revise to a specific deadline. In the interest of protecting the conceptual advance provided by the work, we recommend a revision within 3 months (February 20th). Please discuss the revision progress ahead of this time with the editor if you require more time to complete the revisions.

I am also happy to discuss the revision further via e-mail or a video call, if you wish.

You can either publish the study as a short report or as a full article. For short reports, the revised manuscript should not exceed 27,000 characters (including spaces but excluding materials & methods and references) and 5 main plus 5 expanded view figures. The results and discussion sections must further be combined, which will help to shorten the manuscript text by eliminating some redundancy that is inevitable when discussing the same experiments twice. For a normal article there are no length limitations, but it should have more than 5 main figures and the results and discussion sections must be separate. In both cases, the entire materials and methods must be included in the main manuscript file.

Response: Based on the decision letter, we are grateful to the editor for the

encouragement and guidance. We also sincerely thank the referees for their constructive and insightful comments and suggestions, which have greatly helped us improve the manuscript. Over the past three months, we have performed a substantial number of additional experiments and have carefully addressed all concerns and comments raised by the editor and referees. We now resubmit an improved manuscript together with detailed, point-by-point responses.

For the referees' convenience, all revised figures are appended in this response file and labeled as F**igure R1** to **Figure R24**.

Reviewers' comments

Reviewer #1 (Remarks to the Author):

Referee #1:

Chen and collaborators propose in this article a comprehensive study of hypoxia-driven metabolic reprogramming, highlighting on a novel insight that involves cholesterol accumulation, the autophagy-restrictive mTORC1 pathway and histone lactylation, with an important focus on the receptor SCARB1. The work sustained thoroughly explains and validates the hypotheses and supports the biological conclusions. The rationale behind the study is well explained and well sustained by the literature. The paper results in itself well written and the images explain clearly the objectives of the study. The data support a significant value in novel cancer therapy. Although, there are a few minor points to remark.

Response: We appreciate the referee's positive comments that well summarized the major findings and significance of our study. We thank the referee for the constructive suggestions and have included additional data to strengthen our conclusions in the revised manuscript.

- Fig. 1A could benefice of another control diagram of cholesterol accumulation in Hep3B and PLC cells in condition of normoxia at 24 and 48 hours to compare each time points.

Response: We thank the referee for the suggestion. As suggested, we have measured intracellular cholesterol levels in Hep3B and PLC cells cultured under normoxia or

hypoxia for 24 and 48 hours. As shown in **Figure R1**, intracellular cholesterol levels were markedly increased under hypoxia compared with normoxia, with a further increase observed at 48 hours relative to 24 hours. These results support our conclusion that the observed cholesterol accumulation represents a hypoxia-associated response rather than a nonspecific effect of prolonged culture.

Based on the suggestion from the referee#2, the data are now normalized to the control group (Normoxia, 24h). We have replaced the original **Figure 1A** with this new data (**Figure R1**) in the revised manuscript.

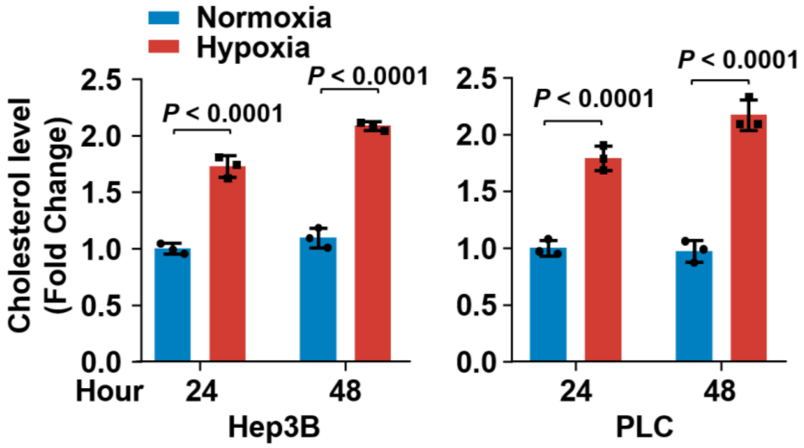

Figure R1. Intracellular total cholesterol levels in Hep3B and PLC cells cultured under normoxia or hypoxia for 24 h or 48 h. Please also see **Figure 1A** in the revised manuscript.

- Fig. 2A, ChIP assays have determined that the transcription factor HIF-1 alfa does not directly regulate the overexpression of SCARB1. However, being HIF-1alfa a key marker of hypoxia, it could be of additional value to monitor it throughout the study as a control.

Response: We appreciate the referee's suggestion. Accordingly, we have performed additional assays to monitor HIF-1α protein levels under hypoxic conditions, and the results showed that HIF-1α protein levels peaked rapidly at 6 h following hypoxia and subsequently declined, while remaining significantly higher than those under normoxia. Given the established role of HIF-1α in driving glycolytic reprogramming and lactate production, we further investigated an indirect regulatory mechanism. Notably, while HIF-1α knockdown reduced SCARB1 expression under hypoxia, supplementation with sodium lactate restored SCARB1 levels (**Figure R2**), indicating that hypoxia-induced SCARB1 expression is mediated through a HIF-1α–lactate axis

rather than by direct transcriptional activation by HIF-1α itself. We have included **Figure R2B** as **Figure 2B** in the revised manuscript.

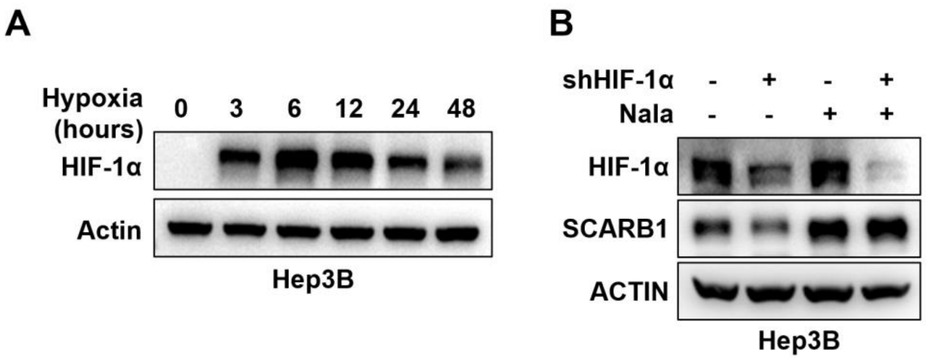

Figure R2. (A) Hep3B cells were cultured under hypoxia for 3, 6, 12, 24, and 48 hours. HIF1-α protein levels were detected by Western blotting. **(B)** Hep3B cells expressing shNTC or shHIF1-α were treated with or without 20 mM sodium lactate. SCARB1 protein levels were detected by Western blotting. Please also see **Figure 2B** in the revised manuscript.

- Authors enlighted that SCARB1 is linked with proliferation in Hep3B and PLC in fig. 3A, B and cell death fig 3C. An analysis of the involvement of SCARB1 in migration and invasion under hypoxia and normoxia (using Boyden Chamber assay, motility assays...for example) could further validate the relevance of SCARB1 in the process.

Response: We thank the referee for the suggestion. As recommended, we performed scratch and transwell assays to assess the effects of SCARB1 knockdown on the migratory and invasive capacities of Hep3B cells under both normoxic and hypoxic conditions (**Figure R3**). Consistent with the reported role of SCARB1 in promoting lung cancer metastasis (Liu et al, 2025), SCARB1 knockdown impaired both cell migration and invasion in Hep3B cells. However, we noted that SCARB1 knockdown triggered pronounced cell death (**Figure 3A-C**), underscoring its strong impact on cell viability. Therefore, our subsequent investigation was directed toward elucidating the mechanism underlying this SCARB1-dependent survival axis.

We acknowledge that SCARB1 may play a role in regulating hepatocellular carcinoma metastasis, as shown by our data; this represents a possibility that future work may explore.

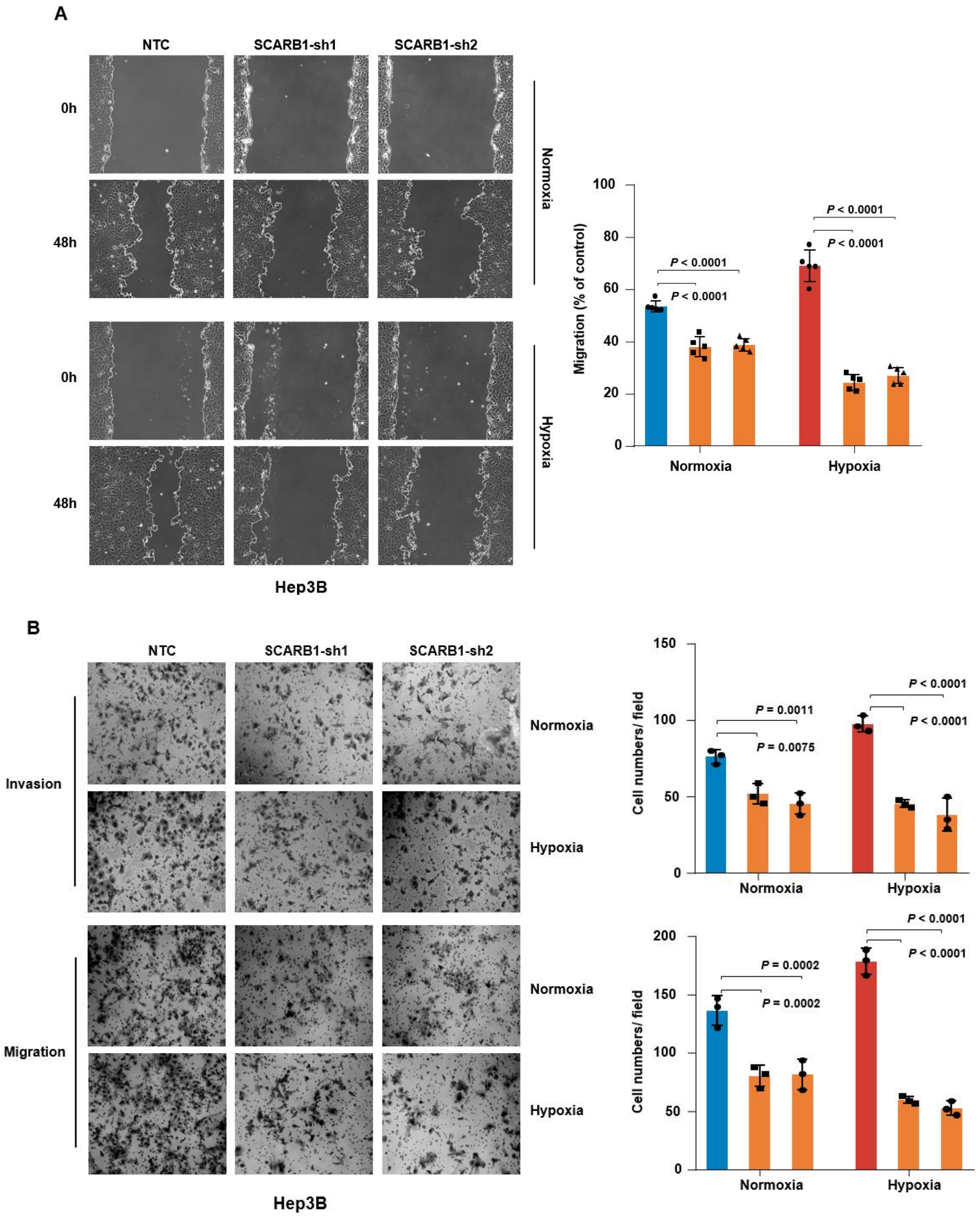

Figure R3. (A) The impacts of SCARB1 knockdown on cell migration under normoxia and hypoxia were assessed by scratch assay. **(B)** The impacts of SCARB1 knockdown on cell migration and invasion under normoxia and hypoxia were assessed by transwell assays.

- The immunohistochemistry assays in fig. 5G lack of the observation of some markers. It should be informative to observe in the tissue (especially in HCC specimens) pSK6, LDHA for example and their change of staining.

Response: We thank the referee for this helpful suggestion. As recommended, we expanded the immunohistochemistry analysis in HCC specimens to include additional markers relevant to the proposed pathway. Specifically, we have now examined the staining of pS6K and LDHA in tumor tissues (**Figure R4**). Quantitative analysis showed coordinated expression of LDHA, H3K18la, SCARB1, and p-S6K in clinicopathologically characterized liver cancer samples. We have replaced the original **Figure 5G** with the new data (**Figure R4A and the last panel of R4B**), and added the first and third panels of **Figure R4B** as **Figure EV5D** in the revised manuscript.

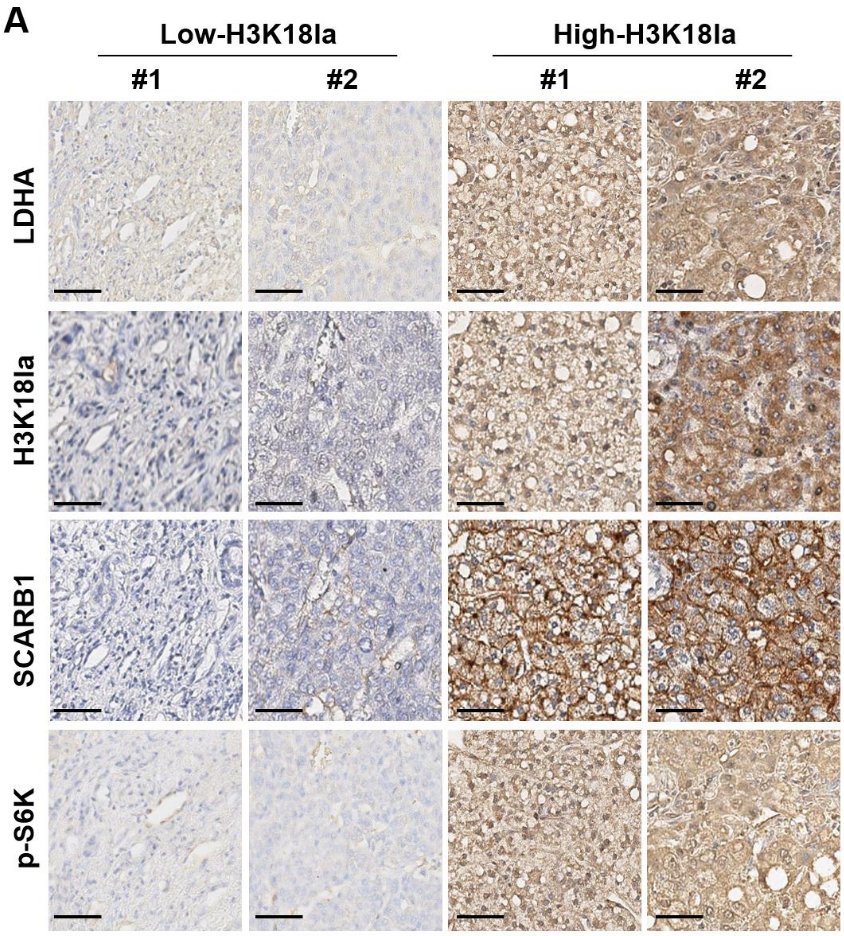

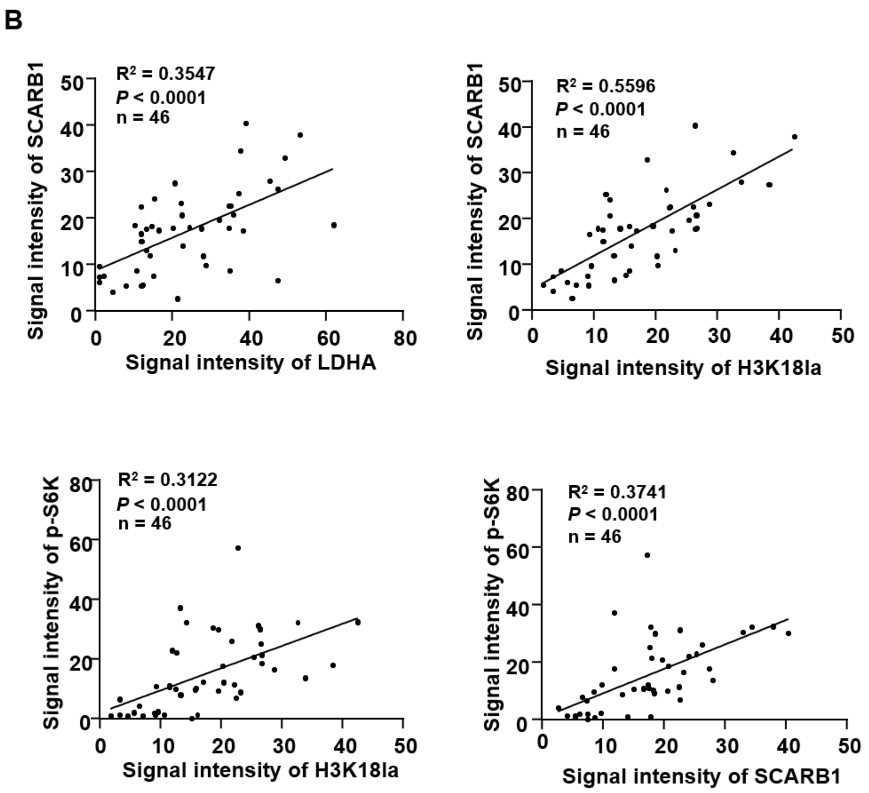

Figure R4. (A) Representative immunohistochemistry images of LDHA, H3K18la, SCARB1, and p-S6K staining in HCC specimens; Scale bars, 30 μm. **(B)** Analysis of expression of LDHA, H3K18la, SCARB1, and p-S6K in HCC specimens. Pearson correlation analyses were performed. Please also see **Figure 5G-H** and **Figure EV5D** in the revised manuscript.

Referee #2:

Summary & Significance

This manuscript identifies a hypoxia-adaptation pathway in hepatocellular carcinoma (HCC) wherein lactate-driven H3K18 lactylation (H3K18la) upregulates SCARB1, enhancing exogenous cholesterol uptake. The resulting cholesterol accumulation activates mTORC1, suppressing excessive autophagy and thereby sustaining tumor growth under hypoxic stress.

The authors propose a coherent mechanistic bridge connecting tumor metabolism (lactate), epigenetic regulation (H3K18la), and lipid-signaling cross-talk (SCARB1-mTORC1-autophagy), and they highlight SCARB1 as a plausible therapeutic target.

The work is conceptually appealing and broadly relevant to cancer metabolism and hypoxia biology. However, several mechanistic gaps, missing controls, and contextual issues limit the

manuscript's current strength.

Response: We appreciate the referee's positive comments on the significance of our study. We agree that strengthening the mechanistic support and experimental rigor is important, and accordingly, we have performed multiple additional experiments and incorporated new data in the revised manuscript to address these points. Detailed responses to each specific comment are provided below.

Major Comments

1. Scope of lactate as a TCA fuel (Introduction)

The statement that lactate is an "irreplaceable and primary fuel for the TCA cycle" (Hui et al., 2017) overstates the case. Lactate is indeed a major circulating carbon source, but its contribution is tissue-, context-, and diet-dependent. Please temper the language and align more closely with the cited literature.

Response: We thank our referee for pointing this out. We agree that the description of lactate as an "irreplaceable and primary fuel" in the original manuscript overstates the case. In the revised manuscript, we have replaced the sentence "lactate is now recognized as an irreplaceable and primary fuel for the TCA cycle" with "lactate can serve as a substrate for aerobic cancer cells" (Faubert et al, 2017; Guillaumond et al, 2013; Hensley et al, 2016; Kennedy et al, 2013).

2. Clarity and flow on histone lactylation phrasing

The sentence "Beyond this, the discovery of histone lysine lactylation (Kla) provides a link between gene regulation and cell metabolism through dysmetabolic activity and epigenetic modifications..." is difficult to follow. Rephrase for clarity, for example:

"The discovery of histone lysine lactylation (Kla) established a direct link between cellular metabolic state and gene regulation, with lactate acting as an epigenetic signal that shapes the tumor microenvironment."

Response: We sincerely thank our referee for this constructive suggestion on improving the clarity of our writing. The suggested rephrasing is excellent and much

clearer, and we have adopted it to replace the original sentence in the manuscript.

3. Placing the SCARB1→mTORC1→autophagy axis in the context of prior literature

The proposed model contrasts with reports showing that SCARB1 can enhance autophagy in macrophages (PMID: 33661763) and that lactylation stabilizes TFEB, thereby promoting autophagy (PMID: 39196068). Please reconcile these findings. For example, by discussing potential cancer-type or hypoxia-specific dependencies, differential cholesterol thresholds, or distinct transcriptional programs (e.g., TFEB, PPARα).

Where feasible, supporting experiments (TFEB and PPARα levels {plus minus} SCARB1 or lactate manipulation) would clarify whether your pathway suppresses autophagy by overriding TFEB activation.

Response: We thank the referee for raising this important point. As discussed in the original manuscript, Tao et al. reported that in macrophages, SCARB1 localized to lysosomes promotes autophagy through a PPARα–TFEB–dependent transcriptional program to facilitate cholesterol clearance during atherosclerosis. In contrast, our study indicates that the primary function of SCARB1 in hepatocellular carcinoma cells is to mediate the uptake of cholesterol from extracellular HDL to meet tumor metabolic demands. Consistently, as shown in **Figure R17**, SCARB1 is localized at the plasma membrane. This distinction likely reflects fundamental metabolic differences between immune cells and cancer cells.

In addition, according to Huang et al., lactate promotes TFEB activation and enhances autophagy in pancreatic cancer; however, sodium oxalate treatment in hepatocellular carcinoma cells promoted autophagy rather than suppressing it, as shown in **Figure 4F** in the original manuscript. This apparent discrepancy may reflect differences in tumor type or context-dependent metabolic adaptations under stress conditions. Following the referee's suggestion, we examined the protein levels of TFEB and PPARα upon SCARB1 knockdown or overexpression in hepatocellular carcinoma cells. Notably, neither manipulation altered the expression levels of PPARα or TFEB (**Figure R5**), indicating that in our system, SCARB1 does not regulate autophagy through the canonical PPARα–TFEB transcriptional axis. Instead, it acts

through a distinct, mTORC1-dependent pathway driven by cholesterol-mediated signaling.

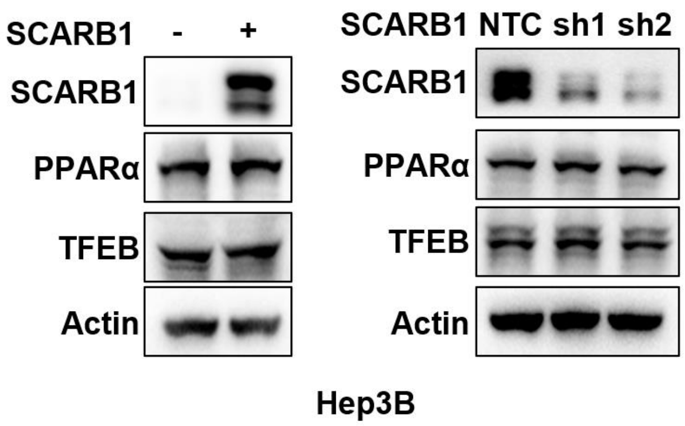

Figure R5. Western blot analysis of PPARα and TFEB protein expression in Hep3B cells with SCARB1 overexpression or knockdown.

4. Mechanistic specificity of lactate source and entry

The current evidence rests largely on lactate synthesis blockade (LDHA knockdown, oxamate). To solidify the causal chain, the authors should also block lactate import (e.g., MCT1, MCT2 and/or MCT4 inhibition). Please specify whether culture media contained exogenous lactate or used dialyzed serum (regular fetal bovine serum that is not dialyzed contains lactate). This clarification is essential to demonstrate that intracellularly generated lactate, rather than extracellular sources, drives H3K18la → SCARB1 induction.

Response: We thank the referee for the very insightful comments. First, we clarify that all cells in this study were cultured in DMEM supplemented with regular (non-dialyzed) fetal bovine serum. We acknowledge that non-dialyzed FBS contains basal levels of lactate and therefore represents a potential extracellular source.

To directly address the referee's suggestion, we examined the role of monocarboxylate transporters. Since MCT2 expression is relatively low in many cancer types, including hepatocellular carcinoma (HCC), while MCT1 is the predominant lactate importer and MCT4 is a hypoxia-inducible lactate exporter, we focused on MCT1 and MCT4. We found that MCT1 knockdown did not significantly affect SCARB1 expression, which is likely because HCC cells, being highly glycolytic, produce most of their intracellular lactate endogenously, and the

contribution from extracellular lactate is likely minimal. In contrast, knockdown of MCT4, which limits lactate efflux and thereby promotes intracellular lactate accumulation, resulted in a marked upregulation of SCARB1 expression. Consistently, overexpression of MCT4 significantly suppressed SCARB1 expression (**Figure R6**).

Together, our data provide strong evidence that the H3K18la–SCARB1 axis is predominantly driven by lactate generated through intracellular glycolysis under hypoxic conditions.

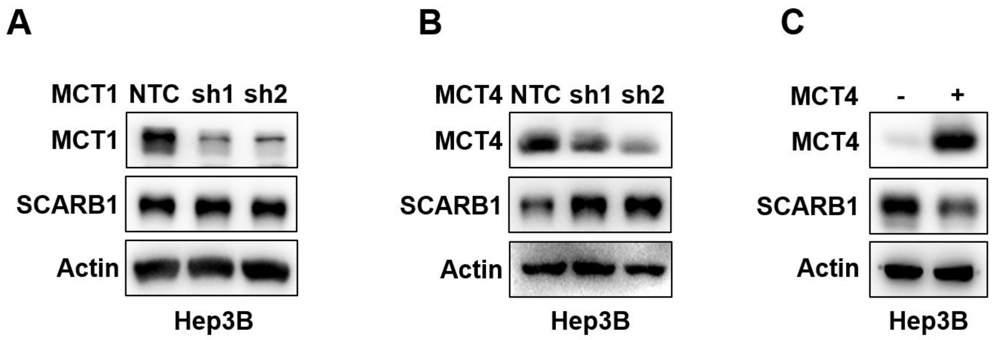

Figure R6. (A) Western blot analysis of SCARB1 protein expression in Hep3B cells with MCT1 knockdown. **(B, C)** Western blot analysis of SCARB1 protein expression in Hep3B cells with MCT4 knockdown or overexpression. Please also see **Figure EV1E-G** in the revised manuscript.

5. Rescue experiments with extracellular lactate

It would strengthen the model to show that exogenous lactate supplementation rescues SCARB1 expression in LDHA-deficient or LDHA-inhibited cells. A positive rescue would confirm that the regulatory axis depends on lactate itself rather than upstream glycolytic flux.

Response: We thank the referee for this important suggestion, which allows us to further distinguish the role of lactate itself from upstream glycolytic flux in regulating SCARB1 expression.

To directly address this point, we supplemented LDHA- knockdown hepatocellular carcinoma cells with sodium lactate. Notably, this supplementation restored SCARB1 expression in LDHA-knockdown cells (**Figure R7**). This result demonstrates that lactate itself is sufficient to drive SCARB1 expression, confirming its central role in the H3K18la–SCARB1 regulatory axis. We have included **Figure R7** as **Figure 1H** in the revised manuscript.

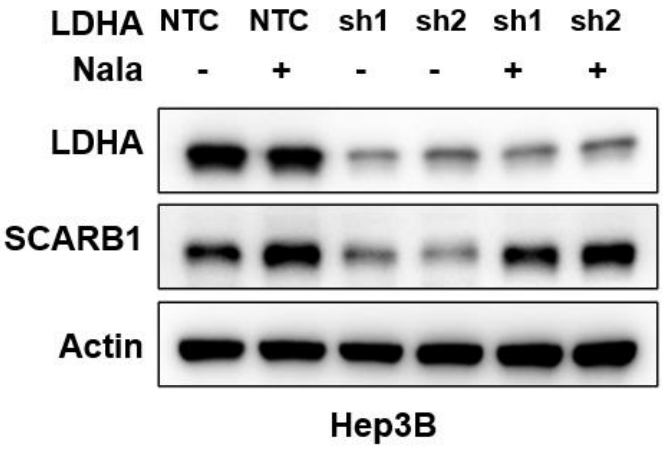

Figure R7. Hep3B cells with LDHA knockdown were treated with or without 20 mM sodium lactate. SCARB1 protein levels were detected by Western blotting. Please also see **Figure 1H** in the revised manuscript.

6. Mechanistic validation of H3K18la-mediated transcriptional activation

Although ChIP-qPCR indicates H3K18la enrichment at the SCARB1 promoter, functional evidence for transcriptional activation is lacking. Reporter assays using SCARB1 promoter constructs {plus minus} p300 inhibition or mutation of the H3K18 site would directly demonstrate transcriptional causality. In addition, testing p300 knockdown or catalytically inactive mutants would confirm that this acetyltransferase mediates lactylation-dependent SCARB1 activation.

Response: We appreciate the referee's suggestion. We agree that luciferase reporter assays are powerful tools for assessing promoter activity. However, such standard reporter assays are not suitable for investigating regulation by histone modifications like lactylation, as the transfected plasmid lacks chromatinized histone substrates necessary for this epigenetic mark.

To directly address the referee's point, we focused on the role of p300, a known histone acetyltransferase and lactyltransferase, in regulating SCARB1 expression. Notably, knockdown of p300 markedly suppressed SCARB1 expression, whereas overexpression of the p300 HAT catalytic domain significantly enhanced SCARB1 expression (**Figure R8**). Together with the enrichment of H3K18la at the SCARB1 promoter revealed by ChIP-qPCR, these results provide strong functional evidence that p300-mediated histone lactylation contributes to the transcriptional activation of

SCARB1. We have included these data as **Figure 2J and K** in the revised manuscript, and the original **Figure. 2I and J** have been moved to **Figure. EV2D and 2E**.

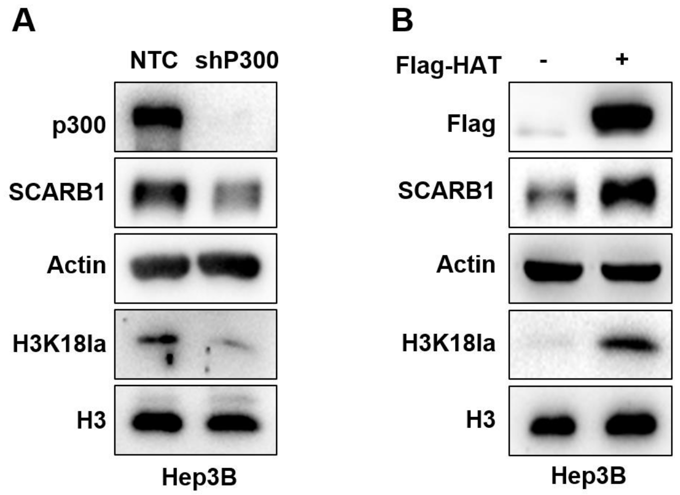

Figure R8. (A) Western blot analysis of SCARB1 protein expression in Hep3B cells with P300 knockdown. **(B)** Western blot analysis of SCARB1 protein expression in Hep3B cells with P300-HAT overexpression. Please also see **Figure 2J and K** in the revised manuscript.

7. Autophagy readouts and mTORC1 depth

The conclusions about autophagy rely mainly on LC3-II accumulation. Measuring autophagic flux (e.g., with bafilomycin A1 or tandem mCherry-GFP-LC3 reporters) would clarify whether changes reflect altered initiation or clearance. Likewise, include additional mTORC1 readouts (p-4EBP1, ULK1 S757) and time-course analyses to substantiate the directionality (SCARB1 → mTORC1 → autophagy suppression).

Response: We thank the referee for the valuable suggestion. As recommended, we treated cells with bafilomycin A1 to evaluate autophagic flux, and we found that LC3-II accumulation was further enhanced in SCARB1-knockdown cells, indicating increased autophagic flux rather than impaired autophagosome clearance. Consistently, mCherry-GFP-LC3 reporter assays showed a significant increase in red-only puncta (autolysosomes) in SCARB1-depleted cells, whereas yellow puncta (autophagosomes) did not accumulate. Together, these results demonstrate that SCARB1 knockdown promotes autophagic flux (**Figure R9**). We have now replaced the original **Figure 3D-E** with these new data (**Figure R9A-B**) in the revised manuscript.

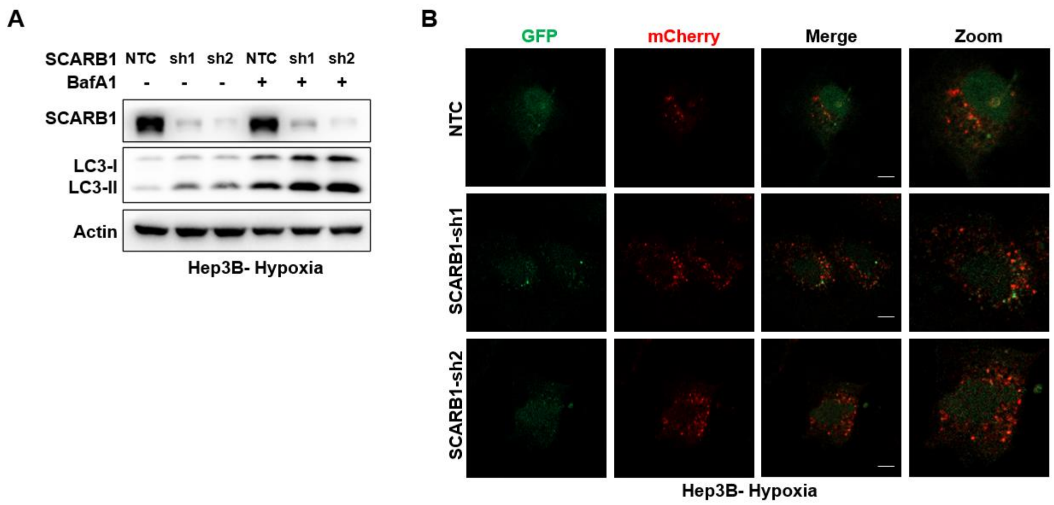

Figure R9. (A) Autophagic flux analysis using bafilomycin A1 (BafA1). Cells with SCARB1 knockdown were treated with BafA1 (100 nM, 12h). LC3-II levels were detected by Western blotting. **(B)** Ad-GFP-mCherry-LC3B was expressed in Hep3B cells with SCARB1 knockdown. The number of LC3 puncta was analyzed using a fluorescence microscope. Scale bar: 10 μm. Please also see **Figure 3D-E** in the revised manuscript.

In addition, following the referee's suggestion, we examined additional mTORC1 readouts, including the phosphorylation of 4EBP1 and ULK1. In response to comments from the referee #3, we included an additional normoxic NTC control in this experiment. And we have now performed densitometric quantification of p-ULK1, p-S6K, p-4EBP1, and LC3-II from these immunoblots, according to your comment No.15. (The p-ULK1, p-S6K, and p-4EBP1 signals were normalized to total ULK1, S6K, and 4EBP1, respectively; LC3-II was normalized to Actin). Changes in these markers were consistent with the alterations observed for p-S6K, further supporting the conclusion that SCARB1 regulates mTORC1 activity (**Figure R10**). We have now replaced the original **Figure 4A and 4C** with these new data (**Figure R10A-B**) in the revised manuscript.

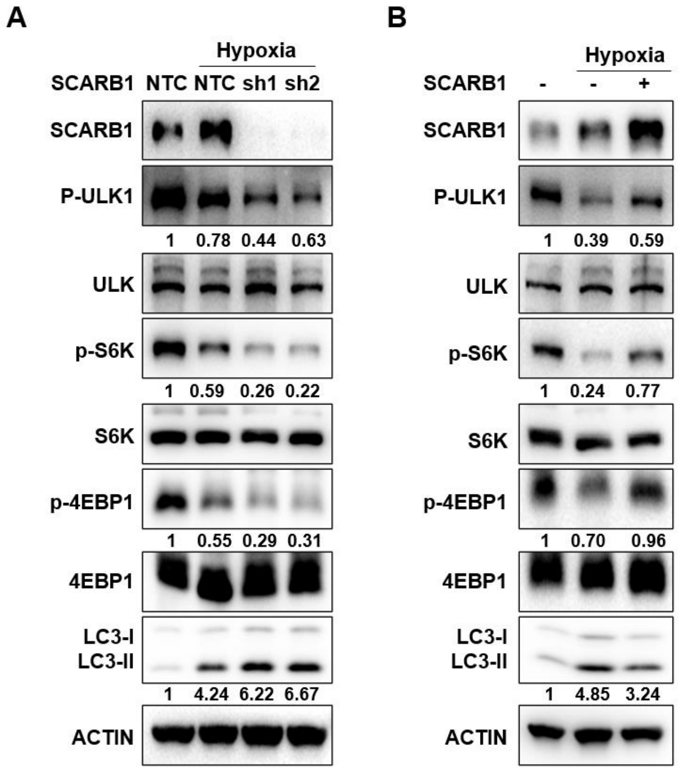

Figure R10. (A, B) Western blot analysis of p-ULK1, ULK1, p-S6K, S6K, p-4EBP1, 4EBP1, and LC3I/II protein expression in Hep3B cells with SCARB1 knockdown or overexpression. Please also see **Figure 4A-B** in the revised manuscript.

In our study, we have shown that SCARB1 knockdown consistently led to reduced mTORC1 activity accompanied by enhanced autophagy, whereas SCARB1 overexpression resulted in mTORC1 activation and suppression of autophagy. These bidirectional effects provide strong evidence that SCARB1 functions upstream of mTORC1 to restrain autophagy. While additional time-course analyses could further refine pathway kinetics, the concordant results from multiple mTORC1 readouts support the directionality of the SCARB1 → mTORC1 → autophagy suppression axis.

8. RNA-seq replication and cholesterol flux

RNA-seq was performed with "one replicate per condition," which undermines statistical confidence. Biological replicates are essential. Furthermore, cholesterol changes are inferred from total content rather than direct uptake assays. Tracer or fluorescent cholesterol uptake measurements would strengthen the claim that SCARB1 mediates enhanced import rather than

synthesis.

Response: We thank the referee for highlighting the importance of biological replication and direct assessment of cholesterol flux. To address concerns regarding RNA-seq rigor, we repeated the experiments with three independent biological replicates per condition (**Figure R11**), and the datasets have been deposited in the GEO database (GSE316557). We have replaced the original **Figure 1D** with **Figure R11A** and the original **Figure EV1B** with **Figure R11B** in the revised manuscript.

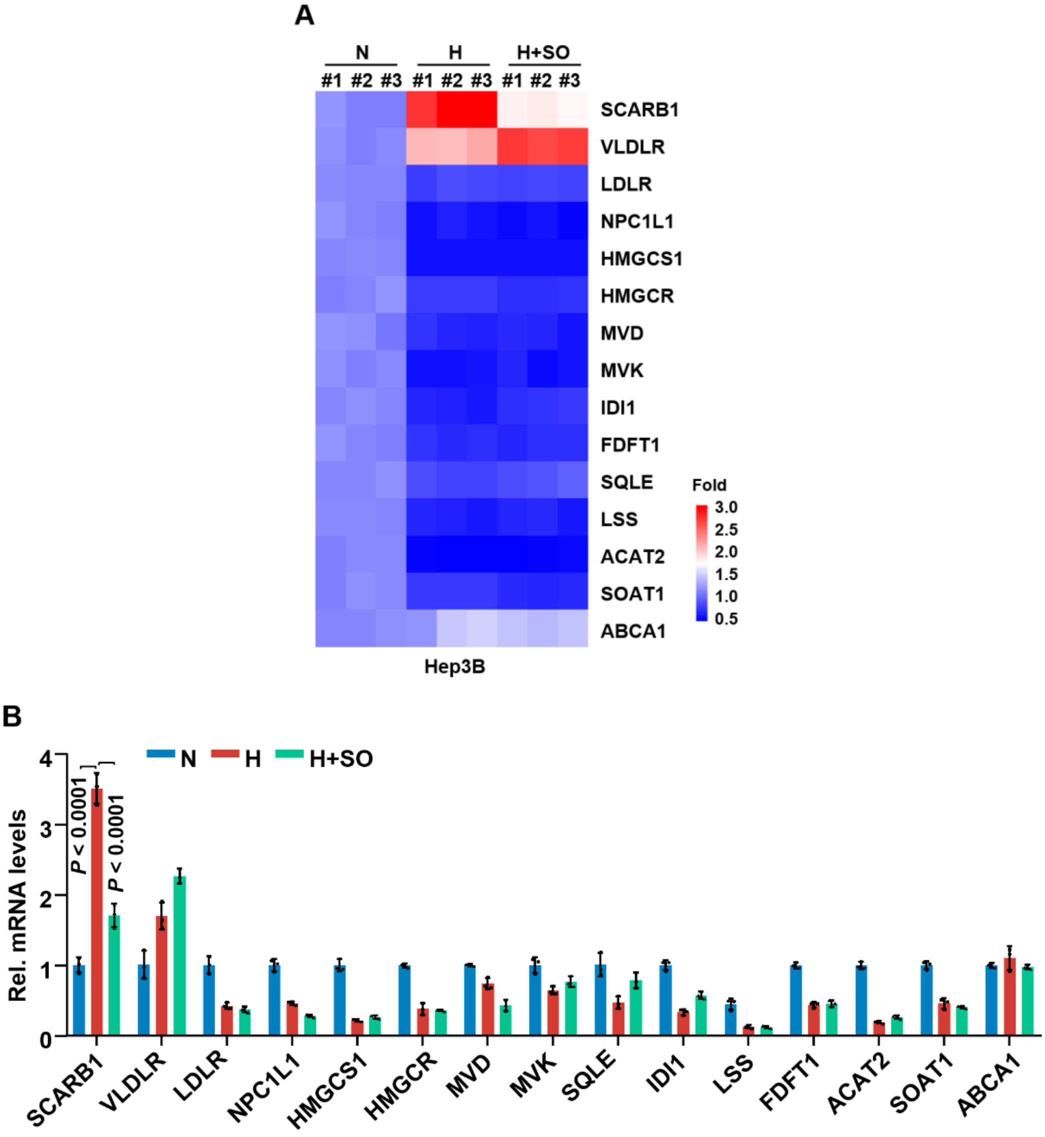

Figure R11. (A) Heatmap of cholesterol metabolism-related gene expression in Hep3B cells cultured under normoxia, hypoxia, or hypoxia with 20 mM sodium oxamate for 48 h. **(B)** RT–qPCR analysis was performed using the same RNA samples as those used for the heatmap shown in **(A).**

In addition, following the referee's suggestion, we performed DiI-HDL uptake

assays to directly measure cholesterol import. Consistent with our previous findings, DiI fluorescence intensity was significantly increased under hypoxia and was markedly suppressed by sodium oxamate treatment. Notably, SCARB1 overexpression enhanced HDL uptake, whereas SCARB1 knockdown effectively abrogated the hypoxia-induced increase (**Figure R12**). These results provide direct functional evidence that SCARB1 mediates enhanced cholesterol import under hypoxia, thereby strengthening the conclusions drawn from total cholesterol measurements. We have included **Figure R12A** as **Figure EV1C** in the revised manuscript.

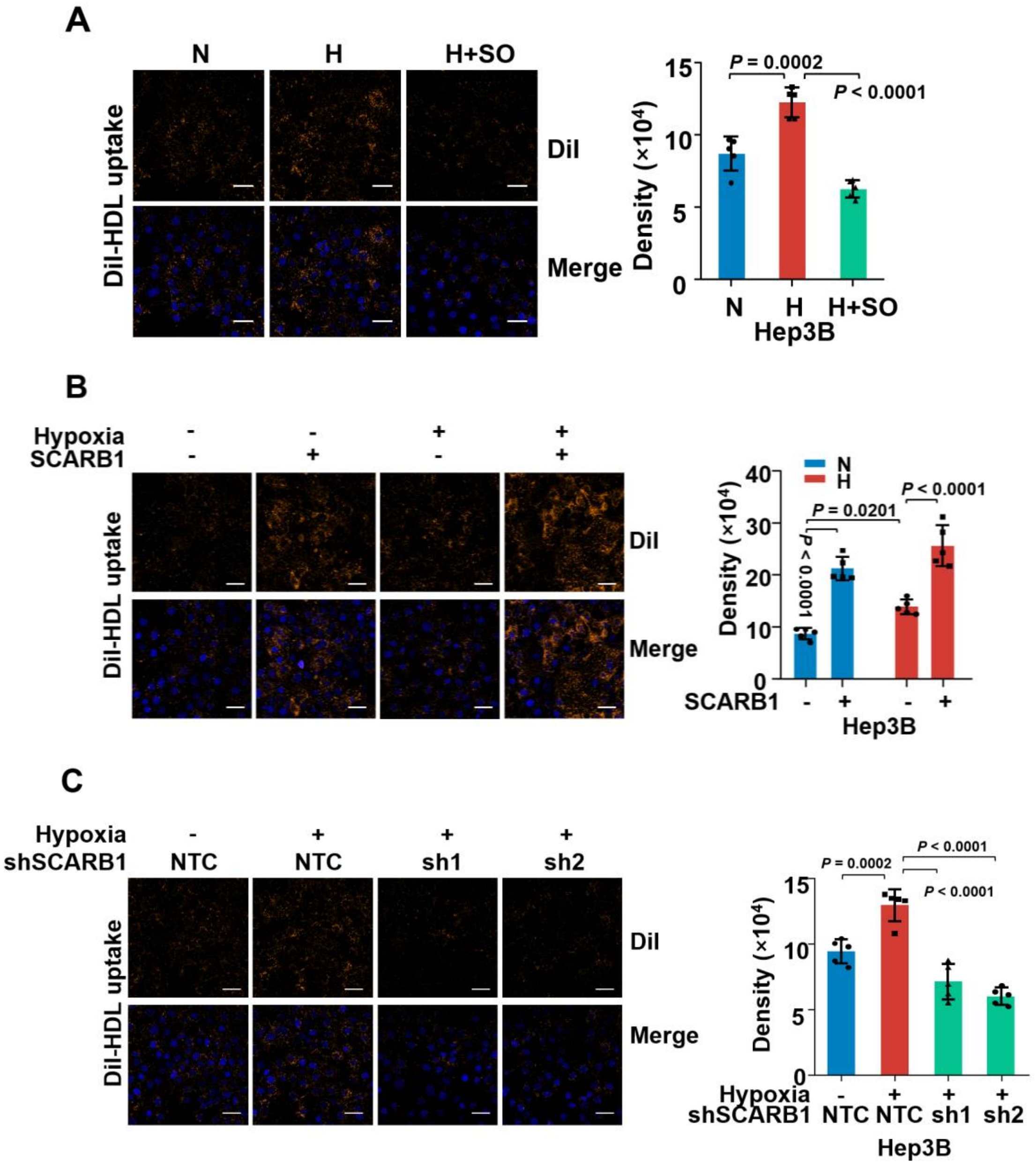

Figure R12. (A) Hep3B cells were cultured under normoxia (N), hypoxia (H), or hypoxia with 20 mM sodium oxamate (H+SO) for 48 h, followed by measurement of DiI–HDL uptake. **(B, C)** Hep3B and PLC cells with SCARB1 overexpression (B) or knockdown (C) were cultured under normoxia or hypoxia for 48 h, followed by measurement of DiI–HDL uptake. Scale bar: 50 μm. Please also see **Figure EV1C** in the revised manuscript.

9. Clinical correlation and prognostic analysis

The IHC data linking H3K18la and SCARB1 in 46 paired samples are encouraging but largely correlative. Including multivariate survival analysis (stage, grade, age) or confirming the survival trend in the study's own cohort, rather than relying solely on GEPIA, would increase translational significance.

Response: We sincerely thank the referee for this important and insightful suggestion. We fully agree that the inclusion of multivariate survival analyses or validation in an independent clinical cohort would further strengthen the independent prognostic significance of our findings.

However, within the current retrospective cohort, key clinicopathological covariates required for reliable multivariate analysis (such as complete staging and grading information) are partially missing, which precludes robust statistical modeling. In addition, the majority of the 46 paired HCC specimens were collected after 2020, and the current follow-up period is relatively short, resulting in a very low number of endpoint events (death). Under these circumstances, performing survival analyses would suffer from insufficient statistical power and may lead to unreliable or biased conclusions. We therefore believe that deferring such analyses until longer follow-up data are available is a more rigorous and cautious approach. Future work will include continued follow-up of this cohort to enable definitive survival validation.

Importantly, we provide additional evidence that supports the clinical relevance of this pathway in human HCC. As shown in Figure 5G, we extended our IHC analysis to include LDHA and pS6K, further supporting activation of the lactate–SCARB1–mTORC1 axis at the tissue level. Moreover, in Figure 5F, we demonstrate that H3K18la, SCARB1, and pS6K are all concomitantly upregulated in human HCC

samples, reinforcing the coordinated activation of this pathway in clinical specimens.

Figure-Specific & Presentation Comments

10. Fig. 1A-C & EV1A - Normalization:

Express cholesterol levels relative to the 0 h time point for clarity.

Response: We thank the referee for this helpful suggestion. As recommended, cholesterol levels in **Figure 1A–C** and **EV1A** are now expressed relative to the 0 h time point or normalized to the control group (Normoxia) **(Figure R13)**. The figures have been updated accordingly in the revised manuscript.

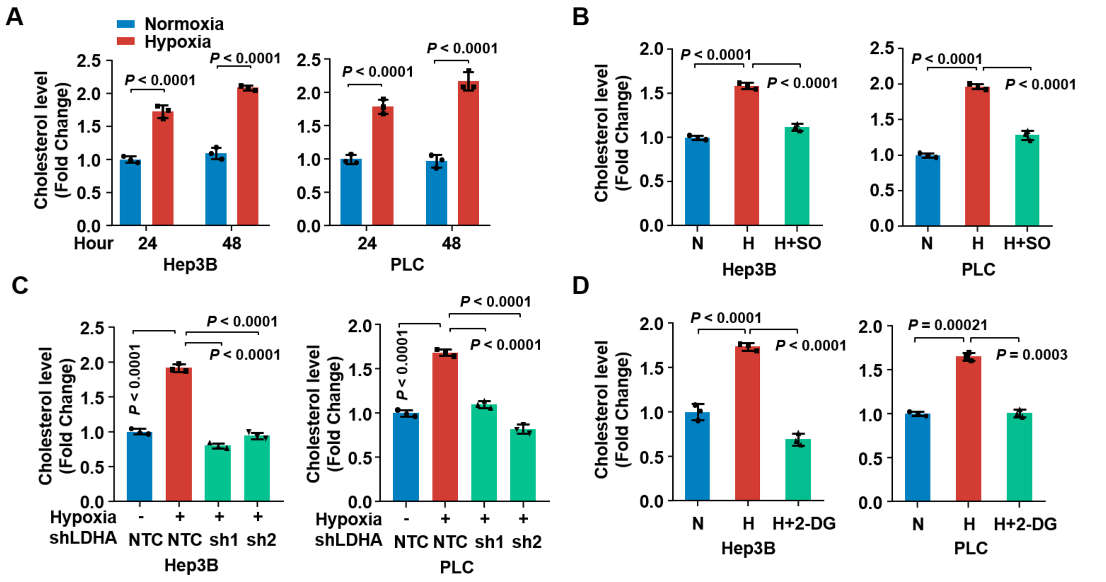

Figure R13. (A) Intracellular total cholesterol levels in Hep3B and PLC cells cultured under normoxia or hypoxia for 24 or 48 h. **(B)** Hep3B and PLC cells were cultured under normoxia (N), hypoxia (H), or hypoxia with 20 mM sodium oxamate (H+SO) for 48 h. The cellular cholesterol content was measured. **(C)** Hep3B and PLC cells expressing shNTC or shLDHA were cultured under normoxia or hypoxia for 48 h. The cellular cholesterol content was measured. **(D)** Hep3B and PLC cells were cultured under normoxia, hypoxia, or hypoxia with 10 mM 2-DG for 48 h. Cellular cholesterol content was measured. Please also see **Figure 1A, 1B, 1C, and EV1A** in the revised manuscript.

11. Figure labeling and navigation:

Add figure and page numbers and define all abbreviations (e.g., NTC = non-targeting control).

Response: Thank you for the suggestion. We have added figure and page numbers throughout the revised manuscript, and all abbreviations are now defined in the figure

legends.

12. Fig. 1D - Controls:

Include a clear normoxia control and discuss the persistent VLDLR upregulation (EV1C): is this statistically or biologically significant? Quantify if relevant.

Response: Thank you for the comment. Following this suggestion, we have included a clear normoxia control and replaced the original **Figure 1D** with updated RNA-seq data (**Figure R11A**).

We did observe that VLDLR expression was modestly upregulated under hypoxia (**Figure R11A and B**). However, inhibition of lactate production did not suppress VLDLR expression, suggesting that its regulation is not linked to the lactate-dependent pathway characterized in this study. In addition, RNA-seq read counts indicated that VLDLR expression levels were relatively low compared with SCARB1.

Given the limited magnitude of induction and lack of lactate responsiveness, we did not pursue further functional analyses of VLDLR. Instead, we focused on SCARB1, as its hypoxia- and lactate-dependent expression pattern was consistent with the observed changes in intracellular cholesterol levels.

13. Fig. 3C - Cell-death pathways:

Provide p-values and indicate markers used for apoptosis (e.g., cleaved CASP3), necroptosis (MLKL phosphorylation), ferroptosis (GPX4 loss), etc.

Response: Thank you for the suggestion. Following the comment from referee #3, we reconducted the experiment with both normoxia and hypoxia and replaced the original **Figure 3C** with the updated results. We have now added the corresponding p-values as suggested. In addition, immunoblot analyses were performed to examine established markers of apoptosis (Cleaved Caspase3), necroptosis (phosphorylated MLKL), and ferroptosis (GPX4). Our results showed that SCARB1 knockdown did not alter markers of necroptosis or ferroptosis, whereas both autophagy and apoptosis

markers were elevated. Pharmacological inhibition of either pathway significantly rescued cell viability, with both interventions showing statistically significant effects (**Figure R14**).

We cannot exclude a role of SCARB1 in regulating apoptosis, consistent with reports showing that SCARB1 depletion promotes apoptosis in renal cell carcinoma (Riscal et al, 2021). However, in our study, we focused on autophagy in the subsequent functional experiments, mainly because under hypoxia, inhibition of autophagy led to a more substantial attenuation of cell death than inhibition of apoptosis. In line with this observation, Western blot analyses revealed more evident changes in LC3 compared with cleaved CASP3. We have included **Figure R14** as **Figure EV3C** in the revised manuscript.

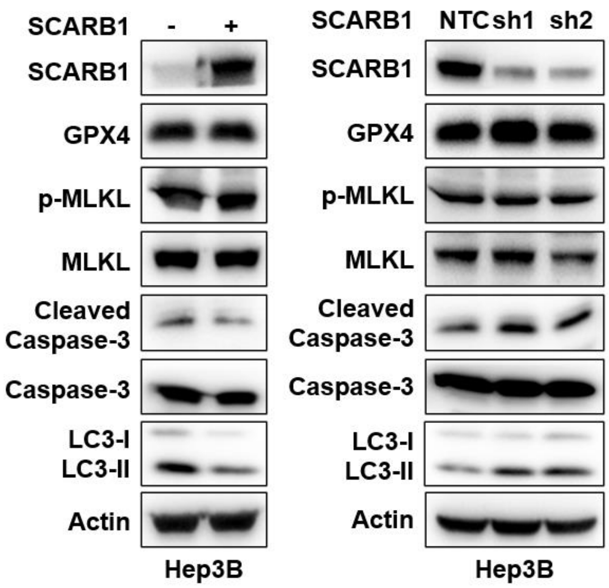

Figure R14. Western blot analysis of GPX4, phosphorylated MLKL, MLKL, cleaved caspase-3, Caspase3, and LC3-I/II in Hep3B cells with SCARB1 overexpression or knockdown under hypoxia. Please also see **Figure EV3C** in the revised manuscript.

14. Fig. 3H - Partial rescue:

Discuss whether residual cell death reflects non-autophagic pathways; targeted inhibitors could clarify this.

Response: We thank the referee for the suggestion. To address this point, we

evaluated the effects of combining an autophagy inhibitor with inhibitors of apoptosis, necroptosis, and ferroptosis on SCARB1 knockdown–induced cell death using flow cytometry (**Figure R15)**. The results showed that the addition of an apoptosis inhibitor further rescued cell death beyond autophagy inhibition alone, indicating that SCARB1 depletion–induced cell death is primarily mediated by autophagy and apoptosis, as shown in **Figure R14**.

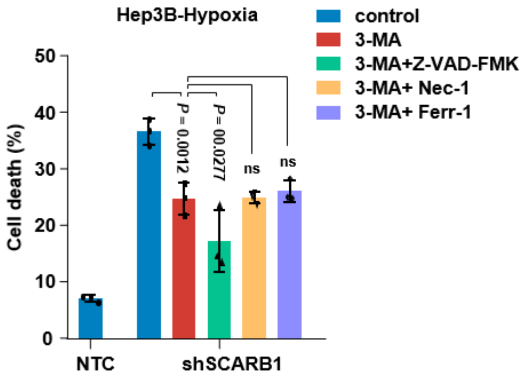

Figure R15. Analysis of the death of Hep3B cells expressing shNTC or shSCARB1 and treated with 2 mM 3-MA alone, 2 mM 3-MA and 5 μM Z-VAD-FMK, or 2 μM Nec-1, or 2 μM Ferr-1 under hypoxia for 48 h**.**

15. Fig. 4A - Quantification:

Add band-intensity quantification (densitometry) for p-S6K and LC3-II to support representative blots.

Response: Thank you for the suggestion. We have performed densitometric quantification of p-ULK1, p-S6K, p-4EBP1, and LC3-II from these immunoblots. Please see **Figure R10**.

16. Integration with TFEB literature:

Incorporate a short paragraph in the Discussion reconciling your model (lactate → SCARB1 → mTORC1 → autophagy suppression) with studies showing lactate- or lactylation-mediated TFEB activation. Distinguish between early adaptive versus chronic hypoxic responses, or nucleus-cytoplasm regulation differences.

Response: Thank you for the suggestion. We noticed that Huang et al. have reported

histone lactylation can promote TFEB activation and enhance autophagy, highlighting an important role of lactate in lysosomal biogenesis and stress adaptation. Our findings do not contradict these observations, but rather suggest that lactate-mediated regulation of autophagy is highly context-dependent and dynamically regulated during hypoxic stress.

We have added the following paragraph to the *Discussion* section:

The regulatory influence of lactate on autophagy appears to be bidirectional, with lactate promoting autophagy in certain contexts, while inhibiting it in others. A recent study revealed that lactate promotes TFEB activation and enhances autophagy in pancreatic cancer (Huang et al, 2024); however, Meng et al. demonstrated that lactate-induced DCBLD1 lactylation suppresses its ubiquitination, resulting in autophagy inhibition and ultimately promoting cervical cancer progression (Meng et al, 2024). Therefore, the seemingly opposite effects of lactate-driven lactylation on autophagy may largely depend on the specific target proteins being modified. In our study, we report that lactate upregulates SCARB1 expression to enhance cholesterol uptake, thereby inhibiting autophagy, consistent with the fact that cholesterol has been shown to activate mTORC1 and suppress autophagy.

Overall Assessment

The manuscript provides an appealing conceptual advance linking lactate-driven epigenetic reprogramming to cholesterol-dependent signaling in hypoxic HCC. Addressing the points above-particularly mechanistic validation of H3K18la transcriptional control, flux and replication rigor, and contextual reconciliation with prior autophagy literature-will substantially enhance the clarity, rigor, and translational relevance of this work.

With these improvements, the study would merit strong consideration for publication in EMBO Reports.

Response: We sincerely thank the referee for this positive and encouraging overall assessment of our work, and we are pleased that the referee finds our study provides an appealing conceptual advance.

We have undertaken extensive revisions to address all points raised, with

particular emphasis on strengthening the mechanistic validation, experimental rigor, and contextual clarity as suggested. We are grateful for the referee's constructive guidance, which has greatly improved the quality of the manuscript. We believe these additions significantly strengthen the manuscript, and we hope that the revised version will now meet the standards for publication in *EMBO Reports*.

Referee #3:

In this manuscript the authors showed data that under hypoxia the cholesterol transporter SCARB1 is elevated to increase cholesterol uptake. They suggest that lactate produced under hypoxia is responsible for the elevation of SCRAB1 expression. They showed that histone H3K18 is lactylated at the SCRAB1 promoter, which could explain the transcriptional elevation of SCRAB1. Further they showed that SCRAB1 depletion impairs the proliferation and tumorigenesis of HCC cells. The authors claim that SCRAB1 is required for the activation of mTORC1 and inhibition of autophagy under hypoxia. However, it is known that hypoxia inhibits mTORC1 through the expression of REDD1. In addition, the authors showed that induction of SCRAB1 by hypoxia is not sufficient to activate mTORC1 and inhibits autophagy questioning the physiological significance of SCRAB1 overexpression. The authors also did not show the mechanism by which SCRAB1 and cholesterol activate mTORC1. Overall, there are several conceptual and specific concerns listed below:

Conceptual concerns:

Response: We thank the referee for the thorough and constructive overall assessment of our manuscript. We appreciate the careful consideration of our findings and the thoughtful points raised regarding the physiological relevance and mechanistic aspects of SCARB1 in hypoxic HCC. We have carefully addressed each of the specific concerns in our point-by-point responses below, including additional experiments and clarifications where appropriate, which we believe strengthen the clarity and significance of our study.

1. *The authors suggest that autophagy induced by mTORC1 inhibition is causing cell death.*

However, autophagy is in fact promoting survival under nutrient stress. Indeed, inhibition of mTORC1 and activation of autophagy by rapamycin does not induce cell death but rather attenuates cell proliferation.

Response: We thank the referee for this important comment. We fully agree that autophagy generally promotes cancer cell survival under nutrient stress. Importantly, the regulation of autophagy by hypoxic stress is dynamic and context-dependent. During the early phase of hypoxia, autophagy indeed serves a protective role by clearing damaged mitochondria and misfolded proteins. However, under chronic hypoxic conditions, sustained and excessive autophagy may lead to over-degradation of essential cellular components, which could compromise cell viability. Tumor cells need mechanisms to balance survival with continued growth and proliferation. Our findings propose that SCARB1-mediated mTORC1 activation provides a modulatory "brake" on autophagy under prolonged hypoxia, helping to prevent excessive self-digestion and maintain a balance between survival and proliferation. In this way, SCARB1 appears to contribute to tumor adaptation to chronic low-oxygen stress, complementing rather than contradicting the classical view of autophagy as a pro-survival mechanism.

We agree with the referee on the point that inhibition of mTORC1 and activation of autophagy by rapamycin does not induce cell death but rather attenuates cell proliferation. We apologize that the wording in the original manuscript was not sufficiently clear and may have caused confusion. Our data do not suggest that rapamycin induces cell death. As shown in Figure 4G, the suppression of autophagy caused by SCARB1 overexpression is largely mediated through the mTORC1 signaling pathway, as pharmacological inhibition of mTORC1 by rapamycin abolished the autophagy-suppressive effect of SCARB1. Consistently, the proliferative advantage conferred by SCARB1 overexpression was also eliminated upon mTORC1 inhibition.

These results indicate that SCARB1 promotes cell proliferation and restrains autophagy in an mTORC1-dependent manner, while rapamycin itself acts in a cytostatic, rather than cytotoxic, fashion in our experimental system. We have revised

the text accordingly to avoid overinterpretation of autophagy induction by rapamycin as a direct cause of cell death.

2. Although depletion of SCRAB1 under hypoxia inhibits mTORC1 and induces autophagy (Fig. 4A), the elevated expression of SCRAB1 under hypoxia is not sufficient to prevent inhibition of mTORC1 and activation of autophagy under hypoxia (Fig. 3F and Fig. 4C). Therefore, the effect of physiological levels of SCARB1 on mTORC1 and autophagy under hypoxia are questionable.

Response: We thank the referee for this comment. We agree that hypoxia broadly suppresses mTORC1 activity and induces autophagy, and that the physiological upregulation of SCARB1 under hypoxia is not sufficient to fully override this global inhibitory effect. Importantly, our data do not suggest that SCARB1 completely prevents hypoxia-induced mTORC1 inhibition. Rather, we propose that SCARB1 functions as a modulatory factor that partially restores mTORC1 activity, thereby conferring a growth advantage under hypoxic stress.

Consistent with this model, depletion of SCARB1 under hypoxia further suppresses mTORC1 activity and exacerbates autophagy, whereas SCARB1 upregulation mitigates, but does not abolish, hypoxia-induced mTORC1 inhibition (**Figure R10**). We therefore interpret SCARB1 as providing a compensatory or buffering signal that restrains excessive autophagy when mTORC1 is globally inhibited by hypoxia. This partial restoration of mTORC1 activity is sufficient to influence autophagy and cell survival, without contradicting the well-established inhibitory effect of hypoxia on mTORC1 signaling.

Specific points:

1. *The authors need to conduct ECAR and show how much lactate is elevated under hypoxia.*

Response: We thank the referee for the suggestion. Accordingly, we have performed additional experiments and measured ECAR and intracellular lactate levels in both Hep3B and PLC cells under normoxia and hypoxia. The results show that hypoxia led to an elevated extracellular acidification rate and a 2- to 3-fold increase in intracellular lactate levels (**Figure R16**). These results support the role of lactate in

promoting SCARB1 expression.

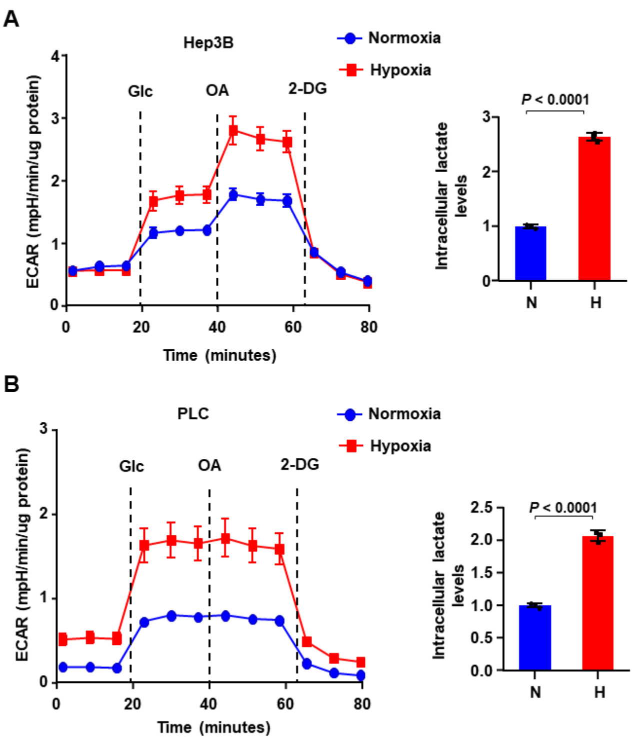

Figure R16. (A-B) The extracellular acidification rate (ECAR) was measured in Hep3B and PLC cells with hypoxia and normoxia treatment for 24 h. Intracellular lactate content was measured in Hep3B and PLC cells with hypoxia and normoxia treatment for 48 h.

2. The authors need to show level and localization of SCARB1 by IF.

Response: We thank the referee for the suggestion. We thus attempted immunofluorescence using several commercially available antibodies from different sources against endogenous SCARB1, but the signal was insufficient for reliable detection. Therefore, we performed immunofluorescence using an anti-Flag antibody in overexpressed SCARB1 cells. The results indicate that SCARB1 is primarily localized to the plasma membrane (**Figure R17**). We noticed, however, that IF using the Flag tag cannot provide accurate quantification of endogenous SCARB1 levels; therefore, SCARB1 expression was evaluated by immunoblotting and qPCR as shown in **Figure 1E** in the original manuscript.

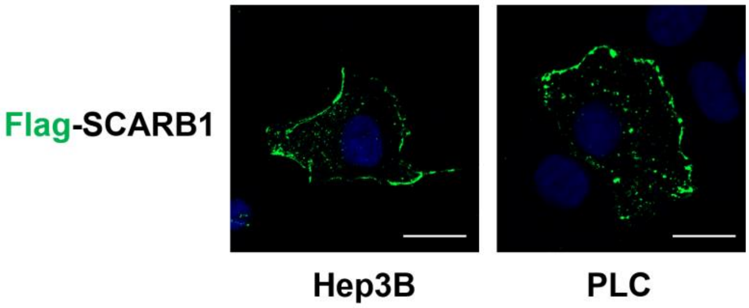

Figure R17. SCARB1 subcellular localization in Hep3B and PLC cells overexpressing SCARB1 was examined by immunofluorescence confocal microscopy. Scale bar: 20 μm.

3. *Fig. 1F, G- It appears that the Induced level of SCARB1 protein is much higher than the elevation of Scrab1 mRNA (especially in Fig. 1G) suggesting that there is a posttranscriptional effect on the level of SCRAB1 protein.*

Response: We thank the referee for pointing this out. To assess whether sodium lactate regulates SCARB1 at the post-transcriptional level, we treated cells with cycloheximide (CHX) to inhibit protein synthesis and monitored SCARB1 protein levels over time (**Figure R18A**). The results showed a gradual decrease in SCARB1 protein, indicating that protein stability is not substantially affected. This suggests that the lactate-induced increase in SCARB1 protein primarily reflects transcriptional upregulation. To further strengthen this conclusion, we increased the sample size and have updated the original **Figure 1G** with **Figure R18B** in the revised manuscript.

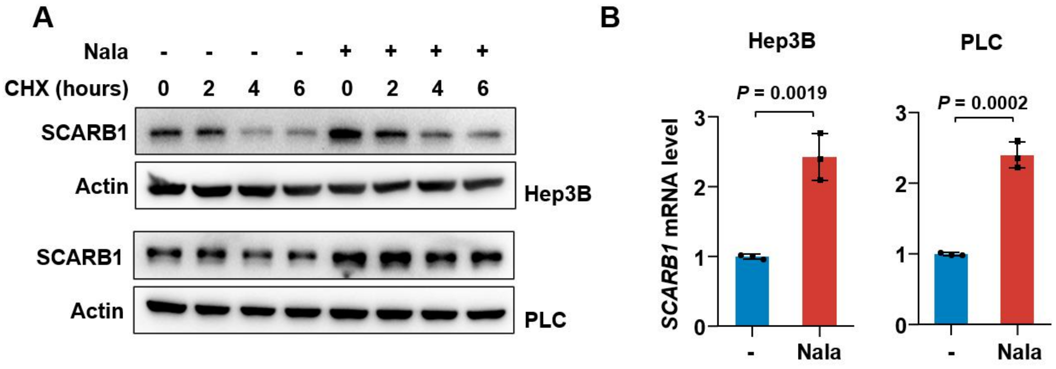

Figure R18. (A) Hep3B and PLC cells are treated with Cycloheximide (CHX) for 0-6 hours. SCARB1 expression was detected by Western blotting. **(B)** Western blot analyses of SCARB1 expression in Hep3B and PLC cells treated with 20 mM sodium lactate (Nala) for 48 h. Please also see **Figure 1G** in the revised manuscript.

4. Unlike the authors' assessment SCARB1 is required for cell proliferation both under hypoxia and normoxia (Fig. EV3A). It is not clear why overexpression of SCRAB1 increases proliferation only under hypoxia as under these conditions it is elevated anyway.

Response: We thank the referee for raising this important point. Under normoxia, hepatocellular carcinoma cells retain active de novo cholesterol biosynthesis, and intracellular cholesterol can be sufficiently supplied by a combination of endogenous synthesis and basal uptake. Consequently, additional SCARB1 overexpression does not further enhance proliferation.

In contrast, under hypoxic stress, cholesterol biosynthetic pathways are suppressed, rendering tumor cells more dependent on exogenous cholesterol uptake. In this context, upregulation or overexpression of SCARB1 facilitates increased cholesterol uptake, thereby preventing prolonged mTORC1 inhibition. Activation of mTORC1 is well established to promote cell growth and proliferation by enhancing anabolic processes and restraining excessive autophagy. Therefore, maintenance of partial mTORC1 activity rather than consistent suppression is particularly critical for sustaining tumor cell proliferation and survival under hypoxic stress.

5. Fig. 3C suggests that the effect of SCARB1 depletion on proliferation is mostly due to cell death (about 30% cell death). The authors should determine cell death also under normoxic conditions.

Response: We thank the referee for the suggestion. In the original manuscript, we conducted the rescue experiment only under hypoxia. Following the referee's suggestion, we performed additional experiments with the inclusion of a normoxic control (**Figure R19**). The results show that SCARB1 knockdown induces cell death under normoxia, and that cell death is further exacerbated under hypoxia, highlighting the critical role of SCARB1 in hypoxic adaptation. We have replaced the original **Figure 3C** with this data in the revised manuscript.

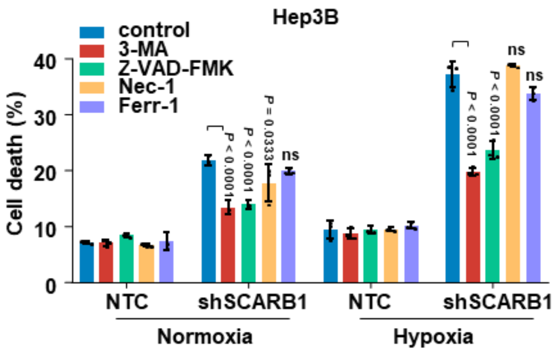

Figure R19. Analysis of the death of Hep3B cells expressing shNTC or shSCARB1 and treated with 2 mM 3-MA, 5 μM Z-VAD-FMK, 2 μM Nec-1, or 2 μM Ferr-1 under normoxia and hypoxia for 48 h. Please also see **Figure 3C** in the revised manuscript.

6. To clarify the effect of SCARB1 on cell proliferation versus cell death the authors should measure BrdU incorporation or Ki67 staining and cell death markers.

Response: We thank the referee for the suggestion. To assess the effect of SCARB1 on cell proliferation, we performed EdU incorporation assays (**Figure R20**). The results show that SCARB1 knockdown slightly reduces DNA synthesis, indicating that SCARB1 can influence cell proliferation. However, as shown in Figure R19, the extent of cell death upon SCARB1 knockdown was substantially greater than its effect on proliferation. Therefore, we subsequently focused on investigating the role of SCARB1 in regulating cell survival and death.

In addition, following the recommendation of referee #2, we examined relevant cell death markers, including markers of apoptosis (cleaved CASP3), necroptosis (phosphorylated MLKL), LC3-II (autophagy), and ferroptosis (GPX4) (**Figure R14**). We found that SCARB1 knockdown not only enhances autophagy but also promotes apoptosis, further supporting its critical role in hepatocellular carcinoma cell survival.

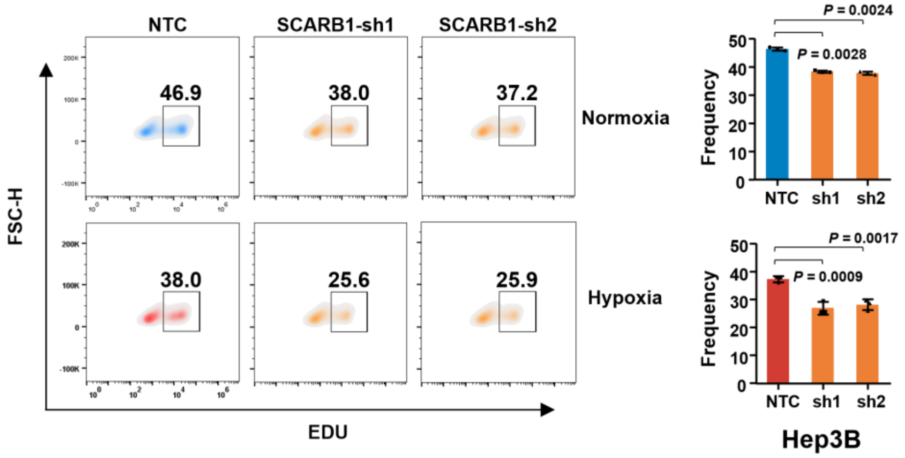

Figure R20. EdU incorporation assay analyzed by flow cytometry in Hep3B cells with SCARB1 knockdown under normoxia and hypoxia.

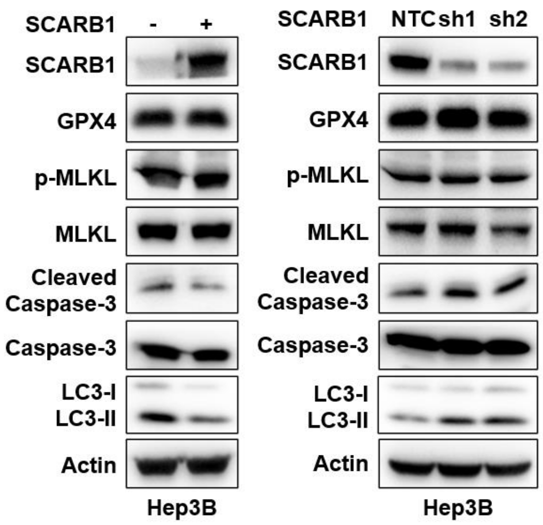

Figure R14. Western blot analysis of GPX4, phosphorylated MLKL, MLKL, cleaved caspase-3, Caspase3, and LC3-I/II in Hep3B cells with SCARB1 overexpression or knockdown under hypoxia. Please also see **Figure EV3C** in the revised manuscript.

7. It is known that hypoxia inhibits mTORC1 by inducing the expression of REDD1. How is SCARB1 related to that? Does it override the effect of REDD1?

Response: We thank the referee for this insightful comment. To dissect the relationship between SCARB1 and REDD1 in regulating mTORC1, we performed single- and combined-knockdown experiments. SCARB1 knockdown reduced p-S6K, whereas REDD1 knockdown increased p-S6K as expected (**Figure R21**). Notably, simultaneous knockdown of SCARB1 in the context of REDD1 depletion still

markedly suppressed p-S6K levels. This result indicates that SCARB1 and REDD1 regulate mTORC1 activity through relatively independent mechanisms. Under hypoxic stress, multiple signaling pathways converge on mTORC1. Although REDD1 functions as a negative regulator, SCARB1 provides a positive input via cholesterol supply.

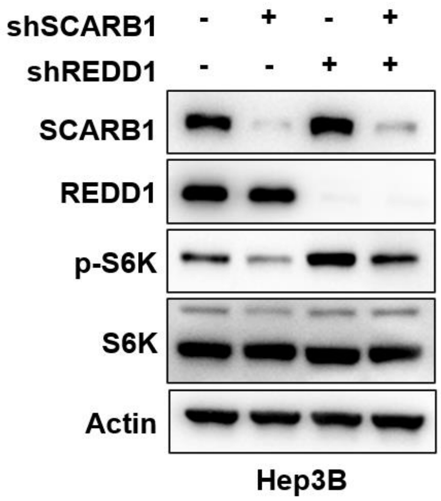

Figure R21. The protein levels of REDD1, SCARB1, and p-S6K in Hep3B cells expressing NTC or shSCARB1 with or without further REDD knockdown.

8. Fig. 4A - The authors should conduct the experiment under normoxia. It is not clear from the WB if mTORC1 is inhibited under hypoxia.

Response: We thank the referee for this helpful suggestion. To clarify whether mTORC1 activity is inhibited under hypoxia, we included an additional normoxic non-targeting control (NTC). As suggested by Referee #2, we also examined additional mTORC1 readouts, including the phosphorylation of 4EBP1 and ULK1, and performed densitometric quantification of p-ULK1, p-S6K, p-4EBP1, and LC3-II from these immunoblots (**Figure R10A)**. We have replaced the original **Figure 4A** with the updated data. The revised immunoblots clearly show that mTORC1 activity is inhibited under hypoxia, which is consistent with the results shown in **Figure R10B**. The results showed that SCARB1 knockdown further suppressed mTORC1 signaling under hypoxia, indicating that loss of SCARB1 leads to excessive inhibition of mTORC1, thereby removing the physiological brake on autophagy and promoting

excessive autophagy activation.

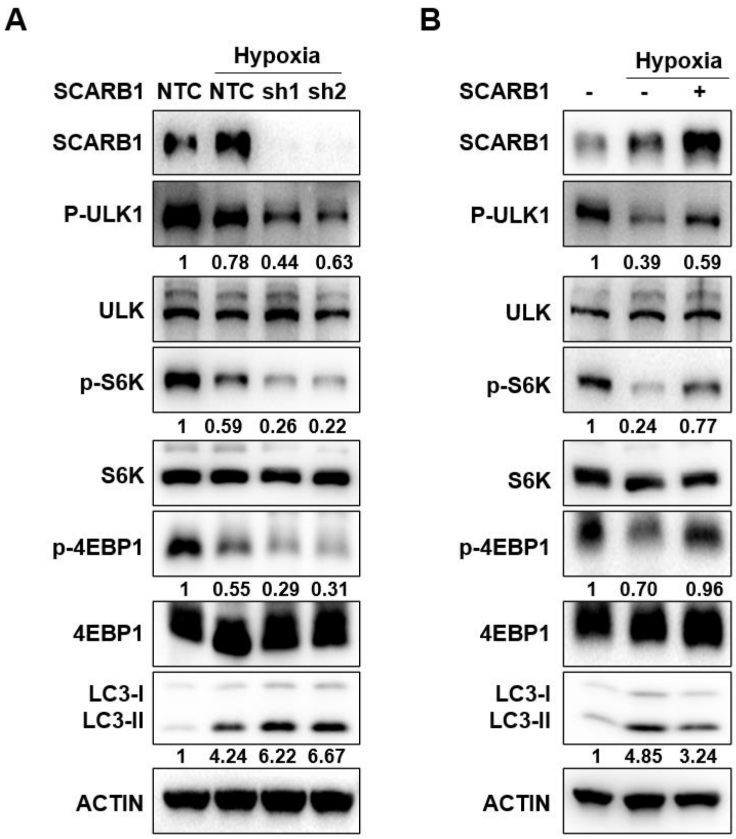

Figure R10. (A, B) Western blot analysis of p-ULK1, ULK1, p-S6K, S6K, p-4EBP1, 4EBP1, and LC3I/II protein expression in Hep3B cells with SCARB1 knockdown or overexpression. Please also see **Figure 4A-B** in the revised manuscript.

9. Fig. 5F- The authors should have H3 and S6K on the immunoblot.

Response: Thank you for pointing this out. We have now added H3 and S6K in **Figure 5F** in the revised manuscript.

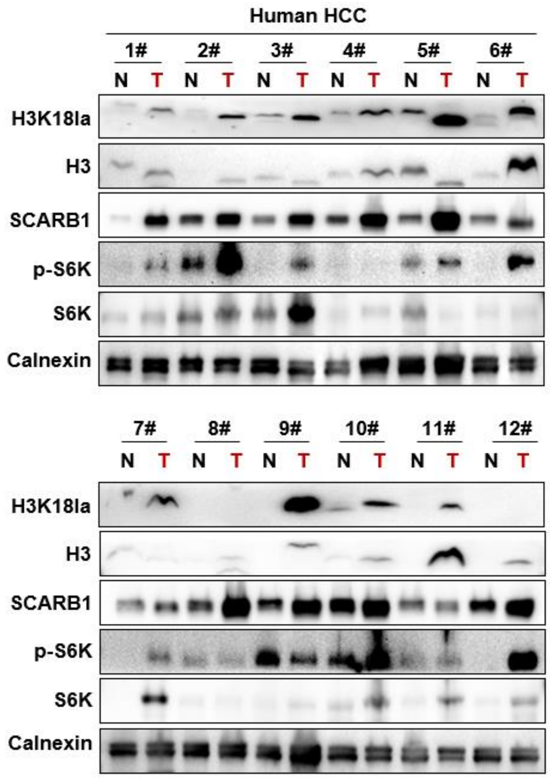

Figure R22. Western blot analysis of H3K18la, H3, SCARB1, p-S6K, and S6K expression in paired tumour-adjacent noncancerous liver tissues and human HCC tissues. Please also see **Figure 5F** in the revised manuscript.

10. Fig. EV4B- Inconsistent with earlier results overexpression of SCRAB1 did not affect mTORC1 and autophagy in normal medium.

Response: We thank the referee for pointing this out. We have increased the sample size and repeated the experiments three times (**Figure R23)**. The results show that SCARB1 overexpression effectively activates mTORC1 and suppresses autophagy, consistent with our previous conclusions. The representative 1st experiment result was used in the manuscript (**Figure EV4C)**.

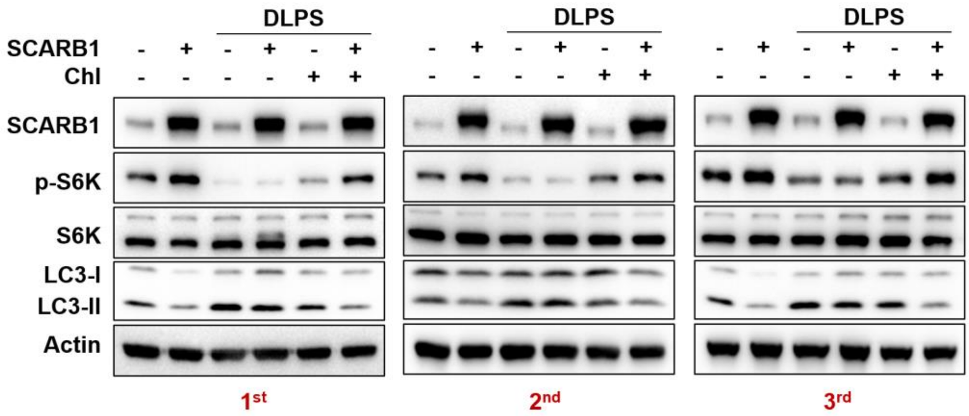

Figure R23. PLC cells expressing SCARB1 were cultured in 10% FBS or 10% delipidated serum (DLPS) in the absence or presence of 10 μg/mL cholesterol (chl) under hypoxia. SCARB1, p-S6K, S6K, and LC3-I/II protein levels were detected by Western blotting.

11. Fig. EV4C- If comparing pS6K/S6K, mTORC1 activity is much higher in regular medium than in DLPS + Cholesterol, yet autophagy is inhibited.

Response: We thank the referee for pointing this out. We thank the referee for pointing this out. Due to technical issues, the S6K band in the first lane of the original experiment was unclear. We have therefore repeated the experiment three times (**Figure R24)**. The results consistently show that cholesterol effectively activates mTORC1, leading to suppression of autophagy. The representative 1st experiment result was used in the manuscript (**Figure EV4D)**.

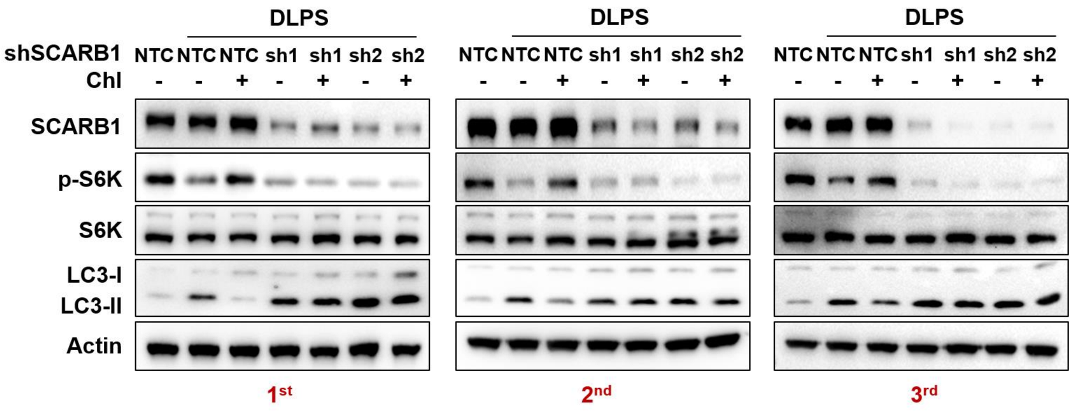

Figure R24. PLC cells with SCARB1 knockdown were cultured in 10% FBS or 10% delipidated serum (DLPS) in the absence or presence of 10 μg/mL cholesterol (chl) under hypoxia. SCARB1, p-S6K, S6K, and LC3-I/II protein levels were detected by Western blotting.

References:

Faubert B., Li K.Y., Cai L., Hensley C.T., Kim J., Zacharias L.G., Yang C., Do Q.N., Doucette S., Burguete D., et al.(2017) Lactate Metabolism in Human Lung Tumors. *Cell*, 171(2): 358-371.e359.

Guillaumond F., Leca J., Olivares O., Lavaut M.N., Vidal N., Berthezène P., Dusetti N.J., Loncle C., Calvo E., Turrini O., et al.(2013) Strengthened glycolysis under hypoxia supports tumor symbiosis and hexosamine biosynthesis in pancreatic adenocarcinoma. *Proceedings of the National Academy of Sciences of the United States of America*, 110(10): 3919-3924.

Hensley C.T., Faubert B., Yuan Q., Lev-Cohain N., Jin E., Kim J., Jiang L., Ko B., Skelton R., Loudat L., et al.(2016) Metabolic Heterogeneity in Human Lung Tumors. *Cell*, 164(4): 681-694.

Huang Y., Luo G., Peng K., Song Y., Wang Y., Zhang H., Li J., Qiu X., Pu M., Liu X., et al.(2024) Lactylation stabilizes TFEB to elevate autophagy and lysosomal activity. *The Journal of cell biology*, 223(11).

Kennedy K.M., Scarbrough P.M., Ribeiro A., Richardson R., Yuan H., Sonveaux P., Landon C.D., Chi J.T., Pizzo S., Schroeder T., et al.(2013) Catabolism of exogenous lactate reveals it as a legitimate metabolic substrate in breast cancer. *PloS one*, 8(9): e75154.

Liu X., Bian H., Shi Y., Du T.(2025) Scavenger receptor class B member 1 promotes lung cancer growth and metastasis through enhanced twist family BHLH transcription factor 1 signaling in vitro and in vivo: Exploration of RPPNs as a therapeutic Strategy. *CytoJournal*, 2223.

Meng Q., Sun H., Zhang Y., Yang X., Hao S., Liu B., Zhou H., Xu Z.X., Wang Y.(2024) Lactylation stabilizes DCBLD1 activating the pentose phosphate pathway to promote cervical cancer progression. *Journal of experimental & clinical cancer research : CR*, 43(1): 36.

Riscal R., Bull C.J., Mesaros C., Finan J.M., Carens M., Ho E.S., Xu J.P., Godfrey J., Brennan P., Johansson M., et al.(2021) Cholesterol Auxotrophy as a Targetable Vulnerability in Clear Cell Renal Cell Carcinoma. *Cancer discovery*, 11(12): 3106-3125.

Dear Dr. Zhang

Thank you for the submission of your revised manuscript to EMBO reports. We have now received the full set of referee reports that is copied below.

As you will see, all referees are very positive about the study and request only minor changes to clarify text and figures.

From the editorial side, there are also a few things that we need before we can proceed:

- As a standard procedure, we edit the title and abstract of manuscripts to make them more accessible to a general readership. Please find the suggested versions below my signature.

- Please mark the corresponding author(s) and provide their institutional email addresses on the title page.

- Please move the data availability statement to after the Acknowledgements section.

- Please state employment in a biotech company in the Disclosure and competing interests statement. In particular, please let us know whether affiliation #5 is a biotech company.

- As we are switching from a free-text author contribution statement towards a more formal statement based on Contributor Role Taxonomy (CRediT) terms, please remove the present Author Contribution section and instead specify each author's contribution(s) directly in the Author Information page of our submission system during upload of the final manuscript. See https://casrai.org/credit/ for more information.

- Please remove the Tianfu Jincheng Laboratory (No. TFJCPI20250001) from the Comments box in the system and provide is as a separate funder (via More Funders option).

- Please include the reference number for the animal ethics approving body.

- Please include a statement on blinding in the Methods section, even if no blinding was performed.

- Please provide information on human patients, particularly on ethics considerations, in the Author checklist and Methods section.

- Please adjust the in-text callouts for individual figures: e.g. Figure 6 appears to not be called out in the text.

- Please note that EMBO press papers are accompanied online by:
A) a short (2 sentences) summary of the findings and their significance,
B) 2-5 short bullet points highlighting the key results, and
C) a synopsis image in .jpg or .png format that is exactly 550 pixels wide and 300 pixels high. Please note that the text needs to be legible at the final size. Please upload this information along with your revised manuscript (the text for A and B should be provided in one separate Word file uploaded as Cover Art/Synopsis Image).

- Please provide the URL for panel 5I in the data availability statement since the source data checklist is not published.

- Can you please explain the repeated values in the attached source data files?

- Our production/data editors have asked you to clarify several points in the figure legends (see below). Please incorporate these changes in the manuscript and return the revised file with tracked changes with your final manuscript submission.
Please note that the exact p values are not provided in the legends of figures 1A-C, E, F, G, I, J; 2D, E; 3A, B, C, H; 4G, 5B, D, E, H; 6A-D; EV3 A, EV4 E, EV5 D

Kurt Weir
Editor
EMBO Reports

Lactate-mediated cholesterol uptake promotes liver cancer progression via the SCARB1-autophagy axis

Metabolic reprogramming, including enhanced glycolysis and altered fatty acid metabolism, supports the proliferation of cancer cells under hypoxic stress. However, the mechanism underlying the regulation of cholesterol metabolism under hypoxic stress remains incompletely understood. Here, we report that lactate-induced cholesterol accumulation activates mammalian target of rapamycin complex 1 (mTORC1) signalling under hypoxic conditions, thereby promoting hepatocellular carcinoma (HCC)

progression. Mechanistically, lactate upregulates scavenger receptor class B type 1 (SCARB1) expression by increasing histone H3 lysine 18 lactylation (H3K18la), leading to increased cholesterol levels. We further demonstrate that SCARB1-mediated cholesterol uptake is essential for the activation of mTORC1, which promotes tumour growth by preventing excessive autophagy in HCC cells. Importantly, analysis of clinical HCC samples reveals a positive correlation between H3K18la expression and SCARB1 expression. Taken together, these findings provide novel insights into hypoxia-driven metabolic reprogramming and reveal a previously unrecognized connection between lactate and cholesterol metabolism, suggesting a potential innovative cancer therapy for HCC.

Referee #1:

Final Review Comments

Lactate-mediated cholesterol uptake promotes liver cancer progression through the SCARB1-autophagy axis

By Jiamin Chen et. Al.

Thank you to the authors for their thorough and detailed responses to the reviewers' comments.
The study remains well-structured, focused, and coherent in scope. The authors present solid and convincing data supporting the role of SCARB1 in the progression of liver cancer, particularly in the context of lactylation and autophagy. Overall, the experimental evidence and the revised sections of the manuscript are strong. In our view, the work is of significant value and is suitable for publication in the journal.
We have only one minor comment:

• The frequent use of acronyms occasionally affects the readability of the manuscript. In several instances throughout the text, acronyms are not clearly defined upon first use. We recommend ensuring that all acronyms are properly introduced and explained when first mentioned. Alternatively, the authors may consider including a dedicated table (e.g., in the appendix or as supplementary material) listing all acronyms and their full definitions to improve clarity and accessibility for readers.

Referee #2:

All the major and minor issues raised in the manuscript have been addressed satisfactorily by the authors. The revisions have improved the scientific clarity, rigor, and presentation of the manuscript.
I believe that the manuscript is ready for publication, and I do not have any further comments.

Referee #3:

The authors have addressed most of my previous comments. The authors should state that SCRAB1 is required for cell proliferation (Fig. EV3A) and cell survival even under normoxia.

Point-by-point response to the comments

Editor's Decision:

Thank you for the submission of your revised manuscript to EMBO reports. We have now received the full set of referee reports that is copied below.

As you will see, all referees are very positive about the study and request only minor changes to clarify text and figures.

From the editorial side, there are also a few things that we need before we can proceed:

- As a standard procedure, we edit the title and abstract of manuscripts to make them more accessible to a general readership. Please find the suggested versions below my signature.

Response: We thank the editor for this helpful suggestion and have adopted the revised title and abstract accordingly.

- Please mark the corresponding author(s) and provide their institutional email addresses on the title page.

Response: We have now indicated the three corresponding authors and provided their institutional email addresses on the title page, as requested.

- Please move the data availability statement to after the Acknowledgements section.

Response: We have moved the data availability statement to after the Acknowledgements section as requested.

- Please state employment in a biotech company in the Disclosure and competing interests statement. In particular, please let us know whether affiliation #5 is a biotech company.

Response: We thank the editor for this query. We would like to clarify that affiliation #5 is a research institute rather than a biotech company, and therefore, no related disclosure is applicable.

- As we are switching from a free-text author contribution statement towards a more formal statement based on Contributor Role Taxonomy (CRediT) terms, please remove the present Author Contribution section and instead specify each author's contribution(s) directly in the Author

Information page of our submission system during upload of the final manuscript. See https://casrai.org/credit/ for more information.

Response: We thank the editor for detailed guidance. We have removed the free-text *Author Contribution* section from the manuscript and have provided the contributions using the CRediT taxonomy in the submission system.

- Please remove the Tianfu Jincheng Laboratory (No. TFJCPI20250001) from the Comments box in the system and provide is as a separate funder (via More Funders option).

Response: We have removed this funder from both the Comments box and the manuscript, as we could not locate it in the system via the "More Funders" option.

- Please include the reference number for the animal ethics approving body.

Response: Thank you. We have added the animal ethics approval number (AEC number) in the *Methods* section of the manuscript, as requested.

- Please include a statement on blinding in the Methods section, even if no blinding was performed.

Response: Thank you for pointing this out. We have added the following blinding statement to the *Methods* section as requested:

"Data collection and analysis were not performed blind to the conditions of experiments, except for the acquisition and analysis of immunohistochemistry and immunofluorescence images, which were performed in a blinded manner."

- Please provide information on human patients, particularly on ethics considerations, in the Author checklist and Methods section.

Response: Thank you for the reminder. We have now provided detailed information on all relevant human subjects in Appendix Table S1. A corresponding statement has been added in the *Methods* section. We have also updated the Author checklist accordingly.

- Please adjust the in-text callouts for individual figures: e.g. Figure 6 appears to not be called out in the text.

Response: We thank the editor for pointing this out. Figure 6 was originally intended as a schematic summary of the study but was not included in the previous submission. We have now removed Figure 6 from the figure legend and the figure checklist to ensure that all in-text figure callouts are consistent with the revised figures. Instead, we have uploaded it as a synopsis image in the submission system.

- Please note that EMBO press papers are accompanied online by:

A) a short (2 sentences) summary of the findings and their significance,

B) 2-5 short bullet points highlighting the key results, and

C) a synopsis image in .jpg or .png format that is exactly 550 pixels wide and 300 pixels high. Please note that the text needs to be legible at the final size. Please upload this information along with your revised manuscript (the text for A and B should be provided in one separate Word file uploaded as Cover Art/Synopsis Image).

Response: Thank you for the detailed guidance. We have prepared all three items as requested, and all files have been uploaded in the submission system. Please see the image file "Cover Art/Synopsis Image" and word file "blurb and highlights".

- Please provide the URL for panel 5I in the data availability statement since the source data checklist is not published.

Response: Thank you for the suggestion. We have added the following information to the *Data Availability* section:

"The survival analysis was performed using the GEPIA database (http://gepia.cancer-pku.cn/) with the following parameters: gene = SCARB1, cancer type = LIHC, and median expression cutoff for grouping."

- Can you please explain the repeated values in the attached source data files?

Response: Thank you for pointing this out. We have carefully reviewed these data and confirmed that these repeated values arise from the data processing procedure itself rather than from data duplication or error. We have verified that these instances do not materially affect the statistical analyses or the conclusions of the study.

In qPCR analyses, due to normalization using the $2^{\wedge-(\Delta\Delta Ct)}$ method, even when the original Ct values are not identical, mathematical transformation can result in identical values across multiple decimal points.

Similarly, in cholesterol quantification assays, when raw measurements are identical or very close, applying the same calculation formula can also yield identical values across multiple decimal places.

For Figure 4H, during cell counting, some wells yielded identical cell counts. When calculating the total cell number based on the average cell count of grids and the resuspension volume, decimal points can appear in the calculated results. We confirm that the raw counts are integers and the calculations are accurate.

For Figure 5B, we have re-examined the raw data. The identical tumor weights within the same group resulted from the limited precision of the balance used (reading to 0.01g) and the fact that tumors from the same treatment group were very similar in size. We confirm that these data are authentic, individual measurements and not data duplication.

In addition, we have re-examined all the raw data and found that in the PLC data of Figures EV3A and EV3B, the cell seeding number was incorrectly recorded as 3×10^4 but should have been 4×10^4. We have corrected this error in the source data files.

- Our production/data editors have asked you to clarify several points in the figure legends (see below). Please incorporate these changes in the manuscript and return the revised file with tracked changes with your final manuscript submission.

Please note that the exact *p* values are not provided in the legends of figures 1A-C, E, F, G, I, J; 2D, E; 3A, B, C, H; 4G, 5B, D, E, H; 6A-D; EV3 A, EV4 E, EV5 D

Response: Thank you. We have now added the exact *p* values in the legends of all the figures mentioned.

Reviewers' comments

Referee #1:

Final Review Comments

Lactate-mediated cholesterol uptake promotes liver cancer progression through the SCARB1-autophagy axis

By Jiamin Chen et. Al.

Thank you to the authors for their thorough and detailed responses to the reviewers' comments.
The study remains well-structured, focused, and coherent in scope. The authors present solid and convincing data supporting the role of SCARB1 in the progression of liver cancer, particularly in the context of lactylation and autophagy. Overall, the experimental evidence and the revised sections of the manuscript are strong. In our view, the work is of significant value and is suitable for publication in the journal.
We have only one minor comment:
• The frequent use of acronyms occasionally affects the readability of the manuscript. In several instances throughout the text, acronyms are not clearly defined upon first use. We recommend ensuring that all acronyms are properly introduced and explained when first mentioned. Alternatively, the authors may consider including a dedicated table (e.g., in the appendix or as supplementary material) listing all acronyms and their full definitions to improve clarity and accessibility for readers.
Response: We thank the referee for the positive feedback and constructive suggestions. We have carefully revised the manuscript and ensured that all acronyms are clearly defined upon first use. Additionally, we have prepared a table listing all acronyms and their full definitions, which has been included in Appendix Table S2 to improve readability.

Referee #2:

All the major and minor issues raised in the manuscript have been addressed satisfactorily by the authors. The revisions have improved the scientific clarity, rigor, and presentation of the manuscript. I believe that the manuscript is ready for publication, and I do not have any further comments.
Response: We thank the referee for the positive evaluation of our manuscript and for the constructive comments that have helped improve the manuscript.

Referee #3:

The authors have addressed most of my previous comments. The authors should state that SCRAB1 is required for cell proliferation (Fig. EV3A) and cell survival even under normoxia.

Response: We are grateful for the referee's suggestion. As shown in the revised *Results* section, we have explicitly stated that SCARB1 is required for HCC cell proliferation and survival under both normoxia and hypoxia. The relevant text has been updated accordingly.

2nd Revision - Editorial Decision 22nd May 2026

Tong Zhang
University of Science and Technology of China
School of Life Science
Hefei 230027
China

Dear Dr. Zhang,

I am pleased to inform you that your manuscript has been accepted for publication in EMBO reports. Your manuscript will be processed for publication by EMBO Press. It will be copy edited and you will receive page proofs prior to publication. Please note that you will be contacted by Springer Nature Author Services to complete licensing and payment information.

You may qualify for financial assistance for your publication charges - either via a Springer Nature fully open access agreement or an EMBO initiative. Check your eligibility: https://link.springer.com/journal/44319/how-to-publish-with-us

Should you be planning a Press Release on your article, please notify the production team via this form in order to coordinate publication and release dates: https://forms.cloud.microsoft/e/eH4A8JVP6g

If you have any questions, please do not hesitate to contact the Editorial Office. Thank you for your contribution to EMBO Reports.

Yours sincerely,

Kurt Weir
Editor
EMBO Reports

>>> Please note that it is EMBO Reports policy for the transcript of the editorial process (containing referee reports and your response letter) to be published as an online supplement to each paper. If you do NOT want this, you will need to inform the Editorial Office via email immediately. More information is available here: https://link.springer.com/partners/embo-press/editorial-policies#Peer%20review

EMBO Press Author Checklist

Corresponding Author Name: Tong Zhang	
Journal Submitted to: EMBO Reports	
Manuscript Number: EMBOR-2025-62823-T	

USEFUL LINKS FOR COMPLETING THIS FORM
The EMBO Journal - Author Guidelines
EMBO Reports - Author Guidelines
Molecular Systems Biology - Author Guidelines
EMBO Molecular Medicine - Author Guidelines

Reporting Checklist for Life Science Articles (updated January
This checklist is adapted from Materials Design Analysis Reporting (MDAR) Checklist for Authors. MDAR establishes a minimum set of requirements in transparent reporting in the life sciences (see Statement of Task: 10.31222/osf.io/9sm4x). Please follow the journal's guidelines in preparing your
Please note that a copy of this checklist will be published alongside your article.

Abridged guidelines for figures
1. Data
The data shown in figures should satisfy the following conditions:
- the data were obtained and processed according to the field's best practice and are presented to reflect the results of the experiments in an accurate and unbiased manner.
- ideally, figure panels should include only measurements that are directly comparable to each other and obtained with the same assay.
- plots include clearly labeled error bars for independent experiments and sample sizes. Unless justified, error bars should not be shown for technical
- if n<5, the individual data points from each experiment should be plotted. Any statistical test employed should be justified.
- Source Data should be included to report the data underlying figures according to the guidelines set out in the authorship guidelines on Data

2. Captions
Each figure caption should contain the following information, for each panel where they are relevant:
- a specification of the experimental system investigated (eg cell line, species name).
- the assay(s) and method(s) used to carry out the reported observations and measurements.
- an explicit mention of the biological and chemical entity(ies) that are being measured.
- an explicit mention of the biological and chemical entity(ies) that are altered/varied/perturbed in a controlled manner.
- the exact sample size (n) for each experimental group/condition, given as a number, not a range;
- a description of the sample collection allowing the reader to understand whether the samples represent technical or biological replicates (including how many animals, litters, cultures, etc.).
- a statement of how many times the experiment shown was independently replicated in the laboratory.
- definitions of statistical methods and measures:
  - common tests, such as t-test (please specify whether paired vs. unpaired), simple χ2 tests, Wilcoxon and Mann-Whitney tests, can be unambiguously identified by name only, but more complex techniques should be described in the methods section;
  - are tests one-sided or two-sided?
  - are there adjustments for multiple comparisons?
  - exact statistical test results, e.g., P values = x but not P values < x;
  - definition of 'center values' as median or average;
  - definition of error bars as s.d. or s.e.m.

Please complete ALL of the questions below.
Select "Not Applicable" only when the requested information is not relevant for your study.

Materials

Newly Created Materials	Information included in the manuscript?	In which section is the information available? (Reagents and Tools Table, Materials and Methods, Figures, Data Availability Section)
New materials and reagents need to be available; do any restrictions apply?	Yes	Materials and Methods

Antibodies	Information included in the manuscript?	In which section is the information available? (Reagents and Tools Table, Materials and Methods, Figures, Data Availability Section)
For **antibodies** provide the following information:<br>- Commercial antibodies: RRID (if possible) or supplier name, catalogue number and or/clone number<br>- Non-commercial: RRID or citation	Yes	Reagents and Tools Table

DNA and RNA sequences	Information included in the manuscript?	In which section is the information available? (Reagents and Tools Table, Materials and Methods, Figures, Data Availability Section)
**Short novel DNA or RNA including primers, probes**: provide the sequences.	Yes	Reagents and Tools Table

Cell materials	Information included in the manuscript?	In which section is the information available? (Reagents and Tools Table, Materials and Methods, Figures, Data Availability Section)
**Cell lines:** Provide species information, strain. Provide accession number in repository **OR** supplier name, catalog number, clone number, and/**OR** RRID.	Yes	Reagents and Tools Table
**Primary cultures:** Provide species, strain, sex of origin, genetic modification status.	Not Applicable	N/A
Report if the cell lines were recently **authenticated** (e.g., by STR profiling) and tested for mycoplasma contamination.	Not Applicable	N/A

Experimental animals	Information included in the manuscript?	In which section is the information available? (Reagents and Tools Table, Materials and Methods, Figures, Data Availability Section)
**Laboratory animals or Model organisms:** Provide species, strain, sex, age, genetic modification status. Provide accession number in repository **OR** supplier name, catalog number, clone number, **OR** RRID.	Yes	Materials and Methods
**Animal observed in or captured from the field:** Provide species, sex, and age where possible.	Not Applicable	N/A
Please detail **housing and husbandry conditions**.	Yes	Materials and Methods

Plants and microbes	Information included in the manuscript?	In which section is the information available? (Reagents and Tools Table, Materials and Methods, Figures, Data Availability Section)
**Plants:** provide species and strain, ecotype and cultivar where relevant, unique accession number if available, and source (including location for collected wild specimens).	Not Applicable	N/A
**Microbes:** provide species and strain, unique accession number if available, and source.	Not Applicable	N/A

Human research participants	Information included in the manuscript?	In which section is the information available? (Reagents and Tools Table, Materials and Methods, Figures, Data Availability Section)
If collected and within the bounds of privacy constraints report on age, sex and gender or ethnicity for all study participants.	Yes	Materials and Methods

Core facilities	Information included in the manuscript?	In which section is the information available? (Reagents and Tools Table, Materials and Methods, Figures, Data Availability Section)
If your work benefited from core facilities, was their service mentioned in the acknowledgments section?	Not Applicable	N/A

Design

Study protocol	Information included in the manuscript?	In which section is the information available? (Reagents and Tools Table, Materials and Methods, Figures, Data Availability Section)
If study protocol has been **pre-registered, provide DOI in the manuscript**. For clinical trials, provide the trial registration number **OR** cite DOI.	Not Applicable	N/A
Report the **clinical trial registration number** (at ClinicalTrials.gov or equivalent), where applicable.	Not Applicable	N/A

Laboratory protocol	Information included in the manuscript?	In which section is the information available? (Reagents and Tools Table, Materials and Methods, Figures, Data Availability Section)
Provide DOI OR other citation details if **external detailed step-by-step protocols** are available.	Yes	Materials and Methods

Experimental study design and statistics	Information included in the manuscript?	In which section is the information available? (Reagents and Tools Table, Materials and Methods, Figures, Data Availability Section)
Include a statement about **sample size** estimate even if no statistical methods were used.	Yes	Figures
Were any steps taken to minimize the effects of subjective bias when allocating animals/samples to treatment (e.g. **randomization procedure**)? If yes, have they been described?	Yes	Materials and Methods
Include a statement about **blinding** even if no blinding was done.	Yes	Materials and Methods
Describe **inclusion/exclusion criteria** if samples or animals were excluded from the analysis. Were the criteria pre-established?<br><br>If sample or data points were omitted from analysis, report if this was due to attrition or intentional exclusion and provide justification.	Not Applicable	N/A
For every figure, are **statistical tests** justified as appropriate? Do the data meet the assumptions of the tests (e.g., normal distribution)? Describe any methods used to assess it. Is there an estimate of variation within each group of data? Is the variance similar between the groups that are being statistically compared?	Yes	Figure legends

Sample definition and in-laboratory replication	Information included in the manuscript?	In which section is the information available? (Reagents and Tools Table, Materials and Methods, Figures, Data Availability Section)
In the figure legends: state number of times the experiment was **replicated** in laboratory.	Yes	Figure Legends
In the figure legends: define whether data describe **technical or biological replicates**.	Yes	Figure Legends

Ethics

Ethics	Information included in the manuscript?	In which section is the information available? (Reagents and Tools Table, Materials and Methods, Figures, Data Availability Section)
Studies involving **human participants**: State details of **authority granting ethics approval** (IRB or equivalent committee(s), provide reference number for approval.	Yes	Materials and Methods
Studies involving **human participants**: Include a statement confirming that **informed consent** was obtained from all subjects and that the experiments conformed to the principles set out in the WMA Declaration of Helsinki and the Department of Health and Human Services Belmont Report.	Yes	Materials and Methods
Studies involving **human participants:** For publication of **patient photos**, include a statement confirming that consent to publish was obtained.	Not Applicable	N/A
Studies involving experimental **animals**: State details of **authority granting ethics approval** (IRB or equivalent committee(s), provide reference number for approval. Include a statement of compliance with ethical regulations.	Yes	Materials and Methods
Studies involving **specimen and field samples:** State if relevant **permits** obtained, provide details of authority approving study; if none were required, explain why.	Yes	Materials and Methods

Dual Use Research of Concern (DURC)	Information included in the manuscript?	In which section is the information available? (Reagents and Tools Table, Materials and Methods, Figures, Data Availability Section)
Could your study fall under dual use research restrictions? Please check biosecurity documents and list of **select agents and toxins** (CDC): https://www.selectagents.gov/sat/list.htm	Not Applicable	N/A
If you used a select agent, is the security level of the lab appropriate and reported in the manuscript?	Not Applicable	N/A
If a study is subject to dual use research of concern regulations, is the name of the **authority granting approval and reference number** for the regulatory approval provided in the manuscript?	Not Applicable	N/A

Reporting

The MDAR framework recommends adoption of discipline-specific guidelines, established and endorsed through community initiatives. Journals have their own policy about requiring specific guidelines and recommendations to complement MDAR.

Adherence to community standards	Information included in the manuscript?	In which section is the information available? (Reagents and Tools Table, Materials and Methods, Figures, Data Availability Section)
State if relevant guidelines or checklists (e.g., **ICMJE, MIBBI, ARRIVE, PRISMA**) have been followed or provided.	Yes	Materials and Methods
For **tumor marker prognostic studies,** we recommend that you follow the **REMARK** reporting guidelines (see link list at top right). See author guidelines, under 'Reporting Guidelines'. Please confirm you have followed these guidelines.	Not Applicable	N/A
For **phase II and III randomized controlled trials**, please refer to the **CONSORT** flow diagram (see link list at top right) and submit the CONSORT checklist (see link list at top right) with your submission. See author guidelines, under 'Reporting Guidelines'. Please confirm you have submitted this list.	Not Applicable	N/A

Data Availability

Data availability	Information included in the manuscript?	In which section is the information available? (Reagents and Tools Table, Materials and Methods, Figures, Data Availability Section)
Have **primary datasets** been deposited according to the journal's guidelines (see 'Data Deposition' section) and the respective accession numbers provided in the Data Availability Section?	Yes	Materials and Methods
Were **human clinical and genomic datasets** deposited in a public access-controlled repository in accordance to ethical obligations to the patients and to the applicable consent agreement?	Not Applicable	N/A
Are **computational models** that are central and integral to a study available without restrictions in a machine-readable form? Were the relevant accession numbers or links provided?	Not Applicable	N/A
If publicly available data were reused, provide the respective **data citations in the reference list**.	Not Applicable	N/A
